# Breast cancer burden (1990–2040) in individuals aged 55 and above: a GBD 2021 analysis of global trends, sex-specific risk factors, and intervention impact

**DOI:** 10.3389/fpubh.2025.1586497

**Published:** 2025-07-31

**Authors:** Shimao Jian, Wanmei Lin, Yuewen Tang, Guangyu Yao

**Affiliations:** ^1^Department of General Surgery, Breast Center, Nanfang Hospital, Southern Medical University, Guangzhou, China; ^2^State Key Laboratory of Ophthalmology, Guangdong Provincial Key Laboratory of Ophthalmology Visual Science, Zhongshan Ophthalmic Center, Sun Yat-sen University, Guangzhou, China

**Keywords:** breast cancer, middle-aged and older adult people, global burden of disease, scenario analysis, aging population and health care, EAPC, BAPC prediction model

## Abstract

**Background:**

Breast cancer remains a leading cause of death among women, with incidence and mortality rates sharply increasing with age, particularly in individuals aged 55 and above. This study provides a comprehensive assessment of the global breast cancer burden in this high-risk demographic.

**Methods:**

Utilizing Global Burden of Disease (GBD) 2021 study data, we analyzed breast cancer incidence, prevalence, mortality, and disability-adjusted life years (DALYs) for individuals aged 55 and older from 1990 to 2021. Temporal trends were quantified using age-standardized rates (ASRs) and Estimated Annual Percentage Changes (EAPCs). Population Attributable Fractions (PAFs) delineated sex-specific contributions of modifiable risk factors. A Bayesian age-period-cohort (BAPC) model projected the future burden to 2040, rigorously validated by sensitivity analysis. Crucially, an evidence-based scenario analysis was employed to model the potential impact of various public health interventions.

**Results:**

Globally, absolute breast cancer cases and deaths in the 55+ population significantly increased (1990–2021), despite a modest decline in the age-standardized mortality rate. Profound disparities emerged across Socio-Demographic Index (SDI) regions, with low-to-middle SDI regions experiencing sharp increases in incidence and mortality, contrasting with declining standardized mortality in high-SDI regions. Risk factor analysis revealed distinct sex-specific profiles: female DALY burden was predominantly driven by metabolic risks (e.g., high BMI), while male burden was overwhelmingly attributable to high alcohol consumption. Baseline projections to 2040 suggest rate stabilization; however, scenario analysis demonstrated high malleability: enhanced screening could avert millions of cases, whereas pessimistic scenarios (e.g., COVID-19 pandemic disruptions) could reverse current progress.

**Conclusion:**

The breast cancer burden in the aging population is substantial and increasingly concentrated in developing regions. The distinct risk profiles between sexes, combined with the profound potential of targeted interventions, underscore the urgent need for tailored, proactive, and resource-stratified public health strategies to mitigate the projected global burden.

## 1 Introduction

Breast cancer is now the most frequently diagnosed malignancy worldwide, with its burden falling disproportionately on the expanding population of adults aged 55 and over (1, 2). This demographic faces a unique set of challenges, including diagnosis at more advanced stages, higher mortality, and a greater risk of recurrence (3). The complexity of this burden is shaped by distinct risk profiles dominated by metabolic and behavioral factors (4), such as hyperglycemia, obesity, and a sedentary lifestyle, which are closely associated with adverse prognoses (5). This is compounded by profound global disparities that create a stark “health divide” (6): while mortality rates decline in high-income nations due to advanced healthcare systems, many developing regions face the dual challenge of rising incidence and mortality (2). Adding a final layer of complexity is the often-overlooked upward trend of male breast cancer, which exhibits regional variations and has etiological drivers that differ significantly from those in females (7, 8).

Although previous Global Burden of Disease (GBD) studies have provided valuable data, they have predominantly concentrated on all-age or geographically restricted analyses (9–11), leaving a critical knowledge gap regarding the specific global dynamics in the older population. While recent work has tracked historical trends, few studies have moved beyond retrospective analysis to quantitatively model how targeted interventions might alter future outcomes. Our research is designed to directly address this gap.

The novelty and core value of this research lie in its forward-looking approach. Going beyond conventional trend description, this study provides the first comprehensive analysis of the breast cancer burden from 1990 to 2021 exclusively within the global population aged 55 and over, combined with robust forecasting. We employ a Bayesian age-period-cohort (BAPC) model to project the future burden to 2040 and, to address the well-recognized challenge of forecasting uncertainty, we innovatively integrate comprehensive sensitivity and multi-scenario analyses. This methodological advancement allows us to assess the potential impact of interventions, providing concrete scientific evidence for policy-making. In the context of the COVID-19 pandemic and its lasting repercussions on global cancer control systems (12), such forward-looking analysis is particularly timely.

This study aims to systematically analyze the breast cancer burden (incidence, mortality, DALYs) and its trends in individuals aged 55 and over from 1990 to 2021. We investigate attributable risk factors and forecast the future burden to 2040 through our modeling approach. The ultimate goal is to provide high-quality, actionable evidence to inform targeted public health policies and clinical guidelines to mitigate the growing challenge of breast cancer in the world’s aging population.

## 2 Methods

### 2.1 Data source and disease definition

This study utilized publicly available data from the Global Burden of Disease (GBD) 2021 study, managed by the Institute for Health Metrics and Evaluation (IHME) and accessed via the Global Health Data Exchange (GHDx) query tool (13). We extracted annual data on breast cancer incidence, prevalence, mortality, and disability-adjusted life years (DALYs), along with corresponding age-standardized rates (ASRs), for individuals aged 55 years and older from 1990 to 2021. The age cutoff of 55 was chosen based on epidemiological evidence identifying this as a critical turning point where breast cancer incidence and mortality rates begin to climb sharply (14). This threshold effectively covers the primary postmenopausal breast cancer population and aligns with the macro-context of global population aging, making a focused study on this group of significant public health importance.

Countries were categorized into five Socio-Demographic Index (SDI) quintiles (low, low-middle, middle, high-middle, and high) to analyze the relationship between breast cancer burden and socioeconomic development (13). The SDI is a composite measure based on fertility rates, education levels, and per capita income, ranging from 0 to 1 (13).

### 2.2 Attributable burden analysis

This study investigates the changes in the burden of breast cancer among individuals aged 55 and above from 1990 to 2021, based on indicators such as mortality and DALYs. The percentage changes in breast cancer deaths in 2021 relative to 1990 were calculated for different risk factors, both in terms of percentage and number. Additionally, risk factors were ranked according to their impact on mortality, and trends in these factors were analyzed. The study focuses on key risk factor data collected from the GBD 2021, including high body mass index (BMI), alcohol consumption, elevated fasting blood glucose, and smoking.

The contribution of modifiable risk factors to the breast cancer burden was quantified using Population Attributable Fractions (PAFs) from the GBD 2021 study. The GBD framework estimates PAFs by integrating data on risk factor exposure, associated relative risks (RRs), and the theoretical minimum-risk exposure level (TMREL) (13). The conceptual formula for the PAF attributed to a single risk factor is:


$$\mathrm{PAF}=\frac{P_{e}(\mathrm{RR}-1)}{P_{e}(\mathrm{RR}-1)+1}$$

where *P_e_* is the prevalence of exposure in the population and RR is the relative risk for a given outcome due to that exposure. This analysis involved visualizing PAFs across GBD regions and age groups, supplemented by a global-level summary for males, females, and both sexes combined.

### 2.3 Statistical analysis

Using data on the global burden of breast cancer among individuals aged 55 and above from the GBD 2021, including prevalence, incidence, deaths, and DALYs, this study grouped the data by 5-year age intervals to analyze the changes in burden across different age groups and genders. Age-standardized rates (ASRs) for prevalence, incidence, deaths, and DALYs, as well as estimated annual percentage changes (EAPCs), were employed to assess the temporal trends in breast cancer (9).

The ASR was calculated per 100,000 individuals using the following formula:


$$\mathrm{ASR}=\frac{\sum_{i=1}^{A}\;\;a_{i}w_{i}}{\sum_{i=1}^{A}\;\;w_{i}}\times 100,000$$

where *a_i_* is the age-specific rate in the *i*th age group, *w_i_* is the number of persons in the corresponding *i*th age group of the standard population, and *A* is the number of age groups.

To assess temporal trends, we calculated age-standardized rates (ASRs) and Estimated Annual Percentage Changes (EAPCs) from 1990 to 2021. The EAPC was derived by fitting a linear regression model to the natural logarithm of the ASR against the calendar year (*ln(ASR) = α + βx+ϵ*), with the EAPC calculated as *100 × (e^β^ − 1)*. A trend was considered increasing or decreasing if the EAPC and its 95% confidence interval (CI) were both positive or negative, respectively (9). The trend was considered to be increasing if the EAPC and its 95% CI were both positive, and decreasing if they were both negative (9). If the 95% CI included 0, the trend was considered stable. Additionally, future trends up to 2040 were projected using a Bayesian age-period-cohort (BAPC) model, recognized for its high accuracy, via the R package BAPC (15–17).

### 2.4 Sensitivity analysis

To address model uncertainty and evaluate the robustness of the BAPC projections, a series of sensitivity analyses were conducted. The projections from the primary model, which utilized second-order random walks (RW2) for age, period, and cohort effects, were compared against the outputs of three alternatively specified models. These included: (1) a model using first-order random walks (RW1) to assume more linear trends; (2) a model with a more informative (stricter) prior distribution for the smoothing parameters; and (3) an age-period model where the cohort effect was excluded to assess its overall impact.

### 2.5 Intervention scenario analysis

To explore how future advancements might plausibly alter the projected burden of breast cancer, an evidence-based scenario analysis was performed. This analysis simulated the impact of different interventions by applying modification factors to the baseline age-standardized rate (ASR) projections and their 95% credible intervals from 2025 onwards. The simulation framework varied depending on the nature of the intervention. For sudden-impact events like a treatment breakthrough, a one-time multiplicative factor was applied to the ASR for all subsequent years. For interventions with a gradual rollout, such as enhanced screening, the reduction was modeled linearly, increasing from zero at the start year to the full effect by the end of the projection period. For sustained behavioral changes, such as a lifestyle intervention, a compounding annual risk reduction was modeled by applying the reduction factor cumulatively each year. The scenarios were: an Optimistic Treatment Scenario, modeling an 18% cumulative mortality reduction based on recent immunotherapy trial outcomes (18); an Enhanced Screening Scenario, simulating a 22% cumulative mortality reduction informed by studies on AI-assisted mammography (19); a Lifestyle Intervention Scenario, applying a 1% compounding annual risk reduction based on public health recommendations (20); and a Pessimistic Scenario, modeling an 8% increase in mortality to account for potential healthcare disruptions, as suggested by modeling studies on the impact of the COVID-19 pandemic (21). The impact of these scenarios was further quantified by calculating the absolute number of avoided or additional cases and deaths in 2040 relative to the baseline projection.

## 3 Result

### 3.1 Global-level analysis

Globally, the absolute number of new breast cancer cases among individuals aged 55 and older increased substantially, rising from 516,906 (95% UI: 486011.4–535689.9) in 1990 to 1294277.7 (95% UI: 1183277.6–1383581.9) in 2021 (Table 1). The age-standardized incidence rate (ASIR) also trended upward, from 77.0 (95% UI: 72.4–79.8) per 100,000 in 1990 to 87.1 (95% UI, 79.6–93.1) per 100,000 in 2021, with a corresponding estimated annual percentage change (EAPC) of 0.36 (95% CI, 0.31–0.41) (Supplementary Figure S1; Table 1). Similarly, the age-standardized prevalence rate (ASPR) reached 904.8 (95% UI, 847.6–956.0) per 100,000 in 2021, showing a comparable increase with an EAPC of 0.32 (95% CI, 0.29–0.34) (Supplementary Figure S1; Supplementary Table S1).

**Table 1:** Incidence of breast cancer between 1990 and 2021 at the global and regional level.

Location	Number 1990	ASR 1990 (95% CI)	Number 2021	ASR 2021 (95% CI)	EAPC (95%CI)
Global	516906 (486011.4–535689.9)	77 (72.4–79.8)	1294277.7 (1183277.6–1383581.9)	87.1 (79.6–93.1)	0.36 (0.31 to 0.41)
High SDI	305050.7 (284950.2–315170.2)	163.6 (152.8–169)	537674.1 (477720.3–567855.6)	155.8 (138.5–164.6)	−0.19 (−0.28 to −0.11)
High-middle SDI	123478.4 (116843.8–128695.6)	71.6 (67.7–74.6)	325962.3 (293221.3–357859.9)	94 (84.6–103.2)	0.82 (0.73 to 0.9)
Middle SDI	55229.9 (50803.2–60423.3)	31.8 (29.3–34.8)	284424.8 (255666.6–317,342)	60.5 (54.4–67.5)	2.03 (1.99 to 2.08)
Low-middle SDI	22384.8 (19714.8–25233.1)	22.2 (19.6–25)	110319.2 (98606.5–120600.7)	45.8 (40.9–50)	2.38 (2.35 to 2.42)
Low SDI	10121.6 (8686.8–11602.2)	27.1 (23.3–31.1)	34459.9 (30431.8–38406.4)	42 (37.1–46.8)	1.39 (1.27 to 1.52)
Andean Latin America	1112.6 (934.9–1322.8)	33.2 (27.9–39.4)	5803.3 (4461.8–7529.6)	58.6 (45–76)	1.64 (1.51 to 1.77)
Australasia	6279.9 (5834.4–6695.5)	159.4 (148.1–170)	14095.6 (12197.9–15771.6)	159.6 (138.1–178.5)	−0.07 (−0.23 to 0.08)
Caribbean	3301.6 (3077.8–3546.6)	76.6 (71.4–82.3)	9634.6 (8309.7–10979.8)	104.1 (89.8–118.6)	1.09 (0.97 to 1.2)
Central Asia	5037.5 (4692.5–5354.2)	63 (58.7–66.9)	8442.6 (7545.3–9332.1)	58 (51.9–64.1)	0.14 (0.05 to 0.24)
Central Europe	22,093 (21067.2–23051.9)	83.3 (79.4–86.9)	48051.8 (43747.7–51,898)	129.8 (118.1–140.2)	1.35 (1.19 to 1.51)
Central Latin America	6588.4 (6344.8–6810.8)	48.6 (46.8–50.2)	40168.8 (35308.8–45043.2)	93.9 (82.6–105.3)	1.95 (1.87 to 2.04)
Central Sub-Saharan Africa	1,248 (877.7–1669.1)	33.2 (23.3–44.4)	4548.5 (3337.4–6009.8)	50.4 (37–66.6)	1.35 (1.21 to 1.48)
East Asia	39,349 (32657.5–47154.2)	26.4 (21.9–31.7)	230746.7 (184278.5–285223.4)	58.8 (47–72.7)	2.76 (2.66 to 2.85)
Eastern Europe	38156.4 (36743.6–39574.8)	78 (75.2–80.9)	73635.3 (66721.7–81371.1)	118.6 (107.5–131.1)	1.08 (0.96 to 1.19)
Eastern Sub-Saharan Africa	4522.8 (3845.5–5400.2)	37.2 (31.6–44.4)	14871.1 (12853.1–17401.4)	55 (47.5–64.4)	1.21 (1.11 to 1.31)
High-income Asia Pacific	14,293 (13189.4–15213.8)	40.9 (37.7–43.5)	65,901 (54916.6–72762.6)	93.5 (77.9–103.2)	2.94 (2.75 to 3.13)
High-income North America	151967.2 (141053–157789.4)	262.3 (243.5–272.4)	227930.6 (205006.5–240480.7)	202.5 (182.2–213.7)	−1.12 (−1.23 to −1.01)
North Africa and Middle East	6607.2 (6004–7298.7)	23.4 (21.2–25.8)	58521.6 (52162.6–65378.1)	76.8 (68.4–85.8)	4.47 (4.19 to 4.76)
Oceania	200.4 (159.6–248.5)	41.7 (33.2–51.7)	602.9 (496.9–724.7)	48.8 (40.3–58.7)	0.38 (0.3 to 0.46)
South Asia	18110.1 (15679–20,538)	19.1 (16.5–21.6)	95995.6 (82449.9–112310.7)	38.7 (33.2–45.2)	2.19 (2.06 to 2.33)
Southeast Asia	13303.3 (11108–16131.3)	31.4 (26.2–38.1)	66753.6 (55597.1–80793.1)	58.3 (48.5–70.5)	2 (1.91 to 2.09)
Southern Latin America	7823.1 (7295.7–8292.2)	98.8 (92.1–104.7)	15422.3 (13816.6–16814.6)	104.8 (93.9–114.3)	0.23 (0.03 to 0.44)
Southern Sub-Saharan Africa	2014.9 (1603–2449.9)	45.5 (36.2–55.4)	8236.2 (7514–8992.2)	84.6 (77.2–92.4)	2.27 (2.06 to 2.48)
Tropical Latin America	8075.1 (7619.1–8,452)	53.3 (50.3–55.8)	34208.9 (31260.3–36670.1)	77.2 (70.6–82.8)	0.95 (0.84 to 1.06)
Western Europe	161327.8 (150906.3–167411.8)	166.1 (155.4–172.4)	249235.1 (217604.4–267193.8)	167.1 (145.9–179.2)	0.08 (−0.07 to 0.24)
Western Sub-Saharan Africa	5494.7 (4518.7–6463.6)	38.1 (31.3–44.8)	21471.8 (17085.3–27085.2)	66.8 (53.2–84.3)	1.93 (1.74 to 2.12)

SDI, socio-demographic index; EAPC, estimated annual percentage change; CI, confidence interval; UI, uncertainty interval.

In contrast to incidence trends, mortality and DALY rates showed significant declines. While the absolute number of deaths increased from 238913.4 (95% UI: 223054.4–249779.1) in 1990 to 473476.5 (95% UI: 428994.0–508325.5) in 2021, the age-standardized mortality rate (ASMR) decreased from 35.6 (95% UI: 33.2–37.2) to 31.9 (95% UI: 28.9–34.2) per 100,000 over the same period, with an EAPC of −0.46 (95% CI: −0.50 to −0.41) (Supplementary Figure S1; Supplementary Table S2). Likewise, disability-adjusted life years (DALYs) rose to 11058137.8 (95% UI: 10223544.4–11869597.9) in 2021, yet the age-standardized DALY rate (ASDR) fell from 841.6 (95% UI: 794.8–884.6) to 744.2 (95% UI: 688.0–798.8) per 100,000, with an EAPC of −0.48 (95% CI: −0.52 to −0.45) (Supplementary Figure S1; Supplementary Table S3). These findings highlight a divergence between rising case numbers and decreasing standardized rates of mortality and disability.

### 3.2 Regional-level analysis

Analysis by Socio-Demographic Index (SDI) reveals significant disparities in breast cancer burden for the 55-and-older population. In 2021, high-SDI regions reported the highest number of incident cases at 537674.1 (95% UI: 477720.3–567855.6) and exhibited the highest ASIR and ASPR, at 155.8 (95% UI: 138.5–164.6) and 1823.0 (95% UI: 1698.6–1929.5) per 100,000, respectively (Supplementary Figure S1; Table 1; Supplementary Table S1). Conversely, low-SDI regions had the lowest ASIR (42.0 per 100,000; 95% UI: 37.1–46.8) (Supplementary Figure S1; Table 1). From 1990 to 2021, all SDI quintiles except for the high-SDI group experienced an increase in ASIR, with the most pronounced rise occurring in low-middle SDI regions (EAPC: 2.38; 95% CI: 2.35–2.42) (Supplementary Figure S1; Table 1). Notably, a significant upward trend in ASIR and ASPR for male breast cancer was observed globally and across all SDI regions (Supplementary Figure S1).

In 2021, high-SDI regions also recorded the greatest number of deaths (147971.6; 95% UI: 128248.7–158356.4) and the highest ASMR and ASDR, at 42.9 (95% UI: 37.2–45.9) and 905.4 (95% UI: 813.5–972.2) per 100,000, respectively (Supplementary Figure S1; Supplementary Tables S2, S3). Despite this, these regions also demonstrated the most substantial reduction in standardized rates since 1990, with an EAPC for ASMR of −1.23 (95% CI: −1.28 to −1.17) (Supplementary Figure S1; Supplementary Table S2). In stark contrast, low-middle SDI regions showed the largest increase in ASMR (EAPC: 1.57; 95% CI: 1.53–1.61) and ASDR (EAPC: 1.48; 95% CI: 1.44–1.53) (Supplementary Figure S1; Supplementary Tables S2, S3). Low-SDI regions reported the lowest absolute number of deaths but maintained relatively high standardized mortality and DALY rates.

Among the 21 GBD regions, the burden of breast cancer in the 55 + population varied widely. The number of incident cases increased in most regions from 1990 to 2021, with the North Africa and Middle East region showing the most dramatic rise (EAPC: 4.47; 95% CI: 4.19–4.76) (Table 1). Conversely, the largest decline in incidence was observed in High-Income North America (EAPC: -1.12; 95% CI: −1.23 to −1.01) (Table 1). Despite this decrease, High-Income North America still held the highest ASIR in 2021, while Western Europe recorded the highest absolute number of incident cases (249235.1; 95% UI: 217604.4–267193.8) and prevalent cases (2995238.8; 95% UI: 2788352.5–3170207.2) (Table 1; Supplementary Table S1).

Mortality patterns also showed significant regional heterogeneity. Notably, Southern Sub-Saharan Africa had the second-highest ASMR (56.9 per 100,000; 95% UI: 52.0–62.2) and the highest ASDR (1335.7 per 100,000; 95% UI: 1217.8–1460.0), with these metrics also showing the largest increases across all regions (ASMR EAPC: 1.68; ASDR EAPC: 1.89) (Supplementary Tables S2, S3). In stark contrast, East Asia reported the lowest ASMR (16.6 per 100,000; 95% UI: 13.3–20.4) and ASDR (433.2 per 100,000; 95% UI: 346.1–534.7) among all regions (Supplementary Tables S2, S3).

### 3.3 Country-level analysis

**Figure 1:**
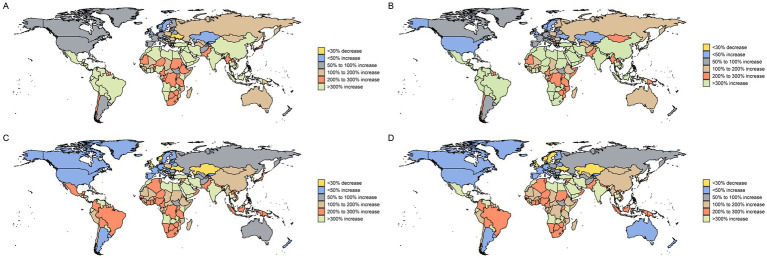
Change in breast cancer cases for both sexes in 204 countries and territories in people over 55 years of age, 1990 to 2021. This figure presents global maps depicting the percentage change in the number of breast cancer cases for individuals over 55 years of age. **(A)** Change in prevalence cases. **(B)** Change in incidence cases. **(C)** Change in deaths cases. **(D)** Change in DALYs. Different color differences indicate the magnitude of change.

At the national level, most countries exhibited an increasing trend in ASIR and ASPR for the 55 + population between 1990 and 2021. In 2021, China reported the highest number of new cases (222613.9; 95% UI: 175869.3–276986.2) and showed a significant increase in ASIR (EAPC: 2.74; 95% CI: 2.65–2.84) (Figure 1; Supplementary Tables S4, S5). Conversely, the United States had the highest number of prevalent cases (2399818.8; 95% UI: 2227392.1–2554692.3) but demonstrated a declining trend in ASIR (EAPC: −1.11; 95% CI: −1.23 to −1.0) (Figure 1; Supplementary Tables S4, S5). The most rapid increase in incidence was observed in Turkey, where the ASIR rose with a globally leading EAPC of 6.55 (95% CI: 5.74–7.36) (Supplementary Table S4). Despite these trends, the highest ASIR in 2021 was recorded in the United States (208.1 per 100,000; 95% UI: 187.2–220.0), while Monaco had the highest ASPR (2403.2 per 100,000; 95% UI: 2175.0–2598.6) (Figure 1; Supplementary Tables S4, S5).

In terms of mortality, many countries showed declining trends in ASMR and ASDR, reflecting improvements in cancer care. Nations such as Denmark, Norway, and Greenland demonstrated substantial reductions; for example, Denmark’s ASMR decreased with an EAPC of −1.90 (95% CI: −1.98 to −1.81) (Figure 1; Supplementary Tables S6, S7). China recorded the highest absolute number of deaths (61934.7; 95% UI: 49020.8–76670.1) and DALYs (1616381.5; 95% UI: 1275221.6–2014511.7) in 2021, although its standardized rates remained relatively low (ASMR: 16.3 per 100,000) (Figure 1; Supplementary Tables S6, S7). The highest ASMR and ASDR globally were observed in Monaco, at 97.7 (95% UI: 72.9–127.6) and 1952.3 (95% UI: 1475.5–2523.1) per 100,000, respectively (Figure 1; Supplementary Tables S6, S7).

### 3.4 Age and gender patterns

**Figure 2:**
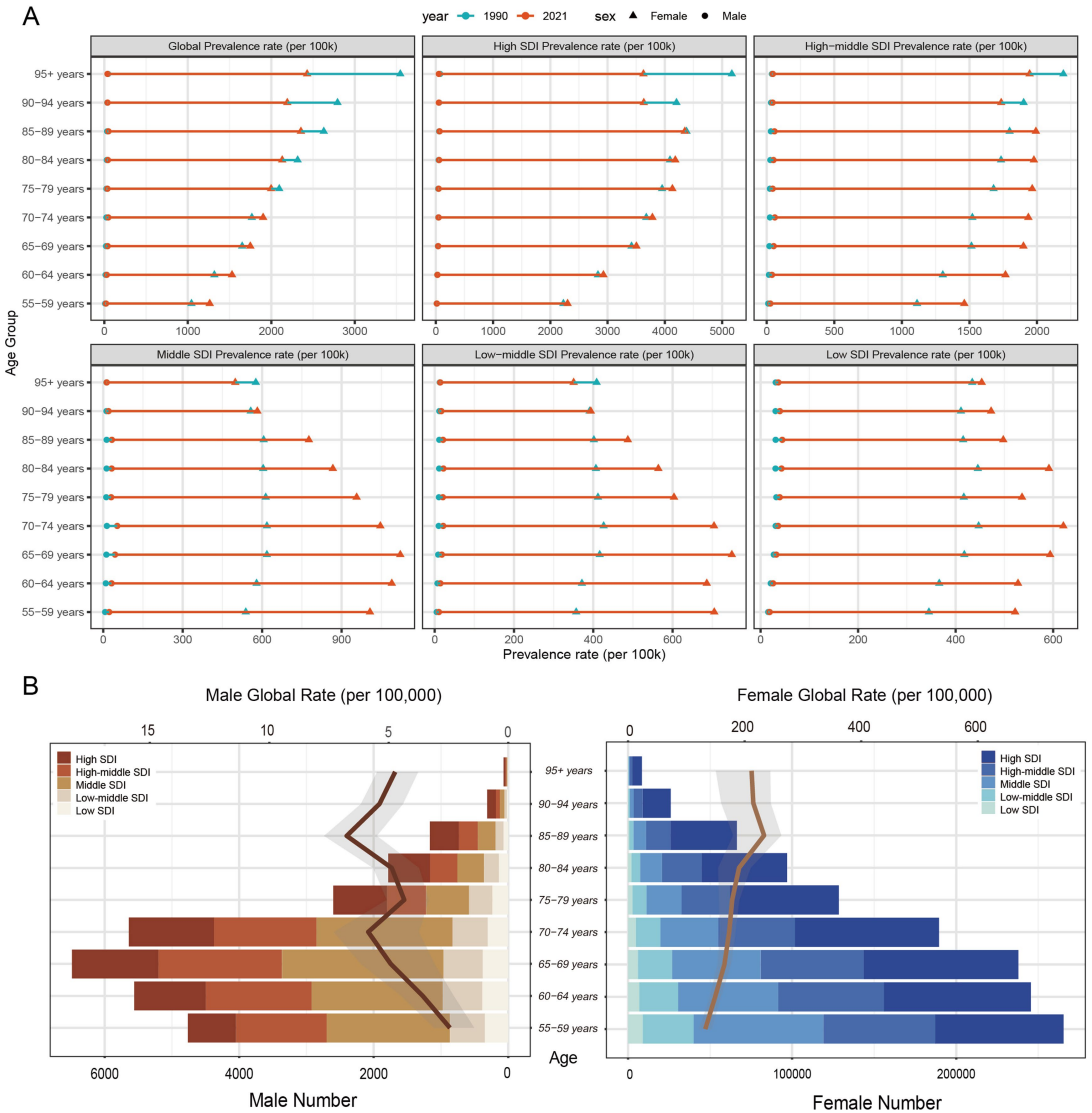
ASPRs, age-specific numbers, and ASIRs of breast cancer by age group and SDI regions. **(A)** Age-standardized prevalence rates (ASPR) of breast cancer for individuals aged 55–95 + years, by sex and SDI regions, in 1990 and 2021. **(B)** The age-specific numbers and age-standardized incidence rates (ASIR) of breast cancer by SDI region in 2021 for males and females.

In 2021, the global prevalence of breast cancer among the 55 + population increased with age, peaking in the 80 and older age group. A notable divergence in prevalence trends from 1990 to 2021 was observed across different Socio-Demographic Index (SDI) regions. In low, low-middle, and middle-SDI regions, there was a marked increase in prevalence among women aged 55–85 (Figure 2A). In contrast, high and high-middle SDI regions showed no significant growth in prevalence for this demographic, and the rate among women aged 90 and above declined significantly (Figure 2A).

Breast cancer incidence rates were substantially higher in women than in men. The majority of global cases were concentrated in high and high-middle SDI regions, primarily within the 55–75 age group (Figure 2B). Peak prevalence occurred at different ages for each sex: the highest rate for women was in the 55–59 age group, whereas for men, it was in the 65–69 age group (Figure 2B). Male breast cancer cases were more prevalent in high-middle and middle-SDI regions, where their incidence rates showed a slight upward trend (Figure 2B). Overall, men were generally diagnosed at an older age than women.

### 3.5 Risk factors in breast cancer

**Figure 3:**
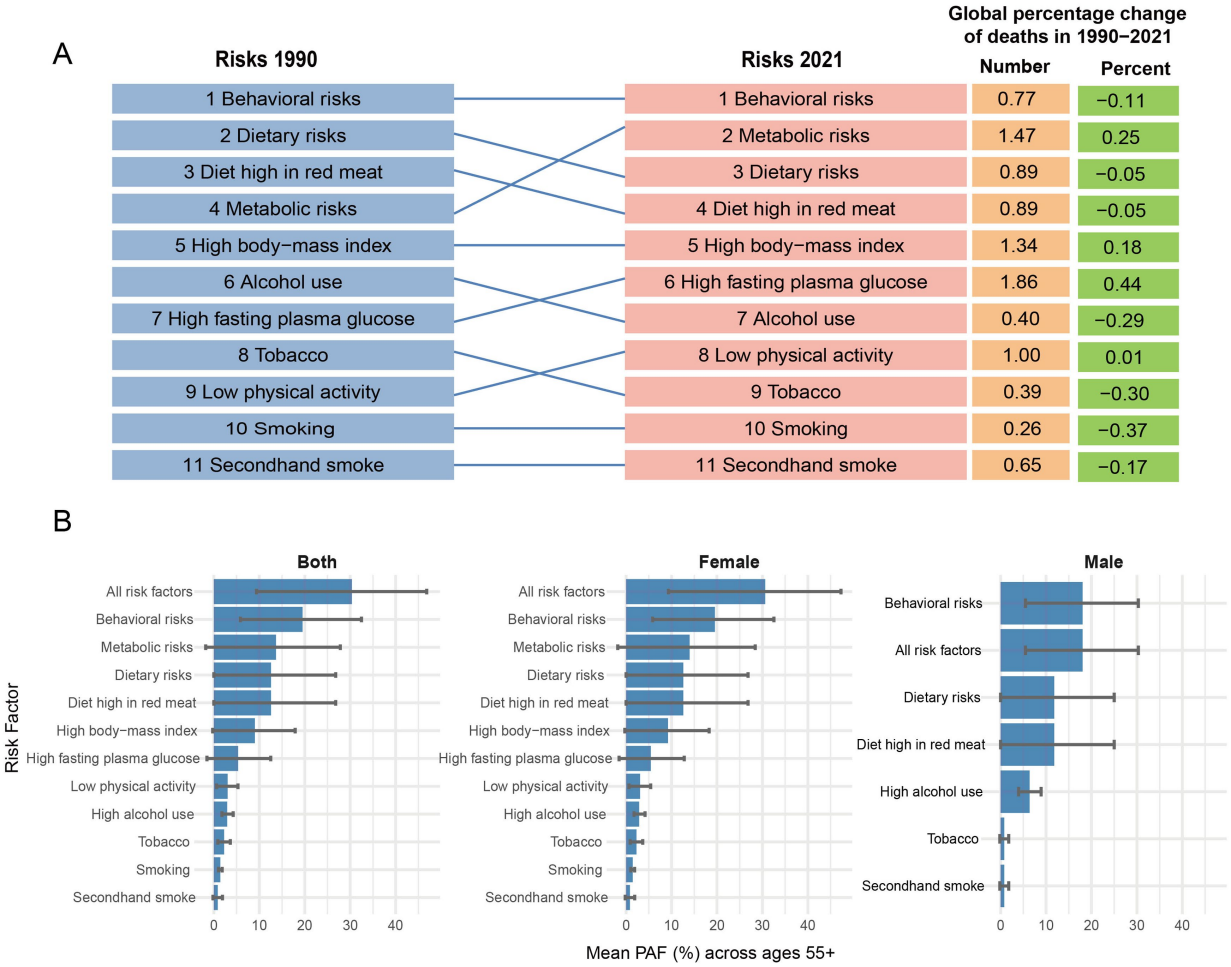
Analysis of risk factors among people aged 55 and above, 1990–2021. **(A)** Global percentage change of risk factor mortality and ranking of risk factors related to mortality, 1990–2021. **(B)** Population attributable fraction for breast cancer DALYs in population aged 55 + and contribution of major risk factors by sex in 2021. **(A)** Illustrates the global percentage change and ranking of selected risk factors associated with breast cancer mortality from 1990 to 2021, showing the changes in their position over time. **(B)** Presents the population attributable fraction (PAF) for breast cancer disability-adjusted life-years (DALYs) in individuals aged 55 and older in 2021, broken down by major risk factors and sex (Both, Female, Male).

Globally, behavioral, metabolic, and dietary risks were the primary contributors to the breast cancer burden in the population aged 55 and above (Figure 3A). The influence of metabolic risks on mortality increased significantly between 1990 and 2021; the absolute number of deaths attributable to these factors rose by 147%, and their proportional contribution to total deaths grew by 25% (Figure 3A). Specifically, high fasting plasma glucose and low physical activity emerged as increasingly significant drivers of mortality (Figure 3A). Deaths attributable to high fasting plasma glucose increased by 186%, while those linked to low physical activity rose by 100% (Figure 3A). As a percentage of total deaths, the contribution from high fasting plasma glucose grew by 44% (Figure 3A). Furthermore, the contribution of dietary risk factors like high red meat intake and alcohol consumption to DALYs and Deaths is particularly prominent in high-SDI regions; for instance, among individuals aged 5–59 in high-SDI areas, high red meat intake contributes 13.6% to Deaths and DALYs, with alcohol consumption reaching 7.4% (Supplementary Figure S3).

The contribution of major risk factors to breast cancer DALYs in the population aged 55 and older at the global level in 2021 highlights significant differences between sexes. For females, the combined PAF from all risk factors was substantial, peaking at 31.2% (95% UI: 9.3–48.3%) in the 75–79 age group (Figure 3B; Supplementary Table S12). The primary contributors were behavioral and metabolic risks, with metabolic risks alone accounting for up to 14.8% (95% UI: −2.0 to 30.0%) of the burden in the 70–74 age group (Figure 3B; Supplementary Table S12). Among specific factors, high body-mass index, dietary risks (particularly high red meat intake), and high fasting plasma glucose were the most prominent drivers of the DALY burden in females (Figure 3B).

In stark contrast, a distinct risk profile was observed for males. The overall attributable burden was lower, with the PAF for all risk factors peaking at 19.9% (95% UI: 7.8–32.3%) in the 55–59 age group (Figure 3B; Supplementary Table S12). Behavioral risks were the leading category, dominated overwhelmingly by high alcohol use, which was the single most significant individual risk factor for males, contributing up to 8.2% (95% UI: 5.6–11.0%) of DALYs in the 55–59 age group (Figure 3B; Supplementary Table S12). While dietary risks also contributed, metabolic factors such as high BMI, which were critical in females, had a negligible impact on the male breast cancer burden (Figure 3B). This highlights a clear divergence in the etiological drivers of breast cancer between sexes in the older population.

### 3.6 Future projections of the global breast cancer burden (2021–2040)

**Figure 4:**
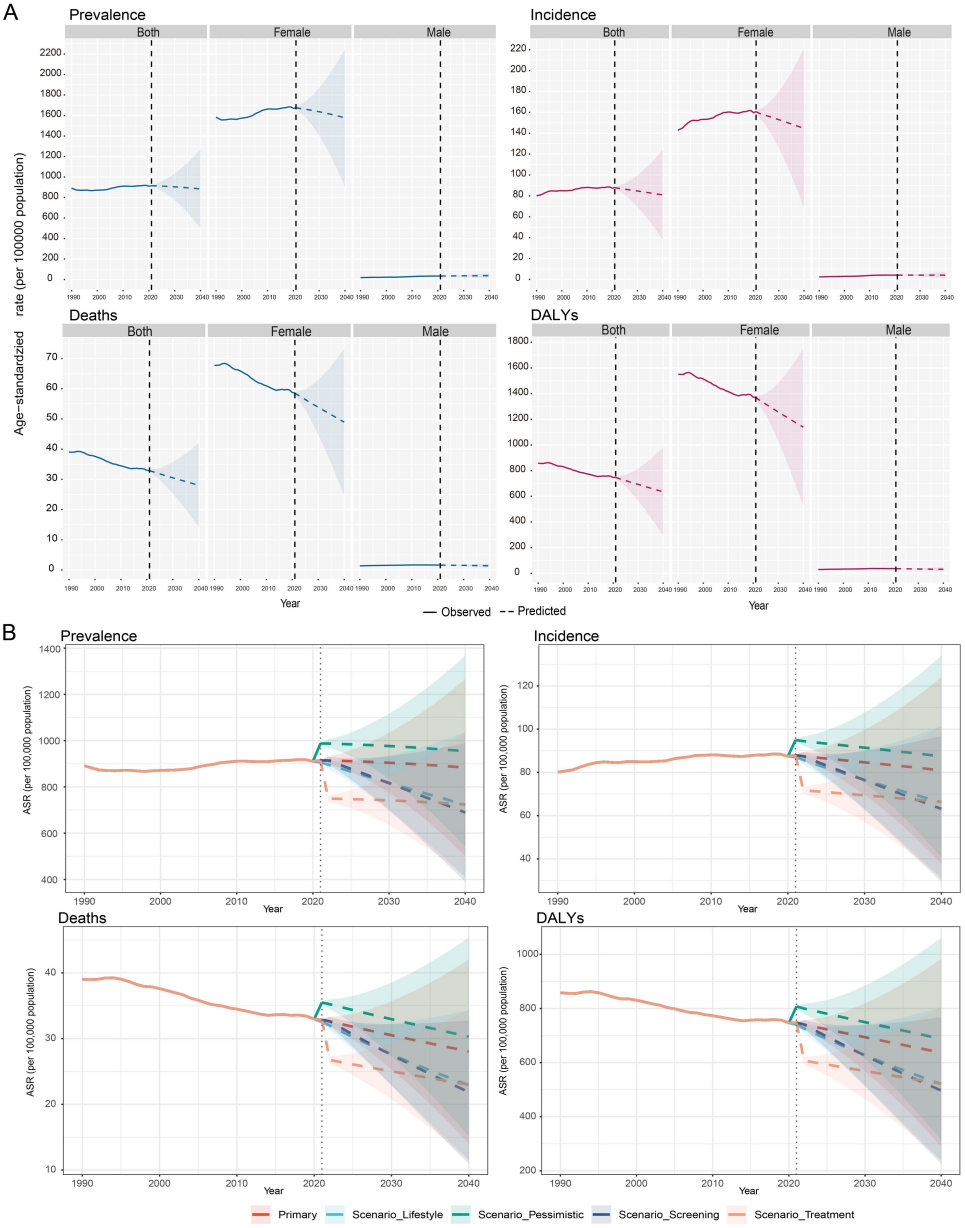
Future forecasts of global burden of breast cancer. **(A)** This panel offers future projections of the age-standardized rates (ASPR, ASIR, ASMR, ASDR, per 100,000 population) for breast cancer at the global level, covering both sexes, females, and males. These forecasts are based on a primary scenario. **(B)** This panel illustrates breast cancer forecasts under various scenarios, including primary, lifestyle, pessimistic, screening, and treatment scenarios.

Based on the Bayesian Age-Period-Cohort (BAPC) model, the global ASIR, ASPR, ASMR and ASDR of breast cancer in the 55 + population are projected to show a steady decline by 2040 (Figure 4A). The baseline projection indicates that the ASIR will decrease from approximately 88 per 100,000 in 2021 to 883.7 per 100,000 in 2040, while the ASPR is projected to drop from 915 to 884 per 100,000 (Supplementary Tables S8, S9). Meanwhile, ASDR decreased from 747.7 in 2021 to 634.8 in 2040; ASMR dropped from 32.8 in 2021 to 28 in 2040 (Supplementary Tables S10, S11). However, a sensitivity analysis revealed that these projections are subject to model specification uncertainty. By 2040, alternative model specifications yielded a range of possible ASR outcomes, with estimates varying from 835.9 per 100,000 population in the age-period model to 971.0 per 100,000 in the RW1 model (Supplementary Figure S4; Supplementary Table S13). Furthermore, this model-based uncertainty was found to widen over the projection period, with the range between the highest and lowest ASPR projections increasing from 45.2 in 2022 to 135.1 by 2040 (Supplementary Table S14).

To explore the potential impact of future interventions, a scenario analysis was conducted, revealing a wide spectrum of possible outcomes. By 2040, an enhanced screening scenario could potentially reduce the ASPR to as low as 689.3 per 100,000, while a pessimistic scenario could see the ASPR rise to 954.4 per 100,000, compared to the baseline projection of 883.7 (Figure 4B; Supplementary Table S13). Translating these rates into a tangible public health metric, an optimistic treatment scenario was projected to avert approximately 3.54 million breast cancer cases globally in 2040 alone, corresponding to an 18.0% reduction from the baseline projection (Table 2). The vast majority of these averted cases would be among females (approximately 3.47 million), with about 74,000 cases averted in males (Table 2). These findings underscore that while the baseline trend is projected to be stable or slightly decreasing, targeted interventions could substantially mitigate the future burden of breast cancer.

**Table 2:** The number of avoidable cases that could be prevented in an different scenario in 2040.

Scenario	Measure	Sex	Estimated cases (95% CI)	Baseline estimated cases	Add/avoid cases
Pessimistic scenario	Prevalence	Both	21261283.24 (12072021.76–30450544.72)	19686373.37	1574909.87
		Female	20818551.28 (11955846.69–29681255.88)	19276436.37	1542114.91
		Male	442731.96 (116175.08–769288.84)	409937.00	32794.96
	Incidence	Both	1955919.26 (913154.76–2998683.76)	1811036.35	144882.91
		Female	1908665.88 (903573.61–2913758.15)	1767283.22	141382.66
		Male	47253.38 (9581.14–84925.61)	43753.13	3500.25
	Deaths	Both	662181.59 (333063.99–991299.18)	613131.10	49050.49
		Female	645789.75 (325144.29–966435.21)	597953.47	47836.28
		Male	16391.83 (7919.70–24863.97)	15177.62	1214.21
	DALYs	Both	15391932.97 (7137478.10–23646387.85)	14251789.79	1140143.18
		Female	15023608.36 (6954455.37–23092761.36)	13910748.49	1112859.88
		Male	368324.61 (183022.72–553626.49)	341041.30	27283.30
Optimistic treatment	Prevalence	Both	16142826.17 (9165794.30–23119858.03)	19686373.37	−3543547.21
		Female	15806677.83 (9077587.30–22535768.35)	19276436.37	−3469758.55
		Male	336148.34 (88207.00–584089.68)	409937.00	−73788.66
	Incidence	Both	1485049.81 (693321.21–2276778.41)	1811036.35	−325986.54
		Female	1449172.24 (686046.63–2212297.85)	1767283.22	−318110.98
		Male	35877.57 (7274.57–64480.56)	43753.13	−7875.56
	Deaths	Both	502767.50 (252881.92–752653.08)	613131.10	−110363.60
		Female	490321.85 (246868.82–733774.88)	597953.47	−107631.63
		Male	12445.65 (6013.10–18878.20)	15177.62	−2731.97
	DALYs	Both	11686467.63 (5419196.33–17953738.92)	14251789.79	−2565322.16
		Female	11406813.76 (5280234.63–17533392.88)	13910748.49	−2503934.73
		Male	279653.87 (138961.70–420346.04)	341041.30	−61387.43
Lifestyle intervention	Prevalence	Both	16101621.36 (9142398.47–23060844.24)	19686373.37	−3584752.02
		Female	15766331.04 (9054416.62–22478245.46)	19276436.37	−3510105.33
		Male	335290.32 (87981.85–582598.78)	409937.00	−74646.68
	Incidence	Both	1481259.20 (691551.49–2270966.90)	1811036.35	−329777.16
		Female	1445473.21 (684295.49–2206650.93)	1767283.22	−321810.01
		Male	35785.99 (7256.00–64315.97)	43753.13	−7967.14
	Deaths	Both	501484.18 (252236.44–750731.92)	613131.10	−111646.92
		Female	489070.29 (246238.68–731901.91)	597953.47	−108883.18
		Male	12413.88 (5997.76–18830.01)	15177.62	−2763.74
	DALYs	Both	11656637.74 (5405363.75–17907911.73)	14251789.79	−2595152.05
		Female	11377697.69 (5266756.76–17488638.63)	13910748.49	−2533050.79
		Male	278940.05 (138607.00–419273.10)	341041.30	−62101.26
Enhanced screening	Prevalence	Both	15355371.23 (8718682.39–21992060.08)	19686373.37	−4331002.14
		Female	15035620.37 (8634778.16–21436462.58)	19276436.37	−4240816.00
		Male	319750.86 (83904.22–555597.50)	409937.00	−90186.14
	Incidence	Both	1412608.35 (659500.66–2165716.05)	1811036.35	−398428.00
		Female	1378480.91 (652580.94–2104380.88)	1767283.22	−388802.31
		Male	34127.44 (6919.72–61335.17)	43753.13	−9625.69
	Deaths	Both	478242.26 (240546.22–715938.30)	613131.10	−134888.84
		Female	466403.71 (234826.43–697980.99)	597953.47	−131549.76
		Male	11838.55 (5719.78–17957.31)	15177.62	−3339.08
	DALYs	Both	11116396.04 (5154845.29–17077946.78)	14251789.79	−3135393.75
		Female	10850383.82 (5022662.21–16678105.42)	13910748.49	−3060364.67
		Male	266012.22 (132183.08–399841.36)	341041.30	−75029.09

## 4 Discussion

### 4.1 Global burden of breast cancer

This study presents a comprehensive analysis of the breast cancer burden among the population aged 55 and older, revealing a complex and diverging global landscape between 1990 and 2021. The principal finding is a striking paradox: while the absolute numbers of incident cases, deaths, and DALYs have increased substantially, the global age-standardized mortality (ASMR) and DALY rates have significantly declined. This indicates that while more older individuals are affected by breast cancer, likely due to population growth and aging, the per-capita risk of dying from the disease has decreased, pointing to net progress in cancer control. However, these global trends mask profound disparities, with the burden increasingly shifting toward regions with lower socioeconomic development. Compared to the younger patient population, individuals aged 55 and over bear the vast majority of the absolute case and mortality burden of breast cancer globally and typically face a worse prognosis. This trend, where the absolute burden continues to escalate due to population aging, is consistent with broader patterns observed for many other non-communicable diseases worldwide.

### 4.2 Epidemiological divide: the role of socio-economic development

Our findings highlight a clear epidemiological divide driven by the Socio-Demographic Index (SDI). High-SDI regions, such as High-Income North America and Western Europe, have achieved significant reductions in ASMR, with EAPCs as low as −1.23. This success can be largely attributed to decades of investment in well-established national screening programs leading to early detection, alongside access to advanced treatment modalities, including novel targeted therapies and immunotherapies (22). In stark contrast, low- and middle-SDI regions are facing a dual challenge: a rapid increase in the age-standardized incidence rate (ASIR), with EAPCs exceeding 2.0 in some quintiles, and a concurrent rise in mortality rates. This alarming trend, particularly pronounced in regions like North Africa and the Middle East and countries such as Turkey where incidence has risen dramatically, is likely fueled by a “Westernization” of lifestyles—including changes in diet and increases in obesity—without the concurrent establishment of robust healthcare infrastructure for early diagnosis and effective treatment (23, 24). This widening gap underscores that progress in the fight against breast cancer is not being shared equitably, and regions with the most rapidly growing burden are also the least equipped to manage it.

### 4.3 Divergent risk factor profiles and the burden of male breast cancer

A key finding of this study, illuminated by our detailed Population Attributable Fraction (PAF) analysis, is the profound divergence in risk factor profiles between sexes. For females aged 55 and older, the DALY burden is driven by a combination of metabolic and behavioral risks. Metabolic factors, particularly high body-mass index (BMI) and high fasting plasma glucose, represent a major and growing threat, consistent with the rising global prevalence of obesity and diabetes. This highlights that for older women, breast cancer prevention is inextricably linked to the management of overall metabolic health.

In stark contrast, our analysis reveals that the breast cancer burden in older men is overwhelmingly driven by behavioral risks, with high alcohol use emerging as the single most dominant attributable risk factor. This finding is critical, as male breast cancer is often underdiagnosed and its specific etiology is less understood than its female counterpart (8, 25). While factors like high red meat consumption also contribute, the prominence of alcohol suggests a distinct biological pathway and offers a clear, actionable target for public health intervention specifically for men (26). This underscores the necessity of moving beyond a female-centric view of breast cancer and developing tailored awareness and prevention campaigns for the male population.

### 4.4 Future projections: model uncertainty and the power of intervention

This study’s novelty is significantly enhanced by its forward-looking analysis. While our baseline Bayesian Age-Period-Cohort (BAPC) model projects a stabilization or slight decline in global ASRs by 2040, the true value of this analysis lies in quantifying uncertainty and exploring alternative futures. Our sensitivity analysis demonstrates that the uncertainty inherent in these projections widens over time, underscoring the need for caution in long-term planning and the importance of continuous monitoring.

More importantly, the intervention scenario analysis transforms our model from a simple forecasting tool into a policy simulation engine. The results show that the future burden of breast cancer is not predetermined. An enhanced screening scenario could reduce the ASR in 2040 by over 20% compared to baseline, potentially averting millions of cases. Conversely, the pessimistic scenario—reflecting potential healthcare disruptions—projects an increase in the burden. The COVID-19 pandemic serves as a stark real-world example of such a disruption, where delayed diagnoses and interruptions to treatment have been shown to lead to worse cancer outcomes (12, 27), highlighting the fragility of public health gains. This scenario analysis provides policymakers with tangible evidence that proactive investment in screening and treatment infrastructure is not only effective but essential to securing a more favorable future.

### 4.5 Clinical implications and recommendations for an aging population

The concentration of the breast cancer burden in the population aged 55 and older mandates a shift toward more personalized, age-adapted care. Clinically, this challenges one-size-fits-all screening protocols, suggesting a need for individualized strategies that consider a patient’s comorbidities and functional status, not just chronological age. For those diagnosed, treatment decisions must be guided by comprehensive geriatric assessments (CGA) to balance efficacy with toxicity (28). Our findings also call for sex-specific integrated care: for women, this means coupling oncologic treatment with proactive management of metabolic risks like obesity and high fasting plasma glucose (29), while for the often-overlooked male population, the distinct risk profile dominated by high alcohol use demands higher clinical suspicion and targeted counseling on alcohol reduction (30).

These clinical actions must be supported by targeted, resource-stratified public health strategies. For developing regions facing a rising burden, policy should prioritize downstaging through improved public awareness and access to early diagnosis, in line with the WHO Global Breast Cancer Initiative (31). For high-income nations, the focus must be on optimizing geriatric oncology care and implementing precision prevention that addresses the distinct metabolic and behavioral risk profiles of older women and men (32, 33). Ultimately, mitigating the future burden of breast cancer requires not only the equitable allocation of current resources but also a commitment to more granular, data-driven surveillance to guide prevention and treatment for all (34, 35).

### 4.6 Strengths and limitations

The primary strength of this study is its use of the comprehensive and standardized GBD 2021 dataset to provide a global overview of breast cancer in a high-risk, older population. A key innovation of our work is the integration of advanced statistical modeling that goes beyond simple trend description. The inclusion of a BAPC sensitivity analysis adds a crucial layer of interpretation regarding model uncertainty, while the multi-scenario analysis provides novel, forward-looking insights into the potential impact of public health interventions, directly addressing calls for more policy-relevant research.

However, several limitations must be acknowledged. As an analysis of aggregated data, this study is subject to the ecological fallacy and the potential underestimation of the true burden in regions with limited primary data. Furthermore, our risk attribution (PAF) analysis is based on statistical associations, not causation, and does not capture the complex interplay of genetic and environmental factors. While statistically robust, our projections cannot account for unforeseen events beyond the scope of our defined scenarios. Finally, a critical limitation is the inability of GBD data to be disaggregated by molecular subtype (e.g., HR+/HER2−, triple-negative). This is significant as these subtypes are distinct diseases with unique risk profiles and prognoses, a factor that impacts the depth of our attributable burden analysis.

## 5 Conclusion

In summary, this study highlights the escalating global impact of breast cancer on individuals aged 55 and older, while also revealing substantial disparities between regions characterized by varying degrees of socioeconomic development. The rising incidence rates in low- and middle-SDI regions, combined with the persistently high burden in high-SDI areas, highlight the need for a comprehensive and region-specific approach to breast cancer control. This approach should prioritize improving access to early screening and advanced treatments in underdeveloped regions, implementing customized prevention strategies, and tackling significant modifiable risk factors, including unhealthy diets, sedentary lifestyles, and obesity.

## Data Availability

The original contributions presented in the study are included in the article/Supplementary material, further inquiries can be directed to the corresponding authors.
