# Supplementary material for: Breast cancer burden (1990–2040) in individuals aged 55 and above: a GBD 2021 analysis of global trends, sex-specific risk factors, and intervention impact
Source: Front Public Health. 2025 Jul 31;13:1586497. doi: 10.3389/fpubh.2025.1586497 (PMC12350376; doi:10.3389/fpubh.2025.1586497)
Supplement: Supplementary file 1 [file Supplementary_file_1.docx]

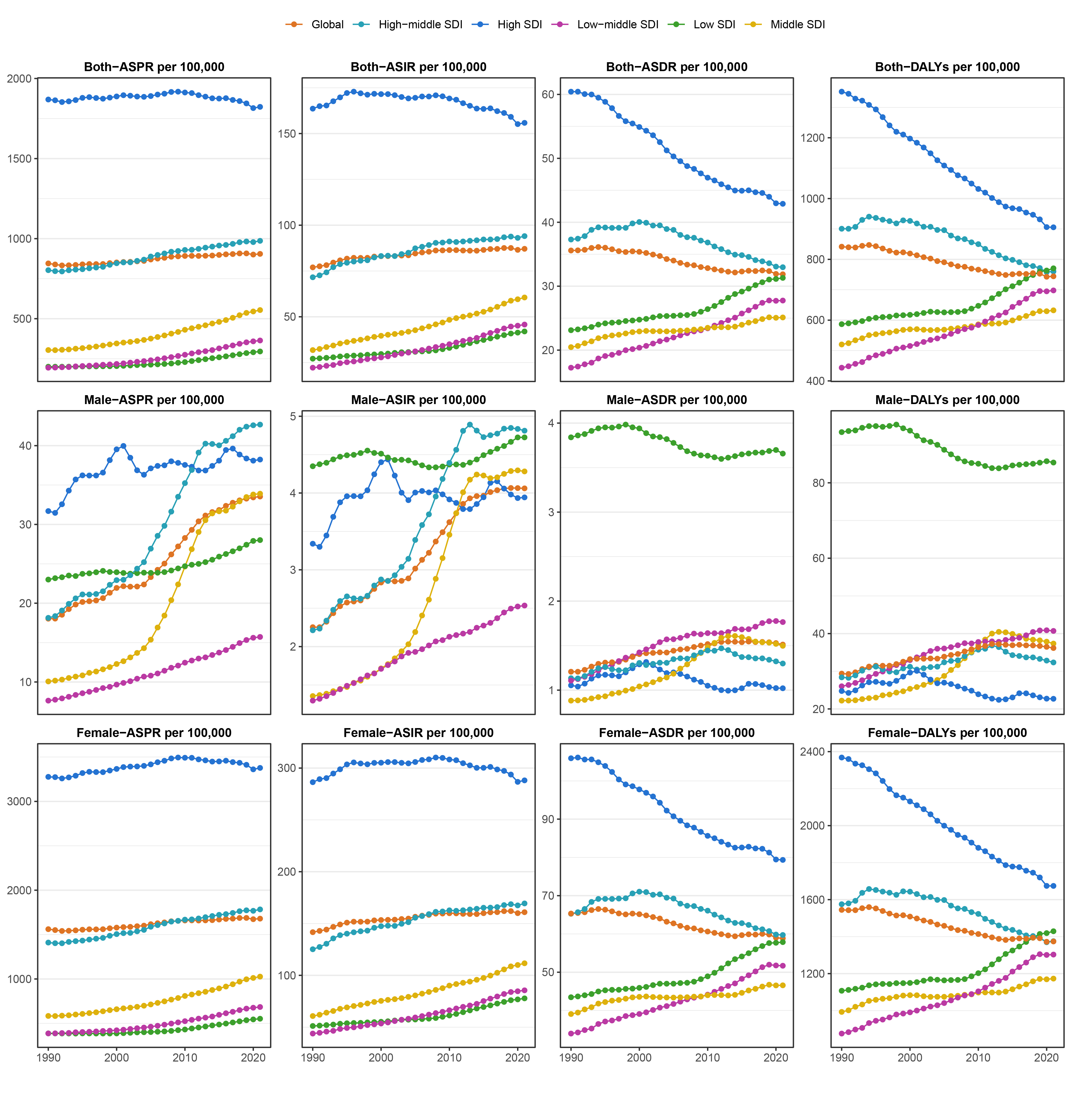
**Fig S1** Trends in breast cancer prevalence, incidence, deaths, and DALYs from 1990 to 2021. This figure illustrates the trends of age-standardized rates (ASPR, ASIR, ASMR, ASDR) for breast cancer across different Socio-Demographic Index (SDI) regions (Global, High, High-middle, Middle, Low-middle, Low) for both sexes, males, and females, from 1990 to 2021.

**
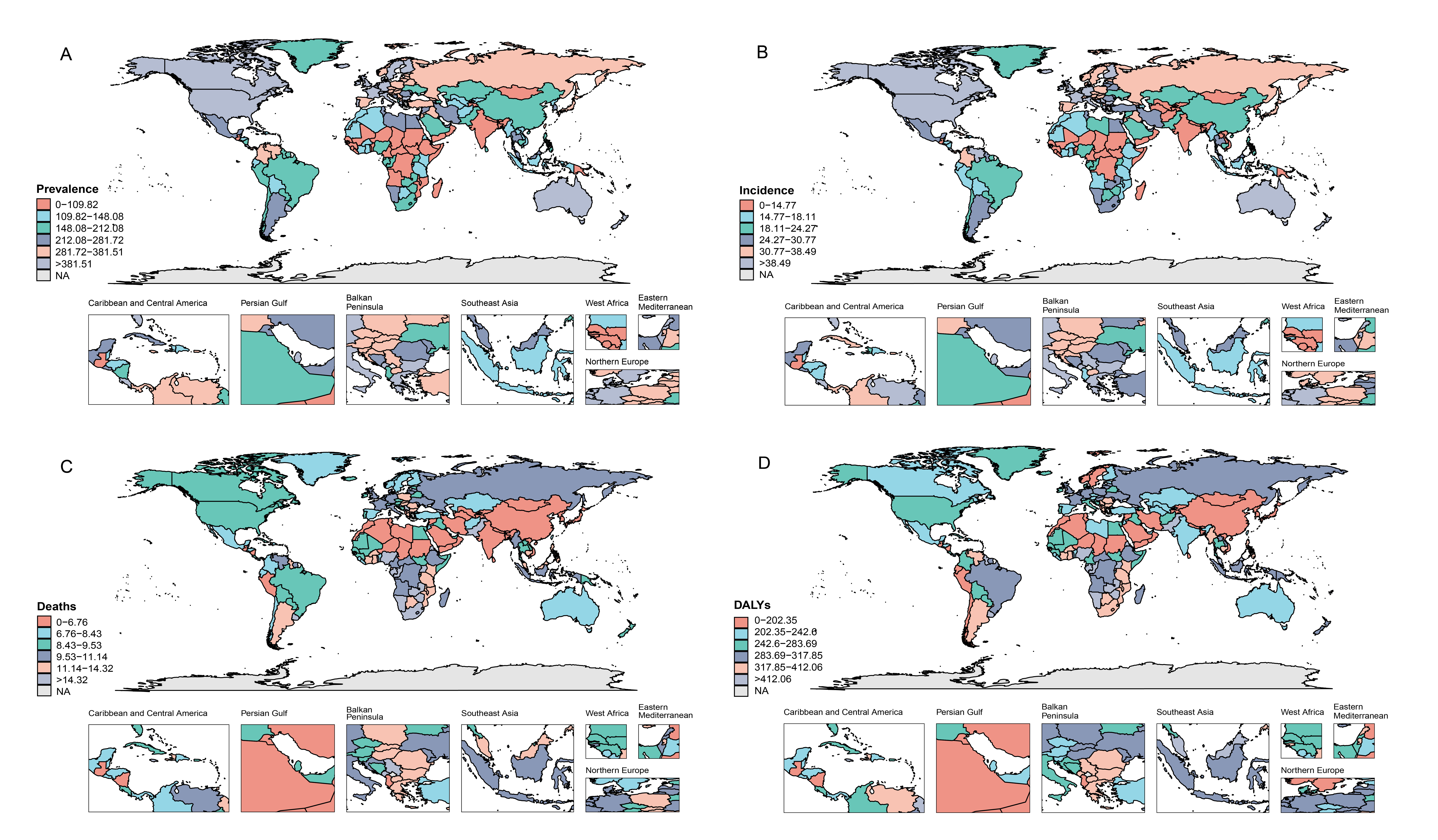
Fig S2** The global disease burden of breast cancer in people over 55 years of age in 204 countries and territories. (A) Age-standardized prevalence rate (ASPR). (B) Age-standardized incidence rate (ASIR). (C) Age-standardized death rate (ASMR). (D) Age-standardized disability-adjusted life-years rate (ASDR). Different color differences indicate the magnitude of rate.

**
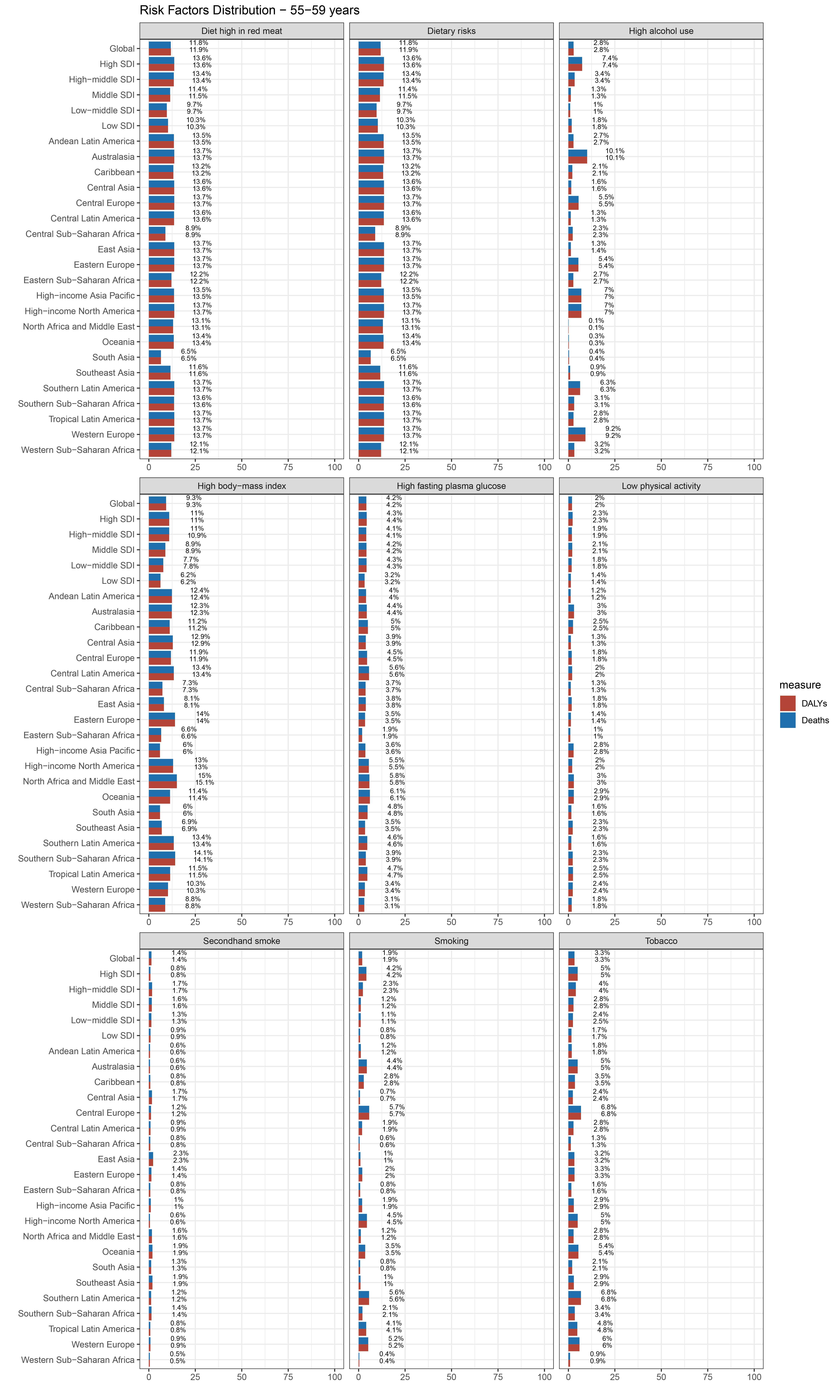
**

**Fig S3** The breast cancer DALYs and deaths attributable to risk factors in people aged 55-59 years, 1990 and 2021. This figure illustrates the percentage of breast cancer DALYs and deaths attributable to specific risk factors for individuals aged 55-59 years, globally and by region, comparing 1990 and 2021.


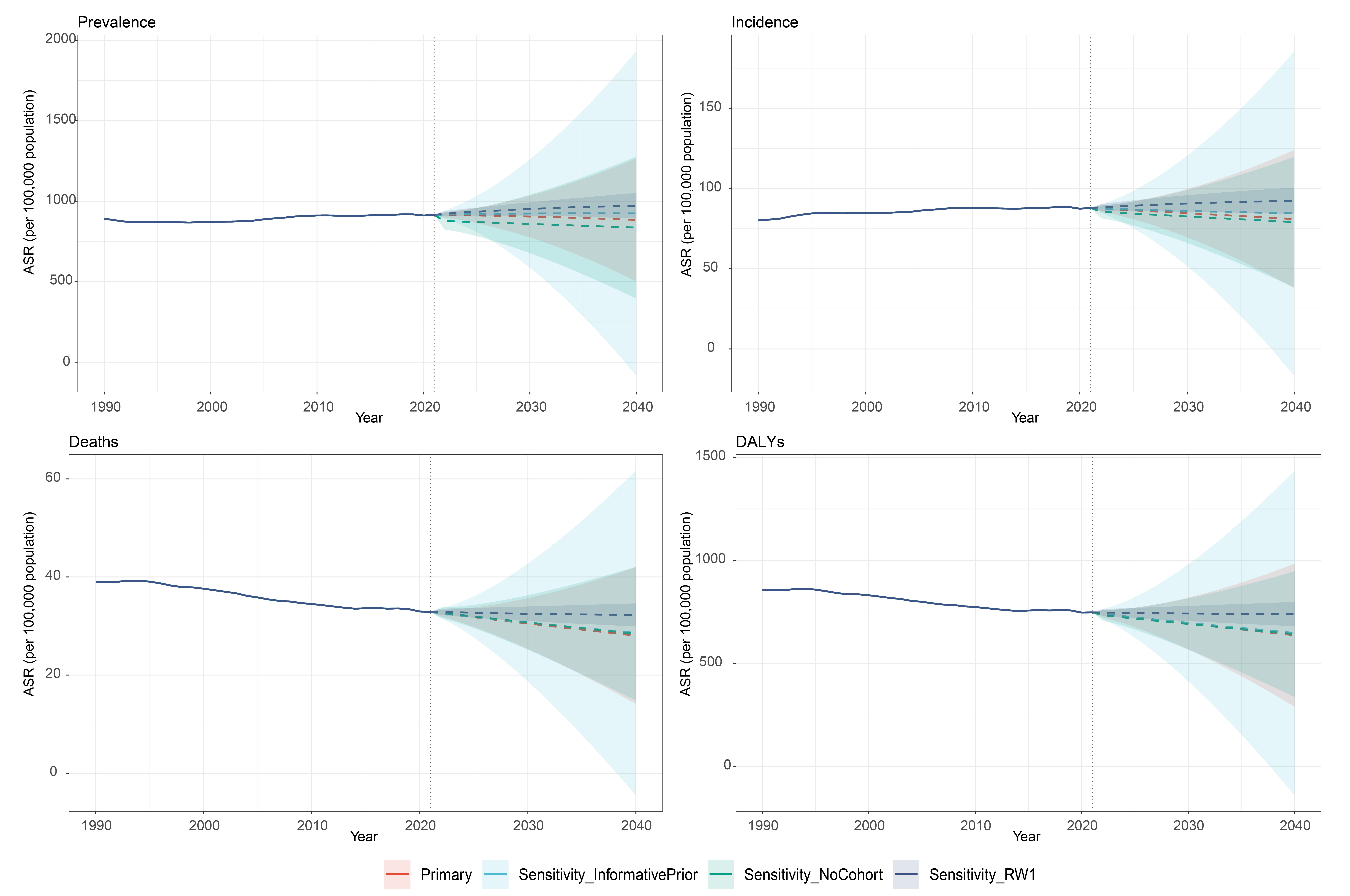


**Fig S4.** BAPC model sensitivity analysis. This panel offers future projections of the age-standardized rates (ASPR, ASIR, ASMR, ASDR, per 100,000 population) for breast cancer at the global level. This figure presents the results of the model sensitivity analysis, including: (1) a model using first-order random walks (RW1) for more linear trends; (2) a model with a more informative (stricter) prior for smoothing parameters; and (3) an age-period model excluding the cohort effect to assess its overall impact.

**Table S1. Prevalence of Breast Cancer Between 1990 and 2021 at the Global and Regional Level**

| **location** | **Number 1990** | **ASR 1990**  **(95% CI)** | **Number 2021** | **ASR 2021**  **(95% CI)** | **EAPC**  **(95%CI)** |
| --- | --- | --- | --- | --- | --- |
| Global | 5675458.9 (5129012.5-6360073.4) | 845.3 (763.9-947.2) | 13445598.7 (12595043.1-14206323.6) | 904.8 (847.6-956) | 0.32 (0.29 to 0.34) |
| High SDI | 3484795 (3160807.4-3873044.9) | 1868.9 (1695.1-2077.1) | 6289444.3 (5860424.6-6657145.4) | 1823 (1698.6-1929.5) | -0.02 (-0.07 to 0.03) |
| High-middle SDI | 1385645.5 (1236596.9-1586909.7) | 803.2 (716.8-919.8) | 3419335.7 (3157078.2-3694476.9) | 986.3 (910.7-1065.7) | 0.78 (0.75 to 0.82) |
| Middle SDI | 528165.7 (465766.8-603254.8) | 304.3 (268.4-347.6) | 2603046.8 (2380356.1-2837406.1) | 554 (506.6-603.9) | 2.09 (2 to 2.19) |
| Low-middle SDI | 195595.2 (172270-224865.7) | 194 (170.9-223.1) | 877396.6 (808321.3-947288.6) | 363.9 (335.3-392.9) | 2.19 (2.07 to 2.31) |
| Low SDI | 74703.7 (64736.4-86462.1) | 200.2 (173.5-231.8) | 242470.7 (219356.7-264719.1) | 295.5 (267.3-322.6) | 1.3 (1.13 to 1.47) |
| Andean Latin America | 8576.1 (7297.2-9888.8) | 255.6 (217.4-294.7) | 47233.1 (38177.7-58806.8) | 476.8 (385.4-593.6) | 2.06 (1.99 to 2.13) |
| Australasia | 75146.3 (66883-85641.5) | 1907.5 (1697.7-2173.9) | 169744.5 (155559.5-183276.5) | 1921.4 (1760.9-2074.6) | 0.15 (0.05 to 0.25) |
| Caribbean | 27716.4 (25546.8-30355.1) | 643.1 (592.8-704.3) | 81566.7 (72321.8-90911) | 881 (781.1-981.9) | 1.19 (1.09 to 1.29) |
| Central Asia | 46771 (42191.6-52218.4) | 584.8 (527.5-652.9) | 81256.6 (74953.1-88232.6) | 558.5 (515.1-606.4) | 0 (-0.05 to 0.05) |
| Central Europe | 229389.1 (205147.3-260968.5) | 865 (773.5-984) | 466448.5 (437280.5-495177) | 1259.7 (1180.9-1337.3) | 1.4 (1.3 to 1.5) |
| Central Latin America | 55100.2 (51087.3-59590.1) | 406 (376.5-439.1) | 344151.6 (308969.2-380032.8) | 804.7 (722.5-888.6) | 2.2 (2.15 to 2.25) |
| Central Sub-Saharan Africa | 9074 (6916.5-11534.9) | 241.3 (183.9-306.8) | 32090 (25223.5-40391.5) | 355.6 (279.5-447.6) | 1.28 (1.1 to 1.46) |
| East Asia | 436883.7 (368029.7-516892) | 293.3 (247.1-347) | 2375001.5 (2004621-2802934) | 605.7 (511.2-714.8) | 2.77 (2.6 to 2.94) |
| Eastern Europe | 412349.8 (368007.4-472049.9) | 843.4 (752.7-965.5) | 703775.7 (645364.9-763008.6) | 1133.7 (1039.6-1229.1) | 0.9 (0.85 to 0.94) |
| Eastern Sub-Saharan Africa | 30766.3 (26637.7-36181) | 252.9 (219-297.4) | 101761 (90028.3-114704.3) | 376.4 (333-424.2) | 1.29 (1.16 to 1.43) |
| High-income Asia Pacific | 247802.8 (211030.3-293823.7) | 708.7 (603.5-840.3) | 853049.4 (761902.9-923076.6) | 1209.9 (1080.7-1309.3) | 2.25 (2.1 to 2.41) |
| High-income North America | 1655322.5 (1516937.1-1824341.4) | 2857.5 (2618.7-3149.3) | 2626218.7 (2441784.3-2790350.6) | 2333.7 (2169.8-2479.6) | -0.92 (-1.02 to -0.82) |
| North Africa and Middle East | 86138.3 (74198.5-101215.1) | 304.8 (262.5-358.1) | 573480.3 (524016.6-622895.1) | 752.3 (687.4-817.1) | 3.65 (3.33 to 3.98) |
| Oceania | 1679.2 (1414.7-2006.3) | 349 (294.1-417) | 4832.5 (4198.1-5581.2) | 391.6 (340.2-452.2) | 0.27 (0.21 to 0.32) |
| South Asia | 149195.6 (130051.6-171074.7) | 157.1 (137-180.2) | 750249.8 (664032.3-849629.6) | 302.2 (267.4-342.2) | 2.1 (1.96 to 2.24) |
| Southeast Asia | 119806.2 (101421.3-143647) | 283 (239.5-339.3) | 592125.3 (518301.8-682084.5) | 516.9 (452.4-595.4) | 2.1 (2.06 to 2.14) |
| Southern Latin America | 74825.4 (66745.2-84022.3) | 944.6 (842.6-1060.7) | 154792.1 (143796.6-165633.3) | 1051.9 (977.1-1125.5) | 0.53 (0.4 to 0.67) |
| Southern Sub-Saharan Africa | 16383.2 (13213.5-19263.9) | 370.3 (298.6-435.4) | 60726.5 (56182-65657.8) | 623.8 (577.1-674.4) | 1.95 (1.86 to 2.04) |
| Tropical Latin America | 63374.3 (58606.6-68496.8) | 418.6 (387.1-452.4) | 284527.5 (263666.1-302987.4) | 642.3 (595.2-684) | 1.28 (1.21 to 1.34) |
| Western Europe | 1891552.7 (1692591.8-2137591.6) | 1947.8 (1742.9-2201.2) | 2995238.8 (2788352.5-3170207.2) | 2008.4 (1869.7-2125.7) | 0.32 (0.21 to 0.43) |
| Western Sub-Saharan Africa | 37605.7 (31821-43179.9) | 260.5 (220.4-299.1) | 147328.7 (122308-179145.9) | 458.4 (380.5-557.3) | 1.97 (1.74 to 2.19) |

**Table S2. Deaths of Breast Cancer Between 1990 and 2021 at the Global and Regional Level**

| **location** | **Number 1990** | **ASR 1990**  **(95% CI)** | **Number 2021** | **ASR 2021**  **(95% CI)** | **EAPC**  **(95%CI)** |
| --- | --- | --- | --- | --- | --- |
| Global | 238913.4 (223054.4-249779.1) | 35.6 (33.2-37.2) | 473476.5 (428994-508325.5) | 31.9 (28.9-34.2) | -0.46 (-0.5 to -0.41) |
| High SDI | 112673.8 (104275.5-117053) | 60.4 (55.9-62.8) | 147971.6 (128248.7-158356.4) | 42.9 (37.2-45.9) | -1.23 (-1.28 to -1.17) |
| High-middle SDI | 64355.8 (60604.3-67171.9) | 37.3 (35.1-38.9) | 114291.2 (102333.5-123847) | 33 (29.5-35.7) | -0.54 (-0.67 to -0.41) |
| Middle SDI | 35517.4 (32560.6-38827.7) | 20.5 (18.8-22.4) | 117986.1 (106020.7-130990.1) | 25.1 (22.6-27.9) | 0.54 (0.48 to 0.6) |
| Low-middle SDI | 17386.6 (15283-19688.5) | 17.2 (15.2-19.5) | 66913.2 (59543-73575.3) | 27.8 (24.7-30.5) | 1.57 (1.53 to 1.61) |
| Low SDI | 8623.6 (7412.7-9880.3) | 23.1 (19.9-26.5) | 25683.1 (22749.5-28656.3) | 31.3 (27.7-34.9) | 0.98 (0.88 to 1.07) |
| Andean Latin America | 789.8 (665.8-939.6) | 23.5 (19.8-28) | 2733.8 (2139.4-3535.6) | 27.6 (21.6-35.7) | 0.33 (0.22 to 0.44) |
| Australasia | 2462 (2261.2-2630.1) | 62.5 (57.4-66.8) | 3663.2 (3066.1-4198.4) | 41.5 (34.7-47.5) | -1.46 (-1.53 to -1.39) |
| Caribbean | 1710.5 (1577.2-1853) | 39.7 (36.6-43) | 4088.6 (3543.3-4653.8) | 44.2 (38.3-50.3) | 0.45 (0.36 to 0.53) |
| Central Asia | 3203.3 (2988-3396.9) | 40.1 (37.4-42.5) | 4388.8 (3940.6-4869.8) | 30.2 (27.1-33.5) | -0.48 (-0.64 to -0.33) |
| Central Europe | 12933.3 (12313.9-13514) | 48.8 (46.4-51) | 21824.7 (19760.6-23653.7) | 58.9 (53.4-63.9) | 0.42 (0.3 to 0.53) |
| Central Latin America | 3204.4 (3064.9-3310.8) | 23.6 (22.6-24.4) | 12870.4 (11327.4-14398.4) | 30.1 (26.5-33.7) | 0.68 (0.59 to 0.78) |
| Central Sub-Saharan Africa | 1053.9 (749.3-1393.5) | 28 (19.9-37.1) | 3418.7 (2529.4-4487.3) | 37.9 (28-49.7) | 0.98 (0.91 to 1.06) |
| East Asia | 23108.6 (19256.6-27585.4) | 15.5 (12.9-18.5) | 65214 (52337.1-80094.5) | 16.6 (13.3-20.4) | 0.02 (-0.07 to 0.11) |
| Eastern Europe | 20479.8 (19654.9-21165.7) | 41.9 (40.2-43.3) | 29702.9 (26583.3-33273.5) | 47.8 (42.8-53.6) | 0.06 (-0.19 to 0.32) |
| Eastern Sub-Saharan Africa | 3939.1 (3343.6-4748.4) | 32.4 (27.5-39) | 11453.5 (9980.7-13353.8) | 42.4 (36.9-49.4) | 0.84 (0.76 to 0.91) |
| High-income Asia Pacific | 4498.9 (4187.8-4692.4) | 12.9 (12-13.4) | 16436.2 (13190.7-18277.7) | 23.3 (18.7-25.9) | 2.06 (1.98 to 2.13) |
| High-income North America | 42195.1 (38820.1-44093.2) | 72.8 (67-76.1) | 51049.9 (44977.5-54477.3) | 45.4 (40-48.4) | -1.82 (-1.9 to -1.74) |
| North Africa and Middle East | 3560.6 (3253.4-3929.8) | 12.6 (11.5-13.9) | 17191.5 (15228.9-19339.3) | 22.6 (20-25.4) | 2.4 (2.15 to 2.66) |
| Oceania | 150.2 (119.4-188.1) | 31.2 (24.8-39.1) | 447.1 (367-539.3) | 36.2 (29.7-43.7) | 0.45 (0.41 to 0.5) |
| South Asia | 14517.5 (12545-16545.8) | 15.3 (13.2-17.4) | 61153.8 (52838.2-71109.7) | 24.6 (21.3-28.6) | 1.46 (1.35 to 1.56) |
| Southeast Asia | 10348.9 (8652.6-12524.1) | 24.4 (20.4-29.6) | 39390.5 (32752.7-48049.4) | 34.4 (28.6-41.9) | 1.07 (0.96 to 1.18) |
| Southern Latin America | 5339 (4990.2-5649.6) | 67.4 (63-71.3) | 8082 (7170.5-8834.7) | 54.9 (48.7-60) | -0.6 (-0.8 to -0.4) |
| Southern Sub-Saharan Africa | 1603.2 (1281.9-1946.8) | 36.2 (29-44) | 5540.8 (5063.7-6054) | 56.9 (52-62.2) | 1.68 (1.41 to 1.96) |
| Tropical Latin America | 5113 (4806-5353) | 33.8 (31.7-35.4) | 16499 (14972.3-17651.5) | 37.2 (33.8-39.8) | 0.15 (0.07 to 0.23) |
| Western Europe | 74000.5 (68450.4-77211.4) | 76.2 (70.5-79.5) | 82775.4 (69888.5-89714.1) | 55.5 (46.9-60.2) | -1.05 (-1.1 to -1.01) |
| Western Sub-Saharan Africa | 4702.1 (3899-5514.6) | 32.6 (27-38.2) | 15551.9 (12529.8-19349.9) | 48.4 (39-60.2) | 1.38 (1.23 to 1.53) |

**Table S3. DALYs of Breast Cancer Between 1990 and 2021 at the Global and Regional Level**

| **location** | **Number 1990** | **ASR 1990**  **(95% CI)** | **Number 2021** | **ASR 2021**  **(95% CI)** | **EAPC**  **(95%CI)** |
| --- | --- | --- | --- | --- | --- |
| Global | 5651007.3 (5336459.5-5939102.4) | 841.6 (794.8-884.6) | 11058137.8 (10223544.4-11869597.9) | 744.2 (688-798.8) | -0.48 (-0.52 to -0.45) |
| High SDI | 2520402 (2373430.1-2629587.5) | 1351.7 (1272.9-1410.2) | 3123806.8 (2806713.6-3354206.5) | 905.4 (813.5-972.2) | -1.35 (-1.38 to -1.32) |
| High-middle SDI | 1553895.4 (1469471.1-1625043.7) | 900.7 (851.7-941.9) | 2635532.5 (2412932.3-2891873.8) | 760.2 (696-834.2) | -0.7 (-0.8 to -0.6) |
| Middle SDI | 902465 (826137.9-992495.3) | 520 (476-571.8) | 2970652.2 (2666905.1-3318540.9) | 632.2 (567.6-706.3) | 0.52 (0.47 to 0.57) |
| Low-middle SDI | 447057.2 (392542-508982.4) | 443.5 (389.4-504.9) | 1682190.1 (1496560.8-1855806.7) | 697.8 (620.8-769.8) | 1.48 (1.44 to 1.53) |
| Low SDI | 218896.7 (187649.9-252566.4) | 586.7 (503-677) | 632243.6 (555268.7-710934.2) | 770.5 (676.7-866.4) | 0.84 (0.73 to 0.95) |
| Andean Latin America | 18913.8 (15837.3-22628.4) | 563.6 (471.9-674.3) | 64341.4 (50351.3-82549.2) | 649.5 (508.3-833.3) | 0.23 (0.13 to 0.34) |
| Australasia | 55897.2 (52136.8-59507.3) | 1418.9 (1323.4-1510.5) | 78460.7 (68586.9-87940.1) | 888.1 (776.4-995.4) | -1.59 (-1.65 to -1.53) |
| Caribbean | 39474.6 (36118-43533) | 915.9 (838.1-1010.1) | 91886.4 (78920.9-105565.2) | 992.5 (852.4-1140.2) | 0.36 (0.28 to 0.44) |
| Central Asia | 80812 (75338.1-85944.5) | 1010.4 (942-1074.6) | 113379.9 (101552-126139.1) | 779.3 (698-866.9) | -0.51 (-0.6 to -0.42) |
| Central Europe | 303146.8 (288850.8-317172.3) | 1143.1 (1089.2-1196) | 451024.3 (415893.5-485436.5) | 1218.1 (1123.2-1311) | 0.06 (-0.05 to 0.17) |
| Central Latin America | 77284.8 (74506.1-79967) | 569.5 (549-589.3) | 317858.7 (280793.2-357682) | 743.2 (656.6-836.4) | 0.75 (0.65 to 0.84) |
| Central Sub-Saharan Africa | 28168.8 (19664.3-37938) | 749.1 (522.9-1008.9) | 88574.1 (64052.8-118179.6) | 981.6 (709.8-1309.7) | 0.89 (0.78 to 1.01) |
| East Asia | 602115.3 (502721.4-723229.4) | 404.2 (337.5-485.5) | 1698475.2 (1357058.8-2096766.9) | 433.2 (346.1-534.7) | 0.11 (0.06 to 0.17) |
| Eastern Europe | 508760 (488572-528043) | 1040.6 (999.3-1080) | 689942.1 (619587.2-777994.5) | 1111.4 (998.1-1253.2) | -0.2 (-0.43 to 0.03) |
| Eastern Sub-Saharan Africa | 97173.9 (82120.7-116805.4) | 798.8 (675-960.1) | 270974.1 (233726.9-316917) | 1002.2 (864.5-1172.1) | 0.67 (0.58 to 0.75) |
| High-income Asia Pacific | 122091.2 (113908.3-129154.9) | 349.1 (325.7-369.3) | 356019.4 (302298.3-393036.1) | 505 (428.8-557.5) | 1.36 (1.23 to 1.48) |
| High-income North America | 979938.3 (915678.2-1031896.8) | 1691.6 (1580.7-1781.3) | 1148536.6 (1047450.1-1236042) | 1020.6 (930.8-1098.4) | -1.82 (-1.88 to -1.77) |
| North Africa and Middle East | 94200.2 (85510.9-104628) | 333.3 (302.5-370.2) | 448276.5 (396203.3-508751.5) | 588 (519.7-667.4) | 2.29 (2.06 to 2.53) |
| Oceania | 4010.2 (3144.5-5145.8) | 833.6 (653.6-1069.6) | 11854.6 (9578.5-14496.7) | 960.5 (776.1-1174.6) | 0.45 (0.4 to 0.5) |
| South Asia | 383662.8 (333279.3-434886.4) | 404.1 (351-458.1) | 1540232.5 (1329846.8-1795876) | 620.3 (535.6-723.3) | 1.26 (1.14 to 1.39) |
| Southeast Asia | 267844.1 (220703.1-328256.6) | 632.6 (521.2-775.3) | 1008167.7 (832151.4-1232733.9) | 880.1 (726.4-1076.1) | 1.06 (0.98 to 1.14) |
| Southern Latin America | 120408.9 (112995.4-127078.2) | 1520 (1426.4-1604.2) | 169157.1 (153380.4-183184.1) | 1149.5 (1042.3-1244.8) | -0.82 (-0.97 to -0.66) |
| Southern Sub-Saharan Africa | 36093.1 (28830.4-43831.4) | 815.7 (651.6-990.6) | 130034.9 (118552.3-142138.4) | 1335.7 (1217.8-1460) | 1.89 (1.62 to 2.16) |
| Tropical Latin America | 124198 (117839.9-129755) | 820.3 (778.3-857) | 386003.8 (353407.8-410823.3) | 871.4 (797.8-927.4) | 0 (-0.07 to 0.07) |
| Western Europe | 1594163.1 (1499042.1-1667198.1) | 1641.6 (1543.6-1716.8) | 1605816.4 (1412258.6-1743785.8) | 1076.8 (947-1169.3) | -1.39 (-1.43 to -1.35) |
| Western Sub-Saharan Africa | 112650 (93217.1-132295.8) | 780.4 (645.7-916.5) | 389121.2 (308835.1-492540.4) | 1210.6 (960.8-1532.3) | 1.54 (1.36 to 1.72) |

**Table S4. Incidence of Breast Cancer Between 1990 and 2021 at the National Level**

| **location** | **Number 1990** | **ASR 1990 (95%UI)** | **Number 2021** | **ASR 2021 (95%UI)** | **EAPC**  **(95%CI)** |
| --- | --- | --- | --- | --- | --- |
| Afghanistan | 245.4 (145.5-390.7) | 20.2 (12-32.1) | 548.4 (328-870.7) | 44.6 (26.7-70.9) | 2.82 (2.62 to 3.02) |
| Albania | 97.5 (77.2-124.4) | 28.1 (22.2-35.8) | 395.5 (278.5-534.6) | 50.3 (35.4-68) | 2.48 (2.25 to 2.72) |
| Algeria | 447.5 (334.9-577.9) | 21.4 (16-27.6) | 2678 (1995.5-3550.3) | 44.1 (32.9-58.5) | 2.44 (2.34 to 2.55) |
| American Samoa | 2.5 (2.1-3.1) | 68.7 (56.4-84.2) | 9.1 (7.1-11.5) | 107.3 (84.6-135.6) | 1.61 (1.52 to 1.7) |
| Andean Latin America | 1112.6 (934.9-1322.8) | 33.2 (27.9-39.4) | 5803.3 (4461.8-7529.6) | 58.6 (45-76) | 1.64 (1.51 to 1.77) |
| Andorra | 12.5 (8.3-18.1) | 126.3 (83.8-183.6) | 37.1 (25.2-51.5) | 140.2 (95.3-194.6) | 0.74 (0.46 to 1.01) |
| Angola | 173.9 (121.5-250) | 27.9 (19.5-40.1) | 963.3 (654.4-1324.2) | 49.8 (33.8-68.5) | 1.91 (1.81 to 2.01) |
| Antigua and Barbuda | 10.2 (8.9-11.4) | 117.6 (103.5-131.8) | 33.4 (30.5-37) | 177.2 (161.6-196.1) | 1.28 (1.07 to 1.5) |
| Argentina | 5895 (5434.8-6359.8) | 105.9 (97.7-114.3) | 10691.5 (9510.1-11781.2) | 114.6 (101.9-126.2) | 0.29 (0.06 to 0.53) |
| Armenia | 479.4 (438.7-522.7) | 98.5 (90.1-107.4) | 897.1 (765.1-1038.8) | 114 (97.3-132.1) | 0.33 (0.03 to 0.63) |
| Australasia | 6279.9 (5834.4-6695.5) | 159.4 (148.1-170) | 14095.6 (12197.9-15771.6) | 159.6 (138.1-178.5) | -0.07 (-0.23 to 0.08) |
| Australia | 4884.9 (4514.4-5268.5) | 148.9 (137.6-160.5) | 11689.7 (10146.3-13242.4) | 157.8 (137-178.8) | 0.05 (-0.14 to 0.24) |
| Austria | 3139 (2849.6-3403.1) | 161.3 (146.4-174.9) | 3891.3 (3373.6-4357.5) | 131.7 (114.2-147.5) | -0.54 (-0.68 to -0.39) |
| Azerbaijan | 433.8 (364.7-495.2) | 49.7 (41.8-56.8) | 1097.7 (815.9-1430.6) | 57.3 (42.6-74.7) | 0.71 (0.46 to 0.97) |
| Bahamas | 32.8 (29.3-36.4) | 133.6 (119.3-148.3) | 119.4 (97.8-143.6) | 165.9 (135.9-199.5) | 1.01 (0.79 to 1.22) |
| Bahrain | 25.2 (20.6-30.9) | 89.9 (73.6-110.2) | 256.8 (188-354.3) | 158.2 (115.8-218.2) | 1.2 (0.84 to 1.56) |
| Bangladesh | 593.6 (453.4-781.7) | 7.8 (6-10.3) | 3195.7 (2368.8-4233.9) | 13.7 (10.1-18.1) | 1.37 (1.13 to 1.61) |
| Barbados | 67.6 (60.2-74.4) | 144.2 (128.4-158.6) | 186.1 (147.4-231.4) | 204.3 (161.8-254) | 1.4 (1.13 to 1.67) |
| Belarus | 1716.8 (1556.2-1895.7) | 74.2 (67.2-81.9) | 3102 (2446.6-3863.5) | 107.8 (85.1-134.3) | 0.42 (0.17 to 0.66) |
| Belgium | 5343.3 (4831.2-5754.2) | 203.8 (184.3-219.5) | 6660.5 (5751.5-7460.5) | 176.2 (152.1-197.3) | -0.38 (-0.55 to -0.21) |
| Belize | 5 (4.5-5.5) | 32.5 (29.1-35.9) | 27.1 (23.4-31.3) | 54.2 (46.6-62.5) | 1.55 (1.28 to 1.81) |
| Benin | 88.2 (70.3-106.8) | 27.6 (22-33.4) | 305.5 (234.2-403.4) | 36.9 (28.3-48.8) | 0.92 (0.86 to 0.98) |
| Bermuda | 21.5 (19.2-24.2) | 203.3 (181.5-228.6) | 43.4 (35.2-56.8) | 186.4 (151.3-243.9) | -0.74 (-0.94 to -0.54) |
| Bhutan | 6.1 (4.2-8.3) | 15.5 (10.6-21) | 24.5 (17.3-33.9) | 24.6 (17.3-34) | 1.55 (1.41 to 1.68) |
| Bolivia (Plurinational State of) | 213.9 (134.1-321.5) | 40.8 (25.6-61.3) | 974.7 (607.8-1468) | 63.1 (39.3-95) | 1.33 (1.28 to 1.39) |
| Bosnia and Herzegovina | 351.6 (296.4-409.5) | 47.9 (40.4-55.8) | 1102.8 (876.8-1351.4) | 100.7 (80.1-123.4) | 3.15 (2.79 to 3.51) |
| Botswana | 39 (27-51.5) | 41.9 (29-55.4) | 156.7 (119.5-206.9) | 64.6 (49.3-85.3) | 1.87 (1.56 to 2.19) |
| Brazil | 7929.9 (7498.2-8305.9) | 53.7 (50.7-56.2) | 33437.1 (30631.9-35835.2) | 77.2 (70.7-82.7) | 0.92 (0.81 to 1.03) |
| Brunei Darussalam | 7.6 (5.6-10.3) | 48.6 (36-65.7) | 44.2 (33.4-56.4) | 73.9 (55.8-94.3) | 1.89 (1.72 to 2.07) |
| Bulgaria | 1962.7 (1715.6-2255.2) | 86.7 (75.8-99.6) | 3699.4 (3000.9-4444.2) | 155.6 (126.3-187) | 2.08 (1.93 to 2.22) |
| Burkina Faso | 291.5 (222.8-368.8) | 40.3 (30.8-51) | 784 (576.8-1028.5) | 52.1 (38.4-68.4) | 0.84 (0.74 to 0.93) |
| Burundi | 154.8 (112.2-213.3) | 41.1 (29.8-56.7) | 279.7 (213.6-368.6) | 34.9 (26.7-46) | -0.93 (-1.1 to -0.77) |
| Cabo Verde | 15.3 (12.1-19.1) | 38.7 (30.7-48.5) | 43.2 (32.2-56.6) | 56.4 (42-73.9) | 1.6 (1.39 to 1.8) |
| Cambodia | 234.5 (147.6-361.5) | 31.6 (19.9-48.8) | 1319.5 (941.9-1787.3) | 60.7 (43.3-82.2) | 2.2 (2.15 to 2.25) |
| Cameroon | 262.6 (203.6-335.8) | 35.5 (27.5-45.4) | 995.5 (698.2-1381.7) | 48.9 (34.3-67.9) | 0.92 (0.81 to 1.04) |
| Canada | 11411.7 (10306.2-12427.7) | 209 (188.8-227.7) | 19281.4 (16792.1-21640.9) | 157.1 (136.8-176.4) | -1.03 (-1.18 to -0.88) |
| Caribbean | 3301.6 (3077.8-3546.6) | 76.6 (71.4-82.3) | 9634.6 (8309.7-10979.8) | 104.1 (89.8-118.6) | 1.09 (0.97 to 1.2) |
| Central African Republic | 66.1 (47.7-88.6) | 35 (25.3-46.9) | 149.9 (105.2-204.2) | 41.9 (29.4-57.1) | 0.53 (0.49 to 0.58) |
| Central Asia | 5037.5 (4692.5-5354.2) | 63 (58.7-66.9) | 8442.6 (7545.3-9332.1) | 58 (51.9-64.1) | 0.14 (0.05 to 0.24) |
| Central Europe | 22093 (21067.2-23051.9) | 83.3 (79.4-86.9) | 48051.8 (43747.7-51898) | 129.8 (118.1-140.2) | 1.35 (1.19 to 1.51) |
| Central Latin America | 6588.4 (6344.8-6810.8) | 48.6 (46.8-50.2) | 40168.8 (35308.8-45043.2) | 93.9 (82.6-105.3) | 1.95 (1.87 to 2.04) |
| Central Sub-Saharan Africa | 1248 (877.7-1669.1) | 33.2 (23.3-44.4) | 4548.5 (3337.4-6009.8) | 50.4 (37-66.6) | 1.35 (1.21 to 1.48) |
| Chad | 98.8 (71-130.9) | 21.3 (15.3-28.3) | 254.6 (188.8-332.7) | 27.4 (20.3-35.8) | 0.82 (0.79 to 0.85) |
| Chile | 998.6 (905.7-1091.7) | 59.4 (53.9-64.9) | 3257 (2847.5-3682.3) | 72.7 (63.6-82.2) | 0.89 (0.75 to 1.03) |
| China | 37880.4 (31283.6-45580.2) | 26.4 (21.8-31.8) | 222613.9 (175869.3-276986.2) | 58.7 (46.4-73.1) | 2.74 (2.65 to 2.84) |
| Colombia | 1775.6 (1626.1-1938.1) | 61.7 (56.5-67.3) | 10444.6 (8676.1-12423.6) | 109.2 (90.7-129.9) | 1.71 (1.53 to 1.88) |
| Comoros | 12.7 (9.3-16.8) | 39.1 (28.7-51.9) | 50.9 (37.7-67.3) | 63 (46.6-83.3) | 1.49 (1.44 to 1.54) |
| Congo | 96.3 (57.5-149.6) | 54.1 (32.3-84) | 324.5 (201.8-498.3) | 74.7 (46.4-114.7) | 1.01 (0.9 to 1.11) |
| Cook Islands | 2.2 (1.7-2.8) | 102.6 (78.8-130.8) | 7.1 (5.4-8.9) | 149.7 (114.8-189.7) | 1.25 (1.16 to 1.35) |
| Costa Rica | 209 (186.7-234.1) | 73.5 (65.7-82.3) | 1368.1 (1154.2-1607.7) | 142.9 (120.6-167.9) | 2.29 (2.14 to 2.44) |
| Croatia | 1298.4 (1113.5-1488.4) | 116.6 (100-133.7) | 2164.4 (1828-2521.3) | 145.1 (122.6-169.1) | 0.93 (0.72 to 1.14) |
| Cuba | 1501 (1356.9-1628.6) | 88.6 (80.1-96.1) | 4167.4 (3513.2-4950.5) | 122 (102.8-144.9) | 1.17 (1.01 to 1.33) |
| Cyprus | 165.1 (133.2-202.6) | 118.2 (95.3-145) | 657.5 (532.2-808.6) | 188.1 (152.3-231.4) | 1.97 (1.59 to 2.35) |
| Czechia | 2887.8 (2574.9-3218.5) | 122 (108.7-135.9) | 4425 (3651.9-5211) | 126.7 (104.5-149.2) | -0.33 (-0.64 to -0.02) |
| Côte d'Ivoire | 251.9 (199.8-317) | 39.3 (31.2-49.5) | 1096.1 (811.8-1490) | 61.1 (45.3-83.1) | 1.52 (1.43 to 1.6) |
| Democratic People's Republic of Korea | 716.8 (462.1-1044.4) | 26.9 (17.4-39.2) | 2084.5 (1471.9-2777) | 37 (26.1-49.3) | 1.33 (1.2 to 1.47) |
| Democratic Republic of the Congo | 847.9 (573.5-1159.1) | 32.1 (21.7-43.9) | 2906.7 (2014.7-4009.9) | 48.1 (33.4-66.4) | 1.32 (1.15 to 1.49) |
| Denmark | 2509.7 (2332-2666.5) | 192.4 (178.7-204.4) | 3022.2 (2641-3375.5) | 157 (137.2-175.3) | -0.48 (-0.76 to -0.2) |
| Djibouti | 8.8 (6.6-11.6) | 40.9 (30.7-54) | 56.5 (39.4-80.5) | 54.5 (38-77.6) | 0.85 (0.8 to 0.9) |
| Dominica | 12.1 (9.8-14.4) | 122.8 (99.4-146.5) | 18.8 (14.3-24.1) | 124.9 (94.5-159.7) | -0.02 (-0.15 to 0.12) |
| Dominican Republic | 195.9 (163.7-234) | 32 (26.8-38.3) | 904.8 (671.4-1187.8) | 54.1 (40.2-71.1) | 1.83 (1.58 to 2.08) |
| East Asia | 39349 (32657.5-47154.2) | 26.4 (21.9-31.7) | 230746.7 (184278.5-285223.4) | 58.8 (47-72.7) | 2.76 (2.66 to 2.85) |
| Eastern Europe | 38156.4 (36743.6-39574.8) | 78 (75.2-80.9) | 73635.3 (66721.7-81371.1) | 118.6 (107.5-131.1) | 1.08 (0.96 to 1.19) |
| Eastern Sub-Saharan Africa | 4522.8 (3845.5-5400.2) | 37.2 (31.6-44.4) | 14871.1 (12853.1-17401.4) | 55 (47.5-64.4) | 1.21 (1.11 to 1.31) |
| Ecuador | 213.5 (196.2-228.4) | 24.8 (22.8-26.6) | 1519.8 (1181.1-1911.3) | 54.9 (42.7-69.1) | 2.62 (2.38 to 2.86) |
| Egypt | 996.7 (868.3-1152.9) | 22.3 (19.4-25.8) | 10599.5 (8590.4-13147.4) | 95.8 (77.6-118.8) | 5.35 (4.83 to 5.88) |
| El Salvador | 138.5 (122.1-156.1) | 28.3 (25-31.9) | 822.2 (653.8-999.5) | 80.4 (63.9-97.7) | 3.35 (3.07 to 3.64) |
| Equatorial Guinea | 10.7 (7.3-15.5) | 33.6 (22.7-48.5) | 65.2 (39.2-98.8) | 82.5 (49.6-124.9) | 3.28 (3.13 to 3.42) |
| Eritrea | 73.6 (53.2-102.1) | 41.6 (30.1-57.8) | 276.4 (195.3-368.5) | 62.7 (44.3-83.6) | 1.36 (1.28 to 1.44) |
| Estonia | 369.8 (335.3-403) | 103.1 (93.5-112.4) | 576.3 (464.7-685.8) | 131.7 (106.2-156.8) | 0.9 (0.77 to 1.03) |
| Eswatini | 20.3 (15-26.9) | 45 (33.2-59.5) | 72.9 (43.9-113.4) | 80.5 (48.5-125.3) | 2.07 (1.8 to 2.34) |
| Ethiopia | 1193.2 (826.5-1688.7) | 37.1 (25.7-52.6) | 3429 (2816.9-4200.2) | 50.1 (41.1-61.4) | 0.9 (0.69 to 1.11) |
| Fiji | 43.1 (33.1-56.2) | 76.5 (58.7-99.8) | 133.5 (96.7-172.5) | 97.6 (70.7-126.2) | 0.66 (0.51 to 0.81) |
| Finland | 1689.9 (1558.6-1809.3) | 142.3 (131.3-152.4) | 3399.4 (2895.3-3819.8) | 168.9 (143.9-189.8) | 0.82 (0.62 to 1.02) |
| France | 22520 (20497.2-24357.6) | 161.8 (147.3-175) | 43725.3 (37640.6-49002.6) | 197.8 (170.2-221.6) | 0.8 (0.64 to 0.96) |
| Gabon | 53.1 (36.8-70.8) | 54.2 (37.6-72.3) | 138.9 (94.5-194.9) | 77.7 (52.8-109) | 0.89 (0.74 to 1.04) |
| Gambia | 8.5 (6.5-10.8) | 15.3 (11.8-19.5) | 44.2 (32.1-58.2) | 28.6 (20.7-37.7) | 1.92 (1.76 to 2.08) |
| Georgia | 1107.1 (985.1-1225.7) | 99.7 (88.7-110.3) | 1445.4 (1241.3-1680.9) | 137.7 (118.2-160.1) | 1.4 (1.05 to 1.75) |
| Germany | 35156.8 (32149.9-37864.7) | 167.5 (153.2-180.5) | 57159 (49162.9-63448.5) | 181.4 (156-201.3) | 0.42 (0.25 to 0.58) |
| Ghana | 398.6 (308.6-504.7) | 39 (30.2-49.4) | 1616.2 (1183.1-2067.8) | 59.3 (43.4-75.8) | 1.2 (1.13 to 1.28) |
| Global | 516906 (486011.4-535689.9) | 77 (72.4-79.8) | 1294277.7 (1183277.6-1383581.9) | 87.1 (79.6-93.1) | 0.36 (0.31 to 0.41) |
| Greece | 3952.3 (3616.7-4257.7) | 147.8 (135.3-159.2) | 6604.5 (5718.8-7280.7) | 179.7 (155.6-198.1) | 0.24 (0.02 to 0.47) |
| Greenland | 5.8 (4.6-7.3) | 104.6 (82.6-131.9) | 9.3 (6.8-12.4) | 68.8 (50.4-91) | -1.57 (-1.77 to -1.38) |
| Grenada | 11.7 (10.4-13.1) | 97 (86.6-108.9) | 28.5 (24.8-32.6) | 141.8 (123.3-162.4) | 1.25 (0.98 to 1.51) |
| Guam | 6.9 (5.5-8.6) | 53.4 (42.8-66.6) | 15.8 (12.3-20.6) | 42 (32.6-54.5) | -0.21 (-0.54 to 0.12) |
| Guatemala | 98 (88.7-107.5) | 17 (15.4-18.7) | 749.1 (636.2-894.5) | 40.9 (34.8-48.9) | 3.05 (2.73 to 3.36) |
| Guinea | 161.6 (119.7-207.7) | 29.2 (21.6-37.5) | 384.1 (276.8-523.1) | 42 (30.3-57.3) | 1.08 (1 to 1.16) |
| Guinea-Bissau | 20 (13.8-29.2) | 31.3 (21.7-45.7) | 54.6 (37-75.7) | 47.6 (32.2-65.9) | 1.34 (1.3 to 1.38) |
| Guyana | 36.9 (31.8-42.3) | 60.2 (51.9-68.9) | 98.1 (74.1-124.9) | 87.1 (65.8-110.9) | 1.36 (1.15 to 1.57) |
| Haiti | 270.2 (163.5-429.9) | 50.1 (30.3-79.7) | 815.1 (504.9-1271.2) | 68.8 (42.6-107.3) | 1.11 (1.01 to 1.21) |
| High SDI | 305050.7 (284950.2-315170.2) | 163.6 (152.8-169) | 537674.1 (477720.3-567855.6) | 155.8 (138.5-164.6) | -0.19 (-0.28 to -0.11) |
| High-income Asia Pacific | 14293 (13189.4-15213.8) | 40.9 (37.7-43.5) | 65901 (54916.6-72762.6) | 93.5 (77.9-103.2) | 2.94 (2.75 to 3.13) |
| High-income North America | 151967.2 (141053-157789.4) | 262.3 (243.5-272.4) | 227930.6 (205006.5-240480.7) | 202.5 (182.2-213.7) | -1.12 (-1.23 to -1.01) |
| High-middle SDI | 123478.4 (116843.8-128695.6) | 71.6 (67.7-74.6) | 325962.3 (293221.3-357859.9) | 94 (84.6-103.2) | 0.82 (0.73 to 0.9) |
| Honduras | 81.4 (50.9-118.1) | 24.4 (15.3-35.4) | 646.4 (466.4-917.7) | 61.5 (44.3-87.3) | 3.01 (2.8 to 3.23) |
| Hungary | 2864.4 (2524.6-3236.6) | 111.2 (98-125.7) | 4516.6 (3839.9-5281.4) | 142 (120.7-166.1) | 0.38 (0.13 to 0.63) |
| Iceland | 84.4 (75.4-94.3) | 177 (157.9-197.6) | 157.8 (135.1-180.2) | 162.1 (138.7-185.1) | -0.3 (-0.55 to -0.06) |
| India | 13630.9 (11388.7-16109.7) | 17.8 (14.9-21) | 77591 (64194.5-93906.3) | 38.6 (31.9-46.7) | 2.51 (2.33 to 2.69) |
| Indonesia | 4939.7 (3189.9-7181.5) | 30.6 (19.7-44.5) | 22437.8 (14466.2-32828) | 53.6 (34.5-78.4) | 1.67 (1.57 to 1.78) |
| Iran (Islamic Republic of) | 1108 (923.8-1318.4) | 24 (20-28.6) | 9608.8 (8471.4-10919.2) | 74 (65.2-84.1) | 4.18 (3.88 to 4.47) |
| Iraq | 474.3 (354-622.9) | 37.6 (28-49.3) | 3726.4 (2557.5-5045.8) | 95.9 (65.8-129.9) | 3.24 (3.05 to 3.42) |
| Ireland | 1188.8 (1096.5-1283.1) | 174.6 (161.1-188.5) | 2078.5 (1785.5-2360.5) | 158.4 (136-179.9) | 0.06 (-0.13 to 0.25) |
| Israel | 1141.3 (1026.1-1271.1) | 140.8 (126.6-156.8) | 2856.2 (2426-3266) | 142.9 (121.4-163.4) | -0.44 (-0.79 to -0.09) |
| Italy | 24900.8 (22954.1-26640.8) | 163.6 (150.8-175) | 37510 (31633.2-41282) | 165.1 (139.3-181.7) | -0.08 (-0.26 to 0.11) |
| Jamaica | 257.7 (231.7-285.6) | 87.5 (78.7-97) | 702 (535.9-888.7) | 132.7 (101.3-168) | 1.19 (0.94 to 1.45) |
| Japan | 13209.4 (12127.2-14141.7) | 44.6 (41-47.8) | 56192.5 (45716-62517) | 107.6 (87.6-119.8) | 3.09 (2.93 to 3.25) |
| Jordan | 111 (85.1-143.9) | 51.6 (39.6-66.9) | 1412.4 (989.1-1903.2) | 112.3 (78.7-151.4) | 3.15 (2.66 to 3.65) |
| Kazakhstan | 1606.9 (1376.8-1855.4) | 76.8 (65.8-88.7) | 2005.1 (1644.6-2385.8) | 63.2 (51.8-75.2) | 0.38 (0.06 to 0.7) |
| Kenya | 410.4 (296.6-551.5) | 30.8 (22.3-41.4) | 2104.3 (1536.6-2847.6) | 56.3 (41.1-76.1) | 2.07 (1.96 to 2.19) |
| Kiribati | 3.1 (2.3-4.1) | 51.7 (38.5-68.5) | 9.1 (6.7-12.1) | 72.3 (53.6-96.6) | 1.07 (0.95 to 1.18) |
| Kuwait | 49.4 (43.1-55.9) | 54 (47.1-61.2) | 381.7 (312.1-451.6) | 81.9 (67-96.9) | 2.14 (1.53 to 2.74) |
| Kyrgyzstan | 289.1 (261-320.8) | 56.1 (50.7-62.3) | 407.4 (337.9-486) | 48.2 (40-57.5) | -0.55 (-0.87 to -0.23) |
| Lao People's Democratic Republic | 97.4 (56.4-159.5) | 28.4 (16.4-46.5) | 355.3 (243-506.8) | 46 (31.4-65.6) | 1.67 (1.62 to 1.71) |
| Latvia | 589 (527.7-665.7) | 94.2 (84.4-106.5) | 869.3 (721-1022.6) | 132.1 (109.6-155.4) | 1.24 (1.05 to 1.43) |
| Lebanon | 306.6 (224.6-413.3) | 80.9 (59.2-109) | 1896.9 (1499.7-2369.4) | 193.6 (153-241.8) | 3.33 (3 to 3.66) |
| Lesotho | 52.8 (39.2-71.8) | 37.8 (28-51.3) | 138.5 (88-200.8) | 76.7 (48.7-111.2) | 2.76 (2.42 to 3.11) |
| Liberia | 49.1 (38.6-60.8) | 26.1 (20.5-32.2) | 138.7 (97.8-189.5) | 42 (29.6-57.4) | 1.72 (1.61 to 1.83) |
| Libya | 97 (76.6-120.2) | 31.2 (24.6-38.6) | 634 (476.6-828.5) | 75.8 (57-99.1) | 3.36 (2.96 to 3.76) |
| Lithuania | 697.6 (621.5-778.9) | 88 (78.4-98.3) | 1108.4 (919.1-1323.9) | 114.5 (95-136.8) | 0.8 (0.59 to 1.01) |
| Low SDI | 10121.6 (8686.8-11602.2) | 27.1 (23.3-31.1) | 34459.9 (30431.8-38406.4) | 42 (37.1-46.8) | 1.39 (1.27 to 1.52) |
| Low-middle SDI | 22384.8 (19714.8-25233.1) | 22.2 (19.6-25) | 110319.2 (98606.5-120600.7) | 45.8 (40.9-50) | 2.38 (2.35 to 2.42) |
| Luxembourg | 164.7 (151.6-177.9) | 176.4 (162.3-190.5) | 265.3 (228.8-299) | 149.3 (128.7-168.2) | -0.07 (-0.35 to 0.22) |
| Madagascar | 304.3 (244.4-379.9) | 36 (28.9-45) | 757 (531.8-1003.5) | 41 (28.8-54.3) | 0.31 (0.13 to 0.49) |
| Malawi | 202.7 (157.4-257.7) | 32.2 (25-41) | 627.4 (467.2-819.5) | 53.7 (40-70.1) | 1.61 (1.54 to 1.68) |
| Malaysia | 755.5 (634.2-885.2) | 51.2 (43-60) | 4534.9 (3807.3-5325.9) | 92.5 (77.7-108.6) | 1.89 (1.8 to 1.98) |
| Maldives | 2.8 (1.7-4.5) | 18.4 (10.9-29.5) | 16.8 (13.1-20.9) | 30.8 (24.1-38.4) | 1.44 (1.06 to 1.82) |
| Mali | 223.5 (174.1-275.1) | 33.4 (26-41.1) | 636.4 (459.5-867.6) | 44 (31.7-59.9) | 0.85 (0.77 to 0.92) |
| Malta | 126.3 (114.7-139) | 174.4 (158.3-191.9) | 246.9 (210-285.6) | 156.4 (133.1-181) | -0.74 (-0.99 to -0.48) |
| Marshall Islands | 1.1 (0.8-1.6) | 45 (31.3-64) | 3.7 (2.2-5.6) | 62.7 (37.7-96.4) | 0.78 (0.69 to 0.87) |
| Mauritania | 62.7 (44.2-85) | 37.7 (26.6-51.2) | 196.7 (146.7-260.7) | 54.8 (40.9-72.7) | 0.97 (0.81 to 1.13) |
| Mauritius | 44.4 (40.1-48.2) | 36.5 (33-39.6) | 298.4 (269.5-324) | 88.2 (79.7-95.8) | 2.42 (2.03 to 2.81) |
| Mexico | 3157.8 (3036.9-3265.9) | 45.4 (43.7-47) | 18381.1 (15497.3-21475.8) | 85.3 (71.9-99.7) | 1.71 (1.59 to 1.84) |
| Micronesia (Federated States of) | 4.6 (3.1-6.3) | 57.6 (39.6-79.2) | 10.5 (7.6-14.3) | 79.6 (57.5-108.4) | 0.89 (0.79 to 0.98) |
| Middle SDI | 55229.9 (50803.2-60423.3) | 31.8 (29.3-34.8) | 284424.8 (255666.6-317342) | 60.5 (54.4-67.5) | 2.03 (1.99 to 2.08) |
| Monaco | 24.7 (17.4-33.2) | 223.1 (156.7-299.8) | 46.5 (35.1-60.8) | 297.1 (224.2-388.9) | 1.1 (0.95 to 1.25) |
| Mongolia | 23.8 (17.6-30.9) | 13.7 (10.2-17.8) | 76.2 (57.9-96.7) | 19.3 (14.7-24.5) | 0.98 (0.87 to 1.09) |
| Montenegro | 125.5 (94.4-163.5) | 115.2 (86.6-150.1) | 289 (220.3-368.2) | 165.8 (126.3-211.2) | 1.49 (1.41 to 1.58) |
| Morocco | 383.2 (301.4-481) | 16.3 (12.9-20.5) | 2556.1 (1752.2-3559.2) | 42.5 (29.2-59.2) | 3.36 (3.25 to 3.46) |
| Mozambique | 318 (258.7-382) | 32.5 (26.5-39.1) | 969.9 (683.4-1261.6) | 54.2 (38.2-70.5) | 1.91 (1.82 to 1.99) |
| Myanmar | 1307.5 (910.2-1847) | 33.4 (23.3-47.2) | 4336.3 (3385-5691.1) | 51.4 (40.1-67.4) | 1.36 (1.32 to 1.4) |
| Namibia | 51.6 (41.3-64.4) | 47.6 (38-59.3) | 243.2 (160.3-334.4) | 107.6 (71-148) | 3.1 (2.96 to 3.24) |
| Nauru | 0.4 (0.3-0.7) | 59.8 (35.7-92.8) | 0.9 (0.5-1.4) | 98.6 (59.1-151.7) | 1.62 (1.55 to 1.68) |
| Nepal | 234.1 (170.1-311.3) | 15.2 (11.1-20.2) | 976.6 (689-1366) | 24.7 (17.4-34.5) | 1.65 (1.39 to 1.91) |
| Netherlands | 6034.1 (5481.2-6520.5) | 182.2 (165.5-196.9) | 9860.8 (8504.6-11054.4) | 168.5 (145.4-188.9) | -0.21 (-0.38 to -0.05) |
| New Zealand | 1395.1 (1258.8-1518.8) | 212 (191.3-230.8) | 2405.9 (2044.8-2700.8) | 168.7 (143.3-189.3) | -0.58 (-0.67 to -0.5) |
| Nicaragua | 57 (47.7-67.5) | 22.9 (19.2-27.2) | 468.1 (368.6-591.9) | 57.5 (45.3-72.7) | 3.44 (3.22 to 3.65) |
| Niger | 78.6 (58.7-106.4) | 17.9 (13.4-24.2) | 329.5 (236.9-452.8) | 23.7 (17-32.5) | 0.93 (0.89 to 0.98) |
| Nigeria | 3202.1 (2446.1-4081.9) | 44 (33.6-56) | 13455.5 (9401-19015) | 89.7 (62.7-126.8) | 2.58 (2.27 to 2.89) |
| Niue | 0.3 (0.2-0.4) | 85.5 (66.2-109) | 0.4 (0.3-0.5) | 104.1 (78.8-134.9) | 0.62 (0.55 to 0.69) |
| North Africa and Middle East | 6607.2 (6004-7298.7) | 23.4 (21.2-25.8) | 58521.6 (52162.6-65378.1) | 76.8 (68.4-85.8) | 4.47 (4.19 to 4.76) |
| North Macedonia | 244.7 (200.4-288.9) | 74.4 (60.9-87.8) | 759.7 (589.4-966.8) | 128.1 (99.4-163.1) | 2.05 (1.73 to 2.37) |
| Northern Mariana Islands | 1.6 (1.2-2.1) | 65.6 (49.5-85.8) | 8.6 (7-10.4) | 88.9 (72.2-107.3) | 0.44 (0.24 to 0.65) |
| Norway | 1459 (1338.8-1547.5) | 134.7 (123.6-142.9) | 1960.8 (1726.6-2162.1) | 120.9 (106.5-133.3) | -0.38 (-0.67 to -0.1) |
| Oceania | 200.4 (159.6-248.5) | 41.7 (33.2-51.7) | 602.9 (496.9-724.7) | 48.8 (40.3-58.7) | 0.38 (0.3 to 0.46) |
| Oman | 16.7 (11.9-22.9) | 16.7 (11.9-22.8) | 114 (85.6-147.7) | 36.5 (27.4-47.3) | 2.84 (2.46 to 3.22) |
| Pakistan | 3645.3 (2770.2-4656) | 40 (30.4-51.1) | 14207.8 (10353.1-18494.6) | 71.7 (52.2-93.3) | 1.59 (1.39 to 1.79) |
| Palau | 1.9 (1.4-2.4) | 117.5 (89.9-152.6) | 4.8 (3.7-6.2) | 115.6 (87.9-149.2) | -0.32 (-0.42 to -0.22) |
| Palestine | 101.4 (74.5-137.9) | 69.8 (51.3-95) | 589.7 (470.5-729.8) | 137 (109.4-169.6) | 2.45 (2.28 to 2.63) |
| Panama | 143.2 (128.8-157.5) | 59.1 (53.2-65) | 836.7 (664.7-1016.5) | 112.5 (89.4-136.7) | 2.12 (1.99 to 2.24) |
| Papua New Guinea | 96 (67.8-138.6) | 32.5 (22.9-46.9) | 304.1 (223.9-415.1) | 37 (27.3-50.5) | 0.21 (0.09 to 0.33) |
| Paraguay | 145.3 (115.7-179.4) | 40 (31.9-49.5) | 771.8 (570.8-1016.4) | 78 (57.7-102.7) | 2.19 (2.01 to 2.37) |
| Peru | 685.3 (552.2-829.8) | 34.8 (28-42.1) | 3308.8 (2353.8-4539.7) | 59.2 (42.1-81.2) | 1.37 (1.15 to 1.58) |
| Philippines | 2143.5 (1887.3-2420.1) | 44.9 (39.5-50.7) | 9739.1 (7834.8-11983.7) | 69.8 (56.2-85.9) | 1.26 (1.13 to 1.39) |
| Poland | 5738.6 (5409.9-6043.6) | 74.5 (70.2-78.4) | 15550.7 (13638.7-17312.7) | 128.4 (112.6-142.9) | 1.72 (1.55 to 1.89) |
| Portugal | 2643.4 (2407.8-2878) | 108.4 (98.8-118.1) | 4974.7 (4290.2-5507.1) | 127.9 (110.3-141.5) | 0.52 (0.35 to 0.7) |
| Puerto Rico | 558.4 (510.5-614.2) | 92 (84.1-101.2) | 1553 (1273.4-1843.9) | 133 (109.1-158) | 1.26 (1.06 to 1.46) |
| Qatar | 11.3 (8.5-14.4) | 69.2 (51.9-88) | 227 (164.2-306.1) | 148.2 (107.2-199.8) | 2.47 (2.25 to 2.7) |
| Republic of Korea | 840.4 (730.9-985.1) | 16.9 (14.7-19.8) | 8119.6 (6175.3-10075.9) | 48.6 (36.9-60.2) | 3.76 (3.43 to 4.09) |
| Republic of Moldova | 639.5 (569.4-720.4) | 82.8 (73.8-93.3) | 1149.6 (958.3-1373.6) | 108.9 (90.8-130.1) | 1.48 (1.16 to 1.8) |
| Romania | 2791.4 (2559.4-3052.4) | 55.8 (51.1-61) | 6869.9 (5970.8-7830.7) | 114.4 (99.4-130.4) | 2.25 (2.08 to 2.42) |
| Russian Federation | 22999.1 (22178.5-23686.5) | 72.9 (70.3-75) | 55592.3 (49847.6-61274.6) | 130.8 (117.3-144.2) | 1.66 (1.51 to 1.81) |
| Rwanda | 245.5 (179.6-337.7) | 52.5 (38.4-72.2) | 680.7 (487.9-949.7) | 64.4 (46.1-89.8) | 0.26 (0.07 to 0.46) |
| Saint Kitts and Nevis | 9.3 (8.4-10.2) | 145 (131.9-159.2) | 17.9 (14.7-21.3) | 138.8 (113.9-165.4) | 0.45 (0.15 to 0.75) |
| Saint Lucia | 15 (13.7-16.4) | 102.8 (93.9-112.4) | 42.4 (34.8-51.1) | 100.7 (82.6-121.4) | -0.63 (-0.89 to -0.37) |
| Saint Vincent and the Grenadines | 12.6 (11.2-13.9) | 104.8 (93.5-115.8) | 28.3 (24.2-32.4) | 111.2 (95.3-127.6) | 0.04 (-0.22 to 0.31) |
| Samoa | 6.4 (4.9-8.1) | 44.5 (33.8-56.4) | 14.7 (11.1-19.8) | 59.7 (44.9-80.4) | 0.76 (0.67 to 0.86) |
| San Marino | 8.1 (6.2-10.2) | 137.2 (105.6-173.9) | 11.2 (6.9-16.4) | 94.4 (58-138.8) | -0.56 (-0.88 to -0.25) |
| Sao Tome and Principe | 3.1 (2.4-3.9) | 27.6 (21.8-35.2) | 9.5 (6.9-12.6) | 51.8 (37.8-69.2) | 2.07 (2.02 to 2.12) |
| Saudi Arabia | 183.3 (138.2-238) | 20.6 (15.5-26.7) | 1766.8 (1378-2313.2) | 58.7 (45.8-76.8) | 3.39 (3.09 to 3.68) |
| Senegal | 140 (110.8-180.8) | 26.5 (21-34.2) | 594.3 (450.2-793.9) | 46 (34.9-61.5) | 1.8 (1.7 to 1.9) |
| Serbia | 1951.4 (1469.6-2526) | 93.8 (70.7-121.5) | 4341.3 (3282.5-5557.8) | 154.8 (117-198.1) | 1.55 (1.41 to 1.68) |
| Seychelles | 4.2 (3.6-4.9) | 43.8 (37.5-51) | 15.7 (13-18.3) | 75.5 (62.7-88.1) | 1.52 (1.07 to 1.98) |
| Sierra Leone | 78 (57.3-100.8) | 23.5 (17.3-30.4) | 225 (166.5-294.6) | 37.2 (27.6-48.8) | 1.68 (1.61 to 1.75) |
| Singapore | 235.6 (214-260.1) | 65 (59-71.7) | 1544.7 (1358.1-1721.5) | 101.7 (89.4-113.3) | 1.97 (1.72 to 2.21) |
| Slovakia | 930.1 (780.9-1108.4) | 89.7 (75.3-106.8) | 2276.7 (1687.6-2926.9) | 138.8 (102.9-178.4) | 1.3 (1.11 to 1.49) |
| Slovenia | 495.6 (446-546.7) | 115.1 (103.6-126.9) | 961.3 (786.4-1153.4) | 131.3 (107.4-157.5) | 0.39 (0.16 to 0.62) |
| Solomon Islands | 5.6 (3.7-8.5) | 24.9 (16.5-37.7) | 24 (16.1-33.7) | 44.2 (29.7-62.1) | 1.8 (1.62 to 1.97) |
| Somalia | 112.4 (78.5-153.8) | 31.2 (21.8-42.7) | 341.1 (230.1-462) | 36.2 (24.4-49) | 0.41 (0.37 to 0.45) |
| South Africa | 1571.3 (1170.9-1981.5) | 46.6 (34.7-58.7) | 6689 (6098.9-7417.2) | 84.8 (77.3-94) | 2.13 (1.94 to 2.31) |
| South Asia | 18110.1 (15679-20538) | 19.1 (16.5-21.6) | 95995.6 (82449.9-112310.7) | 38.7 (33.2-45.2) | 2.19 (2.06 to 2.33) |
| South Sudan | 125.1 (89.1-171.9) | 30.2 (21.5-41.4) | 244.7 (167.1-348.7) | 39.3 (26.8-56) | 0.79 (0.64 to 0.94) |
| Southeast Asia | 13303.3 (11108-16131.3) | 31.4 (26.2-38.1) | 66753.6 (55597.1-80793.1) | 58.3 (48.5-70.5) | 2 (1.91 to 2.09) |
| Southern Latin America | 7823.1 (7295.7-8292.2) | 98.8 (92.1-104.7) | 15422.3 (13816.6-16814.6) | 104.8 (93.9-114.3) | 0.23 (0.03 to 0.44) |
| Southern Sub-Saharan Africa | 2014.9 (1603-2449.9) | 45.5 (36.2-55.4) | 8236.2 (7514-8992.2) | 84.6 (77.2-92.4) | 2.27 (2.06 to 2.48) |
| Spain | 11323.1 (10242.4-12248) | 118.4 (107.1-128.1) | 18187.3 (15372.2-20645.4) | 117.5 (99.3-133.4) | -0.06 (-0.23 to 0.11) |
| Sri Lanka | 474.7 (386.3-583.6) | 26.7 (21.7-32.8) | 2643.4 (1726.9-3696.8) | 54.9 (35.9-76.8) | 2.67 (2.46 to 2.89) |
| Sudan | 198.1 (135.1-277.3) | 13.3 (9.1-18.6) | 913.3 (623.2-1322.1) | 29.3 (20-42.5) | 2.71 (2.54 to 2.88) |
| Suriname | 22.9 (19.3-26.7) | 52.7 (44.4-61.3) | 76.4 (58.2-97.5) | 68.4 (52.1-87.3) | 1.2 (0.99 to 1.41) |
| Sweden | 3578.3 (3242.4-3917.7) | 150.7 (136.6-165) | 4750.5 (3899.1-5550.6) | 141.1 (115.8-164.9) | 0.16 (-0.17 to 0.49) |
| Switzerland | 2553.1 (2306.9-2772.4) | 151.1 (136.6-164.1) | 4111.9 (3537.9-4641.6) | 140.3 (120.7-158.4) | -0.21 (-0.59 to 0.18) |
| Syrian Arab Republic | 226.7 (173.7-287.4) | 25.7 (19.7-32.6) | 1584.9 (1130.3-2121.6) | 67.1 (47.8-89.8) | 3.08 (2.88 to 3.28) |
| Taiwan (Province of China) | 751.8 (698.5-816.9) | 27.1 (25.2-29.5) | 6048.3 (5295.3-6811.9) | 80.3 (70.3-90.5) | 3.75 (3.29 to 4.21) |
| Tajikistan | 174.6 (140.9-210.8) | 37.3 (30.1-45) | 319.7 (216-450.8) | 31.1 (21-43.9) | -0.71 (-0.87 to -0.55) |
| Thailand | 1677.5 (1286.2-2075.8) | 28.3 (21.7-35) | 13070.9 (9816.8-16781) | 67.2 (50.5-86.3) | 3.07 (2.73 to 3.41) |
| Timor-Leste | 9.4 (5.7-14.7) | 21.6 (13.1-33.7) | 50.6 (33.4-72.2) | 35.6 (23.6-50.8) | 1.95 (1.78 to 2.11) |
| Togo | 60.4 (48.3-76.5) | 31 (24.8-39.2) | 307.8 (219.9-421.7) | 49.3 (35.2-67.5) | 1.51 (1.44 to 1.57) |
| Tokelau | 0.1 (0.1-0.2) | 62.6 (41.9-88.3) | 0.2 (0.2-0.3) | 90.4 (65.4-121.8) | 1.2 (1.1 to 1.31) |
| Tonga | 8.3 (6.6-10.4) | 87.5 (69.2-109.4) | 15.1 (11.2-20.1) | 112.5 (83.7-149.3) | 0.68 (0.6 to 0.76) |
| Trinidad and Tobago | 131.8 (120.1-144.3) | 96 (87.5-105.1) | 411.5 (312.8-523.9) | 118.1 (89.8-150.4) | 0.83 (0.63 to 1.03) |
| Tropical Latin America | 8075.1 (7619.1-8452) | 53.3 (50.3-55.8) | 34208.9 (31260.3-36670.1) | 77.2 (70.6-82.8) | 0.95 (0.84 to 1.06) |
| Tunisia | 311.5 (246.8-390.4) | 35.8 (28.3-44.8) | 1871.3 (1319.1-2617.3) | 80.1 (56.4-112) | 2.46 (2.32 to 2.6) |
| Turkey | 1168.7 (913.6-1457.8) | 19.6 (15.3-24.4) | 15935.6 (12162.5-20078.3) | 96.5 (73.6-121.5) | 6.55 (5.74 to 7.36) |
| Turkmenistan | 126.8 (110.7-143.8) | 39 (34.1-44.3) | 297.4 (220.3-397.9) | 42 (31.1-56.2) | 0.52 (-0.01 to 1.06) |
| Tuvalu | 0.6 (0.4-0.8) | 49.7 (32-72.3) | 1.3 (1-1.8) | 73.2 (52.6-98.8) | 1.09 (0.94 to 1.24) |
| Uganda | 493.8 (367.9-643.8) | 47.2 (35.2-61.6) | 1891.8 (1420.3-2504) | 79.8 (59.9-105.6) | 1.33 (1.12 to 1.54) |
| Ukraine | 11144.6 (10139.1-12183) | 89.4 (81.3-97.7) | 11237.4 (7560.7-15949.1) | 82.8 (55.7-117.5) | -0.64 (-0.81 to -0.47) |
| United Arab Emirates | 42.1 (31.8-53.8) | 75.1 (56.6-95.9) | 526.8 (362.3-729.6) | 73.4 (50.5-101.6) | 1.14 (0.54 to 1.74) |
| United Kingdom | 31476.7 (29654.8-32376.1) | 211.9 (199.7-218) | 36840.6 (33194.6-38848.3) | 175.2 (157.8-184.7) | -0.57 (-0.68 to -0.46) |
| United Republic of Tanzania | 707.3 (565.5-895.3) | 39.2 (31.3-49.6) | 2341.2 (1745-3089.1) | 56.8 (42.3-74.9) | 1.19 (1.15 to 1.23) |
| United States Virgin Islands | 17.1 (14-21) | 122.4 (100.6-150.6) | 34.9 (23.6-49.8) | 108.4 (73.2-154.7) | -0.38 (-0.65 to -0.1) |
| United States of America | 140546.3 (130767.8-146155.6) | 267.9 (249.3-278.6) | 208636.2 (187636.6-220534.4) | 208.1 (187.2-220) | -1.11 (-1.23 to -1) |
| Uruguay | 929.1 (854.9-1002) | 137.7 (126.7-148.5) | 1472.9 (1289.5-1637.2) | 162.9 (142.6-181) | 0.37 (0.2 to 0.53) |
| Uzbekistan | 796.1 (687.2-919.5) | 40.7 (35.1-47) | 1896.5 (1518.3-2320.1) | 40.8 (32.7-49.9) | 0.19 (-0.06 to 0.45) |
| Vanuatu | 2.8 (1.9-4) | 28 (19-39.7) | 12.9 (9.7-16.5) | 44.9 (33.8-57.6) | 1.31 (1.18 to 1.43) |
| Venezuela (Bolivarian Republic of) | 927.9 (842.6-1010.6) | 59.3 (53.8-64.5) | 6452.4 (4940.8-8210.7) | 123.3 (94.4-156.9) | 2.28 (2.14 to 2.43) |
| Viet Nam | 1592.8 (1241.5-2113.4) | 22.8 (17.8-30.3) | 7841.8 (5794.9-10449.4) | 44.9 (33.2-59.8) | 2.29 (2.24 to 2.34) |
| Western Europe | 161327.8 (150906.3-167411.8) | 166.1 (155.4-172.4) | 249235.1 (217604.4-267193.8) | 167.1 (145.9-179.2) | 0.08 (-0.07 to 0.24) |
| Western Sub-Saharan Africa | 5494.7 (4518.7-6463.6) | 38.1 (31.3-44.8) | 21471.8 (17085.3-27085.2) | 66.8 (53.2-84.3) | 1.93 (1.74 to 2.12) |
| Yemen | 99.6 (66.4-136) | 12.4 (8.3-17) | 638.8 (439.5-867.4) | 28.4 (19.5-38.6) | 3.04 (2.88 to 3.21) |
| Zambia | 157 (116.9-208.5) | 34.1 (25.4-45.3) | 807.4 (518.9-1203.3) | 74.8 (48-111.4) | 2.74 (2.45 to 3.03) |
| Zimbabwe | 279.9 (212.8-359.4) | 42.2 (32.1-54.2) | 935.9 (687.1-1219.5) | 84.5 (62-110.1) | 2.89 (2.27 to 3.51) |

**Table S5. Prevalence of Breast Cancer Between 1990 and 2021 at the National Level**

| **location** | **Number 1990** | **ASR 1990 (95%UI)** | **Number 2021** | **ASR 2021 (95%UI)** | **EAPC**  **(95%CI)** |
| --- | --- | --- | --- | --- | --- |
| Afghanistan | 2633.5 (1909.1-3594.9) | 216.7 (157.1-295.8) | 4606 (3148.2-6680.4) | 374.9 (256.2-543.7) | 2.14 (1.75 to 2.52) |
| Albania | 1351.1 (1111.7-1671.7) | 389.3 (320.3-481.7) | 4174.3 (3346.6-5180.2) | 530.9 (425.6-658.8) | 1.86 (1.54 to 2.19) |
| Algeria | 6416.8 (5145-7933.5) | 306.3 (245.6-378.7) | 27033.6 (21992.4-33762) | 445.1 (362.1-555.9) | 1.57 (1.28 to 1.87) |
| American Samoa | 21.1 (17.7-25.1) | 577.3 (484.7-685.6) | 73.2 (62.4-86.9) | 866.3 (738.5-1029.1) | 1.55 (1.47 to 1.64) |
| Andean Latin America | 8576.1 (7297.2-9888.8) | 255.6 (217.4-294.7) | 47233.1 (38177.7-58806.8) | 476.8 (385.4-593.6) | 2.06 (1.99 to 2.13) |
| Andorra | 152.2 (117.4-197.8) | 1542.7 (1189.9-2005.1) | 475.6 (386.7-588.2) | 1797.2 (1461.2-2222.9) | 0.99 (0.82 to 1.17) |
| Angola | 1277.7 (965.5-1667.2) | 205.1 (155-267.6) | 6989.8 (5148-9128.2) | 361.5 (266.2-472.1) | 1.98 (1.8 to 2.16) |
| Antigua and Barbuda | 82.1 (73.1-91.3) | 950.1 (845.6-1056.9) | 274.1 (253.3-300.4) | 1453 (1342.5-1592.3) | 1.45 (1.27 to 1.64) |
| Argentina | 55448.4 (49456.8-62474.6) | 996.4 (888.8-1122.7) | 106243.7 (97941.8-114184.1) | 1138.3 (1049.4-1223.4) | 0.61 (0.45 to 0.78) |
| Armenia | 4358.4 (3905.7-4846.7) | 895.4 (802.4-995.7) | 9070.6 (8201-10060.2) | 1153.1 (1042.6-1278.9) | 0.64 (0.39 to 0.88) |
| Australasia | 75146.3 (66883-85641.5) | 1907.5 (1697.7-2173.9) | 169744.5 (155559.5-183276.5) | 1921.4 (1760.9-2074.6) | 0.15 (0.05 to 0.25) |
| Australia | 59479.5 (52602.7-67972.1) | 1812.6 (1603-2071.3) | 140968 (128305.3-153511.9) | 1903 (1732-2072.3) | 0.29 (0.18 to 0.41) |
| Austria | 36814.8 (32415.4-41755.4) | 1891.9 (1665.8-2145.8) | 46739.9 (42678.1-50612.4) | 1581.6 (1444.2-1712.7) | -0.35 (-0.46 to -0.25) |
| Azerbaijan | 4105.8 (3548.9-4709.3) | 470.7 (406.9-539.9) | 10505.4 (8592.5-12741.6) | 548.4 (448.5-665.1) | 0.54 (0.41 to 0.67) |
| Bahamas | 259.5 (232.4-287.6) | 1056.8 (946.3-1171.4) | 1010.8 (871.4-1184.9) | 1404.6 (1210.9-1646.6) | 1.19 (1.02 to 1.37) |
| Bahrain | 234.8 (196.9-280.5) | 836.8 (701.7-999.6) | 2460 (1911.9-3199) | 1515 (1177.5-1970.1) | 1.42 (1.15 to 1.69) |
| Bangladesh | 6205.5 (5041.1-7724.7) | 81.9 (66.6-102) | 34603.9 (28852.5-41671.6) | 148.1 (123.5-178.3) | 1.7 (1.55 to 1.86) |
| Barbados | 539.3 (478.9-597.9) | 1150.2 (1021.4-1275.3) | 1524.7 (1258-1853) | 1673.3 (1380.6-2033.6) | 1.52 (1.32 to 1.72) |
| Belarus | 19270.3 (17014.8-22068.4) | 832.4 (735-953.3) | 31492.2 (26539-36831.9) | 1094.8 (922.6-1280.4) | 0.48 (0.33 to 0.63) |
| Belgium | 58185.6 (52211.3-65387.9) | 2219.2 (1991.3-2493.9) | 79933.7 (72644.1-85867.1) | 2114.2 (1921.4-2271.2) | -0.06 (-0.22 to 0.09) |
| Belize | 46.6 (41.2-52.8) | 303.1 (267.4-342.8) | 221.7 (195-250.1) | 442.6 (389.3-499.3) | 1.32 (1.22 to 1.42) |
| Benin | 645.3 (533.3-764.6) | 201.7 (166.7-239) | 2174.2 (1729-2743.1) | 262.9 (209.1-331.7) | 0.91 (0.84 to 0.98) |
| Bermuda | 170.7 (151.8-191.5) | 1613.5 (1435.4-1810.3) | 395.6 (335.9-493) | 1698.6 (1442.4-2116.9) | -0.24 (-0.41 to -0.08) |
| Bhutan | 51.1 (40.5-62.8) | 128.8 (102-158.3) | 199.7 (153.6-259.7) | 200.6 (154.2-260.7) | 1.58 (1.43 to 1.74) |
| Bolivia (Plurinational State of) | 1459 (1002.4-2049.1) | 278.3 (191.2-390.8) | 7118.2 (4820.8-10205.7) | 460.6 (311.9-660.4) | 1.71 (1.69 to 1.74) |
| Bosnia and Herzegovina | 4230.1 (3551.8-5001.9) | 576.7 (484.2-681.9) | 10979.4 (9345-12851) | 1002.4 (853.2-1173.3) | 2.69 (2.42 to 2.96) |
| Botswana | 288.3 (223.5-354.3) | 310.1 (240.4-381) | 1107.8 (882.8-1403.4) | 457 (364.2-578.9) | 1.6 (1.34 to 1.85) |
| Brazil | 62142.4 (57405.3-67173) | 420.5 (388.4-454.5) | 278173.1 (257171.6-296275.5) | 642.3 (593.8-684.1) | 1.26 (1.19 to 1.32) |
| Brunei Darussalam | 93.9 (75.2-115.6) | 599.8 (480.1-738.6) | 477.7 (398.3-564.1) | 797.8 (665.3-942.1) | 1.45 (1.27 to 1.64) |
| Bulgaria | 21392.7 (18640.3-24602.1) | 945.2 (823.6-1087) | 35382.4 (30424.4-40587.7) | 1488.6 (1280-1707.6) | 1.68 (1.58 to 1.78) |
| Burkina Faso | 2004.4 (1620.1-2432.9) | 277.1 (224-336.4) | 5576.1 (4448.3-6889.3) | 370.9 (295.9-458.2) | 0.98 (0.92 to 1.05) |
| Burundi | 1003.3 (777.8-1305.2) | 266.6 (206.7-346.8) | 1920.6 (1560.4-2370.7) | 239.9 (194.9-296.1) | -0.63 (-0.74 to -0.51) |
| Cabo Verde | 121.3 (101.7-144.5) | 307.4 (257.7-366.1) | 343.2 (274.4-426.9) | 447.7 (358.1-556.9) | 1.31 (1.18 to 1.44) |
| Cambodia | 1933.5 (1426.6-2691) | 261 (192.5-363.2) | 10428 (8165.2-13407.7) | 479.7 (375.6-616.8) | 2.27 (2.14 to 2.39) |
| Cameroon | 1858.2 (1507.9-2266.1) | 251 (203.6-306) | 7020.6 (5347.7-9178.6) | 344.9 (262.7-450.9) | 0.9 (0.75 to 1.06) |
| Canada | 121784.1 (109441.5-134789.7) | 2230.9 (2004.8-2469.1) | 226258.3 (207165.7-246456.4) | 1843.8 (1688.2-2008.4) | -0.73 (-0.87 to -0.59) |
| Caribbean | 27716.4 (25546.8-30355.1) | 643.1 (592.8-704.3) | 81566.7 (72321.8-90911) | 881 (781.1-981.9) | 1.19 (1.09 to 1.29) |
| Central African Republic | 456.2 (355.1-582.7) | 241.6 (188.1-308.6) | 993.9 (760.6-1276.9) | 277.9 (212.6-357) | 0.44 (0.39 to 0.5) |
| Central Asia | 46771 (42191.6-52218.4) | 584.8 (527.5-652.9) | 81256.6 (74953.1-88232.6) | 558.5 (515.1-606.4) | 0 (-0.05 to 0.05) |
| Central Europe | 229389.1 (205147.3-260968.5) | 865 (773.5-984) | 466448.5 (437280.5-495177) | 1259.7 (1180.9-1337.3) | 1.4 (1.3 to 1.5) |
| Central Latin America | 55100.2 (51087.3-59590.1) | 406 (376.5-439.1) | 344151.6 (308969.2-380032.8) | 804.7 (722.5-888.6) | 2.2 (2.15 to 2.25) |
| Central Sub-Saharan Africa | 9074 (6916.5-11534.9) | 241.3 (183.9-306.8) | 32090 (25223.5-40391.5) | 355.6 (279.5-447.6) | 1.28 (1.1 to 1.46) |
| Chad | 762 (609.3-922.6) | 164.4 (131.5-199.1) | 1755.9 (1403.3-2186.2) | 189.1 (151.1-235.5) | 0.59 (0.48 to 0.7) |
| Chile | 10729.2 (9320.3-12392.7) | 638 (554.2-736.9) | 34246.3 (31102.7-37376.5) | 764.8 (694.6-834.7) | 1.04 (0.92 to 1.16) |
| China | 420183.9 (353357.2-498140.8) | 292.8 (246.2-347.1) | 2284590.6 (1915262.5-2708549.1) | 602.9 (505.4-714.7) | 2.75 (2.57 to 2.92) |
| Colombia | 14194.5 (12835-15661.3) | 493 (445.8-544) | 91899 (78076.9-106628.3) | 960.9 (816.4-1115) | 2.12 (1.99 to 2.26) |
| Comoros | 90.6 (71.8-114.3) | 279.3 (221.3-352.1) | 357.2 (284.1-442.5) | 441.9 (351.4-547.5) | 1.53 (1.49 to 1.57) |
| Congo | 660.9 (452.1-947.2) | 371.3 (254-532.1) | 2342.4 (1648-3333.6) | 539.1 (379.3-767.2) | 1.23 (1.11 to 1.35) |
| Cook Islands | 16.9 (13.7-20.9) | 793 (643-981.6) | 60.2 (49.8-72.9) | 1277.4 (1056.7-1547.7) | 1.52 (1.47 to 1.57) |
| Costa Rica | 1803 (1620.3-2002.7) | 634 (569.8-704.2) | 12112.4 (10454.3-13931.8) | 1265.1 (1091.9-1455.1) | 2.37 (2.24 to 2.5) |
| Croatia | 13130.6 (11316.8-14980.9) | 1179.6 (1016.6-1345.8) | 21929.9 (19390.6-24317.8) | 1470.5 (1300.2-1630.6) | 0.96 (0.81 to 1.12) |
| Cuba | 12890.8 (11719.7-14152.7) | 760.7 (691.6-835.1) | 36205.7 (31376.9-42048.9) | 1059.6 (918.2-1230.6) | 1.24 (1.1 to 1.39) |
| Cyprus | 2076.4 (1736.2-2517) | 1486.4 (1242.9-1801.9) | 7461.7 (6470.1-8524.1) | 2135 (1851.2-2438.9) | 1.71 (1.47 to 1.95) |
| Czechia | 28353.5 (24925.8-32199.9) | 1197.4 (1052.7-1359.9) | 46344.4 (40557.2-51804.6) | 1326.6 (1160.9-1482.9) | 0.2 (-0.02 to 0.42) |
| C么te d'Ivoire | 1737.8 (1412.4-2138.4) | 271.1 (220.4-333.6) | 7856.8 (6212.4-10183.8) | 438.2 (346.4-567.9) | 1.67 (1.62 to 1.72) |
| Democratic People's Republic of Korea | 7563.9 (5692.4-9798.3) | 284.2 (213.9-368.2) | 20242.7 (16225.3-24773.9) | 359.2 (287.9-439.6) | 1.21 (1.02 to 1.4) |
| Democratic Republic of the Congo | 6213.9 (4619.2-8012.8) | 235.3 (174.9-303.4) | 20214.3 (15132.5-26595.7) | 334.7 (250.5-440.3) | 1.14 (0.94 to 1.34) |
| Denmark | 26071.7 (23424.7-29574) | 1998.4 (1795.5-2266.8) | 35698.4 (33021.9-38717.4) | 1854.2 (1715.2-2011.1) | -0.07 (-0.27 to 0.13) |
| Djibouti | 62.2 (49.5-78) | 289.8 (230.7-363.6) | 402.6 (306.7-541.5) | 388.3 (295.8-522.2) | 0.89 (0.81 to 0.97) |
| Dominica | 90.3 (75.6-105.7) | 919.7 (770.1-1076.6) | 140.6 (110.3-175.2) | 932.2 (731.3-1161.3) | -0.04 (-0.14 to 0.07) |
| Dominican Republic | 1791.2 (1537.1-2097.6) | 293 (251.4-343.1) | 7407.9 (5898.6-9254.4) | 443.3 (353-553.8) | 1.67 (1.53 to 1.81) |
| East Asia | 436883.7 (368029.7-516892) | 293.3 (247.1-347) | 2375001.5 (2004621-2802934) | 605.7 (511.2-714.8) | 2.77 (2.6 to 2.94) |
| Eastern Europe | 412349.8 (368007.4-472049.9) | 843.4 (752.7-965.5) | 703775.7 (645364.9-763008.6) | 1133.7 (1039.6-1229.1) | 0.9 (0.85 to 0.94) |
| Eastern Sub-Saharan Africa | 30766.3 (26637.7-36181) | 252.9 (219-297.4) | 101761 (90028.3-114704.3) | 376.4 (333-424.2) | 1.29 (1.16 to 1.43) |
| Ecuador | 1780.6 (1586.2-1994.8) | 207.1 (184.5-232) | 12287.5 (10004.4-14976.2) | 444 (361.5-541.1) | 2.86 (2.68 to 3.03) |
| Egypt | 14209.6 (11821-17173.3) | 317.8 (264.4-384.1) | 93629.4 (79311.4-112340.6) | 846.1 (716.7-1015.2) | 4.05 (3.66 to 4.43) |
| El Salvador | 1263 (1102.9-1440.5) | 258.3 (225.6-294.6) | 7166.3 (5923.4-8419.3) | 700.8 (579.3-823.3) | 3.51 (3.34 to 3.68) |
| Equatorial Guinea | 80 (60.3-104) | 250.7 (188.9-325.9) | 499.4 (334.5-702.7) | 631.4 (422.9-888.4) | 3.52 (3.34 to 3.71) |
| Eritrea | 495 (379.1-643.9) | 280.1 (214.5-364.4) | 1905.2 (1477.1-2394.6) | 432.4 (335.2-543.5) | 1.43 (1.4 to 1.45) |
| Estonia | 3746.6 (3304.6-4198.8) | 1045.2 (921.9-1171.3) | 5788 (4975.4-6577.7) | 1323.1 (1137.3-1503.6) | 0.89 (0.8 to 0.98) |
| Eswatini | 152 (122.3-186.7) | 336.6 (270.9-413.5) | 506 (346.6-724.2) | 558.9 (382.9-799.9) | 1.94 (1.85 to 2.03) |
| Ethiopia | 7645.5 (5814.3-10065.4) | 238 (181-313.3) | 23238.3 (19919.6-27667) | 339.5 (291-404.2) | 1.12 (0.92 to 1.32) |
| Fiji | 333.4 (271.1-408.4) | 591.4 (481-724.5) | 1021.5 (812.3-1253.8) | 747.1 (594-916.9) | 0.53 (0.41 to 0.65) |
| Finland | 21140.9 (18540-24283) | 1780.5 (1561.5-2045.1) | 41860.4 (37993.8-45470.8) | 2079.8 (1887.7-2259.2) | 0.9 (0.76 to 1.04) |
| France | 271245.4 (238718.9-307361.6) | 1949.1 (1715.4-2208.6) | 531357.4 (480901.3-574550.5) | 2403.2 (2175-2598.6) | 1.01 (0.91 to 1.11) |
| Gabon | 385.3 (294.1-486.6) | 393.6 (300.4-497.1) | 1050.2 (782.4-1394.6) | 587.5 (437.7-780.2) | 1.09 (0.99 to 1.18) |
| Gambia | 71.1 (57.8-85.6) | 128.5 (104.4-154.7) | 312.4 (245.7-389.9) | 202 (158.9-252.1) | 1.64 (1.44 to 1.85) |
| Georgia | 10027.1 (8928.3-11167.7) | 902.7 (803.8-1005.4) | 13448.1 (12008.9-15036) | 1280.9 (1143.9-1432.2) | 1.18 (1 to 1.37) |
| Germany | 411671.3 (366552.4-471175.3) | 1961.9 (1746.9-2245.5) | 654393.5 (595056.9-704022.8) | 2076.5 (1888.2-2234) | 0.63 (0.48 to 0.79) |
| Ghana | 2925.6 (2358.7-3553) | 286.5 (231-347.9) | 12302.8 (9718.4-14940.1) | 451.1 (356.4-547.8) | 1.38 (1.32 to 1.44) |
| Global | 5675458.9 (5129012.5-6360073.4) | 845.3 (763.9-947.2) | 13445598.7 (12595043.1-14206323.6) | 904.8 (847.6-956) | 0.32 (0.29 to 0.34) |
| Greece | 48111.1 (42501.3-54798.2) | 1799.4 (1589.6-2049.6) | 73382.5 (67532-78756.2) | 1996.6 (1837.4-2142.8) | 0.31 (0.21 to 0.42) |
| Greenland | 53.7 (44.7-63.6) | 974.2 (812-1154.4) | 100.5 (82.2-120.2) | 740 (605.8-885.5) | -0.91 (-1.06 to -0.75) |
| Grenada | 89.2 (79.5-99.7) | 740 (659.2-826.7) | 223.9 (197.1-254.4) | 1114.9 (981.5-1266.7) | 1.33 (1.09 to 1.57) |
| Guam | 61.1 (49.7-74.5) | 473.9 (385.5-578) | 174.4 (149.4-204.7) | 462.8 (396.4-543.2) | 0.36 (0.16 to 0.57) |
| Guatemala | 882.2 (775-1022.9) | 153.4 (134.8-177.9) | 5992 (5192.1-7009.7) | 327.3 (283.6-382.9) | 2.96 (2.73 to 3.19) |
| Guinea | 1145.5 (915.1-1391.6) | 207 (165.4-251.5) | 2668.9 (2099-3404.8) | 292.1 (229.8-372.7) | 1.16 (1.12 to 1.2) |
| Guinea-Bissau | 139.4 (108.5-184.8) | 218.4 (170-289.6) | 380.7 (287.9-496.2) | 331.4 (250.6-432) | 1.37 (1.35 to 1.39) |
| Guyana | 274.8 (238.9-313.8) | 448.2 (389.8-511.9) | 733.9 (581.3-907.4) | 651.6 (516.2-805.8) | 1.45 (1.27 to 1.62) |
| Haiti | 1852.8 (1269-2670.3) | 343.5 (235.3-495.1) | 5356.4 (3578.2-8016.8) | 452 (301.9-676.5) | 1.11 (0.99 to 1.22) |
| High SDI | 3484795 (3160807.4-3873044.9) | 1868.9 (1695.1-2077.1) | 6289444.3 (5860424.6-6657145.4) | 1823 (1698.6-1929.5) | -0.02 (-0.07 to 0.03) |
| High-income Asia Pacific | 247802.8 (211030.3-293823.7) | 708.7 (603.5-840.3) | 853049.4 (761902.9-923076.6) | 1209.9 (1080.7-1309.3) | 2.25 (2.1 to 2.41) |
| High-income North America | 1655322.5 (1516937.1-1824341.4) | 2857.5 (2618.7-3149.3) | 2626218.7 (2441784.3-2790350.6) | 2333.7 (2169.8-2479.6) | -0.92 (-1.02 to -0.82) |
| High-middle SDI | 1385645.5 (1236596.9-1586909.7) | 803.2 (716.8-919.8) | 3419335.7 (3157078.2-3694476.9) | 986.3 (910.7-1065.7) | 0.78 (0.75 to 0.82) |
| Honduras | 714.6 (511-945) | 214.4 (153.3-283.5) | 4847.6 (3646.3-6664.5) | 460.9 (346.7-633.7) | 2.76 (2.67 to 2.84) |
| Hungary | 28260 (24749.1-32682) | 1097.2 (960.9-1268.9) | 45035.8 (40121.1-50473.6) | 1416.2 (1261.6-1587.2) | 0.75 (0.59 to 0.91) |
| Iceland | 952.4 (840.9-1067.5) | 1996 (1762.3-2237.3) | 1833.5 (1658.4-2012.3) | 1882.8 (1702.9-2066.3) | -0.22 (-0.4 to -0.05) |
| India | 114685.6 (98818.7-133693.6) | 149.6 (128.9-174.4) | 608188.7 (526889.9-708522.7) | 302.5 (262.1-352.4) | 2.33 (2.16 to 2.51) |
| Indonesia | 43964.1 (32968-58077.6) | 272.1 (204.1-359.5) | 189029.4 (138960.1-256590.6) | 451.4 (331.8-612.7) | 1.62 (1.58 to 1.65) |
| Iran (Islamic Republic of) | 14723.3 (12516.9-17567.3) | 319.4 (271.5-381.1) | 100337.7 (89892.5-111727.6) | 772.4 (692-860) | 3.45 (3.14 to 3.76) |
| Iraq | 5337.1 (4309.4-6616) | 422.8 (341.4-524.1) | 36051.7 (27261.1-46173.4) | 927.9 (701.7-1188.4) | 2.88 (2.62 to 3.13) |
| Ireland | 13100.8 (11621.7-14902.3) | 1924.5 (1707.2-2189.2) | 25512.5 (23138.8-27756) | 1944 (1763.1-2115) | 0.31 (0.19 to 0.43) |
| Israel | 13208.9 (11546.9-15293.5) | 1629.6 (1424.6-1886.8) | 33389.1 (30205.8-36781.2) | 1670.7 (1511.4-1840.4) | 0.05 (-0.15 to 0.24) |
| Italy | 302878.6 (267473.9-345674.7) | 1990.1 (1757.5-2271.3) | 467429.9 (420459.5-507130.2) | 2057.6 (1850.9-2232.4) | 0.15 (0.02 to 0.28) |
| Jamaica | 2150.5 (1932.1-2345.2) | 730.2 (656-796.3) | 5824.8 (4672.7-7136.3) | 1101.3 (883.5-1349.3) | 1.35 (1.16 to 1.53) |
| Japan | 225057.9 (192386.6-265245) | 760 (649.7-895.7) | 723514.9 (642995.9-791133.7) | 1386 (1231.8-1515.6) | 2.44 (2.29 to 2.59) |
| Jordan | 1186.3 (957.8-1470.8) | 551.8 (445.5-684) | 13720.4 (10451.5-17546.5) | 1091.3 (831.3-1395.6) | 2.92 (2.53 to 3.31) |
| Kazakhstan | 14503.1 (12617.5-16576.6) | 693.5 (603.3-792.6) | 20280.5 (17596.9-22881.2) | 639.1 (554.5-721) | 0.21 (0.05 to 0.36) |
| Kenya | 3170.8 (2496.5-3923.9) | 237.9 (187.3-294.4) | 15094.9 (11672.5-19501) | 403.5 (312-521.3) | 1.81 (1.6 to 2.02) |
| Kiribati | 23.8 (19.2-29.2) | 400.2 (322.9-490.5) | 62.3 (50.5-78.5) | 495.5 (401.4-624.5) | 0.91 (0.85 to 0.98) |
| Kuwait | 523 (457-592.5) | 572 (499.8-648) | 4272 (3719.7-4877.8) | 916.3 (797.8-1046.2) | 2.03 (1.53 to 2.53) |
| Kyrgyzstan | 2715.4 (2396.1-3079.1) | 527.3 (465.3-598) | 3990.4 (3504.7-4530.1) | 471.8 (414.4-535.6) | -0.57 (-0.72 to -0.42) |
| Lao People's Democratic Republic | 761 (536.6-1101.8) | 221.9 (156.5-321.3) | 2736.5 (2087.1-3653.2) | 353.9 (269.9-472.5) | 1.77 (1.66 to 1.88) |
| Latvia | 6131.8 (5378.8-6980) | 980.7 (860.3-1116.4) | 8551.8 (7497.7-9694.4) | 1299.6 (1139.4-1473.3) | 1.06 (0.94 to 1.18) |
| Lebanon | 2982.8 (2383.4-3772.2) | 786.7 (628.7-994.9) | 18813.4 (15820.9-22295.6) | 1919.7 (1614.3-2275) | 3.45 (3.21 to 3.69) |
| Lesotho | 423.3 (343.3-520.3) | 302.7 (245.5-372.1) | 890.1 (636.5-1195.5) | 493 (352.5-662.1) | 2.06 (1.87 to 2.25) |
| Liberia | 348.6 (289.8-423.7) | 185 (153.8-224.9) | 1000.7 (764.7-1298.9) | 303.1 (231.6-393.4) | 1.89 (1.73 to 2.05) |
| Libya | 1185.6 (998.6-1400.2) | 381.2 (321.1-450.2) | 6522 (5389.5-7997.3) | 779.8 (644.4-956.3) | 2.96 (2.7 to 3.22) |
| Lithuania | 7476.5 (6610.4-8454) | 943.5 (834.2-1066.8) | 11569.2 (10182-13026.5) | 1195.4 (1052.1-1346) | 0.8 (0.66 to 0.95) |
| Low SDI | 74703.7 (64736.4-86462.1) | 200.2 (173.5-231.8) | 242470.7 (219356.7-264719.1) | 295.5 (267.3-322.6) | 1.3 (1.13 to 1.47) |
| Low-middle SDI | 195595.2 (172270-224865.7) | 194 (170.9-223.1) | 877396.6 (808321.3-947288.6) | 363.9 (335.3-392.9) | 2.19 (2.07 to 2.31) |
| Luxembourg | 1810 (1612.3-2060.4) | 1938.1 (1726.5-2206.3) | 3100.1 (2813.5-3374.2) | 1744.5 (1583.3-1898.8) | 0.07 (-0.14 to 0.29) |
| Madagascar | 2147.3 (1782.7-2556.8) | 254.2 (211-302.6) | 5448.1 (4169.8-6900.6) | 295 (225.8-373.7) | 0.34 (0.2 to 0.48) |
| Malawi | 1436.5 (1169.4-1709.9) | 228.4 (185.9-271.9) | 4121.8 (3256.4-5174.8) | 352.6 (278.5-442.6) | 1.46 (1.33 to 1.58) |
| Malaysia | 6336.5 (5402.3-7331) | 429.3 (366-496.7) | 40888.6 (35927-46341.1) | 834 (732.8-945.2) | 2.17 (2.1 to 2.24) |
| Maldives | 25.9 (17.6-36.8) | 168.8 (114.9-239.8) | 163.4 (137.6-193.2) | 300.5 (252.9-355.2) | 2.01 (1.74 to 2.27) |
| Mali | 1547.6 (1278.5-1830.5) | 231.5 (191.2-273.8) | 4545.5 (3504.7-5835.9) | 314.1 (242.1-403.2) | 0.97 (0.91 to 1.03) |
| Malta | 1332.5 (1193.8-1515.9) | 1839.3 (1647.8-2092.5) | 2844 (2581.4-3117.8) | 1802.3 (1635.9-1975.8) | -0.21 (-0.37 to -0.06) |
| Marshall Islands | 9.9 (7.6-12.9) | 387.1 (296.8-503.9) | 28.6 (20.5-40.1) | 487.4 (350.2-684.7) | 0.78 (0.64 to 0.91) |
| Mauritania | 436.8 (337.9-559.9) | 263.1 (203.6-337.3) | 1458.2 (1151.8-1864) | 406.5 (321.1-519.6) | 1.3 (1.12 to 1.47) |
| Mauritius | 428.3 (378.4-491.2) | 352.2 (311.1-403.8) | 2645.9 (2428.4-2850.7) | 782 (717.7-842.6) | 2.61 (2.36 to 2.87) |
| Mexico | 26839.1 (24784-29250.9) | 386 (356.5-420.7) | 155670.5 (134691.3-177617.6) | 722.4 (625-824.2) | 1.93 (1.87 to 1.99) |
| Micronesia (Federated States of) | 35.3 (27.4-45.8) | 443 (344.3-575.1) | 80 (63.4-102) | 607.9 (481.7-775) | 1.04 (1.01 to 1.07) |
| Middle SDI | 528165.7 (465766.8-603254.8) | 304.3 (268.4-347.6) | 2603046.8 (2380356.1-2837406.1) | 554 (506.6-603.9) | 2.09 (2 to 2.19) |
| Monaco | 284.5 (230-349.6) | 2567.7 (2075.7-3154.3) | 515.1 (429.6-618.8) | 3292.1 (2746-3955.3) | 1.1 (1.01 to 1.18) |
| Mongolia | 302.1 (247.9-372.2) | 174.3 (143-214.8) | 702.9 (590.7-840) | 178 (149.6-212.8) | 0.45 (0.15 to 0.75) |
| Montenegro | 1315.1 (1068.9-1585) | 1207.2 (981.2-1454.9) | 2922 (2438-3461.1) | 1675.7 (1398.2-1984.9) | 1.35 (1.27 to 1.43) |
| Morocco | 5161.4 (4259.2-6230) | 220.1 (181.6-265.7) | 24164.5 (18589.2-31309.5) | 402.2 (309.4-521.1) | 2.41 (2.06 to 2.76) |
| Mozambique | 2222.8 (1871-2682.5) | 227.5 (191.5-274.5) | 6059 (4631-7508.1) | 338.7 (258.8-419.7) | 1.56 (1.4 to 1.72) |
| Myanmar | 10445.1 (7925.6-13317.6) | 266.8 (202.5-340.2) | 36447.7 (30493.7-44709.6) | 431.7 (361.2-529.6) | 1.64 (1.6 to 1.68) |
| Namibia | 389.4 (323.4-474.5) | 358.9 (298-437.3) | 1814.7 (1296.5-2374.7) | 803.2 (573.8-1051) | 3.14 (2.94 to 3.35) |
| Nauru | 3.3 (2.4-4.6) | 461.9 (325.2-641.2) | 7.2 (5.2-9.9) | 775.8 (558.9-1066.9) | 1.7 (1.64 to 1.75) |
| Nepal | 1890.5 (1502.7-2327.9) | 122.8 (97.6-151.2) | 7742.8 (5995.1-10137) | 195.6 (151.5-256.1) | 1.63 (1.38 to 1.88) |
| Netherlands | 67916.3 (60887-76849.5) | 2051.3 (1838.9-2321.1) | 121212.3 (110312.4-131766.3) | 2071.7 (1885.4-2252.1) | 0.21 (0.11 to 0.32) |
| New Zealand | 15666.8 (13912.7-17885.7) | 2381.1 (2114.5-2718.3) | 28776.5 (25754.5-31431.9) | 2017.4 (1805.5-2203.6) | -0.48 (-0.52 to -0.44) |
| Nicaragua | 557.8 (475-644.9) | 224.5 (191.2-259.5) | 4137.9 (3392.2-5127.9) | 508.3 (416.7-629.9) | 3.27 (3.07 to 3.46) |
| Niger | 594.5 (476.1-739.4) | 135.3 (108.3-168.2) | 2307.6 (1797.9-2934.1) | 165.7 (129.1-210.8) | 0.87 (0.77 to 0.97) |
| Nigeria | 21136.3 (16969.4-25293.6) | 290.1 (232.9-347.2) | 89403.5 (66867.1-119646.3) | 596 (445.7-797.6) | 2.6 (2.25 to 2.95) |
| Niue | 2.4 (2-2.8) | 653.3 (543.9-781.2) | 3.1 (2.5-3.8) | 798.7 (645.1-978.4) | 0.78 (0.69 to 0.87) |
| North Africa and Middle East | 86138.3 (74198.5-101215.1) | 304.8 (262.5-358.1) | 573480.3 (524016.6-622895.1) | 752.3 (687.4-817.1) | 3.65 (3.33 to 3.98) |
| North Macedonia | 2748.9 (2355.4-3242.6) | 835.6 (716-985.7) | 7301.1 (6155.6-8732.1) | 1231.4 (1038.2-1472.8) | 1.76 (1.57 to 1.96) |
| Northern Mariana Islands | 13.6 (11-17) | 557.3 (451.7-696.4) | 80.7 (69.5-92.7) | 830.4 (715.5-954.1) | 0.73 (0.54 to 0.93) |
| Norway | 17856.4 (15708.4-20529.1) | 1648.9 (1450.6-1895.7) | 24711.3 (22662.6-26801) | 1523.8 (1397.4-1652.6) | -0.01 (-0.2 to 0.17) |
| Oceania | 1679.2 (1414.7-2006.3) | 349 (294.1-417) | 4832.5 (4198.1-5581.2) | 391.6 (340.2-452.2) | 0.27 (0.21 to 0.32) |
| Oman | 226.5 (185.4-279.6) | 225.3 (184.4-278.2) | 1120.1 (897-1384.9) | 358.6 (287.2-443.4) | 2.15 (1.94 to 2.36) |
| Pakistan | 26363.1 (21326.5-31238.8) | 289.1 (233.9-342.6) | 99514.6 (78626.6-122818) | 501.9 (396.6-619.5) | 1.62 (1.5 to 1.73) |
| Palau | 14 (11.5-17.4) | 872.9 (717.1-1085.3) | 38.2 (30.8-47.3) | 912.7 (736.3-1129.4) | 0.02 (-0.04 to 0.08) |
| Palestine | 1001.5 (806.8-1266.7) | 690 (555.8-872.7) | 5503.9 (4659.3-6495.9) | 1279.2 (1082.9-1509.8) | 2.28 (2.15 to 2.41) |
| Panama | 1213.1 (1103.3-1350.4) | 500.7 (455.4-557.3) | 7362.1 (6064.5-8758.5) | 990.1 (815.6-1177.9) | 2.31 (2.19 to 2.42) |
| Papua New Guinea | 832.6 (650.7-1081.8) | 281.4 (219.9-365.6) | 2455.7 (1956.2-3075.2) | 299 (238.2-374.4) | 0.03 (-0.06 to 0.11) |
| Paraguay | 1231.9 (1039.7-1461.2) | 339.6 (286.6-402.8) | 6354.4 (4945.4-8153.3) | 641.8 (499.5-823.5) | 2.23 (2.13 to 2.32) |
| Peru | 5336.5 (4356.5-6320.8) | 270.7 (221-320.6) | 27827.3 (20881.9-36310.5) | 497.5 (373.3-649.2) | 1.87 (1.75 to 2) |
| Philippines | 17558.2 (15350.8-20232) | 367.8 (321.5-423.8) | 77550 (66399.6-91235.9) | 556.1 (476.2-654.3) | 1.32 (1.24 to 1.4) |
| Poland | 59773.7 (52620.5-68206.2) | 775.7 (682.9-885.1) | 148274.1 (134189.1-161593.7) | 1223.9 (1107.6-1333.8) | 1.75 (1.64 to 1.86) |
| Portugal | 33739 (29207.3-39359.5) | 1383.9 (1198-1614.4) | 63483.3 (57922-68498.2) | 1631.7 (1488.8-1760.6) | 0.84 (0.73 to 0.96) |
| Puerto Rico | 4952.5 (4479.3-5470) | 816.2 (738.2-901.5) | 14517 (12393.8-16847.8) | 1243.6 (1061.7-1443.2) | 1.49 (1.33 to 1.65) |
| Qatar | 101.6 (80.9-125.2) | 621.8 (495.3-766.3) | 2131.7 (1631.4-2738.3) | 1391.6 (1065-1787.6) | 2.31 (2.12 to 2.5) |
| Republic of Korea | 19640.4 (15992.5-24712.6) | 394.6 (321.3-496.6) | 110614.7 (95017.2-125087.6) | 661.4 (568.2-748) | 2.47 (2.1 to 2.84) |
| Republic of Moldova | 6085.9 (5318.5-7003.4) | 788.3 (688.9-907.2) | 11017 (9599.9-12634.7) | 1043.4 (909.2-1196.7) | 1.22 (1.01 to 1.42) |
| Romania | 31731.6 (27538.1-37030.6) | 634.1 (550.3-740) | 64490.5 (58159.6-70925.7) | 1073.9 (968.5-1181) | 2.05 (1.94 to 2.15) |
| Russian Federation | 256633.5 (227287-294237.2) | 813.1 (720.1-932.2) | 522709.9 (477206-568997) | 1229.8 (1122.7-1338.7) | 1.35 (1.28 to 1.41) |
| Rwanda | 1528.3 (1183.7-1993.2) | 326.6 (253-425.9) | 4686 (3607.2-6092.6) | 443.1 (341.1-576.1) | 0.81 (0.6 to 1.03) |
| Saint Kitts and Nevis | 61.2 (54.7-68) | 957.3 (856-1063.5) | 139.5 (117-163.7) | 1083.3 (908.5-1270.7) | 0.84 (0.64 to 1.05) |
| Saint Lucia | 112.3 (102.2-123) | 767.5 (698.6-840.4) | 340.5 (288.3-399.9) | 809.1 (685.1-950.3) | -0.3 (-0.5 to -0.1) |
| Saint Vincent and the Grenadines | 95.4 (85.4-106.3) | 794.2 (710.9-884.4) | 223.4 (197.6-253.5) | 878.5 (777.2-996.8) | 0.13 (-0.08 to 0.35) |
| Samoa | 56.8 (46.7-68.8) | 393.8 (324.2-477.2) | 122.7 (101-152.7) | 498.3 (410.3-620.2) | 0.81 (0.77 to 0.85) |
| San Marino | 97.2 (80.3-116.7) | 1654.2 (1367.4-1986.8) | 155.1 (123.8-196.6) | 1311.7 (1046.8-1661.9) | -0.15 (-0.33 to 0.03) |
| Sao Tome and Principe | 25.2 (21.1-30.9) | 225.1 (188.1-276.1) | 74 (57.8-94.3) | 404.9 (316.1-515.5) | 2.1 (2 to 2.19) |
| Saudi Arabia | 2502.1 (2068.2-3061.7) | 280.8 (232.1-343.7) | 17918.3 (14937.7-21958.1) | 595.2 (496.2-729.3) | 2.87 (2.75 to 2.99) |
| Senegal | 1069.8 (869.8-1319.4) | 202.4 (164.6-249.7) | 4357.6 (3505.4-5467.2) | 337.5 (271.5-423.4) | 1.8 (1.67 to 1.93) |
| Serbia | 18728.5 (14971.5-22868.7) | 900.5 (719.9-1099.6) | 40920.9 (33807.6-49474.7) | 1458.7 (1205.2-1763.7) | 1.7 (1.58 to 1.81) |
| Seychelles | 39.1 (33.5-46.2) | 407.9 (348.8-481.3) | 145.3 (127.7-162.9) | 700.6 (615.4-785.4) | 1.8 (1.51 to 2.09) |
| Sierra Leone | 587.8 (470.2-726.5) | 177.3 (141.8-219.2) | 1564.5 (1243-1948.5) | 259 (205.8-322.5) | 1.46 (1.35 to 1.58) |
| Singapore | 3010.6 (2623.9-3498) | 830.4 (723.7-964.8) | 18442.1 (16826.4-20030.3) | 1214.2 (1107.9-1318.8) | 1.75 (1.59 to 1.9) |
| Slovakia | 9669 (8284.8-11244.2) | 932 (798.6-1083.8) | 21930.5 (17852.7-26395.7) | 1337 (1088.4-1609.2) | 1.3 (1.2 to 1.41) |
| Slovenia | 5036 (4416.6-5760.5) | 1169.2 (1025.4-1337.4) | 9973.9 (8803.5-11403.7) | 1362.2 (1202.4-1557.5) | 0.63 (0.42 to 0.83) |
| Solomon Islands | 51.1 (39.3-68.8) | 226.2 (173.8-304.3) | 176.8 (135.4-228.8) | 326 (249.6-421.9) | 1.37 (1.13 to 1.61) |
| Somalia | 773.1 (594.1-978.3) | 214.7 (165-271.7) | 2286.1 (1747.2-2875.1) | 242.4 (185.3-304.9) | 0.44 (0.37 to 0.5) |
| South Africa | 12995.8 (10110.9-15671.2) | 385 (299.5-464.3) | 49870.6 (46124.2-54313.4) | 632.2 (584.7-688.5) | 1.79 (1.68 to 1.89) |
| South Asia | 149195.6 (130051.6-171074.7) | 157.1 (137-180.2) | 750249.8 (664032.3-849629.6) | 302.2 (267.4-342.2) | 2.1 (1.96 to 2.24) |
| South Sudan | 850.3 (669.7-1090.3) | 205.1 (161.5-262.9) | 1672.5 (1261.7-2220.7) | 268.6 (202.6-356.6) | 0.79 (0.65 to 0.93) |
| Southeast Asia | 119806.2 (101421.3-143647) | 283 (239.5-339.3) | 592125.3 (518301.8-682084.5) | 516.9 (452.4-595.4) | 2.1 (2.06 to 2.14) |
| Southern Latin America | 74825.4 (66745.2-84022.3) | 944.6 (842.6-1060.7) | 154792.1 (143796.6-165633.3) | 1051.9 (977.1-1125.5) | 0.53 (0.4 to 0.67) |
| Southern Sub-Saharan Africa | 16383.2 (13213.5-19263.9) | 370.3 (298.6-435.4) | 60726.5 (56182-65657.8) | 623.8 (577.1-674.4) | 1.95 (1.86 to 2.04) |
| Spain | 144045.5 (126171-167114.7) | 1506.7 (1319.7-1748) | 236737.4 (215189.7-257383.2) | 1529.8 (1390.6-1663.2) | 0.22 (0.14 to 0.3) |
| Sri Lanka | 4555.2 (3863.1-5381) | 255.9 (217.1-302.3) | 25439.3 (19076.9-32385.9) | 528.2 (396.1-672.4) | 2.83 (2.67 to 2.99) |
| Sudan | 2664.2 (2095.4-3417.7) | 179.2 (140.9-229.8) | 8481.8 (6413.3-11419.6) | 272.4 (206-366.7) | 1.69 (1.27 to 2.11) |
| Suriname | 181.2 (155.1-208.3) | 416.3 (356.5-478.7) | 592.8 (478.7-740.7) | 530.7 (428.6-663.1) | 1.21 (1.06 to 1.37) |
| Sweden | 44847.3 (39642.5-51315.4) | 1889 (1669.8-2161.5) | 60375.7 (53120.4-66958.1) | 1793.4 (1577.9-1989) | 0.2 (0 to 0.4) |
| Switzerland | 31513.2 (27808.7-35997.5) | 1865.6 (1646.3-2131.1) | 52067.7 (47169.7-56617.4) | 1776.5 (1609.4-1931.8) | 0.1 (-0.18 to 0.37) |
| Syrian Arab Republic | 3179.8 (2543.9-3931.7) | 360.7 (288.5-445.9) | 16368.8 (12754.9-20633) | 692.8 (539.8-873.3) | 2.44 (2.26 to 2.62) |
| Taiwan (Province of China) | 9135.9 (7961.9-10745.8) | 329.4 (287.1-387.4) | 70168.2 (64165.8-76372.6) | 931.9 (852.1-1014.3) | 3.82 (3.51 to 4.14) |
| Tajikistan | 1723.8 (1451.8-2039.5) | 368.1 (310-435.5) | 2907.4 (2253.2-3772.9) | 283.1 (219.4-367.3) | -1.07 (-1.16 to -0.97) |
| Thailand | 17166.7 (13950.4-20572.2) | 289.3 (235.1-346.7) | 132157.1 (109520.9-158011.1) | 679.9 (563.4-812.9) | 3.16 (2.94 to 3.39) |
| Timor-Leste | 77.9 (56.9-108.6) | 179.3 (130.9-249.9) | 382.3 (284.4-508.4) | 269.2 (200.2-358) | 1.76 (1.54 to 1.98) |
| Togo | 447.2 (368.5-537.3) | 229.2 (188.9-275.4) | 2223.7 (1726.1-2865.9) | 356 (276.3-458.8) | 1.46 (1.36 to 1.56) |
| Tokelau | 1.2 (0.9-1.5) | 492.2 (378.3-631.6) | 1.8 (1.4-2.2) | 692.4 (548.4-869.7) | 1.24 (1.16 to 1.31) |
| Tonga | 65.6 (53.2-78.8) | 691 (560.3-830.3) | 124.9 (102.1-153) | 930.2 (760.5-1139.3) | 0.79 (0.67 to 0.91) |
| Trinidad and Tobago | 1000.6 (905-1113.4) | 728.8 (659.1-811) | 3359.7 (2676.8-4130.2) | 964.5 (768.5-1185.7) | 1.14 (0.96 to 1.32) |
| Tropical Latin America | 63374.3 (58606.6-68496.8) | 418.6 (387.1-452.4) | 284527.5 (263666.1-302987.4) | 642.3 (595.2-684) | 1.28 (1.21 to 1.34) |
| Tunisia | 3662.8 (3027.5-4409.1) | 420.7 (347.7-506.4) | 19182.4 (14766.6-24929.2) | 820.8 (631.8-1066.7) | 2.35 (2.3 to 2.4) |
| Turkey | 16359.8 (13375-19451.2) | 273.8 (223.9-325.6) | 159984.8 (131696.4-193882.6) | 968.4 (797.1-1173.5) | 5.35 (4.61 to 6.1) |
| Turkmenistan | 1222.4 (1060-1427) | 376.4 (326.4-439.4) | 2864.5 (2390.6-3535.8) | 404.8 (337.8-499.6) | 0.38 (0 to 0.77) |
| Tuvalu | 4.8 (3.7-6.4) | 417 (314.9-551.5) | 10.3 (8.3-12.8) | 567.1 (453.1-704.4) | 1.1 (1.06 to 1.13) |
| Uganda | 3314.6 (2663.3-4163.7) | 316.9 (254.6-398.1) | 13126.1 (10295.6-16641.8) | 553.5 (434.1-701.7) | 1.73 (1.64 to 1.82) |
| Ukraine | 113005.1 (100023.9-130929.6) | 906.4 (802.3-1050.2) | 112647.6 (87376.7-143201.8) | 829.6 (643.5-1054.7) | -0.45 (-0.58 to -0.32) |
| United Arab Emirates | 410.7 (332-504.3) | 731.8 (591.5-898.6) | 4780 (3544.2-6336.4) | 665.7 (493.6-882.4) | 0.71 (0.2 to 1.22) |
| United Kingdom | 340946.2 (307085.3-382228.2) | 2295.5 (2067.5-2573.4) | 427931.6 (398615.1-453255.3) | 2034.8 (1895.4-2155.3) | -0.33 (-0.43 to -0.24) |
| United Republic of Tanzania | 4930.7 (4069.3-5954.4) | 273.3 (225.5-330) | 16080.4 (12641.1-19965.1) | 390.1 (306.7-484.3) | 1.2 (1.11 to 1.3) |
| United States Virgin Islands | 136.9 (115.3-162.9) | 980.3 (825.4-1166.3) | 313.5 (233.5-417.4) | 973.8 (725.3-1296.8) | 0 (-0.23 to 0.23) |
| United States of America | 1533446.7 (1401087.3-1690017.5) | 2923 (2670.7-3221.4) | 2399818.8 (2227392.1-2554692.3) | 2393.9 (2221.9-2548.4) | -0.92 (-1.03 to -0.82) |
| Uruguay | 8644.3 (7766.5-9648.1) | 1281.1 (1151-1429.8) | 14293.5 (13069.7-15503.5) | 1580.6 (1445.3-1714.4) | 0.61 (0.48 to 0.74) |
| Uzbekistan | 7812.9 (6741.4-9092.8) | 399.5 (344.7-464.9) | 17486.8 (14992-20460.4) | 376.2 (322.5-440.1) | -0.05 (-0.25 to 0.15) |
| Vanuatu | 25.2 (19.9-32.1) | 252.8 (199.2-322.5) | 93.9 (74.9-114.6) | 327.5 (261.3-399.5) | 0.92 (0.74 to 1.09) |
| Venezuela (Bolivarian Republic of) | 7633 (6921.9-8380.3) | 487.5 (442.1-535.2) | 54963.6 (43952.5-68219) | 1050.5 (840-1303.8) | 2.48 (2.35 to 2.61) |
| Viet Nam | 16341.3 (13562.6-20264.2) | 234.4 (194.5-290.6) | 73285.7 (59261.3-91056.9) | 419.5 (339.2-521.3) | 2.2 (2.1 to 2.31) |
| Western Europe | 1891552.7 (1692591.8-2137591.6) | 1947.8 (1742.9-2201.2) | 2995238.8 (2788352.5-3170207.2) | 2008.4 (1869.7-2125.7) | 0.32 (0.21 to 0.43) |
| Western Sub-Saharan Africa | 37605.7 (31821-43179.9) | 260.5 (220.4-299.1) | 147328.7 (122308-179145.9) | 458.4 (380.5-557.3) | 1.97 (1.74 to 2.19) |
| Yemen | 1387.9 (1082.2-1721) | 173.1 (134.9-214.6) | 5862.9 (4545.4-7471.1) | 260.7 (202.1-332.2) | 1.84 (1.36 to 2.33) |
| Zambia | 1073.2 (855.1-1366.5) | 233.1 (185.8-296.8) | 5273.5 (3640-7518.1) | 488.2 (337-696) | 2.56 (2.24 to 2.89) |
| Zimbabwe | 2134.5 (1749.3-2628.1) | 322 (263.9-396.5) | 6537.2 (5214.7-8105.6) | 590.4 (471-732) | 2.62 (2.31 to 2.94) |

**Table S6. Deaths of Breast Cancer Between 1990 and 2021 at the National Level**

| **location** | **Number 1990** | **ASR 1990**  **(95%UI)** | **Number 2021** | **ASR 2021**  **(95%UI)** | **EAPC**  **(95%CI)** |
| --- | --- | --- | --- | --- | --- |
| Afghanistan | 181.9 (112.2-284.6) | 15 (9.2-23.4) | 317.3 (195.9-494) | 25.8 (15.9-40.2) | 1.98 (1.84 to 2.12) |
| Albania | 65.7 (51.3-83.5) | 18.9 (14.8-24.1) | 181.7 (127.5-246.2) | 23.1 (16.2-31.3) | 1.06 (0.89 to 1.23) |
| Algeria | 252.4 (189.5-329.1) | 12 (9-15.7) | 882.6 (675.2-1131.7) | 14.5 (11.1-18.6) | 0.71 (0.63 to 0.79) |
| American Samoa | 1.8 (1.5-2.2) | 48.9 (40.4-59.4) | 6.2 (4.9-7.7) | 73.9 (58.4-91.8) | 1.64 (1.54 to 1.75) |
| Andean Latin America | 789.8 (665.8-939.6) | 23.5 (19.8-28) | 2733.8 (2139.4-3535.6) | 27.6 (21.6-35.7) | 0.33 (0.22 to 0.44) |
| Andorra | 4.7 (3.1-6.7) | 47.3 (31.2-68) | 10.9 (7.8-14.8) | 41.3 (29.6-55.8) | -0.02 (-0.25 to 0.22) |
| Angola | 150 (106.2-210.9) | 24.1 (17-33.9) | 711.2 (486.6-965.5) | 36.8 (25.2-49.9) | 1.4 (1.34 to 1.46) |
| Antigua and Barbuda | 5.8 (5.2-6.5) | 67.1 (59.8-75.5) | 13.3 (12.3-14.6) | 70.7 (65.1-77.3) | 0.09 (-0.13 to 0.32) |
| Argentina | 4082 (3774.4-4351.8) | 73.4 (67.8-78.2) | 5876.9 (5248.9-6498.8) | 63 (56.2-69.6) | -0.41 (-0.63 to -0.19) |
| Armenia | 280 (255.9-303.4) | 57.5 (52.6-62.3) | 433.3 (376.4-506.2) | 55.1 (47.9-64.4) | -0.24 (-0.58 to 0.1) |
| Australasia | 2462 (2261.2-2630.1) | 62.5 (57.4-66.8) | 3663.2 (3066.1-4198.4) | 41.5 (34.7-47.5) | -1.46 (-1.53 to -1.39) |
| Australia | 1960.4 (1789.4-2125.7) | 59.7 (54.5-64.8) | 3058.3 (2554.8-3527.3) | 41.3 (34.5-47.6) | -1.36 (-1.44 to -1.28) |
| Austria | 1538.3 (1400.4-1656.8) | 79.1 (72-85.1) | 1536.5 (1302.6-1718.8) | 52 (44.1-58.2) | -1.24 (-1.32 to -1.17) |
| Azerbaijan | 294.8 (248.5-334.6) | 33.8 (28.5-38.4) | 557 (424.3-710.8) | 29.1 (22.1-37.1) | -0.2 (-0.36 to -0.04) |
| Bahamas | 18 (16.4-19.8) | 73.2 (66.7-80.5) | 54.4 (45.4-65.1) | 75.7 (63.1-90.5) | 0.32 (0.16 to 0.49) |
| Bahrain | 11.8 (9.8-14.5) | 42.2 (34.9-51.8) | 58.6 (43.7-80.5) | 36.1 (26.9-49.5) | -1.32 (-1.69 to -0.95) |
| Bangladesh | 477.2 (372.6-625.9) | 6.3 (4.9-8.3) | 1861 (1390.7-2462.7) | 8 (6-10.5) | 0.3 (0.05 to 0.54) |
| Barbados | 37.2 (33.6-40.9) | 79.4 (71.8-87.3) | 79 (64.9-96.7) | 86.7 (71.2-106.2) | 0.44 (0.18 to 0.71) |
| Belarus | 856.4 (786.5-930.4) | 37 (34-40.2) | 1101.8 (888.7-1350.9) | 38.3 (30.9-47) | -0.77 (-1.08 to -0.46) |
| Belgium | 2409.8 (2160.4-2588.1) | 91.9 (82.4-98.7) | 2310.9 (1891-2622.4) | 61.1 (50-69.4) | -1.17 (-1.25 to -1.09) |
| Belize | 3.1 (2.8-3.4) | 20 (18-22.1) | 13 (11.3-14.7) | 26 (22.6-29.3) | 0.72 (0.36 to 1.08) |
| Benin | 76.2 (61.4-92) | 23.8 (19.2-28.8) | 234.2 (181.4-304.4) | 28.3 (21.9-36.8) | 0.57 (0.51 to 0.63) |
| Bermuda | 9.6 (8.6-10.5) | 90.8 (81.3-99.6) | 13 (10.5-16.4) | 55.8 (45.3-70.6) | -1.96 (-2.21 to -1.71) |
| Bhutan | 5.1 (3.5-6.9) | 12.8 (8.8-17.3) | 16.2 (11.9-21.5) | 16.3 (11.9-21.6) | 0.83 (0.73 to 0.93) |
| Bolivia (Plurinational State of) | 165.7 (106.2-244.8) | 31.6 (20.3-46.7) | 582.1 (375.2-857.3) | 37.7 (24.3-55.5) | 0.49 (0.44 to 0.54) |
| Bosnia and Herzegovina | 201.5 (172.5-229.8) | 27.5 (23.5-31.3) | 497.2 (398.8-609.1) | 45.4 (36.4-55.6) | 2.2 (1.97 to 2.44) |
| Botswana | 31.6 (22.2-41.9) | 34 (23.9-45.1) | 112.6 (87.4-148.5) | 46.5 (36.1-61.3) | 1.46 (1.14 to 1.78) |
| Brazil | 5020.3 (4723.3-5261.8) | 34 (32-35.6) | 16115.3 (14640.7-17277.7) | 37.2 (33.8-39.9) | 0.12 (0.04 to 0.2) |
| Brunei Darussalam | 4.5 (3.4-6.1) | 29 (21.9-38.7) | 19.2 (14.9-24.1) | 32.1 (24.9-40.3) | 0.86 (0.68 to 1.04) |
| Bulgaria | 915.7 (799.9-1051.3) | 40.5 (35.3-46.5) | 1476.1 (1214.3-1745.7) | 62.1 (51.1-73.4) | 1.57 (1.43 to 1.72) |
| Burkina Faso | 252.6 (197.4-320.4) | 34.9 (27.3-44.3) | 635.3 (475.9-822.8) | 42.3 (31.7-54.7) | 0.6 (0.49 to 0.7) |
| Burundi | 138.2 (100.9-188.9) | 36.7 (26.8-50.2) | 228.8 (174.4-305.8) | 28.6 (21.8-38.2) | -1.23 (-1.39 to -1.07) |
| Cabo Verde | 12.1 (9.8-14.9) | 30.8 (24.8-37.6) | 27.8 (20.7-36) | 36.2 (27-46.9) | 0.9 (0.76 to 1.04) |
| Cambodia | 202.1 (128.6-308.5) | 27.3 (17.4-41.6) | 909.2 (657.8-1213.6) | 41.8 (30.3-55.8) | 1.42 (1.36 to 1.48) |
| Cameroon | 217.3 (170.4-277.3) | 29.3 (23-37.5) | 730.2 (520.3-994.9) | 35.9 (25.6-48.9) | 0.57 (0.51 to 0.64) |
| Canada | 3802.1 (3469.6-4079.1) | 69.6 (63.6-74.7) | 5312.3 (4551.7-5936.4) | 43.3 (37.1-48.4) | -1.71 (-1.8 to -1.62) |
| Caribbean | 1710.5 (1577.2-1853) | 39.7 (36.6-43) | 4088.6 (3543.3-4653.8) | 44.2 (38.3-50.3) | 0.45 (0.36 to 0.53) |
| Central African Republic | 58.2 (42.4-76.9) | 30.8 (22.4-40.7) | 126.3 (91.1-169) | 35.3 (25.5-47.3) | 0.4 (0.35 to 0.45) |
| Central Asia | 3203.3 (2988-3396.9) | 40.1 (37.4-42.5) | 4388.8 (3940.6-4869.8) | 30.2 (27.1-33.5) | -0.48 (-0.64 to -0.33) |
| Central Europe | 12933.3 (12313.9-13514) | 48.8 (46.4-51) | 21824.7 (19760.6-23653.7) | 58.9 (53.4-63.9) | 0.42 (0.3 to 0.53) |
| Central Latin America | 3204.4 (3064.9-3310.8) | 23.6 (22.6-24.4) | 12870.4 (11327.4-14398.4) | 30.1 (26.5-33.7) | 0.68 (0.59 to 0.78) |
| Central Sub-Saharan Africa | 1053.9 (749.3-1393.5) | 28 (19.9-37.1) | 3418.7 (2529.4-4487.3) | 37.9 (28-49.7) | 0.98 (0.91 to 1.06) |
| Chad | 86.8 (63.3-116.7) | 18.7 (13.6-25.2) | 205.5 (153.4-267.6) | 22.1 (16.5-28.8) | 0.54 (0.48 to 0.6) |
| Chile | 666.1 (604.3-723.4) | 39.6 (35.9-43) | 1414.2 (1237.6-1569.4) | 31.6 (27.6-35.1) | -0.59 (-0.7 to -0.48) |
| China | 22195.5 (18370.6-26622.1) | 15.5 (12.8-18.5) | 61934.7 (49020.8-76670.1) | 16.3 (12.9-20.2) | -0.05 (-0.15 to 0.05) |
| Colombia | 841 (779.1-903) | 29.2 (27.1-31.4) | 2952.5 (2474-3490) | 30.9 (25.9-36.5) | 0.1 (-0.1 to 0.3) |
| Comoros | 10.8 (8-14.4) | 33.2 (24.7-44.4) | 39.6 (29.1-52.9) | 49 (36-65.5) | 1.21 (1.15 to 1.27) |
| Congo | 81.3 (48.9-125.4) | 45.7 (27.5-70.4) | 232 (148.7-345.5) | 53.4 (34.2-79.5) | 0.44 (0.36 to 0.52) |
| Cook Islands | 1.5 (1.2-1.9) | 70.1 (54-88.1) | 3.9 (3-4.9) | 83.7 (64.6-104.7) | 0.74 (0.6 to 0.88) |
| Costa Rica | 74.9 (68.2-81.4) | 26.3 (24-28.6) | 357.2 (306.5-410.5) | 37.3 (32-42.9) | 1.26 (1.1 to 1.43) |
| Croatia | 653.6 (572.4-748.5) | 58.7 (51.4-67.2) | 859.1 (727.6-1001.4) | 57.6 (48.8-67.1) | 0.22 (0 to 0.44) |
| Cuba | 679.2 (624.3-723.1) | 40.1 (36.8-42.7) | 1513.9 (1298.8-1752) | 44.3 (38-51.3) | 0.51 (0.35 to 0.68) |
| Cyprus | 76.8 (61.7-93.2) | 55 (44.2-66.7) | 177.8 (146.4-216) | 50.9 (41.9-61.8) | -0.08 (-0.26 to 0.11) |
| Czechia | 1589.1 (1431.4-1780) | 67.1 (60.5-75.2) | 1760.1 (1461-2065) | 50.4 (41.8-59.1) | -1.38 (-1.55 to -1.21) |
| Côte d'Ivoire | 205.9 (162.6-257.1) | 32.1 (25.4-40.1) | 785.4 (586-1058.8) | 43.8 (32.7-59) | 1.11 (0.97 to 1.24) |
| Democratic People's Republic of Korea | 523 (337.3-756.6) | 19.6 (12.7-28.4) | 1231.9 (886-1621.2) | 21.9 (15.7-28.8) | 0.67 (0.54 to 0.81) |
| Democratic Republic of the Congo | 710.7 (488.5-961.5) | 26.9 (18.5-36.4) | 2213.1 (1569.2-3055) | 36.6 (26-50.6) | 1.03 (0.93 to 1.13) |
| Denmark | 1405.5 (1299-1495.7) | 107.7 (99.6-114.6) | 1209.7 (1045.8-1339.7) | 62.8 (54.3-69.6) | -1.9 (-1.98 to -1.81) |
| Djibouti | 7.2 (5.4-9.5) | 33.7 (25.3-44.4) | 41.5 (29.3-58) | 40 (28.2-55.9) | 0.49 (0.44 to 0.53) |
| Dominica | 7.6 (6.1-9) | 77.1 (62.2-92) | 10.5 (8.1-13.4) | 69.9 (53.9-88.6) | -0.27 (-0.4 to -0.14) |
| Dominican Republic | 130.5 (110-154.7) | 21.4 (18-25.3) | 494.8 (374.3-639.3) | 29.6 (22.4-38.3) | 1.28 (1.03 to 1.53) |
| East Asia | 23108.6 (19256.6-27585.4) | 15.5 (12.9-18.5) | 65214 (52337.1-80094.5) | 16.6 (13.3-20.4) | 0.02 (-0.07 to 0.11) |
| Eastern Europe | 20479.8 (19654.9-21165.7) | 41.9 (40.2-43.3) | 29702.9 (26583.3-33273.5) | 47.8 (42.8-53.6) | 0.06 (-0.19 to 0.32) |
| Eastern Sub-Saharan Africa | 3939.1 (3343.6-4748.4) | 32.4 (27.5-39) | 11453.5 (9980.7-13353.8) | 42.4 (36.9-49.4) | 0.84 (0.76 to 0.91) |
| Ecuador | 150.8 (140.5-160.8) | 17.5 (16.3-18.7) | 754.3 (598.5-932.7) | 27.3 (21.6-33.7) | 1.56 (1.36 to 1.75) |
| Egypt | 600.5 (529.3-692.3) | 13.4 (11.8-15.5) | 3666 (2991.5-4491.1) | 33.1 (27-40.6) | 3.63 (3.13 to 4.14) |
| El Salvador | 75.7 (66.4-85.4) | 15.5 (13.6-17.5) | 277.7 (221.3-340.2) | 27.2 (21.6-33.3) | 1.81 (1.68 to 1.95) |
| Equatorial Guinea | 9.4 (6.5-13.1) | 29.5 (20.2-41.2) | 41.9 (25.9-63) | 52.9 (32.8-79.7) | 2.13 (2.02 to 2.24) |
| Eritrea | 63.1 (46-86.6) | 35.7 (26-49) | 220.8 (155.3-291.5) | 50.1 (35.2-66.2) | 1.16 (1.07 to 1.24) |
| Estonia | 188.9 (174-204) | 52.7 (48.5-56.9) | 221 (177.6-264.1) | 50.5 (40.6-60.4) | -0.23 (-0.36 to -0.11) |
| Eswatini | 16.8 (12.6-22.1) | 37.3 (27.8-48.9) | 54.1 (33.1-82.3) | 59.7 (36.5-90.9) | 1.72 (1.43 to 2.01) |
| Ethiopia | 1082.2 (755.6-1526.6) | 33.7 (23.5-47.5) | 2703.5 (2241.2-3305.6) | 39.5 (32.7-48.3) | 0.45 (0.27 to 0.64) |
| Fiji | 31.1 (24.1-40.5) | 55.3 (42.7-71.9) | 99.3 (72-127) | 72.6 (52.7-92.9) | 0.89 (0.76 to 1.02) |
| Finland | 698.7 (640.2-750.6) | 58.8 (53.9-63.2) | 937.9 (796-1041.8) | 46.6 (39.5-51.8) | -0.79 (-0.88 to -0.7) |
| France | 10678.9 (9743.7-11425) | 76.7 (70-82.1) | 13640.5 (11256.7-15252.7) | 61.7 (50.9-69) | -0.76 (-0.85 to -0.67) |
| Gabon | 44.3 (31.1-58.4) | 45.2 (31.7-59.7) | 94.2 (65.1-129.6) | 52.7 (36.4-72.5) | 0.26 (0.09 to 0.44) |
| Gambia | 7 (5.4-8.8) | 12.6 (9.7-16) | 32.4 (23.3-41.9) | 21 (15.1-27.1) | 1.6 (1.46 to 1.74) |
| Georgia | 632.2 (572-695.7) | 56.9 (51.5-62.6) | 791.5 (685.7-906.5) | 75.4 (65.3-86.3) | 1.54 (1.21 to 1.87) |
| Germany | 16650.8 (15208.5-17903.5) | 79.4 (72.5-85.3) | 18641.2 (15675.6-20824.6) | 59.2 (49.7-66.1) | -0.88 (-0.97 to -0.79) |
| Ghana | 323 (251.9-415.3) | 31.6 (24.7-40.7) | 1138.3 (850.8-1488.3) | 41.7 (31.2-54.6) | 0.83 (0.78 to 0.88) |
| Global | 238913.4 (223054.4-249779.1) | 35.6 (33.2-37.2) | 473476.5 (428994-508325.5) | 31.9 (28.9-34.2) | -0.46 (-0.5 to -0.41) |
| Greece | 1567.3 (1440.1-1670) | 58.6 (53.9-62.5) | 2581.8 (2169.3-2843.8) | 70.2 (59-77.4) | 0.21 (0.01 to 0.41) |
| Greenland | 3.6 (2.9-4.4) | 64.8 (52-80.8) | 4.1 (3-5.3) | 29.9 (22.2-38.9) | -2.77 (-2.98 to -2.56) |
| Grenada | 7.7 (6.9-8.5) | 64 (57.3-70.5) | 13.7 (12-15.4) | 68.3 (60-76.9) | 0.38 (0.07 to 0.7) |
| Guam | 4.1 (3.4-5.1) | 32.1 (26.3-39.2) | 8.7 (6.7-11.3) | 23.1 (17.9-30) | -0.32 (-0.65 to 0.01) |
| Guatemala | 61.5 (56.5-66.4) | 10.7 (9.8-11.5) | 325.1 (282.2-379.6) | 17.8 (15.4-20.7) | 1.82 (1.56 to 2.08) |
| Guinea | 139.3 (103.8-179.3) | 25.2 (18.7-32.4) | 301.4 (217.7-407.5) | 33 (23.8-44.6) | 0.83 (0.72 to 0.93) |
| Guinea-Bissau | 17.5 (12.4-25.2) | 27.5 (19.5-39.5) | 43.3 (29.6-59.1) | 37.7 (25.8-51.4) | 1.02 (0.96 to 1.08) |
| Guyana | 25.8 (22.5-29.4) | 42.1 (36.7-47.9) | 56.4 (43.5-70.8) | 50 (38.7-62.9) | 0.83 (0.63 to 1.04) |
| Haiti | 214.5 (132.2-338.3) | 39.8 (24.5-62.7) | 572.9 (359.8-870.1) | 48.3 (30.4-73.4) | 0.72 (0.62 to 0.82) |
| High SDI | 112673.8 (104275.5-117053) | 60.4 (55.9-62.8) | 147971.6 (128248.7-158356.4) | 42.9 (37.2-45.9) | -1.23 (-1.28 to -1.17) |
| High-income Asia Pacific | 4498.9 (4187.8-4692.4) | 12.9 (12-13.4) | 16436.2 (13190.7-18277.7) | 23.3 (18.7-25.9) | 2.06 (1.98 to 2.13) |
| High-income North America | 42195.1 (38820.1-44093.2) | 72.8 (67-76.1) | 51049.9 (44977.5-54477.3) | 45.4 (40-48.4) | -1.82 (-1.9 to -1.74) |
| High-middle SDI | 64355.8 (60604.3-67171.9) | 37.3 (35.1-38.9) | 114291.2 (102333.5-123847) | 33 (29.5-35.7) | -0.54 (-0.67 to -0.41) |
| Honduras | 48 (30-69.7) | 14.4 (9-20.9) | 297.1 (216.7-412.8) | 28.2 (20.6-39.3) | 2.19 (1.99 to 2.39) |
| Hungary | 1649.4 (1462.5-1850.8) | 64 (56.8-71.9) | 1923 (1628.2-2221.4) | 60.5 (51.2-69.9) | -0.54 (-0.7 to -0.37) |
| Iceland | 31.8 (28.7-34.6) | 66.7 (60.2-72.6) | 44.8 (37.7-50.6) | 46 (38.7-51.9) | -1.2 (-1.35 to -1.04) |
| India | 10781.5 (8980.4-12840.3) | 14.1 (11.7-16.7) | 48247.3 (40236.6-57952.7) | 24 (20-28.8) | 1.75 (1.62 to 1.88) |
| Indonesia | 3952.3 (2582.8-5733.6) | 24.5 (16-35.5) | 14531.5 (9317.8-21295.7) | 34.7 (22.2-50.8) | 1.02 (0.87 to 1.16) |
| Iran (Islamic Republic of) | 443.8 (382.2-509.3) | 9.6 (8.3-11) | 2235.3 (1986.3-2480) | 17.2 (15.3-19.1) | 2.48 (2.22 to 2.73) |
| Iraq | 263.8 (201.2-344.6) | 20.9 (15.9-27.3) | 1146.6 (800.2-1539.9) | 29.5 (20.6-39.6) | 1.08 (0.97 to 1.18) |
| Ireland | 544.2 (504.6-583.2) | 80 (74.1-85.7) | 595.8 (506.7-671.8) | 45.4 (38.6-51.2) | -1.66 (-1.76 to -1.56) |
| Israel | 570.9 (514.7-620.7) | 70.4 (63.5-76.6) | 1073.6 (879-1210.5) | 53.7 (44-60.6) | -1.56 (-1.87 to -1.24) |
| Italy | 10287.3 (9390.9-10807.3) | 67.6 (61.7-71) | 12940.6 (10443.9-14459.4) | 57 (46-63.7) | -0.58 (-0.64 to -0.52) |
| Jamaica | 141.2 (128.3-156) | 47.9 (43.6-53) | 322.9 (245.2-400.6) | 61 (46.4-75.7) | 0.65 (0.38 to 0.92) |
| Japan | 3950.4 (3659.4-4124.2) | 13.3 (12.4-13.9) | 14170.1 (11280.9-15792.5) | 27.1 (21.6-30.3) | 2.43 (2.36 to 2.5) |
| Jordan | 53.8 (41.8-67.6) | 25 (19.4-31.4) | 358.1 (254.1-487.6) | 28.5 (20.2-38.8) | 0.7 (0.31 to 1.09) |
| Kazakhstan | 1033.9 (898.3-1178.1) | 49.4 (43-56.3) | 956.7 (786.9-1120.8) | 30.1 (24.8-35.3) | -0.71 (-1.15 to -0.26) |
| Kenya | 331.6 (240.7-447.6) | 24.9 (18.1-33.6) | 1525.8 (1106-2086.6) | 40.8 (29.6-55.8) | 1.83 (1.74 to 1.92) |
| Kiribati | 2.7 (2-3.6) | 45.5 (33.7-59.9) | 7.7 (5.7-10.1) | 61 (45.6-80.5) | 0.98 (0.85 to 1.11) |
| Kuwait | 16.7 (14.7-18.4) | 18.3 (16.1-20.1) | 74 (61.7-85.8) | 15.9 (13.2-18.4) | 0.09 (-0.43 to 0.62) |
| Kyrgyzstan | 196 (177.2-215.6) | 38.1 (34.4-41.9) | 210.1 (174.1-248.2) | 24.8 (20.6-29.4) | -1.42 (-1.6 to -1.23) |
| Lao People's Democratic Republic | 86.4 (51.4-138.8) | 25.2 (15-40.5) | 259.9 (182.5-364.2) | 33.6 (23.6-47.1) | 1.01 (0.93 to 1.09) |
| Latvia | 299.3 (271.1-334.3) | 47.9 (43.4-53.5) | 371.8 (310.4-430.2) | 56.5 (47.2-65.4) | 0.74 (0.56 to 0.92) |
| Lebanon | 148.7 (111-196.9) | 39.2 (29.3-51.9) | 500.5 (396.6-620.2) | 51.1 (40.5-63.3) | 1.18 (0.99 to 1.38) |
| Lesotho | 45.5 (33.3-62) | 32.5 (23.8-44.4) | 111.2 (72.5-160.7) | 61.6 (40.2-89) | 2.53 (2.18 to 2.88) |
| Liberia | 42.5 (33.6-52.4) | 22.5 (17.9-27.8) | 99.8 (71.6-135.5) | 30.2 (21.7-41) | 1.1 (1 to 1.19) |
| Libya | 48.7 (38.4-60) | 15.7 (12.3-19.3) | 203.8 (156.6-262.9) | 24.4 (18.7-31.4) | 1.68 (1.44 to 1.93) |
| Lithuania | 336.5 (302.4-373) | 42.5 (38.2-47.1) | 488 (399.3-577) | 50.4 (41.3-59.6) | 0.67 (0.45 to 0.9) |
| Low SDI | 8623.6 (7412.7-9880.3) | 23.1 (19.9-26.5) | 25683.1 (22749.5-28656.3) | 31.3 (27.7-34.9) | 0.98 (0.88 to 1.07) |
| Low-middle SDI | 17386.6 (15283-19688.5) | 17.2 (15.2-19.5) | 66913.2 (59543-73575.3) | 27.8 (24.7-30.5) | 1.57 (1.53 to 1.61) |
| Luxembourg | 79.7 (73.7-85.3) | 85.3 (79-91.4) | 89.3 (77.5-100.5) | 50.3 (43.6-56.6) | -1.3 (-1.48 to -1.12) |
| Madagascar | 260.1 (210.9-320.5) | 30.8 (25-37.9) | 577.1 (407.7-753.9) | 31.2 (22.1-40.8) | -0.04 (-0.2 to 0.12) |
| Malawi | 173.7 (134.9-220.2) | 27.6 (21.4-35) | 490.8 (367.1-639.7) | 42 (31.4-54.7) | 1.31 (1.23 to 1.38) |
| Malaysia | 555.8 (471.6-645.7) | 37.7 (32-43.8) | 2398.4 (2054.1-2777.4) | 48.9 (41.9-56.7) | 0.75 (0.64 to 0.85) |
| Maldives | 2.1 (1.3-3.4) | 14 (8.5-22.2) | 8.4 (6.7-10.3) | 15.4 (12.3-18.9) | -0.01 (-0.34 to 0.31) |
| Mali | 187.1 (145.6-227.8) | 28 (21.8-34.1) | 473.8 (343.1-647) | 32.7 (23.7-44.7) | 0.5 (0.42 to 0.58) |
| Malta | 62.8 (57.3-68.7) | 86.7 (79.1-94.9) | 86.5 (71.1-99.5) | 54.8 (45.1-63.1) | -2 (-2.2 to -1.8) |
| Marshall Islands | 1 (0.7-1.4) | 38.1 (26.8-53.6) | 2.8 (1.7-4.2) | 47.8 (29.5-72.3) | 0.42 (0.32 to 0.52) |
| Mauritania | 53.1 (37.6-71.1) | 32 (22.6-42.9) | 130.9 (98.3-169.9) | 36.5 (27.4-47.4) | 0.22 (0.11 to 0.34) |
| Mauritius | 30.6 (27.9-32.8) | 25.1 (23-27) | 162.3 (146.2-174.5) | 48 (43.2-51.6) | 1.66 (1.33 to 2) |
| Mexico | 1578.4 (1518.2-1631.2) | 22.7 (21.8-23.5) | 6095.7 (5206.5-7033.9) | 28.3 (24.2-32.6) | 0.54 (0.44 to 0.64) |
| Micronesia (Federated States of) | 3.9 (2.7-5.4) | 48.9 (33.6-67.7) | 7.8 (5.8-10.5) | 59.4 (43.9-80.1) | 0.47 (0.36 to 0.58) |
| Middle SDI | 35517.4 (32560.6-38827.7) | 20.5 (18.8-22.4) | 117986.1 (106020.7-130990.1) | 25.1 (22.6-27.9) | 0.54 (0.48 to 0.6) |
| Monaco | 10.4 (7.4-13.9) | 94.3 (66.3-125.7) | 15.3 (11.4-20) | 97.7 (72.9-127.6) | 0.18 (0.07 to 0.28) |
| Mongolia | 18.7 (13.9-24.2) | 10.8 (8-14) | 45.5 (34.4-57.6) | 11.5 (8.7-14.6) | -0.08 (-0.23 to 0.08) |
| Montenegro | 55.3 (41.8-71.7) | 50.7 (38.4-65.8) | 112.9 (87.4-143.8) | 64.7 (50.1-82.5) | 0.91 (0.78 to 1.04) |
| Morocco | 238.1 (188.8-301.3) | 10.2 (8.1-12.8) | 988.3 (704.7-1356.5) | 16.5 (11.7-22.6) | 1.8 (1.71 to 1.89) |
| Mozambique | 286 (234.8-344) | 29.3 (24-35.2) | 802.1 (563.5-1051.5) | 44.8 (31.5-58.8) | 1.68 (1.58 to 1.78) |
| Myanmar | 1108.2 (785.1-1536.9) | 28.3 (20.1-39.3) | 2995 (2341.2-3878.3) | 35.5 (27.7-45.9) | 0.67 (0.59 to 0.75) |
| Namibia | 41.5 (33.4-51.5) | 38.2 (30.8-47.4) | 163 (108.4-220.8) | 72.1 (48-97.7) | 2.47 (2.31 to 2.63) |
| Nauru | 0.3 (0.2-0.5) | 45.9 (27.6-70.6) | 0.7 (0.4-1) | 72.9 (44.6-111.8) | 1.54 (1.48 to 1.61) |
| Nepal | 195.8 (141.5-263.3) | 12.7 (9.2-17.1) | 660.4 (464.7-938.4) | 16.7 (11.7-23.7) | 0.96 (0.71 to 1.22) |
| Netherlands | 2963.3 (2625.2-3210.8) | 89.5 (79.3-97) | 3313.7 (2848.2-3731.8) | 56.6 (48.7-63.8) | -1.79 (-1.93 to -1.64) |
| New Zealand | 501.5 (456-540.1) | 76.2 (69.3-82.1) | 604.9 (504.3-674.7) | 42.4 (35.4-47.3) | -1.9 (-1.98 to -1.82) |
| Nicaragua | 29 (24.4-34.5) | 11.7 (9.8-13.9) | 155.5 (123.7-195.1) | 19.1 (15.2-24) | 1.94 (1.7 to 2.19) |
| Niger | 68.1 (50.7-91.5) | 15.5 (11.5-20.8) | 258.9 (188.8-352.2) | 18.6 (13.6-25.3) | 0.58 (0.53 to 0.64) |
| Nigeria | 2775.7 (2130.4-3500.3) | 38.1 (29.2-48) | 9611.4 (6949.9-13301.5) | 64.1 (46.3-88.7) | 1.9 (1.64 to 2.15) |
| Niue | 0.3 (0.2-0.3) | 72.2 (56.3-91.4) | 0.3 (0.2-0.4) | 73.4 (55.9-94.6) | 0.19 (0.08 to 0.3) |
| North Africa and Middle East | 3560.6 (3253.4-3929.8) | 12.6 (11.5-13.9) | 17191.5 (15228.9-19339.3) | 22.6 (20-25.4) | 2.4 (2.15 to 2.66) |
| North Macedonia | 144.9 (120-172.2) | 44 (36.5-52.4) | 337.2 (264-418.5) | 56.9 (44.5-70.6) | 0.96 (0.73 to 1.2) |
| Northern Mariana Islands | 1 (0.7-1.3) | 39.6 (29.9-51.8) | 4.9 (4.1-5.8) | 50.3 (41.9-59.3) | 0.44 (0.27 to 0.62) |
| Norway | 713.4 (648.3-753.6) | 65.9 (59.9-69.6) | 642.6 (552.1-708.1) | 39.6 (34-43.7) | -1.9 (-2.05 to -1.75) |
| Oceania | 150.2 (119.4-188.1) | 31.2 (24.8-39.1) | 447.1 (367-539.3) | 36.2 (29.7-43.7) | 0.45 (0.41 to 0.5) |
| Oman | 8.4 (6.2-11.4) | 8.4 (6.1-11.3) | 27.7 (21.4-35.1) | 8.9 (6.8-11.2) | 0.52 (0.17 to 0.86) |
| Pakistan | 3057.9 (2311.9-3888.4) | 33.5 (25.4-42.6) | 10368.8 (7635.9-13501.3) | 52.3 (38.5-68.1) | 1.14 (0.9 to 1.38) |
| Palau | 1.4 (1.1-1.8) | 86.9 (67.1-111.6) | 3.2 (2.5-4.1) | 76.4 (59-97.4) | -0.56 (-0.63 to -0.49) |
| Palestine | 53.3 (39.7-72.4) | 36.7 (27.4-49.9) | 181.9 (146.9-222.4) | 42.3 (34.1-51.7) | 0.73 (0.56 to 0.91) |
| Panama | 57.4 (52.5-62.6) | 23.7 (21.7-25.8) | 222.8 (174.2-267.9) | 30 (23.4-36) | 0.9 (0.75 to 1.05) |
| Papua New Guinea | 73.2 (51.9-104.2) | 24.7 (17.5-35.2) | 229.8 (167.8-317.6) | 28 (20.4-38.7) | 0.28 (0.21 to 0.35) |
| Paraguay | 92.6 (74.2-114.6) | 25.5 (20.5-31.6) | 383.7 (278-507) | 38.8 (28.1-51.2) | 1.45 (1.25 to 1.66) |
| Peru | 473.3 (380.6-576.4) | 24 (19.3-29.2) | 1397.3 (1002.5-1911.7) | 25 (17.9-34.2) | -0.24 (-0.44 to -0.05) |
| Philippines | 1668.4 (1486.5-1880.5) | 34.9 (31.1-39.4) | 6464.8 (5249.2-7899.4) | 46.4 (37.6-56.6) | 0.79 (0.68 to 0.91) |
| Poland | 3707.7 (3520.8-3852.1) | 48.1 (45.7-50) | 7570.6 (6628.5-8408.3) | 62.5 (54.7-69.4) | 0.5 (0.32 to 0.68) |
| Portugal | 1364.4 (1259.6-1458.6) | 56 (51.7-59.8) | 1802.7 (1521.3-2006.6) | 46.3 (39.1-51.6) | -0.72 (-0.79 to -0.65) |
| Puerto Rico | 247.2 (226-266.3) | 40.7 (37.2-43.9) | 503 (415-590.9) | 43.1 (35.5-50.6) | 0.09 (-0.04 to 0.22) |
| Qatar | 4.8 (3.7-6) | 29.3 (22.8-36.5) | 41.1 (30.1-54.7) | 26.8 (19.7-35.7) | -0.51 (-0.75 to -0.27) |
| Republic of Korea | 439.6 (384.6-517.9) | 8.8 (7.7-10.4) | 1880.6 (1432.5-2311.6) | 11.2 (8.6-13.8) | 0.76 (0.67 to 0.85) |
| Republic of Moldova | 354.9 (318.6-389.6) | 46 (41.3-50.5) | 480.7 (410.1-561.4) | 45.5 (38.8-53.2) | 0.63 (0.32 to 0.93) |
| Romania | 1784.7 (1655.5-1937.1) | 35.7 (33.1-38.7) | 3475.1 (3031.6-3977) | 57.9 (50.5-66.2) | 1.42 (1.26 to 1.57) |
| Russian Federation | 11747.3 (11277.1-12094.1) | 37.2 (35.7-38.3) | 20866.9 (18700.3-22890.1) | 49.1 (44-53.9) | 0.52 (0.19 to 0.86) |
| Rwanda | 217.3 (159-295.6) | 46.4 (34-63.2) | 515.8 (372.5-707) | 48.8 (35.2-66.9) | -0.3 (-0.49 to -0.11) |
| Saint Kitts and Nevis | 6.4 (5.8-7) | 99.5 (91.3-108.7) | 8.3 (6.8-9.7) | 64.1 (53.1-75.2) | -0.89 (-1.18 to -0.59) |
| Saint Lucia | 9.4 (8.6-10.1) | 64.1 (59-69.3) | 21 (17.5-24.9) | 49.9 (41.6-59.1) | -1.31 (-1.62 to -1.01) |
| Saint Vincent and the Grenadines | 7.9 (7.1-8.6) | 65.7 (58.9-71.3) | 14.6 (12.7-16.4) | 57.5 (50.1-64.7) | -0.49 (-0.72 to -0.25) |
| Samoa | 4.9 (3.7-6.2) | 34 (25.7-43) | 10.2 (7.8-13.5) | 41.4 (31.6-55) | 0.61 (0.55 to 0.67) |
| San Marino | 3.2 (2.4-4.1) | 54.8 (41.3-69.5) | 3.7 (2.3-5.2) | 31.1 (19-44.3) | -1.28 (-1.59 to -0.97) |
| Sao Tome and Principe | 2.6 (2-3.2) | 22.9 (18.2-28.8) | 6.2 (4.6-8.1) | 34 (25.3-44.3) | 1.35 (1.27 to 1.43) |
| Saudi Arabia | 85.8 (66.6-110.1) | 9.6 (7.5-12.4) | 367.3 (284.9-471.1) | 12.2 (9.5-15.6) | 0.6 (0.26 to 0.94) |
| Senegal | 117.8 (94.3-151.7) | 22.3 (17.8-28.7) | 440.3 (335.9-580.9) | 34.1 (26-45) | 1.45 (1.35 to 1.55) |
| Serbia | 1189.3 (889.1-1540.3) | 57.2 (42.8-74.1) | 2020.5 (1544.1-2606.6) | 72 (55-92.9) | 0.65 (0.56 to 0.74) |
| Seychelles | 3.2 (2.8-3.7) | 33.6 (28.8-38.7) | 9.1 (7.6-10.6) | 43.7 (36.7-51) | 0.66 (0.21 to 1.11) |
| Sierra Leone | 67.4 (50.3-86.7) | 20.3 (15.2-26.2) | 172.6 (128.5-227.7) | 28.6 (21.3-37.7) | 1.3 (1.21 to 1.4) |
| Singapore | 104.3 (96.4-113.5) | 28.8 (26.6-31.3) | 366.2 (322.6-402.6) | 24.1 (21.2-26.5) | -0.32 (-0.48 to -0.15) |
| Slovakia | 518 (434-622.7) | 49.9 (41.8-60) | 897.6 (659.5-1162.1) | 54.7 (40.2-70.8) | 0.09 (-0.05 to 0.24) |
| Slovenia | 251.6 (229.9-273.1) | 58.4 (53.4-63.4) | 396 (325.3-474.3) | 54.1 (44.4-64.8) | -0.48 (-0.68 to -0.28) |
| Solomon Islands | 4.6 (3-7) | 20.5 (13.5-30.8) | 19.2 (13.1-27.1) | 35.4 (24.1-50) | 1.82 (1.69 to 1.95) |
| Somalia | 98.6 (68.3-134.1) | 27.4 (19-37.2) | 287 (195.9-387.5) | 30.4 (20.8-41.1) | 0.28 (0.23 to 0.32) |
| South Africa | 1243.9 (926.6-1570.3) | 36.9 (27.5-46.5) | 4386.2 (3956.6-4842.5) | 55.6 (50.2-61.4) | 1.45 (1.2 to 1.7) |
| South Asia | 14517.5 (12545-16545.8) | 15.3 (13.2-17.4) | 61153.8 (52838.2-71109.7) | 24.6 (21.3-28.6) | 1.46 (1.35 to 1.56) |
| South Sudan | 109.4 (79.2-153.7) | 26.4 (19.1-37.1) | 190.1 (131-264) | 30.5 (21-42.4) | 0.4 (0.28 to 0.52) |
| Southeast Asia | 10348.9 (8652.6-12524.1) | 24.4 (20.4-29.6) | 39390.5 (32752.7-48049.4) | 34.4 (28.6-41.9) | 1.07 (0.96 to 1.18) |
| Southern Latin America | 5339 (4990.2-5649.6) | 67.4 (63-71.3) | 8082 (7170.5-8834.7) | 54.9 (48.7-60) | -0.6 (-0.8 to -0.4) |
| Southern Sub-Saharan Africa | 1603.2 (1281.9-1946.8) | 36.2 (29-44) | 5540.8 (5063.7-6054) | 56.9 (52-62.2) | 1.68 (1.41 to 1.96) |
| Spain | 5188.5 (4663-5641.8) | 54.3 (48.8-59) | 6076.7 (4971.6-6957.7) | 39.3 (32.1-45) | -1.05 (-1.11 to -0.99) |
| Sri Lanka | 348.2 (287.3-424.6) | 19.6 (16.1-23.9) | 1298.5 (867.5-1757.2) | 27 (18-36.5) | 1.18 (1 to 1.36) |
| Sudan | 133.3 (92.5-185.6) | 9 (6.2-12.5) | 395.3 (277.3-554.3) | 12.7 (8.9-17.8) | 1.26 (1.13 to 1.39) |
| Suriname | 15 (12.8-17.2) | 34.4 (29.4-39.6) | 42.5 (32.6-54.4) | 38.1 (29.2-48.7) | 0.71 (0.52 to 0.91) |
| Sweden | 1359.1 (1228.3-1476.1) | 57.2 (51.7-62.2) | 1460.7 (1199.2-1684.4) | 43.4 (35.6-50) | -0.71 (-0.89 to -0.53) |
| Switzerland | 1155.5 (1038.1-1246.3) | 68.4 (61.5-73.8) | 1342.8 (1100.7-1526.4) | 45.8 (37.6-52.1) | -1.24 (-1.4 to -1.09) |
| Syrian Arab Republic | 122.9 (95.9-155.4) | 13.9 (10.9-17.6) | 443.6 (322.9-596.4) | 18.8 (13.7-25.2) | 0.86 (0.74 to 0.98) |
| Taiwan (Province of China) | 390.2 (364.5-418.1) | 14.1 (13.1-15.1) | 2047.5 (1813.7-2268.1) | 27.2 (24.1-30.1) | 2.28 (2 to 2.56) |
| Tajikistan | 125.4 (100.2-152.7) | 26.8 (21.4-32.6) | 195.7 (132.7-271.8) | 19.1 (12.9-26.5) | -1.2 (-1.41 to -0.99) |
| Thailand | 1150.3 (886.9-1416.5) | 19.4 (14.9-23.9) | 6197.7 (4648.5-7844.6) | 31.9 (23.9-40.4) | 1.8 (1.51 to 2.09) |
| Timor-Leste | 7.8 (4.9-12.2) | 18.1 (11.2-28) | 37.1 (25.1-51.4) | 26.1 (17.7-36.2) | 1.43 (1.27 to 1.59) |
| Togo | 49.7 (40.2-61.9) | 25.5 (20.6-31.7) | 223.9 (160.9-303.8) | 35.8 (25.8-48.6) | 1.14 (1.1 to 1.18) |
| Tokelau | 0.1 (0.1-0.2) | 51.2 (34.8-70.7) | 0.2 (0.1-0.2) | 64.8 (47-86) | 0.89 (0.77 to 1.01) |
| Tonga | 6 (4.7-7.5) | 62.8 (49.6-78.8) | 10.8 (8.1-14.4) | 80.7 (60.1-106.9) | 0.86 (0.76 to 0.96) |
| Trinidad and Tobago | 77.9 (72.2-84.2) | 56.7 (52.6-61.4) | 187.8 (144.6-234.8) | 53.9 (41.5-67.4) | -0.22 (-0.41 to -0.03) |
| Tropical Latin America | 5113 (4806-5353) | 33.8 (31.7-35.4) | 16499 (14972.3-17651.5) | 37.2 (33.8-39.8) | 0.15 (0.07 to 0.23) |
| Tunisia | 150 (120.7-185) | 17.2 (13.9-21.2) | 514.7 (369-702.7) | 22 (15.8-30.1) | 0.59 (0.44 to 0.73) |
| Turkey | 652.1 (520.1-808.8) | 10.9 (8.7-13.5) | 4312.1 (3301.1-5426.9) | 26.1 (20-32.8) | 3.96 (3.26 to 4.68) |
| Turkmenistan | 88 (77.2-99.3) | 27.1 (23.8-30.6) | 164.9 (124.6-216.6) | 23.3 (17.6-30.6) | -0.22 (-0.68 to 0.25) |
| Tuvalu | 0.5 (0.3-0.7) | 41.5 (27.1-60.1) | 1 (0.7-1.3) | 55.2 (40.3-73.6) | 0.84 (0.7 to 0.98) |
| Uganda | 419 (315.2-552.2) | 40.1 (30.1-52.8) | 1400.5 (1060.8-1852.5) | 59.1 (44.7-78.1) | 0.88 (0.66 to 1.11) |
| Ukraine | 6696.4 (6186.6-7217.1) | 53.7 (49.6-57.9) | 6172.7 (4137.1-8697.9) | 45.5 (30.5-64.1) | -0.94 (-1.14 to -0.73) |
| United Arab Emirates | 21.4 (16.4-27.4) | 38.2 (29.2-48.8) | 153.1 (107.9-207.9) | 21.3 (15-29) | -0.63 (-1.25 to -0.02) |
| United Kingdom | 14574.1 (13572.7-15046) | 98.1 (91.4-101.3) | 12166.4 (10631.9-12968) | 57.9 (50.6-61.7) | -1.8 (-1.89 to -1.71) |
| United Republic of Tanzania | 601.5 (481.8-769.9) | 33.3 (26.7-42.7) | 1808.8 (1367.6-2374.1) | 43.9 (33.2-57.6) | 0.93 (0.9 to 0.95) |
| United States Virgin Islands | 8.7 (7.2-10.6) | 61.9 (51.4-75.6) | 15.1 (10.4-20.7) | 46.9 (32.4-64.3) | -0.84 (-1.12 to -0.55) |
| United States of America | 38388.5 (35276.9-40172.3) | 73.2 (67.2-76.6) | 45732.8 (40386.1-48880.1) | 45.6 (40.3-48.8) | -1.83 (-1.91 to -1.74) |
| Uruguay | 590.6 (543.9-632.5) | 87.5 (80.6-93.7) | 790.6 (692.9-880.8) | 87.4 (76.6-97.4) | -0.21 (-0.35 to -0.06) |
| Uzbekistan | 534.2 (461.2-614.9) | 27.3 (23.6-31.4) | 1034 (834.2-1265.8) | 22.2 (17.9-27.2) | -0.52 (-0.75 to -0.3) |
| Vanuatu | 2.3 (1.6-3.2) | 22.7 (15.6-31.9) | 10.3 (7.7-13.2) | 35.8 (27-46.1) | 1.33 (1.22 to 1.43) |
| Venezuela (Bolivarian Republic of) | 438.3 (405.2-469.8) | 28 (25.9-30) | 2186.7 (1719.2-2750.4) | 41.8 (32.9-52.6) | 1.14 (1.01 to 1.26) |
| Viet Nam | 1218.3 (963.7-1590.6) | 17.5 (13.8-22.8) | 4063.5 (3025.3-5328.4) | 23.3 (17.3-30.5) | 0.99 (0.89 to 1.09) |
| Western Europe | 74000.5 (68450.4-77211.4) | 76.2 (70.5-79.5) | 82775.4 (69888.5-89714.1) | 55.5 (46.9-60.2) | -1.05 (-1.1 to -1.01) |
| Western Sub-Saharan Africa | 4702.1 (3899-5514.6) | 32.6 (27-38.2) | 15551.9 (12529.8-19349.9) | 48.4 (39-60.2) | 1.38 (1.23 to 1.53) |
| Yemen | 66.3 (44.6-91.4) | 8.3 (5.6-11.4) | 307.6 (219.9-416.2) | 13.7 (9.8-18.5) | 1.81 (1.67 to 1.95) |
| Zambia | 137.6 (103.3-181.9) | 29.9 (22.4-39.5) | 611.2 (402.8-892) | 56.6 (37.3-82.6) | 2.28 (2.06 to 2.5) |
| Zimbabwe | 223.8 (171.8-287.6) | 33.8 (25.9-43.4) | 713.8 (526.8-923.2) | 64.5 (47.6-83.4) | 2.8 (2.19 to 3.42) |

**Table S7. DALYs of Breast Cancer Between 1990 and 2021 at the National Level**

| **location** | **Number 1990** | **ASR 1990 (95%UI)** | **Number 2021** | **ASR 2021 (95%UI)** | **EAPC**  **(95%CI)** |
| --- | --- | --- | --- | --- | --- |
| Afghanistan | 4926.6 (2841.5-7954.4) | 405.4 (233.8-654.6) | 8391.8 (4897.3-13792.2) | 683 (398.6-1122.5) | 1.74 (1.62 to 1.86) |
| Albania | 1515.7 (1194.3-1923.5) | 436.8 (344.2-554.3) | 3975.6 (2840.6-5384.6) | 505.6 (361.3-684.8) | 0.9 (0.73 to 1.06) |
| Algeria | 6285.6 (4684.1-8160.1) | 300 (223.6-389.5) | 21575.8 (16328.9-28294.2) | 355.3 (268.9-465.9) | 0.55 (0.47 to 0.62) |
| American Samoa | 43.4 (35.4-52.8) | 1187.6 (967-1444.1) | 148.6 (118.1-185) | 1759.9 (1397.8-2190.2) | 1.59 (1.48 to 1.7) |
| Andean Latin America | 18913.8 (15837.3-22628.4) | 563.6 (471.9-674.3) | 64341.4 (50351.3-82549.2) | 649.5 (508.3-833.3) | 0.23 (0.13 to 0.34) |
| Andorra | 113.4 (76.3-163.8) | 1149.5 (773.3-1659.7) | 239.4 (168.8-332.9) | 904.8 (637.8-1258) | -0.36 (-0.54 to -0.17) |
| Angola | 4003.4 (2757.8-5863.6) | 642.7 (442.7-941.3) | 18694.1 (12473.8-25641) | 966.8 (645.1-1326.1) | 1.34 (1.28 to 1.41) |
| Antigua and Barbuda | 117.9 (104.4-132) | 1364.9 (1208.4-1527.6) | 316.4 (291.3-346) | 1677.1 (1544.1-1834) | 0.65 (0.43 to 0.88) |
| Argentina | 91949.8 (85194.6-97899.3) | 1652.4 (1531-1759.3) | 123425.2 (111877.8-134747.3) | 1322.4 (1198.7-1443.7) | -0.62 (-0.8 to -0.44) |
| Armenia | 7340.5 (6775.5-7948.2) | 1508 (1391.9-1632.9) | 10091.7 (8821.5-11645) | 1282.9 (1121.4-1480.4) | -0.74 (-1.02 to -0.45) |
| Australasia | 55897.2 (52136.8-59507.3) | 1418.9 (1323.4-1510.5) | 78460.7 (68586.9-87940.1) | 888.1 (776.4-995.4) | -1.59 (-1.65 to -1.53) |
| Australia | 44281.3 (41028.9-47422) | 1349.4 (1250.3-1445.1) | 64897 (56642.8-73164.2) | 876.1 (764.6-987.7) | -1.51 (-1.59 to -1.44) |
| Austria | 32124.6 (29620-34438.9) | 1650.9 (1522.2-1769.8) | 28835.3 (25219.2-32094.1) | 975.8 (853.4-1086) | -1.56 (-1.62 to -1.49) |
| Azerbaijan | 7577.3 (6311.1-8678.5) | 868.8 (723.6-995) | 15278.2 (11391-19682.7) | 797.5 (594.6-1027.4) | -0.11 (-0.31 to 0.08) |
| Bahamas | 430.2 (388.6-473.9) | 1751.9 (1582.7-1930) | 1310.7 (1080.5-1591.4) | 1821.4 (1501.6-2211.5) | 0.33 (0.16 to 0.49) |
| Bahrain | 319.9 (260.9-395.4) | 1140.1 (929.9-1409.1) | 1646 (1203.4-2268.3) | 1013.7 (741.2-1396.9) | -1.13 (-1.44 to -0.83) |
| Bangladesh | 13655.9 (10295.8-18220.4) | 180.3 (135.9-240.5) | 51901.4 (38320.3-69513.3) | 222.1 (164-297.5) | 0.19 (-0.06 to 0.44) |
| Barbados | 760.6 (682.2-835.2) | 1622.2 (1455-1781.4) | 1704.7 (1376.9-2111.5) | 1870.9 (1511.2-2317.3) | 0.79 (0.55 to 1.03) |
| Belarus | 21769.1 (19829.4-23774.8) | 940.4 (856.6-1027) | 26303.5 (20993.7-32551) | 914.4 (729.8-1131.6) | -0.97 (-1.25 to -0.69) |
| Belgium | 51953.9 (47506.2-55401.7) | 1981.5 (1811.9-2113) | 45243.2 (39169.9-50430.2) | 1196.7 (1036-1333.9) | -1.55 (-1.65 to -1.44) |
| Belize | 68.9 (62.1-76.3) | 447.8 (403.6-495.7) | 324.4 (282.5-369.9) | 647.7 (564-738.5) | 1.03 (0.72 to 1.35) |
| Benin | 1844.7 (1455.9-2268.7) | 576.6 (455.1-709.1) | 5608.2 (4176.7-7437.5) | 678.1 (505-899.3) | 0.48 (0.41 to 0.55) |
| Bermuda | 209.3 (186.2-233.4) | 1978.1 (1759.9-2206.4) | 255.7 (212.6-325.6) | 1097.7 (912.6-1398.1) | -2.36 (-2.6 to -2.11) |
| Bhutan | 135.3 (93-183.7) | 340.9 (234.3-463) | 384.6 (267.6-533) | 386.1 (268.7-535.2) | 0.38 (0.26 to 0.5) |
| Bolivia (Plurinational State of) | 4127.3 (2578.7-6150.7) | 787.2 (491.8-1173.1) | 14143.5 (8971.9-21089.6) | 915.2 (580.5-1364.6) | 0.38 (0.34 to 0.42) |
| Bosnia and Herzegovina | 5183.6 (4325.7-5951.6) | 706.7 (589.7-811.3) | 11013.7 (8684.9-13590) | 1005.6 (792.9-1240.8) | 1.66 (1.44 to 1.88) |
| Botswana | 779.6 (543.2-1047.7) | 838.5 (584.3-1127) | 2661.5 (1985.6-3643.9) | 1097.9 (819.1-1503.1) | 1.32 (1.01 to 1.63) |
| Brazil | 122047.6 (115849.4-127575.4) | 825.8 (783.9-863.2) | 377024.3 (345997.2-401231.9) | 870.6 (798.9-926.5) | -0.03 (-0.1 to 0.04) |
| Brunei Darussalam | 123.5 (90.6-167.1) | 788.6 (578.5-1067.2) | 532.7 (409.5-677.1) | 889.8 (684-1130.9) | 0.98 (0.78 to 1.19) |
| Bulgaria | 23223.6 (20510.7-26541.6) | 1026.1 (906.2-1172.7) | 32029.2 (26348.2-38094.8) | 1347.5 (1108.5-1602.7) | 1.08 (0.94 to 1.21) |
| Burkina Faso | 5998.8 (4613.5-7602.6) | 829.4 (637.9-1051.2) | 14572.8 (10655.3-19403.7) | 969.3 (708.7-1290.6) | 0.51 (0.4 to 0.63) |
| Burundi | 3378 (2440.3-4674.2) | 897.6 (648.4-1242) | 5573.8 (4188.2-7406.7) | 696.3 (523.2-925.2) | -1.25 (-1.42 to -1.07) |
| Cabo Verde | 266.6 (207.2-333.2) | 675.6 (525-844.2) | 587.1 (440.1-763.1) | 766 (574.2-995.6) | 0.68 (0.48 to 0.88) |
| Cambodia | 5329.1 (3282.7-8260.7) | 719.3 (443.1-1114.9) | 23417.8 (16556.2-32174.2) | 1077.3 (761.6-1480.1) | 1.36 (1.31 to 1.42) |
| Cameroon | 5490.9 (4260.6-7031.3) | 741.6 (575.4-949.6) | 18106.8 (12705.8-25130.9) | 889.6 (624.2-1234.6) | 0.49 (0.4 to 0.58) |
| Canada | 86104.3 (78657.9-92758.5) | 1577.3 (1440.9-1699.2) | 110026.9 (96050.9-122220.8) | 896.6 (782.7-996) | -1.94 (-2.02 to -1.86) |
| Caribbean | 39474.6 (36118-43533) | 915.9 (838.1-1010.1) | 91886.4 (78920.9-105565.2) | 992.5 (852.4-1140.2) | 0.36 (0.28 to 0.44) |
| Central African Republic | 1568.8 (1124.7-2172) | 830.9 (595.7-1150.3) | 3385.7 (2397.6-4672.5) | 946.5 (670.3-1306.3) | 0.37 (0.32 to 0.41) |
| Central Asia | 80812 (75338.1-85944.5) | 1010.4 (942-1074.6) | 113379.9 (101552-126139.1) | 779.3 (698-866.9) | -0.51 (-0.6 to -0.42) |
| Central Europe | 303146.8 (288850.8-317172.3) | 1143.1 (1089.2-1196) | 451024.3 (415893.5-485436.5) | 1218.1 (1123.2-1311) | 0.06 (-0.05 to 0.17) |
| Central Latin America | 77284.8 (74506.1-79967) | 569.5 (549-589.3) | 317858.7 (280793.2-357682) | 743.2 (656.6-836.4) | 0.75 (0.65 to 0.84) |
| Central Sub-Saharan Africa | 28168.8 (19664.3-37938) | 749.1 (522.9-1008.9) | 88574.1 (64052.8-118179.6) | 981.6 (709.8-1309.7) | 0.89 (0.78 to 1.01) |
| Chad | 2107 (1531.3-2782.1) | 454.7 (330.4-600.4) | 5175.1 (3757.4-6943.4) | 557.3 (404.7-747.8) | 0.64 (0.6 to 0.69) |
| Chile | 15094.8 (13769.3-16514.4) | 897.6 (818.8-982) | 30300.2 (26784.7-33867) | 676.7 (598.2-756.4) | -0.74 (-0.83 to -0.64) |
| China | 578156.7 (479001.7-697816.4) | 402.8 (333.8-486.2) | 1616381.5 (1275221.6-2014511.7) | 426.5 (336.5-531.6) | 0.06 (-0.01 to 0.12) |
| Colombia | 20563.5 (19009.9-22201.2) | 714.3 (660.3-771.1) | 71830.3 (60049.6-85951.2) | 751.1 (627.9-898.7) | 0.06 (-0.15 to 0.26) |
| Comoros | 269.4 (196.5-362.4) | 830.1 (605.4-1116.7) | 922.8 (671.6-1230) | 1141.7 (830.9-1521.7) | 0.9 (0.83 to 0.97) |
| Congo | 2129.1 (1247.8-3394) | 1196.1 (701-1906.6) | 6032.4 (3745.1-9240.9) | 1388.3 (861.9-2126.7) | 0.42 (0.33 to 0.51) |
| Cook Islands | 34.1 (26.3-43.8) | 1599.1 (1234.3-2050.9) | 83 (64.3-105.1) | 1761.4 (1364.7-2229.3) | 0.44 (0.31 to 0.57) |
| Costa Rica | 1784.1 (1618.4-1952.4) | 627.3 (569.1-686.5) | 8502.8 (7310.5-9815.2) | 888.1 (763.5-1025.1) | 1.25 (1.1 to 1.4) |
| Croatia | 14832.1 (12908.2-16900.7) | 1332.4 (1159.6-1518.2) | 17136 (14615.5-19808.8) | 1149 (980-1328.3) | -0.35 (-0.54 to -0.16) |
| Cuba | 15473.9 (14158.1-16650.6) | 913.1 (835.4-982.5) | 32637.4 (27752.5-38378.6) | 955.1 (812.2-1123.2) | 0.29 (0.14 to 0.44) |
| Cyprus | 1780.3 (1425.3-2158.1) | 1274.5 (1020.4-1544.9) | 3947.7 (3223.1-4796.5) | 1129.5 (922.2-1372.4) | -0.23 (-0.42 to -0.04) |
| Czechia | 34502.8 (31080.3-38560.6) | 1457.1 (1312.6-1628.5) | 34861.3 (29157.1-41019.3) | 997.9 (834.6-1174.2) | -1.62 (-1.8 to -1.44) |
| C么te d'Ivoire | 5373.7 (4205.1-6759.7) | 838.4 (656.1-1054.7) | 19967.5 (14627.3-27595.8) | 1113.5 (815.7-1538.9) | 1 (0.89 to 1.11) |
| Democratic People's Republic of Korea | 13590.6 (8633.5-20272.2) | 510.7 (324.4-761.7) | 30680.1 (21480.5-40826.6) | 544.4 (381.1-724.4) | 0.4 (0.31 to 0.49) |
| Democratic Republic of the Congo | 19136.1 (13048.8-26492.4) | 724.6 (494.1-1003.2) | 57069.1 (39493.9-80322.2) | 944.9 (653.9-1329.9) | 0.91 (0.75 to 1.07) |
| Denmark | 29551.8 (27572.5-31471) | 2265.1 (2113.4-2412.2) | 22579.6 (19889.2-25069.6) | 1172.8 (1033.1-1302.2) | -2.2 (-2.33 to -2.07) |
| Djibouti | 186.3 (138-251.9) | 868.3 (643.1-1174.3) | 1029.9 (709.3-1465.8) | 993.1 (684-1413.4) | 0.33 (0.28 to 0.39) |
| Dominica | 161.4 (131.3-191.9) | 1643.7 (1337.1-1955.1) | 226.9 (171.5-290.3) | 1504.2 (1136.9-1924.9) | -0.32 (-0.41 to -0.23) |
| Dominican Republic | 3002 (2512.4-3563.8) | 491 (410.9-582.9) | 11014.9 (8387.6-14404.7) | 659.2 (501.9-862) | 1.17 (1.01 to 1.34) |
| East Asia | 602115.3 (502721.4-723229.4) | 404.2 (337.5-485.5) | 1698475.2 (1357058.8-2096766.9) | 433.2 (346.1-534.7) | 0.11 (0.06 to 0.17) |
| Eastern Europe | 508760 (488572-528043) | 1040.6 (999.3-1080) | 689942.1 (619587.2-777994.5) | 1111.4 (998.1-1253.2) | -0.2 (-0.43 to 0.03) |
| Eastern Sub-Saharan Africa | 97173.9 (82120.7-116805.4) | 798.8 (675-960.1) | 270974.1 (233726.9-316917) | 1002.2 (864.5-1172.1) | 0.67 (0.58 to 0.75) |
| Ecuador | 3486.2 (3221.3-3746.4) | 405.4 (374.6-435.7) | 17319.6 (13542.9-21586.7) | 625.8 (489.4-780) | 1.53 (1.32 to 1.73) |
| Egypt | 16340.7 (14296.2-19181.3) | 365.4 (319.7-429) | 99052.5 (80161.9-123251.4) | 895.1 (724.4-1113.8) | 3.56 (3.07 to 4.04) |
| El Salvador | 1832.3 (1615.4-2059.3) | 374.7 (330.4-421.2) | 6572.2 (5272.7-8042.6) | 642.7 (515.6-786.5) | 1.71 (1.59 to 1.84) |
| Equatorial Guinea | 246.4 (162.8-356.6) | 771.9 (510-1117.2) | 1059.7 (630.6-1617.6) | 1339.8 (797.2-2045.1) | 1.95 (1.86 to 2.05) |
| Eritrea | 1721 (1228.2-2413.4) | 973.8 (695-1365.7) | 5530.2 (3898.4-7457.3) | 1255.1 (884.8-1692.5) | 0.87 (0.79 to 0.94) |
| Estonia | 4442.3 (4099.1-4800) | 1239.2 (1143.5-1339) | 4336.8 (3538.3-5128.3) | 991.3 (808.8-1172.2) | -0.87 (-0.99 to -0.76) |
| Eswatini | 398.3 (293.4-529.9) | 882.1 (649.7-1173.5) | 1320.1 (773.3-2063.7) | 1458.1 (854.1-2279.3) | 1.84 (1.48 to 2.2) |
| Ethiopia | 27065.7 (18592-38885.9) | 842.4 (578.7-1210.3) | 60028.6 (49484-73972.4) | 876.9 (722.9-1080.6) | -0.01 (-0.18 to 0.17) |
| Fiji | 804.4 (609.1-1054.4) | 1427.1 (1080.6-1870.7) | 2441 (1749.4-3195.3) | 1785.1 (1279.4-2336.8) | 0.74 (0.63 to 0.86) |
| Finland | 15381.1 (14224.8-16588.5) | 1295.4 (1198-1397.1) | 18994.7 (16503.4-21264.6) | 943.8 (820-1056.5) | -0.93 (-0.99 to -0.87) |
| France | 222956.4 (206553.2-238706.8) | 1602.1 (1484.2-1715.3) | 262480 (223108.8-292311.4) | 1187.1 (1009.1-1322.1) | -0.99 (-1.09 to -0.89) |
| Gabon | 1085 (749.5-1464) | 1108.2 (765.5-1495.4) | 2333 (1611.3-3289.2) | 1305.2 (901.4-1840.1) | 0.25 (0.09 to 0.4) |
| Gambia | 170.6 (131.5-222) | 308 (237.4-400.8) | 786.2 (561.2-1034.4) | 508.4 (362.9-668.9) | 1.46 (1.28 to 1.64) |
| Georgia | 15876.4 (14192.9-17690.8) | 1429.3 (1277.8-1592.7) | 18170.5 (15491-21064) | 1730.7 (1475.5-2006.3) | 1.08 (0.74 to 1.41) |
| Germany | 354888.5 (327790.1-383564.8) | 1691.3 (1562.2-1828) | 365714.1 (316499.2-405970.9) | 1160.5 (1004.3-1288.2) | -1.16 (-1.22 to -1.11) |
| Ghana | 8216.9 (6313.8-10562.8) | 804.6 (618.3-1034.3) | 28271.3 (20638.6-37537.7) | 1036.7 (756.8-1376.4) | 0.72 (0.67 to 0.78) |
| Global | 5651007.3 (5336459.5-5939102.4) | 841.6 (794.8-884.6) | 11058137.8 (10223544.4-11869597.9) | 744.2 (688-798.8) | -0.48 (-0.52 to -0.45) |
| Greece | 36090.6 (33566.5-38471.8) | 1349.9 (1255.4-1438.9) | 47494.6 (41919.8-52294.4) | 1292.2 (1140.5-1422.8) | -0.49 (-0.66 to -0.32) |
| Greenland | 91 (71.3-115.4) | 1651.6 (1294.4-2094.4) | 109.8 (81.1-147.4) | 808.6 (597.6-1085.6) | -2.61 (-2.81 to -2.42) |
| Grenada | 163.3 (145.4-183.1) | 1354.1 (1205.7-1518) | 321.6 (279.3-365.4) | 1600.9 (1390.5-1818.9) | 0.69 (0.4 to 0.98) |
| Guam | 99 (79.6-121.9) | 767.9 (617.6-945.4) | 223.3 (172.8-287.1) | 592.5 (458.4-761.8) | -0.24 (-0.5 to 0.01) |
| Guatemala | 1515.2 (1375.5-1655.4) | 263.5 (239.2-287.8) | 7902.3 (6735.4-9309.3) | 431.6 (367.9-508.5) | 1.84 (1.57 to 2.12) |
| Guinea | 3496 (2570.4-4485) | 631.7 (464.5-810.4) | 7565.6 (5346.3-10358.8) | 828.1 (585.2-1133.9) | 0.79 (0.74 to 0.85) |
| Guinea-Bissau | 455.3 (311.1-667.4) | 713.2 (487.4-1045.5) | 1124.2 (750.7-1575) | 978.7 (653.5-1371.1) | 1 (0.95 to 1.05) |
| Guyana | 626.9 (541.3-722.4) | 1022.7 (883-1178.6) | 1440.9 (1097.8-1851.3) | 1279.5 (974.8-1643.9) | 1.01 (0.81 to 1.22) |
| Haiti | 5469.9 (3256.6-8864.8) | 1014.2 (603.8-1643.6) | 14696.1 (8918.1-22801.2) | 1240.1 (752.5-1924) | 0.74 (0.66 to 0.83) |
| High SDI | 2520402 (2373430.1-2629587.5) | 1351.7 (1272.9-1410.2) | 3123806.8 (2806713.6-3354206.5) | 905.4 (813.5-972.2) | -1.35 (-1.38 to -1.32) |
| High-income Asia Pacific | 122091.2 (113908.3-129154.9) | 349.1 (325.7-369.3) | 356019.4 (302298.3-393036.1) | 505 (428.8-557.5) | 1.36 (1.23 to 1.48) |
| High-income North America | 979938.3 (915678.2-1031896.8) | 1691.6 (1580.7-1781.3) | 1148536.6 (1047450.1-1236042) | 1020.6 (930.8-1098.4) | -1.82 (-1.88 to -1.77) |
| High-middle SDI | 1553895.4 (1469471.1-1625043.7) | 900.7 (851.7-941.9) | 2635532.5 (2412932.3-2891873.8) | 760.2 (696-834.2) | -0.7 (-0.8 to -0.6) |
| Honduras | 1203 (761.8-1720.7) | 360.9 (228.6-516.2) | 7432.1 (5335.9-10476.5) | 706.7 (507.4-996.2) | 2.25 (2.07 to 2.43) |
| Hungary | 37199.4 (32903.1-42220.3) | 1444.2 (1277.4-1639.2) | 39953.3 (34277.9-46416.8) | 1256.3 (1077.9-1459.6) | -0.76 (-0.92 to -0.59) |
| Iceland | 717.7 (649.6-786.5) | 1504.1 (1361.4-1648.3) | 945 (805.1-1067.3) | 970.4 (826.8-1095.9) | -1.44 (-1.6 to -1.29) |
| India | 288534.4 (241962-340788.7) | 376.3 (315.6-444.5) | 1209913.3 (1000994.9-1457842.2) | 601.8 (497.9-725.1) | 1.49 (1.33 to 1.66) |
| Indonesia | 104947.2 (68226.3-152812.4) | 649.6 (422.3-945.9) | 383569 (245354.6-566740.3) | 915.9 (585.9-1353.3) | 1.01 (0.91 to 1.11) |
| Iran (Islamic Republic of) | 12233.6 (10450.5-14217.2) | 265.4 (226.7-308.4) | 59865.4 (52954.3-66729.3) | 460.8 (407.6-513.7) | 2.4 (2.1 to 2.7) |
| Iraq | 6822.9 (5133.5-9038.1) | 540.5 (406.7-716) | 31098.4 (21601.9-41834.6) | 800.4 (556-1076.8) | 1.25 (1.15 to 1.34) |
| Ireland | 12108.5 (11344.9-13060.7) | 1778.8 (1666.6-1918.7) | 12961.1 (11198.9-14662.5) | 987.6 (853.3-1117.3) | -1.75 (-1.86 to -1.64) |
| Israel | 12775.9 (11577.9-14072.6) | 1576.2 (1428.4-1736.2) | 21004.5 (17955-23765.5) | 1051 (898.4-1189.1) | -1.87 (-2.14 to -1.59) |
| Italy | 233165.7 (217575.1-244504.5) | 1532.1 (1429.6-1606.6) | 246865 (210096.1-270946.7) | 1086.7 (924.8-1192.7) | -1.18 (-1.24 to -1.13) |
| Jamaica | 3064.3 (2790.1-3375.5) | 1040.4 (947.3-1146.1) | 7188.3 (5410.1-8996.7) | 1359.1 (1022.9-1701.1) | 0.74 (0.47 to 1.02) |
| Japan | 107135 (100127.8-113400.5) | 361.8 (338.1-382.9) | 297530.2 (251016.5-329964.4) | 570 (480.9-632.1) | 1.62 (1.51 to 1.73) |
| Jordan | 1460.5 (1107-1847.5) | 679.2 (514.9-859.3) | 9723.5 (6882.9-13368.7) | 773.4 (547.5-1063.3) | 0.61 (0.23 to 0.99) |
| Kazakhstan | 25701 (22312.3-29588.3) | 1228.9 (1066.9-1414.8) | 24553.9 (20178.9-28834.1) | 773.7 (635.9-908.6) | -0.72 (-1.09 to -0.35) |
| Kenya | 8144 (5834-11069.6) | 611.1 (437.7-830.6) | 37088.8 (26495.6-50863.6) | 991.5 (708.3-1359.7) | 1.75 (1.66 to 1.85) |
| Kiribati | 67.4 (49.6-89.6) | 1130.7 (832.9-1503.9) | 192.3 (140.5-259.5) | 1529.2 (1117.2-2063.1) | 1.05 (0.96 to 1.15) |
| Kuwait | 441.1 (388.1-493.8) | 482.4 (424.4-540) | 2011.3 (1660.1-2383.1) | 431.4 (356.1-511.2) | 0.25 (-0.28 to 0.79) |
| Kyrgyzstan | 4915.5 (4439.4-5423.7) | 954.6 (862.2-1053.3) | 5590.5 (4617.4-6594) | 661 (546-779.7) | -1.28 (-1.54 to -1.01) |
| Lao People's Democratic Republic | 2282 (1292.7-3806.5) | 665.4 (377-1110) | 6759.4 (4593.8-9835) | 874.3 (594.2-1272.1) | 0.97 (0.91 to 1.02) |
| Latvia | 7228.4 (6421.9-8096.9) | 1156.1 (1027.1-1295) | 7690.9 (6356.9-9052.4) | 1168.8 (966.1-1375.7) | 0.14 (-0.05 to 0.33) |
| Lebanon | 3646.1 (2671.5-4899.7) | 961.7 (704.6-1292.4) | 11218.5 (8998.3-13924.9) | 1144.7 (918.2-1420.9) | 0.81 (0.61 to 1.02) |
| Lesotho | 1043.3 (766.6-1434.3) | 746.1 (548.2-1025.6) | 2693.6 (1707-3982) | 1491.8 (945.4-2205.5) | 2.81 (2.43 to 3.2) |
| Liberia | 1040.4 (816.6-1296.6) | 552.1 (433.3-688.1) | 2514.5 (1787.6-3480) | 761.7 (541.5-1054.2) | 1.17 (1.05 to 1.29) |
| Libya | 1233 (976-1532.6) | 396.4 (313.8-492.8) | 5382.4 (4144.6-7082.6) | 643.6 (495.6-846.9) | 1.79 (1.6 to 1.99) |
| Lithuania | 8237.5 (7443.4-9080) | 1039.5 (939.3-1145.8) | 10175.7 (8460-11958.5) | 1051.4 (874.2-1235.7) | 0.07 (-0.16 to 0.3) |
| Low SDI | 218896.7 (187649.9-252566.4) | 586.7 (503-677) | 632243.6 (555268.7-710934.2) | 770.5 (676.7-866.4) | 0.84 (0.73 to 0.95) |
| Low-middle SDI | 447057.2 (392542-508982.4) | 443.5 (389.4-504.9) | 1682190.1 (1496560.8-1855806.7) | 697.8 (620.8-769.8) | 1.48 (1.44 to 1.53) |
| Luxembourg | 1722.4 (1596-1863.1) | 1844.4 (1709.1-1995.1) | 1759 (1556-1980) | 989.9 (875.6-1114.2) | -1.61 (-1.81 to -1.42) |
| Madagascar | 6538.9 (5174.7-8253.9) | 773.9 (612.5-976.9) | 14844.5 (10279.1-19929.9) | 803.8 (556.6-1079.2) | 0.04 (-0.14 to 0.22) |
| Malawi | 4406.7 (3369.3-5589.8) | 700.6 (535.7-888.7) | 11573.7 (8494-15262.3) | 989.9 (726.5-1305.4) | 1.07 (1.02 to 1.13) |
| Malaysia | 14197.3 (11792.8-16738.9) | 961.9 (799-1134.2) | 61556.8 (52263.9-71589) | 1255.5 (1066-1460.2) | 0.78 (0.67 to 0.89) |
| Maldives | 59.3 (34.3-96.3) | 386.7 (223.8-628.2) | 203.8 (158.5-256.3) | 374.6 (291.3-471.1) | -0.5 (-0.8 to -0.2) |
| Mali | 4942.4 (3815.3-6088.2) | 739.2 (570.6-910.5) | 12319.2 (8728.4-17171.3) | 851.1 (603-1186.4) | 0.4 (0.32 to 0.48) |
| Malta | 1433.9 (1308.7-1574) | 1979.2 (1806.5-2172.7) | 1765.6 (1534.4-2013.9) | 1118.9 (972.4-1276.2) | -2.25 (-2.45 to -2.05) |
| Marshall Islands | 23 (15.7-33.7) | 901.6 (616.4-1320.2) | 74.6 (45.2-116) | 1272.5 (770.8-1980.2) | 0.98 (0.9 to 1.06) |
| Mauritania | 1296.2 (903.2-1746.2) | 780.8 (544.1-1051.9) | 3186 (2359.9-4261.5) | 888.1 (657.8-1187.8) | 0.24 (0.1 to 0.38) |
| Mauritius | 760.6 (692.2-826) | 625.3 (569.1-679.1) | 3928.9 (3541.7-4262.1) | 1161.2 (1046.8-1259.7) | 1.71 (1.38 to 2.04) |
| Mexico | 37485.8 (36145.8-38714.6) | 539.2 (519.9-556.8) | 152371.3 (129297-177694.8) | 707 (600-824.6) | 0.67 (0.57 to 0.77) |
| Micronesia (Federated States of) | 93 (63.1-131.3) | 1168.4 (792.8-1648.7) | 201.8 (145.7-278) | 1533.2 (1107.3-2113) | 0.82 (0.73 to 0.9) |
| Middle SDI | 902465 (826137.9-992495.3) | 520 (476-571.8) | 2970652.2 (2666905.1-3318540.9) | 632.2 (567.6-706.3) | 0.52 (0.47 to 0.57) |
| Monaco | 210.6 (150-276.8) | 1900.7 (1353.7-2498) | 305.4 (230.8-394.7) | 1952.3 (1475.5-2523.1) | 0.2 (0.11 to 0.29) |
| Mongolia | 484.2 (360.5-623.7) | 279.4 (208-360) | 1190.4 (904.9-1516.4) | 301.5 (229.2-384.1) | -0.01 (-0.17 to 0.16) |
| Montenegro | 1342.3 (1013.3-1733.6) | 1232.2 (930.1-1591.3) | 2540.7 (1989.5-3223.6) | 1457.1 (1140.9-1848.7) | 0.66 (0.54 to 0.78) |
| Morocco | 6076.5 (4740.5-7728.3) | 259.1 (202.2-329.6) | 25974.4 (17916.3-35988.9) | 432.3 (298.2-599) | 1.9 (1.8 to 2) |
| Mozambique | 6698.2 (5422.1-8130.3) | 685.5 (554.9-832) | 18646.3 (12861.4-24693.5) | 1042.2 (718.9-1380.2) | 1.65 (1.54 to 1.76) |
| Myanmar | 29870.7 (20712.1-42494) | 763.1 (529.1-1085.6) | 77032.9 (59892.9-104102) | 912.5 (709.5-1233.1) | 0.53 (0.47 to 0.58) |
| Namibia | 1057.3 (841-1340) | 974.4 (775-1234.9) | 4103.2 (2658.1-5680) | 1816 (1176.4-2513.9) | 2.46 (2.3 to 2.61) |
| Nauru | 8.8 (5.3-13.6) | 1212.7 (727.5-1886) | 17.1 (10.4-26.4) | 1852.9 (1124.3-2856.4) | 1.52 (1.46 to 1.59) |
| Nepal | 5173.4 (3747.2-6817.1) | 336.1 (243.4-442.9) | 16343 (11355.9-23698.2) | 412.9 (286.9-598.8) | 0.74 (0.47 to 1) |
| Netherlands | 62792 (57627.9-67809.8) | 1896.5 (1740.5-2048) | 66917.9 (58918.4-74941.8) | 1143.7 (1007-1280.9) | -1.84 (-1.96 to -1.73) |
| New Zealand | 11615.9 (10730.6-12541.8) | 1765.4 (1630.9-1906.1) | 13563.8 (11816.3-15059.6) | 950.9 (828.4-1055.8) | -1.91 (-1.99 to -1.84) |
| Nicaragua | 714.6 (607.8-841.7) | 287.6 (244.6-338.7) | 3999.6 (3168.3-5105.1) | 491.3 (389.2-627.1) | 2.12 (1.92 to 2.33) |
| Niger | 1742.1 (1296.7-2333.2) | 396.4 (295-530.9) | 6577.9 (4758.9-9035.7) | 472.5 (341.8-649) | 0.53 (0.47 to 0.59) |
| Nigeria | 64378.5 (49092.1-82134.9) | 883.7 (673.9-1127.4) | 242106.3 (169226.7-342741) | 1613.9 (1128.1-2284.7) | 2.23 (1.93 to 2.52) |
| Niue | 5 (3.9-6.4) | 1386 (1068.2-1767.2) | 6.1 (4.6-8) | 1560.9 (1166.3-2032.8) | 0.39 (0.32 to 0.47) |
| North Africa and Middle East | 94200.2 (85510.9-104628) | 333.3 (302.5-370.2) | 448276.5 (396203.3-508751.5) | 588 (519.7-667.4) | 2.29 (2.06 to 2.53) |
| North Macedonia | 3680.8 (3068.3-4329.2) | 1118.9 (932.7-1316) | 7831.4 (6161.3-9768.6) | 1320.9 (1039.2-1647.6) | 0.67 (0.46 to 0.88) |
| Northern Mariana Islands | 24.3 (18-31.9) | 993.4 (737.8-1304.8) | 125.3 (102.3-151) | 1289.4 (1052.5-1553.7) | 0.61 (0.43 to 0.79) |
| Norway | 14631.2 (13655-15479.7) | 1351.1 (1260.9-1429.4) | 12613.5 (11180.4-13772.8) | 777.8 (689.4-849.3) | -1.92 (-2.07 to -1.77) |
| Oceania | 4010.2 (3144.5-5145.8) | 833.6 (653.6-1069.6) | 11854.6 (9578.5-14496.7) | 960.5 (776.1-1174.6) | 0.45 (0.4 to 0.5) |
| Oman | 216.2 (154.1-297) | 215.1 (153.3-295.5) | 758.2 (566.7-987.4) | 242.7 (181.4-316.1) | 0.73 (0.37 to 1.09) |
| Pakistan | 76163.8 (58250.8-97031) | 835.2 (638.8-1064) | 261690.3 (191983.4-344903.1) | 1319.9 (968.3-1739.7) | 1.16 (0.92 to 1.4) |
| Palau | 31.4 (24-40.8) | 1963.6 (1497.9-2551.9) | 75 (56.4-98) | 1790.4 (1346.6-2339.5) | -0.4 (-0.46 to -0.35) |
| Palestine | 1337.1 (981.4-1820) | 921.2 (676.1-1253.9) | 4742.5 (3807.5-5805.6) | 1102.2 (884.9-1349.3) | 0.8 (0.65 to 0.95) |
| Panama | 1346.7 (1237-1478.5) | 555.8 (510.6-610.2) | 5269 (4140.2-6446) | 708.6 (556.8-866.9) | 0.96 (0.8 to 1.12) |
| Papua New Guinea | 2059.3 (1453.2-3001.6) | 696 (491.2-1014.5) | 6474.3 (4686.3-8945.7) | 788.3 (570.6-1089.2) | 0.33 (0.24 to 0.42) |
| Paraguay | 2150.3 (1717-2647.7) | 592.8 (473.3-729.9) | 8979.6 (6612.1-11799.2) | 906.9 (667.8-1191.7) | 1.47 (1.28 to 1.66) |
| Peru | 11300.3 (9067.4-13825.8) | 573.1 (459.9-701.2) | 32878.3 (23442.1-44533.7) | 587.8 (419.1-796.2) | -0.35 (-0.56 to -0.15) |
| Philippines | 40924.3 (36328.8-45938.4) | 857.2 (761-962.3) | 164725.5 (132629-204165.1) | 1181.2 (951.1-1464.1) | 0.98 (0.89 to 1.07) |
| Poland | 85336.3 (81430.5-88626.7) | 1107.4 (1056.7-1150.1) | 150268.7 (131921.3-167701.1) | 1240.3 (1088.9-1384.2) | 0.17 (0.03 to 0.3) |
| Portugal | 30797.7 (28498.7-33155.1) | 1263.3 (1169-1359.9) | 35009.8 (30392.4-38546.9) | 899.8 (781.2-990.8) | -1.18 (-1.24 to -1.13) |
| Puerto Rico | 5700.5 (5252-6184.1) | 939.5 (865.6-1019.2) | 10575.3 (8755.9-12428) | 905.9 (750.1-1064.6) | -0.16 (-0.31 to -0.02) |
| Qatar | 127.9 (96.8-163.9) | 782.9 (592.4-1003.6) | 1190.3 (854.1-1637.7) | 777 (557.5-1069.1) | -0.29 (-0.51 to -0.08) |
| Republic of Korea | 12132 (10583.3-14362.6) | 243.8 (212.7-288.6) | 48583.7 (38506.1-58930.9) | 290.5 (230.2-352.4) | 0.68 (0.61 to 0.75) |
| Republic of Moldova | 9243.8 (8228-10231.4) | 1197.4 (1065.8-1325.3) | 11919.3 (10207.5-14102.5) | 1128.9 (966.8-1335.7) | 0.43 (0.12 to 0.75) |
| Romania | 44814.7 (41485.8-48536.3) | 895.6 (829.1-970) | 73992.4 (64488.6-83750) | 1232.1 (1073.9-1394.6) | 0.88 (0.74 to 1.02) |
| Russian Federation | 294306.5 (284031-304430.5) | 932.4 (899.9-964.5) | 482210.8 (430582-534807.7) | 1134.5 (1013-1258.2) | 0.22 (-0.07 to 0.52) |
| Rwanda | 5578.9 (4044.1-7756.6) | 1192.2 (864.2-1657.6) | 12380.9 (8810.8-17013.9) | 1170.8 (833.2-1608.9) | -0.52 (-0.71 to -0.32) |
| Saint Kitts and Nevis | 138.3 (126.3-151.4) | 2163.5 (1975.4-2367.8) | 197.9 (164-235.5) | 1536.7 (1273.2-1828.6) | -0.46 (-0.68 to -0.23) |
| Saint Lucia | 206.1 (189.2-223.9) | 1408.5 (1293.5-1530.1) | 456.7 (380.9-550) | 1085.2 (905.2-1306.9) | -1.35 (-1.68 to -1.02) |
| Saint Vincent and the Grenadines | 170.4 (152.6-188) | 1418.5 (1269.9-1564.8) | 334.7 (289.1-381.1) | 1316.5 (1136.9-1498.8) | -0.37 (-0.65 to -0.09) |
| Samoa | 118.9 (89.5-152) | 825 (621.1-1055) | 248.8 (186.3-333.6) | 1010.7 (756.7-1355) | 0.6 (0.54 to 0.65) |
| San Marino | 66.7 (51.4-84.2) | 1136.2 (874.7-1433.4) | 74 (46.2-107.9) | 625.5 (391-912.1) | -1.29 (-1.55 to -1.02) |
| Sao Tome and Principe | 60.3 (46.6-77.6) | 538.9 (416.4-693) | 154.3 (112.1-206.1) | 843.7 (613-1127.1) | 1.45 (1.39 to 1.51) |
| Saudi Arabia | 2169.7 (1635.2-2787.3) | 243.5 (183.5-312.9) | 11113.3 (8574.5-14371.2) | 369.1 (284.8-477.3) | 1.17 (0.86 to 1.49) |
| Senegal | 2898 (2295.3-3784.3) | 548.4 (434.4-716.1) | 10653.9 (7915.3-14230.7) | 825.1 (613-1102.2) | 1.37 (1.25 to 1.49) |
| Serbia | 28818.4 (21803.7-37016.1) | 1385.7 (1048.4-1779.8) | 43903.7 (33144.9-56410.2) | 1565.1 (1181.5-2010.9) | 0.22 (0.1 to 0.34) |
| Seychelles | 74.6 (63.7-86.6) | 777.9 (663.8-902.5) | 219.1 (184.1-257.5) | 1056.1 (887.8-1241.3) | 0.77 (0.39 to 1.15) |
| Sierra Leone | 1607.2 (1165.9-2096.1) | 484.9 (351.7-632.4) | 4185.2 (3066.9-5610.9) | 692.8 (507.7-928.8) | 1.4 (1.31 to 1.5) |
| Singapore | 2700.8 (2480.1-2947.2) | 744.9 (684-812.9) | 9372.7 (8382.1-10478.6) | 617.1 (551.9-689.9) | -0.2 (-0.38 to -0.03) |
| Slovakia | 12188.5 (10330.5-14472.1) | 1174.8 (995.8-1395) | 19685.6 (14749.3-25222.5) | 1200.1 (899.2-1537.7) | -0.1 (-0.23 to 0.02) |
| Slovenia | 5660.7 (5171.7-6157) | 1314.3 (1200.8-1429.5) | 7267.7 (6017.6-8761.6) | 992.6 (821.9-1196.6) | -1.13 (-1.34 to -0.92) |
| Solomon Islands | 122 (79.1-185.2) | 539.5 (349.8-819) | 485.8 (329.2-684.9) | 895.5 (606.9-1262.7) | 1.63 (1.46 to 1.81) |
| Somalia | 2603.3 (1775.9-3574.1) | 723 (493.2-992.6) | 7387.8 (4983.9-10008.6) | 783.4 (528.5-1061.3) | 0.19 (0.15 to 0.23) |
| South Africa | 27535.1 (20435.4-34717.2) | 815.7 (605.4-1028.5) | 101057.2 (91528.1-111337.8) | 1281.1 (1160.3-1411.4) | 1.63 (1.39 to 1.87) |
| South Asia | 383662.8 (333279.3-434886.4) | 404.1 (351-458.1) | 1540232.5 (1329846.8-1795876) | 620.3 (535.6-723.3) | 1.26 (1.14 to 1.39) |
| South Sudan | 2582 (1828.8-3715.5) | 622.6 (441-895.9) | 4746.6 (3183.8-6719.9) | 762.2 (511.3-1079.1) | 0.58 (0.41 to 0.75) |
| Southeast Asia | 267844.1 (220703.1-328256.6) | 632.6 (521.2-775.3) | 1008167.7 (832151.4-1232733.9) | 880.1 (726.4-1076.1) | 1.06 (0.98 to 1.14) |
| Southern Latin America | 120408.9 (112995.4-127078.2) | 1520 (1426.4-1604.2) | 169157.1 (153380.4-183184.1) | 1149.5 (1042.3-1244.8) | -0.82 (-0.97 to -0.66) |
| Southern Sub-Saharan Africa | 36093.1 (28830.4-43831.4) | 815.7 (651.6-990.6) | 130034.9 (118552.3-142138.4) | 1335.7 (1217.8-1460) | 1.89 (1.62 to 2.16) |
| Spain | 117614.4 (107004-127364.4) | 1230.2 (1119.2-1332.2) | 119983.4 (103103.3-134731.6) | 775.3 (666.3-870.6) | -1.55 (-1.6 to -1.49) |
| Sri Lanka | 8579.1 (7012.2-10498.2) | 482 (394-589.9) | 31283.1 (20606.8-42829.3) | 649.5 (427.9-889.3) | 1.23 (1.01 to 1.46) |
| Sudan | 3421.4 (2293.6-4861) | 230.1 (154.2-326.9) | 10498.7 (7017.6-15210.8) | 337.2 (225.4-488.5) | 1.38 (1.22 to 1.55) |
| Suriname | 354.5 (299.9-413.6) | 814.6 (689.3-950.5) | 1001.3 (761.1-1272.8) | 896.4 (681.3-1139.5) | 0.62 (0.43 to 0.81) |
| Sweden | 29129.9 (26420.3-31833.5) | 1227 (1112.9-1340.9) | 27935.5 (23245.9-32071.4) | 829.8 (690.5-952.7) | -0.92 (-1.16 to -0.68) |
| Switzerland | 24577.5 (22262.4-26543.8) | 1455 (1318-1571.4) | 26149.3 (22069.6-29343.3) | 892.2 (753-1001.2) | -1.55 (-1.73 to -1.36) |
| Syrian Arab Republic | 3251.7 (2519.1-4133.3) | 368.8 (285.7-468.8) | 12058.6 (8692.4-16507.1) | 510.4 (367.9-698.7) | 0.9 (0.77 to 1.03) |
| Taiwan (Province of China) | 10367.9 (9619.3-11175.8) | 373.8 (346.8-402.9) | 51413.6 (45477.4-57387.8) | 682.8 (604-762.1) | 2.16 (1.89 to 2.44) |
| Tajikistan | 3186.9 (2567.6-3886.7) | 680.5 (548.3-829.9) | 5423 (3627.3-7655.6) | 528 (353.1-745.3) | -0.84 (-1.01 to -0.68) |
| Thailand | 29851.2 (22915.2-36669.9) | 503 (386.2-617.9) | 151302.5 (113010.8-192391.1) | 778.4 (581.4-989.8) | 1.6 (1.31 to 1.89) |
| Timor-Leste | 213.3 (126.8-339.3) | 490.8 (291.7-780.6) | 919.3 (606.4-1314.9) | 647.3 (426.9-925.8) | 1.13 (0.99 to 1.28) |
| Togo | 1260.8 (1003.2-1599.4) | 646.3 (514.3-819.9) | 5654.5 (4006.2-7781.8) | 905.2 (641.3-1245.7) | 1.1 (1.06 to 1.13) |
| Tokelau | 2.7 (1.8-3.9) | 1161.3 (764.7-1657.9) | 3.5 (2.5-4.7) | 1398.4 (1005.7-1871.6) | 0.68 (0.57 to 0.79) |
| Tonga | 148.8 (117.1-185.5) | 1568 (1234.3-1954.9) | 241 (177.7-318.6) | 1794.1 (1323.4-2372.5) | 0.4 (0.33 to 0.47) |
| Trinidad and Tobago | 1814.4 (1662.4-1982.9) | 1321.5 (1210.8-1444.2) | 4438.5 (3392.4-5576.1) | 1274.2 (973.9-1600.8) | -0.05 (-0.24 to 0.14) |
| Tropical Latin America | 124198 (117839.9-129755) | 820.3 (778.3-857) | 386003.8 (353407.8-410823.3) | 871.4 (797.8-927.4) | 0 (-0.07 to 0.07) |
| Tunisia | 3844.7 (3069.1-4705.9) | 441.6 (352.5-540.5) | 13023.4 (9324.8-18101.9) | 557.2 (399-774.5) | 0.58 (0.48 to 0.68) |
| Turkey | 17672.3 (13842.8-22099.9) | 295.8 (231.7-369.9) | 106183.5 (80639.5-135262.6) | 642.7 (488.1-818.7) | 3.43 (2.77 to 4.1) |
| Turkmenistan | 2250.6 (1969.9-2552.1) | 693 (606.5-785.8) | 4495.2 (3384.6-5933.8) | 635.2 (478.3-838.5) | 0.02 (-0.51 to 0.55) |
| Tuvalu | 12 (7.7-17.8) | 1036.8 (661.2-1533.6) | 23.9 (17.2-32.4) | 1310.6 (942.3-1777.7) | 0.74 (0.63 to 0.85) |
| Uganda | 10201.6 (7593.2-13476.7) | 975.4 (726-1288.5) | 34662.3 (25658.1-46437.7) | 1461.6 (1081.9-1958.2) | 0.9 (0.65 to 1.14) |
| Ukraine | 163532.5 (149242.4-177854.1) | 1311.7 (1197.1-1426.6) | 147305.2 (98193.2-209775.4) | 1084.9 (723.2-1545) | -1.07 (-1.28 to -0.86) |
| United Arab Emirates | 582.7 (437.1-757.5) | 1038.2 (778.9-1349.7) | 4216.2 (2905.4-5829.1) | 587.2 (404.6-811.8) | -0.7 (-1.31 to -0.09) |
| United Kingdom | 306268.1 (292074.2-316813.8) | 2062 (1966.4-2133) | 234585 (211881.3-249840.9) | 1115.5 (1007.5-1188) | -2.07 (-2.13 to -2.01) |
| United Republic of Tanzania | 14386.5 (11483.8-18356.9) | 797.4 (636.5-1017.5) | 41455.3 (30242.8-55206.4) | 1005.7 (733.7-1339.3) | 0.74 (0.7 to 0.78) |
| United States Virgin Islands | 205.1 (169.5-252.3) | 1468.5 (1213.5-1806.4) | 334.6 (226.2-468.6) | 1039.4 (702.7-1455.8) | -1.02 (-1.29 to -0.75) |
| United States of America | 893720.5 (832578.9-941422.8) | 1703.6 (1587-1794.5) | 1038381.8 (949976.6-1118367.4) | 1035.8 (947.6-1115.6) | -1.81 (-1.87 to -1.75) |
| Uruguay | 13358.6 (12306.2-14281.5) | 1979.7 (1823.8-2116.5) | 15422.5 (13775.6-16978.9) | 1705.5 (1523.4-1877.6) | -0.62 (-0.73 to -0.5) |
| Uzbekistan | 13479.6 (11631-15462.2) | 689.3 (594.7-790.6) | 28586.5 (22920.8-35323.7) | 614.9 (493.1-759.9) | -0.23 (-0.45 to -0.02) |
| Vanuatu | 56.8 (37.8-82.1) | 569.8 (379.8-824) | 256.9 (190-337.2) | 895.8 (662.7-1175.8) | 1.34 (1.22 to 1.47) |
| Venezuela (Bolivarian Republic of) | 10839.6 (9926.2-11664.9) | 692.2 (633.9-745) | 53979.2 (41981.7-68494.5) | 1031.7 (802.4-1309.1) | 1.18 (1.05 to 1.31) |
| Viet Nam | 30368 (23775.8-39979.7) | 435.6 (341-573.4) | 101843.5 (74540.5-136002.1) | 583 (426.7-778.6) | 1.06 (1.01 to 1.11) |
| Western Europe | 1594163.1 (1499042.1-1667198.1) | 1641.6 (1543.6-1716.8) | 1605816.4 (1412258.6-1743785.8) | 1076.8 (947-1169.3) | -1.39 (-1.43 to -1.35) |
| Western Sub-Saharan Africa | 112650 (93217.1-132295.8) | 780.4 (645.7-916.5) | 389121.2 (308835.1-492540.4) | 1210.6 (960.8-1532.3) | 1.54 (1.36 to 1.72) |
| Yemen | 1738.5 (1113.3-2406.9) | 216.8 (138.8-300.1) | 8133.6 (5721.5-11157.4) | 361.7 (254.4-496.1) | 1.88 (1.72 to 2.04) |
| Zambia | 3343.8 (2437.7-4515.3) | 726.4 (529.5-980.9) | 14866.8 (9310.3-22571.1) | 1376.4 (861.9-2089.6) | 2.22 (1.97 to 2.47) |
| Zimbabwe | 5279.5 (4007.6-6868.6) | 796.5 (604.6-1036.3) | 18199.2 (13107.8-23929.8) | 1643.6 (1183.8-2161.2) | 3.26 (2.61 to 3.92) |

**Table S8. Projections of global breast cancer incidence from 2021-2040**

| **sex** | **year** | **ASR** | **lower** | **upper** |
| --- | --- | --- | --- | --- |
| Male | 1990 | 2.40373888878928 | 2.35266489869633 | 2.45481287888223 |
| Male | 1991 | 2.43162638204199 | 2.38644606830514 | 2.47680669577884 |
| Male | 1992 | 2.49776499944629 | 2.45348243579312 | 2.54204756309945 |
| Male | 1993 | 2.58775484396306 | 2.54304921074925 | 2.63246047717686 |
| Male | 1994 | 2.66509045202556 | 2.62001179660679 | 2.71016910744433 |
| Male | 1995 | 2.71206131927326 | 2.66693105335613 | 2.75719158519039 |
| Male | 1996 | 2.73498663002423 | 2.69007817274831 | 2.77989508730016 |
| Male | 1997 | 2.75585707038918 | 2.71111277460398 | 2.80060136617438 |
| Male | 1998 | 2.79904330647165 | 2.75419107995755 | 2.84389553298574 |
| Male | 1999 | 2.87036153982862 | 2.82519823179314 | 2.9155248478641 |
| Male | 2000 | 2.93176973679285 | 2.88633566704941 | 2.9772038065363 |
| Male | 2001 | 2.95521997447174 | 2.90996964620904 | 3.00047030273444 |
| Male | 2002 | 2.96344857033726 | 2.91853176943946 | 3.00836537123506 |
| Male | 2003 | 2.98350316273673 | 2.93871312752307 | 3.02829319795039 |
| Male | 2004 | 3.03012849737281 | 2.985268731 | 3.07498826416063 |
| Male | 2005 | 3.14295177149513 | 3.09752764629918 | 3.18837589669107 |
| Male | 2006 | 3.25581364962739 | 3.20994612612237 | 3.30168117313241 |
| Male | 2007 | 3.35781725860582 | 3.31169129047818 | 3.40394322673347 |
| Male | 2008 | 3.50113852752343 | 3.45443009122791 | 3.54784696381894 |
| Male | 2009 | 3.62744140902562 | 3.58031202754377 | 3.67457079050746 |
| Male | 2010 | 3.76158065744277 | 3.71395258627669 | 3.80920872860885 |
| Male | 2011 | 3.87714624332694 | 3.82923946226056 | 3.92505302439332 |
| Male | 2012 | 3.98903703571237 | 3.94089323156198 | 4.03718083986276 |
| Male | 2013 | 4.05322704691984 | 4.00529019558956 | 4.10116389825011 |
| Male | 2014 | 4.07738249214075 | 4.02991191099315 | 4.12485307328835 |
| Male | 2015 | 4.08127654968659 | 4.0343719677016 | 4.12818113167159 |
| Male | 2016 | 4.11358260368394 | 4.06704052416025 | 4.16012468320763 |
| Male | 2017 | 4.12962467515156 | 4.08357769553814 | 4.17567165476499 |
| Male | 2018 | 4.14415823484963 | 4.09861811059502 | 4.18969835910425 |
| Male | 2019 | 4.14705477984166 | 4.10210226310528 | 4.19200729657805 |
| Male | 2020 | 4.14271333427518 | 4.09806507763805 | 4.18736159091231 |
| Male | 2021 | 4.13625199733046 | 4.08993669078629 | 4.18256730387463 |
| Male | 2022 | 4.13222250278766 | 3.95988999309837 | 4.30455501247695 |
| Male | 2023 | 4.12908875304908 | 3.88158508682043 | 4.37659241927773 |
| Male | 2024 | 4.12549725066921 | 3.78264562216857 | 4.46834887916985 |
| Male | 2025 | 4.12187626440562 | 3.66826602481108 | 4.57548650400016 |
| Male | 2026 | 4.11826002124701 | 3.54091576270695 | 4.69560427978707 |
| Male | 2027 | 4.11444350368338 | 3.40183800516472 | 4.82704900220204 |
| Male | 2028 | 4.11029419259328 | 3.25230141682205 | 4.96828696836452 |
| Male | 2029 | 4.10596643434763 | 3.09328474728017 | 5.11864812141508 |
| Male | 2030 | 4.10183598405469 | 2.92557451710597 | 5.27809745100342 |
| Male | 2031 | 4.09802141712454 | 2.74951000875967 | 5.44653282548941 |
| Male | 2032 | 4.0943500216341 | 2.56516260923252 | 5.62353743403567 |
| Male | 2033 | 4.09066106433192 | 2.37291048083936 | 5.80841164782447 |
| Male | 2034 | 4.0869234488652 | 2.17306579928879 | 6.0007810984416 |
| Male | 2035 | 4.08321674636361 | 1.96586803142229 | 6.20056546130493 |
| Male | 2036 | 4.07945807232106 | 1.75137066504267 | 6.40754547959945 |
| Male | 2037 | 4.07540193060041 | 1.52956868828847 | 6.62123517291236 |
| Male | 2038 | 4.07099854329692 | 1.30078167287106 | 6.84121541372277 |
| Male | 2039 | 4.06631412883705 | 1.06529949720249 | 7.0673287604716 |
| Male | 2040 | 4.06152598852597 | 0.823358621546783 | 7.29969335550517 |
| Female | 1990 | 142.93705663887 | 142.546753384486 | 143.327359893254 |
| Female | 1991 | 144.102929033049 | 143.721273784771 | 144.484584281327 |
| Female | 1992 | 145.293770469485 | 144.914833150736 | 145.672707788235 |
| Female | 1993 | 147.823079022376 | 147.444684307266 | 148.201473737486 |
| Female | 1994 | 149.965773608387 | 149.588291944483 | 150.34325527229 |
| Female | 1995 | 151.606805589461 | 151.230769446782 | 151.982841732141 |
| Female | 1996 | 152.396952832201 | 152.023337019096 | 152.770568645305 |
| Female | 1997 | 152.232197239216 | 151.862128456475 | 152.602266021958 |
| Female | 1998 | 152.17342812018 | 151.80652914091 | 152.54032709945 |
| Female | 1999 | 153.123679076971 | 152.758694965872 | 153.48866318807 |
| Female | 2000 | 153.262462214569 | 152.90037750524 | 153.624546923899 |
| Female | 2001 | 153.328357064775 | 152.969610727119 | 153.687103402432 |
| Female | 2002 | 153.531251022258 | 153.176424033715 | 153.886078010802 |
| Female | 2003 | 154.201369057325 | 153.849841213103 | 154.552896901548 |
| Female | 2004 | 154.730966538367 | 154.38339960871 | 155.078533468023 |
| Female | 2005 | 156.51335581233 | 156.167919802886 | 156.858791821774 |
| Female | 2006 | 157.640954416714 | 157.299093842664 | 157.982814990764 |
| Female | 2007 | 158.469356942733 | 158.131583360024 | 158.807130525441 |
| Female | 2008 | 159.769152454824 | 159.434931852364 | 160.103373057284 |
| Female | 2009 | 159.933592508658 | 159.604247520914 | 160.262937496403 |
| Female | 2010 | 160.34112849927 | 160.016231288049 | 160.666025710491 |
| Female | 2011 | 160.226801799896 | 159.906903422357 | 160.546700177435 |
| Female | 2012 | 159.672937645233 | 159.358383825402 | 159.987491465064 |
| Female | 2013 | 159.384058655127 | 159.074282571271 | 159.693834738983 |
| Female | 2014 | 159.190273487632 | 158.885060342837 | 159.495486632426 |
| Female | 2015 | 159.916640112594 | 159.614876332123 | 160.218403893065 |
| Female | 2016 | 160.625710929895 | 160.32751224958 | 160.92390961021 |
| Female | 2017 | 160.734007749995 | 160.440071113533 | 161.027944386456 |
| Female | 2018 | 161.660516843042 | 161.370162969079 | 161.950870717005 |
| Female | 2019 | 161.687690404445 | 161.401695202599 | 161.97368560629 |
| Female | 2020 | 159.782609319991 | 159.50232722654 | 160.062891413441 |
| Female | 2021 | 160.685332083721 | 160.40634982497 | 160.964314342471 |
| Female | 2022 | 159.363273772477 | 156.029207821586 | 162.697339723369 |
| Female | 2023 | 158.558646016373 | 153.475455975357 | 163.641836057388 |
| Female | 2024 | 157.776404498091 | 150.483638855877 | 165.069170140305 |
| Female | 2025 | 157.000165223706 | 147.15181119824 | 166.848519249172 |
| Female | 2026 | 156.235979441584 | 143.517479032484 | 168.954479850683 |
| Female | 2027 | 155.415220236766 | 139.520912153154 | 171.309528320377 |
| Female | 2028 | 154.605546058136 | 135.284470206197 | 173.926621910075 |
| Female | 2029 | 153.801686655741 | 130.833704604635 | 176.769668706847 |
| Female | 2030 | 152.999330335626 | 126.174966543917 | 179.823694127336 |
| Female | 2031 | 152.206498274899 | 121.307107133672 | 183.105889416126 |
| Female | 2032 | 151.3638691 | 116.168460110821 | 186.559278046779 |
| Female | 2033 | 150.524903915455 | 110.832602300772 | 190.217205530137 |
| Female | 2034 | 149.693226102027 | 105.318919384802 | 194.067532819252 |
| Female | 2035 | 148.868052607926 | 99.6322684260765 | 198.103836789776 |
| Female | 2036 | 148.055638000881 | 93.7718705745074 | 202.339405427254 |
| Female | 2037 | 147.208091245122 | 87.6959725199878 | 206.720209970257 |
| Female | 2038 | 146.36455571284 | 81.4513973200281 | 211.277714105652 |
| Female | 2039 | 145.527769334768 | 75.0516273550862 | 216.00391131445 |
| Female | 2040 | 144.694251491453 | 68.4993784272348 | 220.889124555672 |
| Both | 1990 | 80.10094832 | 79.8806398829881 | 80.321256766252 |
| Both | 1991 | 80.6346818388763 | 80.4195625045771 | 80.8498011731756 |
| Both | 1992 | 81.2229667777328 | 81.0098196819458 | 81.4361138735198 |
| Both | 1993 | 82.5649243368112 | 82.3524399996839 | 82.7774086739385 |
| Both | 1994 | 83.6597821933967 | 83.4482535691273 | 83.8713108176661 |
| Both | 1995 | 84.4985072977743 | 84.288128358905 | 84.7088862366437 |
| Both | 1996 | 84.8622379236133 | 84.6535225605549 | 85.0709532866717 |
| Both | 1997 | 84.6499532059826 | 84.4436617819479 | 84.8562446300174 |
| Both | 1998 | 84.5319266933963 | 84.3277876903412 | 84.7360656964513 |
| Both | 1999 | 84.9799068610881 | 84.7772261083253 | 85.1825876138509 |
| Both | 2000 | 84.9649470399409 | 84.7643032245557 | 85.165590855326 |
| Both | 2001 | 84.8995971263235 | 84.7011660293821 | 85.0980282232649 |
| Both | 2002 | 84.9123325695649 | 84.7163239021469 | 85.108341236983 |
| Both | 2003 | 85.1432672606918 | 84.9494402795907 | 85.3370942417929 |
| Both | 2004 | 85.2951188324631 | 85.1038289732043 | 85.4864086917218 |
| Both | 2005 | 86.1912653544959 | 86.0014898688172 | 86.3810408401747 |
| Both | 2006 | 86.7491620425462 | 86.5615945771976 | 86.9367295078948 |
| Both | 2007 | 87.1557541705527 | 86.9706230858183 | 87.340885255287 |
| Both | 2008 | 87.8478444758525 | 87.6648217971441 | 88.030867154561 |
| Both | 2009 | 87.9083118856716 | 87.728119116976 | 88.0885046543672 |
| Both | 2010 | 88.1131773281529 | 87.9355786106344 | 88.2907760456713 |
| Both | 2011 | 88.0412195737005 | 87.8664765851582 | 88.2159625622427 |
| Both | 2012 | 87.7272968858378 | 87.5556044865557 | 87.8989892851199 |
| Both | 2013 | 87.5210778553898 | 87.3521748619589 | 87.6899808488206 |
| Both | 2014 | 87.3657312265659 | 87.1994696998572 | 87.5319927532747 |
| Both | 2015 | 87.7155594680309 | 87.5513068003447 | 87.879812135717 |
| Both | 2016 | 88.0644606838818 | 87.9022743187659 | 88.2266470489977 |
| Both | 2017 | 88.0597820486908 | 87.9000629185452 | 88.2195011788364 |
| Both | 2018 | 88.5066562366866 | 88.3490102427966 | 88.6643022305766 |
| Both | 2019 | 88.4592400581526 | 88.3041038278249 | 88.6143762884804 |
| Both | 2020 | 87.4387427016141 | 87.2866770830988 | 87.5908083201295 |
| Both | 2021 | 87.9476235660017 | 87.796330859816 | 88.0989162721875 |
| Both | 2022 | 87.4833337527474 | 85.5268195230839 | 89.4398479824109 |
| Both | 2023 | 87.1182862075484 | 84.2015748084724 | 90.0349976066243 |
| Both | 2024 | 86.7630722617175 | 82.629180743507 | 90.896963779928 |
| Both | 2025 | 86.414750803436 | 80.8678337173157 | 91.9616678895564 |
| Both | 2026 | 86.0799376438166 | 78.940126513371 | 93.2197487742623 |
| Both | 2027 | 85.723445000254 | 76.8151547294772 | 94.6317352710308 |
| Both | 2028 | 85.3693360896563 | 74.5492467354595 | 96.1894254438532 |
| Both | 2029 | 85.0138941325037 | 72.1556326751491 | 97.8721555898584 |
| Both | 2030 | 84.6600737573746 | 69.641272592458 | 99.6788749222911 |
| Both | 2031 | 84.3150193996964 | 67.0060379559875 | 101.624000843405 |
| Both | 2032 | 83.9472828225206 | 64.2156828587103 | 103.678882786331 |
| Both | 2033 | 83.5779154772372 | 61.3044346039451 | 105.851396350529 |
| Both | 2034 | 83.2082463803698 | 58.2824024893857 | 108.134090271354 |
| Both | 2035 | 82.8416567738496 | 55.1543275222535 | 110.528986025446 |
| Both | 2036 | 82.4828380870318 | 51.9192484886818 | 113.046427685382 |
| Both | 2037 | 82.1047860014612 | 48.552540852716 | 115.657031150206 |
| Both | 2038 | 81.7252219828963 | 45.077858909998 | 118.372585055795 |
| Both | 2039 | 81.3454222610793 | 41.5025606970015 | 121.188283825157 |
| Both | 2040 | 80.9658166577455 | 37.8291120218848 | 124.102521293606 |

**Table S9. Projections of global breast cancer prevalence from 2021-2040**

| **sex** | **year** | **ASR** | **lower** | **upper** |
| --- | --- | --- | --- | --- |
| Male | 1990 | 19.4403202351042 | 19.2776883488036 | 19.6029521214047 |
| Male | 1991 | 19.4568909066397 | 19.3021062236947 | 19.6116755895848 |
| Male | 1992 | 19.9007428935643 | 19.7470654578357 | 20.0544203292928 |
| Male | 1993 | 20.5426685936881 | 20.3885307244865 | 20.6968064628898 |
| Male | 1994 | 21.0488936303702 | 20.8947635899957 | 21.2030236707448 |
| Male | 1995 | 21.3250949670862 | 21.1716803646134 | 21.478509569559 |
| Male | 1996 | 21.3819526728845 | 21.23001255 | 21.5338927923991 |
| Male | 1997 | 21.4179857775057 | 21.2675512714622 | 21.5684202835492 |
| Male | 1998 | 21.6611752109532 | 21.5113144486714 | 21.8110359732351 |
| Male | 1999 | 22.224576038315 | 22.0743839185824 | 22.3747681580476 |
| Male | 2000 | 22.7353770933982 | 22.5850306592251 | 22.8857235275712 |
| Male | 2001 | 22.9109313522392 | 22.7616550702858 | 23.0602076341925 |
| Male | 2002 | 22.8917604301484 | 22.7442919551435 | 23.0392289051534 |
| Male | 2003 | 22.9530231661568 | 22.8070006173428 | 23.0990457149708 |
| Male | 2004 | 23.2769469652846 | 23.1317101449959 | 23.4221837855733 |
| Male | 2005 | 24.2031451234263 | 24.0568952411471 | 24.3493950057055 |
| Male | 2006 | 25.1516069874909 | 25.0045927451294 | 25.2986212298524 |
| Male | 2007 | 25.9576022444057 | 25.8105192514111 | 26.1046852374003 |
| Male | 2008 | 27.1263616345512 | 26.9782805551267 | 27.2744427139756 |
| Male | 2009 | 28.155626172979 | 28.0070687567912 | 28.3041835891668 |
| Male | 2010 | 29.2635219071682 | 29.1143036617291 | 29.4127401526073 |
| Male | 2011 | 30.2810378218202 | 30.1315604575577 | 30.4305151860826 |
| Male | 2012 | 31.3048736619197 | 31.1552761776007 | 31.4544711462386 |
| Male | 2013 | 31.9981092474501 | 31.8491153315848 | 32.1471031633153 |
| Male | 2014 | 32.3820775144645 | 32.234412382259 | 32.5297426466699 |
| Male | 2015 | 32.5936879958267 | 32.4476703959813 | 32.739705595672 |
| Male | 2016 | 33.0609971137565 | 32.9160797387247 | 33.2059144887883 |
| Male | 2017 | 33.3900915695919 | 33.2467320313583 | 33.5334511078255 |
| Male | 2018 | 33.6746989399856 | 33.5329423473655 | 33.8164555326057 |
| Male | 2019 | 33.8615238031713 | 33.7216261062793 | 34.0014215000633 |
| Male | 2020 | 33.9983197067797 | 33.8599828039729 | 34.1366566095865 |
| Male | 2021 | 34.0802626868643 | 33.9422315864441 | 34.2182937872845 |
| Male | 2022 | 34.4461246917507 | 33.1355143397672 | 35.7567350437342 |
| Male | 2023 | 34.6308993291476 | 32.762508975257 | 36.4992896830382 |
| Male | 2024 | 34.8112769942851 | 32.2182286921501 | 37.4043252964201 |
| Male | 2025 | 34.9951191427298 | 31.5449932912745 | 38.4452449941851 |
| Male | 2026 | 35.1842485835736 | 30.7601317735385 | 39.6083653936088 |
| Male | 2027 | 35.3757168590975 | 29.8687700583229 | 40.8826636598721 |
| Male | 2028 | 35.5636883866866 | 28.8771640059042 | 42.2502127674691 |
| Male | 2029 | 35.749773707029 | 27.7923046503341 | 43.707242763724 |
| Male | 2030 | 35.9407441785853 | 26.6210589990707 | 45.2604293580999 |
| Male | 2031 | 36.139840550519 | 25.364268133224 | 46.915412967814 |
| Male | 2032 | 36.3450343217877 | 24.0182273541575 | 48.6718412894178 |
| Male | 2033 | 36.5517598051102 | 22.5827424954712 | 50.5207771147493 |
| Male | 2034 | 36.7595776953475 | 21.0586240461591 | 52.4605313445358 |
| Male | 2035 | 36.9712714957056 | 19.4466938281376 | 54.4958491632737 |
| Male | 2036 | 37.1872724525115 | 17.7446537632311 | 56.629891141792 |
| Male | 2037 | 37.4043003441543 | 15.948292638921 | 58.8603080493877 |
| Male | 2038 | 37.6197056702663 | 14.0578669860513 | 61.1815443544812 |
| Male | 2039 | 37.8341302967927 | 12.0743141045338 | 63.5939464890516 |
| Male | 2040 | 38.0510080982649 | 9.99840537859871 | 66.103610817931 |
| Female | 1990 | 1583.55163724213 | 1582.2323028651 | 1584.87097161915 |
| Female | 1991 | 1569.96369485319 | 1568.67030037629 | 1571.25708933009 |
| Female | 1992 | 1557.49936854122 | 1556.22701926048 | 1558.77171782195 |
| Female | 1993 | 1557.08699745608 | 1555.82962438842 | 1558.34437052374 |
| Female | 1994 | 1558.97872147498 | 1557.73435647493 | 1560.22308647503 |
| Female | 1995 | 1563.72293397304 | 1562.48978862433 | 1564.95607932176 |
| Female | 1996 | 1566.44739501983 | 1565.22563619057 | 1567.66915384909 |
| Female | 1997 | 1563.44100440714 | 1562.23223598489 | 1564.64977282939 |
| Female | 1998 | 1562.21490817459 | 1561.01778805915 | 1563.41202829004 |
| Female | 1999 | 1569.7042829557 | 1568.51531874547 | 1570.89324716593 |
| Female | 2000 | 1574.3146872571 | 1573.13492097636 | 1575.49445353783 |
| Female | 2001 | 1578.01903640931 | 1576.84993186584 | 1579.18814095278 |
| Female | 2002 | 1581.68471060455 | 1580.52845624312 | 1582.84096496598 |
| Female | 2003 | 1589.08964769754 | 1587.94450173185 | 1590.23479366324 |
| Female | 2004 | 1597.28878767866 | 1596.15598547646 | 1598.42158988087 |
| Female | 2005 | 1614.17601028276 | 1613.05132456826 | 1615.30069599726 |
| Female | 2006 | 1626.5976668146 | 1625.48477379263 | 1627.71055983657 |
| Female | 2007 | 1636.96980873951 | 1635.86997357464 | 1638.06964390438 |
| Female | 2008 | 1651.05266934774 | 1649.96458054887 | 1652.14075814661 |
| Female | 2009 | 1656.97346414821 | 1655.90023884257 | 1658.04668945385 |
| Female | 2010 | 1663.4911805738 | 1662.43200046741 | 1664.5503606802 |
| Female | 2011 | 1664.52150942346 | 1663.47818630047 | 1665.56483254644 |
| Female | 2012 | 1662.26412781501 | 1661.23742415814 | 1663.29083147187 |
| Female | 2013 | 1662.7877734223 | 1661.77595255215 | 1663.79959429245 |
| Female | 2014 | 1663.19644008369 | 1662.19923561828 | 1664.1936445491 |
| Female | 2015 | 1668.8783107001 | 1667.89343435132 | 1669.86318704889 |
| Female | 2016 | 1674.21632149286 | 1673.24426493079 | 1675.18837805493 |
| Female | 2017 | 1676.19814571102 | 1675.24018858323 | 1677.15610283881 |
| Female | 2018 | 1683.5393332734 | 1682.59409404689 | 1684.48457249991 |
| Female | 2019 | 1684.09533971425 | 1683.16455630747 | 1685.02612312103 |
| Female | 2020 | 1670.23287402561 | 1669.31928461248 | 1671.14646343874 |
| Female | 2021 | 1676.27897450498 | 1675.37511223689 | 1677.18283677306 |
| Female | 2022 | 1670.50954338168 | 1641.50620474635 | 1699.512882 |
| Female | 2023 | 1667.02143045343 | 1623.27340079767 | 1710.76946010918 |
| Female | 2024 | 1663.33254724666 | 1600.81249443827 | 1725.85260005506 |
| Female | 2025 | 1659.35049781851 | 1574.99509229476 | 1743.70590334226 |
| Female | 2026 | 1655.3400778633 | 1546.33668573757 | 1764.34346998902 |
| Female | 2027 | 1650.92576361305 | 1514.49502021027 | 1787.35650701582 |
| Female | 2028 | 1646.43785589149 | 1480.29195376834 | 1812.58375801465 |
| Female | 2029 | 1641.69485664879 | 1443.82118842774 | 1839.56852486985 |
| Female | 2030 | 1636.68778370503 | 1405.15614371432 | 1868.21942369575 |
| Female | 2031 | 1631.63327746483 | 1364.40038351056 | 1898.8661714191 |
| Female | 2032 | 1626.18178194451 | 1321.11711873596 | 1931.24644515306 |
| Female | 2033 | 1620.62372668164 | 1275.80891445418 | 1965.4385389091 |
| Female | 2034 | 1614.89622972132 | 1228.56742211069 | 2001.22503733194 |
| Female | 2035 | 1609.01440642177 | 1179.44787260266 | 2038.58094024088 |
| Female | 2036 | 1603.16516198878 | 1128.53478558783 | 2077.79553838974 |
| Female | 2037 | 1597.05383251802 | 1075.51078943929 | 2118.59687559676 |
| Female | 2038 | 1590.89149931243 | 1020.70218028786 | 2161.08081833699 |
| Female | 2039 | 1584.62687083013 | 964.17382669184 | 2205.07991496842 |
| Female | 2040 | 1578.2329122996 | 905.946738414764 | 2250.51908618444 |
| Both | 1990 | 891.267878681907 | 890.515101735723 | 892.020655628091 |
| Both | 1991 | 881.530664031898 | 880.79549905347 | 882.265829010326 |
| Both | 1992 | 872.779201186062 | 872.058419332761 | 873.499983039364 |
| Both | 1993 | 870.815636764261 | 870.105681263705 | 871.525592264817 |
| Both | 1994 | 870.190343902909 | 869.489967286438 | 870.890720519379 |
| Both | 1995 | 871.474076055504 | 870.781837947485 | 872.166314163523 |
| Both | 1996 | 871.808271350965 | 871.123962088324 | 872.492580613606 |
| Both | 1997 | 868.822749377783 | 868.14735606461 | 869.498142690957 |
| Both | 1998 | 866.966404708928 | 866.299107865345 | 867.63370155251 |
| Both | 1999 | 869.96261902578 | 869.30151209196 | 870.623726 |
| Both | 2000 | 871.395323785744 | 870.740953447643 | 872.049694123844 |
| Both | 2001 | 872.30593734101 | 871.658851027114 | 872.953023654907 |
| Both | 2002 | 873.103356708251 | 872.464472908591 | 873.74224050791 |
| Both | 2003 | 875.695262486802 | 875.063791619778 | 876.326733353826 |
| Both | 2004 | 878.896886503989 | 878.27330751331 | 879.520465494669 |
| Both | 2005 | 887.126165507623 | 886.508270929026 | 887.744060086219 |
| Both | 2006 | 893.132108547909 | 892.521612947696 | 893.742604148121 |
| Both | 2007 | 898.072126096743 | 897.469610277761 | 898.674641915726 |
| Both | 2008 | 905.219029840498 | 904.623677687648 | 905.814381993348 |
| Both | 2009 | 907.986113123625 | 907.399521285701 | 908.572704961548 |
| Both | 2010 | 911.151565829359 | 910.573294089693 | 911.729837569025 |
| Both | 2011 | 911.394096954384 | 910.825036717326 | 911.963157191443 |
| Both | 2012 | 909.829945419549 | 909.270518147963 | 910.389372691136 |
| Both | 2013 | 909.549084458083 | 908.998406873007 | 910.099762043159 |
| Both | 2014 | 909.27791824029 | 908.735700966175 | 909.820135514405 |
| Both | 2015 | 911.874950570052 | 911.33991039141 | 912.409990748693 |
| Both | 2016 | 914.354342390556 | 913.826720602397 | 914.881964178715 |
| Both | 2017 | 914.875060614222 | 914.355570384181 | 915.394550844262 |
| Both | 2018 | 918.272541287313 | 917.760380742489 | 918.784701832137 |
| Both | 2019 | 918.005948196011 | 917.502038274775 | 918.509858117246 |
| Both | 2020 | 910.772650030393 | 910.277941357525 | 911.267358703262 |
| Both | 2021 | 914.585882893974 | 914.096162499694 | 915.075603288254 |
| Both | 2022 | 914.330130024079 | 897.740049375999 | 930.92021067216 |
| Both | 2023 | 913.385585410184 | 888.641823509661 | 938.129347310706 |
| Both | 2024 | 912.304225342434 | 877.174327268284 | 947.434123416584 |
| Both | 2025 | 911.099378618532 | 863.860206785249 | 958.338550451815 |
| Both | 2026 | 909.982434301401 | 849.021484081213 | 970.943384521589 |
| Both | 2027 | 908.743866723776 | 832.445509331429 | 985.042224116123 |
| Both | 2028 | 907.442029714597 | 814.486965249875 | 1000.39709417932 |
| Both | 2029 | 905.954982396568 | 795.179605897076 | 1016.73035889606 |
| Both | 2030 | 904.331060131007 | 774.600784196337 | 1034.06133606568 |
| Both | 2031 | 902.73590707448 | 752.825451493437 | 1052.64636265552 |
| Both | 2032 | 900.968396565032 | 729.582766579105 | 1072.35402655096 |
| Both | 2033 | 899.110891353403 | 705.093366438189 | 1093.12841626862 |
| Both | 2034 | 897.112061923126 | 679.395906826362 | 1114.82821701989 |
| Both | 2035 | 895.017604161289 | 652.543233526353 | 1137.49197479622 |
| Both | 2036 | 892.96045846306 | 624.587592896925 | 1161.33332402919 |
| Both | 2037 | 890.760873461201 | 595.321964987988 | 1186.19978193441 |
| Both | 2038 | 888.501072802264 | 564.907694810972 | 1212.09445079356 |
| Both | 2039 | 886.14260999386 | 533.374816814472 | 1238.91040317325 |
| Both | 2040 | 883.690740950885 | 500.743554184618 | 1266.63792771715 |

**Table S10. Projections of global breast cancer deaths from 2021-2040**

| **sex** | **year** | **ASR** | **lower** | **upper** |
| --- | --- | --- | --- | --- |
| Male | 1990 | 1.3747086325321 | 1.3401729187064 | 1.4092443463578 |
| Male | 1991 | 1.38976984122639 | 1.36163771070797 | 1.41790197174481 |
| Male | 1992 | 1.40879126559032 | 1.38313540228409 | 1.43444712889655 |
| Male | 1993 | 1.43107921171944 | 1.40604915504405 | 1.45610926839484 |
| Male | 1994 | 1.45123693033286 | 1.4263289999756 | 1.47614486069012 |
| Male | 1995 | 1.46636904446848 | 1.44157974023245 | 1.49115834870451 |
| Male | 1996 | 1.47786615896292 | 1.4532457813777 | 1.50248653654813 |
| Male | 1997 | 1.48887651618047 | 1.4644025697184 | 1.51335046264254 |
| Male | 1998 | 1.50431411461709 | 1.47990024189627 | 1.52872798733792 |
| Male | 1999 | 1.52125420593281 | 1.49685948838129 | 1.54564892348434 |
| Male | 2000 | 1.53772788357858 | 1.513327583 | 1.56212818376316 |
| Male | 2001 | 1.54830146122072 | 1.52397886040532 | 1.57262406203612 |
| Male | 2002 | 1.55690742458244 | 1.53269240157916 | 1.58112244758572 |
| Male | 2003 | 1.56573556187854 | 1.54160815944809 | 1.58986296430899 |
| Male | 2004 | 1.57381309480245 | 1.54977834774076 | 1.59784784186415 |
| Male | 2005 | 1.58730111857484 | 1.56330355848142 | 1.61129867866827 |
| Male | 2006 | 1.60038302071561 | 1.57645843082796 | 1.62430761060325 |
| Male | 2007 | 1.61253409609125 | 1.58871814633962 | 1.63635004584289 |
| Male | 2008 | 1.6291792638051 | 1.60542017410229 | 1.65293835350791 |
| Male | 2009 | 1.64439449458467 | 1.62069899328826 | 1.66808999588108 |
| Male | 2010 | 1.65844179057929 | 1.63480265529096 | 1.68208092586763 |
| Male | 2011 | 1.66732404932716 | 1.64380233402097 | 1.69084576463335 |
| Male | 2012 | 1.67572545578169 | 1.6523221458797 | 1.69912876568368 |
| Male | 2013 | 1.67611594485654 | 1.65295097168463 | 1.69928091802844 |
| Male | 2014 | 1.67265736320858 | 1.64973865922191 | 1.69557606719525 |
| Male | 2015 | 1.66720400354633 | 1.6445119404195 | 1.68989606667315 |
| Male | 2016 | 1.66391758982526 | 1.64141227540928 | 1.68642290424123 |
| Male | 2017 | 1.65600695060324 | 1.63375296090108 | 1.6782609403054 |
| Male | 2018 | 1.6501594845023 | 1.62815386122295 | 1.67216510778166 |
| Male | 2019 | 1.64114214172688 | 1.61933344948385 | 1.66295083396991 |
| Male | 2020 | 1.63054341348524 | 1.60808074250776 | 1.65300608446271 |
| Male | 2021 | 1.61728688744181 | 1.59118035700246 | 1.64339341788116 |
| Male | 2022 | 1.60800214408026 | 1.55877808090817 | 1.65722620725236 |
| Male | 2023 | 1.59707871368843 | 1.52797662137666 | 1.66618080600019 |
| Male | 2024 | 1.58617380445091 | 1.49300406160004 | 1.67934354730178 |
| Male | 2025 | 1.57531176238804 | 1.45489332740566 | 1.69573019737043 |
| Male | 2026 | 1.56444687099893 | 1.41413767111325 | 1.71475607088461 |
| Male | 2027 | 1.55356385324275 | 1.37106960096256 | 1.73605810552294 |
| Male | 2028 | 1.54262419214931 | 1.32601579641264 | 1.75923258788599 |
| Male | 2029 | 1.53164038135949 | 1.27921720285249 | 1.78406355986649 |
| Male | 2030 | 1.52066709602036 | 1.23086117720408 | 1.81047301483664 |
| Male | 2031 | 1.509693142 | 1.18102563941515 | 1.83836064419286 |
| Male | 2032 | 1.49869397242409 | 1.12977474195381 | 1.86761320289437 |
| Male | 2033 | 1.48762447768976 | 1.07723472456799 | 1.89801423081153 |
| Male | 2034 | 1.47649511658681 | 1.02352672613875 | 1.92946350703488 |
| Male | 2035 | 1.46535176201862 | 0.968754876982553 | 1.96194864705468 |
| Male | 2036 | 1.45418710057344 | 0.912958166454669 | 1.9954160346922 |
| Male | 2037 | 1.44296960401678 | 0.85616515743658 | 2.02977405059698 |
| Male | 2038 | 1.43166582914208 | 0.798465057973778 | 2.06486660031039 |
| Male | 2039 | 1.42029467854248 | 0.739953217863938 | 2.10063613922102 |
| Male | 2040 | 1.40890421009647 | 0.680714040254146 | 2.13709437993879 |
| Female | 1990 | 67.7218331835519 | 67.4518049071423 | 67.9918614599614 |
| Female | 1991 | 67.7420613007938 | 67.4844952874857 | 67.999627314102 |
| Female | 1992 | 67.8601978298312 | 67.6056308675575 | 68.1147647921048 |
| Female | 1993 | 68.2727072669864 | 68.0200398975421 | 68.5253746364307 |
| Female | 1994 | 68.3904601130679 | 68.1401846307659 | 68.6407356 |
| Female | 1995 | 68.0673233117445 | 67.820127989633 | 68.3145186338561 |
| Female | 1996 | 67.4991582529865 | 67.2554265 | 67.742890007553 |
| Female | 1997 | 66.8082982226055 | 66.5682112342132 | 67.0483852109978 |
| Female | 1998 | 66.3460533246547 | 66.1089942316662 | 66.5831124176432 |
| Female | 1999 | 66.2759872700365 | 66.0412254697952 | 66.5107490702778 |
| Female | 2000 | 65.8755564744778 | 65.6436904993429 | 66.1074224496128 |
| Female | 2001 | 65.3855369993029 | 65.156876769507 | 65.6141972290987 |
| Female | 2002 | 64.8801896993089 | 64.6551051665645 | 65.1052742320533 |
| Female | 2003 | 64.4202388994753 | 64.1985877251676 | 64.641890073783 |
| Female | 2004 | 63.6037588520681 | 63.3864033349814 | 63.8211143691548 |
| Female | 2005 | 63.0702930187288 | 62.8564986314705 | 63.2840874059871 |
| Female | 2006 | 62.4151472068391 | 62.2054586733378 | 62.6248357403403 |
| Female | 2007 | 61.9302220534803 | 61.724382705225 | 62.1360614017355 |
| Female | 2008 | 61.7330434663112 | 61.5304666626258 | 61.9356202699966 |
| Female | 2009 | 61.2419499714562 | 61.0431509409913 | 61.440749001921 |
| Female | 2010 | 60.9444388715734 | 60.7490210136506 | 61.1398567294962 |
| Female | 2011 | 60.5533254532562 | 60.3614354087837 | 60.7452154977288 |
| Female | 2012 | 60.1400876537309 | 59.9516812742651 | 60.3284940331967 |
| Female | 2013 | 59.7518057886237 | 59.5666474174709 | 59.9369641597766 |
| Female | 2014 | 59.4474697412626 | 59.2653157879661 | 59.6296236945591 |
| Female | 2015 | 59.6477757268708 | 59.4677006773334 | 59.8278507764082 |
| Female | 2016 | 59.7710063241662 | 59.5931693357856 | 59.9488433125468 |
| Female | 2017 | 59.6278798509832 | 59.4527815273329 | 59.8029781746336 |
| Female | 2018 | 59.7602324942901 | 59.5874761534613 | 59.9329888351189 |
| Female | 2019 | 59.5418906740941 | 59.3720268951909 | 59.7117544529973 |
| Female | 2020 | 58.7398907541321 | 58.5734726405007 | 58.9063088677634 |
| Female | 2021 | 58.5802546016624 | 58.4133108876561 | 58.7471983156687 |
| Female | 2022 | 57.9873491550988 | 56.8627022224079 | 59.1119960877897 |
| Female | 2023 | 57.4594264295089 | 55.7084360959152 | 59.2104167631025 |
| Female | 2024 | 56.9397725969473 | 54.4140548224679 | 59.4654903714266 |
| Female | 2025 | 56.421968666024 | 53.0136430061436 | 59.8302943259043 |
| Female | 2026 | 55.9079791629811 | 51.521069988835 | 60.2948883371273 |
| Female | 2027 | 55.3858744247859 | 49.9275277041337 | 60.8442211454381 |
| Female | 2028 | 54.8778115028455 | 48.2754253840285 | 61.4801976216625 |
| Female | 2029 | 54.3761266275806 | 46.5692763379151 | 62.1829769172461 |
| Female | 2030 | 53.8783319938118 | 44.8115384394318 | 62.9451255481919 |
| Female | 2031 | 53.3834285035097 | 42.9997145251828 | 63.7671424818365 |
| Female | 2032 | 52.8758090789121 | 41.1172938191297 | 64.6343243386945 |
| Female | 2033 | 52.3754291966017 | 39.1918906913498 | 65.5589677018537 |
| Female | 2034 | 51.880484788123 | 37.2283838349989 | 66.5325857412471 |
| Female | 2035 | 51.3913493789281 | 35.2293552819352 | 67.5533434759211 |
| Female | 2036 | 50.9073706166726 | 33.1924877153308 | 68.6222535180145 |
| Female | 2037 | 50.4121031324821 | 31.103296582862 | 69.7209096821023 |
| Female | 2038 | 49.9220246396733 | 28.9807724268341 | 70.8632768525124 |
| Female | 2039 | 49.4369563526655 | 26.8292041553043 | 72.0447085500268 |
| Female | 2040 | 48.9565868774519 | 24.6501453418648 | 73.263028413039 |
| Both | 1990 | 39.0224513377844 | 38.8644096040192 | 39.1804930715497 |
| Both | 1991 | 38.9893373260389 | 38.8381755126466 | 39.1404991394313 |
| Both | 1992 | 39.0228515531629 | 38.8738365807247 | 39.171866525601 |
| Both | 1993 | 39.2285199479289 | 39.0809000725844 | 39.3761398232734 |
| Both | 1994 | 39.2500360025715 | 39.1041730194209 | 39.395898985722 |
| Both | 1995 | 39.0409788694916 | 38.8971603335967 | 39.1847974053866 |
| Both | 1996 | 38.6910125797181 | 38.5494308516929 | 38.8325943077434 |
| Both | 1997 | 38.2389718106688 | 38.0998614540573 | 38.3780821672802 |
| Both | 1998 | 37.9419729258943 | 37.8048963978698 | 38.0790494539188 |
| Both | 1999 | 37.868752359533 | 37.7332912653306 | 38.0042134537354 |
| Both | 2000 | 37.6062729553697 | 37.4727688910047 | 37.7397770197347 |
| Both | 2001 | 37.2966743878079 | 37.1652339212338 | 37.4281148543819 |
| Both | 2002 | 36.981261725403 | 36.8520317611754 | 37.1104916896305 |
| Both | 2003 | 36.6758896066825 | 36.5488485680924 | 36.8029306452726 |
| Both | 2004 | 36.1590211443203 | 36.0346628891553 | 36.2833793994854 |
| Both | 2005 | 35.8254377697182 | 35.7033381201555 | 35.9475374192808 |
| Both | 2006 | 35.4282423696871 | 35.3086446330182 | 35.5478401063559 |
| Both | 2007 | 35.1268235731125 | 35.0095752297579 | 35.2440719164671 |
| Both | 2008 | 34.9900878272239 | 34.8748640710066 | 35.1053115834412 |
| Both | 2009 | 34.6794663010465 | 34.5665744944554 | 34.7923581076375 |
| Both | 2010 | 34.4850401984134 | 34.3742605592878 | 34.595819837539 |
| Both | 2011 | 34.2451896833383 | 34.1365559022179 | 34.3538234644587 |
| Both | 2012 | 33.987767041406 | 33.8812695719808 | 34.0942645108312 |
| Both | 2013 | 33.7342192360799 | 33.6297398683575 | 33.8386986038023 |
| Both | 2014 | 33.5361834623113 | 33.4335440352319 | 33.6388228893908 |
| Both | 2015 | 33.6236002015175 | 33.522268182327 | 33.724932220708 |
| Both | 2016 | 33.6651926079936 | 33.5652599353674 | 33.7651252806198 |
| Both | 2017 | 33.5433479728971 | 33.4451029057856 | 33.6415930400086 |
| Both | 2018 | 33.5825564191142 | 33.4857679260908 | 33.6793449121377 |
| Both | 2019 | 33.4221853289811 | 33.3271811503085 | 33.5171895076538 |
| Both | 2020 | 32.9650997432724 | 32.8720464312076 | 33.0581530553373 |
| Both | 2021 | 32.8650121818736 | 32.7720487914246 | 32.9579755723225 |
| Both | 2022 | 32.6224743436692 | 31.9354490017735 | 33.309499685565 |
| Both | 2023 | 32.345062515371 | 31.3088918586138 | 33.3812331721281 |
| Both | 2024 | 32.0718704714903 | 30.6018415764903 | 33.5418993664903 |
| Both | 2025 | 31.8017138162025 | 29.8355584526128 | 33.7678691797922 |
| Both | 2026 | 31.5377510032675 | 29.0195846561929 | 34.0559173503422 |
| Both | 2027 | 31.2747916888359 | 28.1504565295248 | 34.399126848147 |
| Both | 2028 | 31.0184583618992 | 27.246156757535 | 34.7907599662633 |
| Both | 2029 | 30.7640727072411 | 26.3088534728056 | 35.2192919416766 |
| Both | 2030 | 30.5124566882615 | 25.3414139629487 | 35.6834994135742 |
| Both | 2031 | 30.2645492400256 | 24.3430862421819 | 36.1860122378693 |
| Both | 2032 | 30.012718282658 | 23.3051899098285 | 36.7202466554874 |
| Both | 2033 | 29.7628438965087 | 22.2391531617278 | 37.2865346312896 |
| Both | 2034 | 29.513750410718 | 21.147633164043 | 37.879867657393 |
| Both | 2035 | 29.2676332965986 | 20.0332980389134 | 38.5019685542838 |
| Both | 2036 | 29.0250598371923 | 18.8950264376024 | 39.1550932367821 |
| Both | 2037 | 28.7773011868859 | 17.7246931350643 | 39.8299092387075 |
| Both | 2038 | 28.5304266912555 | 16.5310091001104 | 40.5298442824006 |
| Both | 2039 | 28.2843743973205 | 15.3164964286282 | 41.2522523660128 |
| Both | 2040 | 28.0405562205414 | 14.08283423 | 41.9982782155229 |

**Table S11. Projections of global breast cancer DALYs from 2021-2040**

| **sex** | **year** | **ASR** | **lower** | **upper** |
| --- | --- | --- | --- | --- |
| Male | 1990 | 30.2095274368974 | 30.0138287261071 | 30.4052261476877 |
| Male | 1991 | 30.1627853198469 | 29.9753840101095 | 30.3501866295843 |
| Male | 1992 | 30.5907514729756 | 30.4052266440412 | 30.7762763 |
| Male | 1993 | 31.3914265652224 | 31.205593444359 | 31.5772596860858 |
| Male | 1994 | 32.004787685239 | 31.8190266351686 | 32.1905487353094 |
| Male | 1995 | 32.2328749539365 | 32.0483182101571 | 32.417431697716 |
| Male | 1996 | 32.2194090491142 | 32.0367444949463 | 32.4020736032821 |
| Male | 1997 | 32.2162443166072 | 32.0353405880661 | 32.3971480451482 |
| Male | 1998 | 32.6175406089483 | 32.4370560405037 | 32.7980251773928 |
| Male | 1999 | 33.1730395123892 | 32.9926178149535 | 33.3534612098248 |
| Male | 2000 | 33.7673601852584 | 33.5869678541273 | 33.9477525163895 |
| Male | 2001 | 33.8630733760329 | 33.6842250384941 | 34.0419217135716 |
| Male | 2002 | 33.9257496780236 | 33.7487912520528 | 34.1027081039944 |
| Male | 2003 | 34.0412395333753 | 33.865954176585 | 34.2165248901656 |
| Male | 2004 | 34.0829314130522 | 33.909749921263 | 34.2561129048414 |
| Male | 2005 | 34.6105352018412 | 34.4380986174458 | 34.7829717862365 |
| Male | 2006 | 35.0779184833572 | 34.9067049818642 | 35.2491319848503 |
| Male | 2007 | 35.360078034796 | 35.1907801713112 | 35.5293758982808 |
| Male | 2008 | 36.0220653419662 | 35.8537615148608 | 36.1903691690716 |
| Male | 2009 | 36.5702625209041 | 36.4032801902773 | 36.7372448515309 |
| Male | 2010 | 37.1167689623169 | 36.9510141418081 | 37.2825237828258 |
| Male | 2011 | 37.4379651918377 | 37.2739947180504 | 37.6019356656251 |
| Male | 2012 | 37.9039373494713 | 37.7414693571672 | 38.0664053417755 |
| Male | 2013 | 37.8381227375883 | 37.678166096383 | 37.9980793787936 |
| Male | 2014 | 37.6429381784422 | 37.485656519593 | 37.8002198372914 |
| Male | 2015 | 37.4723659983275 | 37.3176788758168 | 37.6270531208381 |
| Male | 2016 | 37.5372340306251 | 37.3846093453209 | 37.6898587159293 |
| Male | 2017 | 37.3297676779809 | 37.179893407406 | 37.4796419485558 |
| Male | 2018 | 37.2906418512539 | 37.1431257600308 | 37.4381579424771 |
| Male | 2019 | 37.1203598383334 | 36.9754597802747 | 37.265259896392 |
| Male | 2020 | 36.8774209605211 | 36.7347612838889 | 37.0200806371533 |
| Male | 2021 | 36.5412461308179 | 36.3993788521172 | 36.6831134095186 |
| Male | 2022 | 36.3315326636024 | 35.2395751809335 | 37.4234901462713 |
| Male | 2023 | 36.0783679577845 | 34.6078516120433 | 37.5488843035258 |
| Male | 2024 | 35.8226470158025 | 33.8631098607434 | 37.7821841708616 |
| Male | 2025 | 35.5664499580085 | 33.0358395046638 | 38.0970604113533 |
| Male | 2026 | 35.308976270818 | 32.1413772822835 | 38.4765752593525 |
| Male | 2027 | 35.0493502948483 | 31.188802956305 | 38.9098976333916 |
| Male | 2028 | 34.7872216868083 | 30.1872058303452 | 39.3872375432714 |
| Male | 2029 | 34.5238526851017 | 29.1436629477633 | 39.9040424224402 |
| Male | 2030 | 34.2613555763497 | 28.0636654063637 | 40.4590457463357 |
| Male | 2031 | 33.9997423053134 | 26.9494467072654 | 41.0500379033615 |
| Male | 2032 | 33.7382230934515 | 25.8023125712017 | 41.6741336157013 |
| Male | 2033 | 33.4763210796129 | 24.6254539290215 | 42.3271882302043 |
| Male | 2034 | 33.2145957864369 | 23.4218771762407 | 43.0073143966331 |
| Male | 2035 | 32.9541081680962 | 22.1940811249659 | 43.7141352112265 |
| Male | 2036 | 32.6946173382357 | 20.9430237445365 | 44.4462109319349 |
| Male | 2037 | 32.4352346375425 | 19.6692786836216 | 45.2011905914634 |
| Male | 2038 | 32.175657281066 | 18.3749900410475 | 45.9763245210845 |
| Male | 2039 | 31.9164161583669 | 17.0623597235825 | 46.7704725931514 |
| Male | 2040 | 31.6583936885675 | 15.7333083377804 | 47.5834790393546 |
| Female | 1990 | 1550.7945662272 | 1549.49838365393 | 1552.09074880047 |
| Female | 1991 | 1549.28841373029 | 1548.00991733524 | 1550.56691012533 |
| Female | 1992 | 1549.42245424245 | 1548.15797695456 | 1550.68693153033 |
| Female | 1993 | 1559.93091450705 | 1558.67548435445 | 1561.18634465965 |
| Female | 1994 | 1565.24353348131 | 1563.99853969888 | 1566.48852726375 |
| Female | 1995 | 1558.26315780049 | 1557.03287918259 | 1559.49343641839 |
| Female | 1996 | 1544.89268476097 | 1543.67896977278 | 1546.10639974916 |
| Female | 1997 | 1530.85340876082 | 1529.6560382396 | 1532.05077928204 |
| Female | 1998 | 1520.45795227824 | 1519.2746395103 | 1521.64126504619 |
| Female | 1999 | 1521.61677458072 | 1520.44288123148 | 1522.79066792996 |
| Female | 2000 | 1514.05527649034 | 1512.89395762917 | 1515.21659535152 |
| Female | 2001 | 1503.76717929617 | 1502.62091816727 | 1504.91344042507 |
| Female | 2002 | 1492.35888000427 | 1491.23125595479 | 1493.48650405375 |
| Female | 2003 | 1484.43623922155 | 1483.32552110027 | 1485.54695734283 |
| Female | 2004 | 1469.47677830174 | 1468.38733611486 | 1470.56622048863 |
| Female | 2005 | 1461.81235536104 | 1460.73965432497 | 1462.88505639712 |
| Female | 2006 | 1448.54754378567 | 1447.49562314963 | 1449.59946442171 |
| Female | 2007 | 1437.78333535993 | 1436.751307 | 1438.81536389785 |
| Female | 2008 | 1433.66599614748 | 1432.65137669345 | 1434.68061560152 |
| Female | 2009 | 1422.31300587752 | 1421.31843193217 | 1423.30757982288 |
| Female | 2010 | 1415.94282664365 | 1414.96607563335 | 1416.91957765396 |
| Female | 2011 | 1406.64433366411 | 1405.68581808326 | 1407.60284924495 |
| Female | 2012 | 1397.19788055898 | 1396.25703804416 | 1398.1387230738 |
| Female | 2013 | 1388.98872360965 | 1388.06412254816 | 1389.91332467114 |
| Female | 2014 | 1382.765302 | 1381.85572576184 | 1383.67487742816 |
| Female | 2015 | 1387.66957158972 | 1386.77084001716 | 1388.56830316228 |
| Female | 2016 | 1391.22896889373 | 1390.34176043597 | 1392.1161773515 |
| Female | 2017 | 1389.34417817184 | 1388.47073257768 | 1390.21762376601 |
| Female | 2018 | 1394.22540707575 | 1393.36403433631 | 1395.08677981518 |
| Female | 2019 | 1390.91283173508 | 1390.065927 | 1391.75973616616 |
| Female | 2020 | 1371.70147106062 | 1370.87274332695 | 1372.53019879429 |
| Female | 2021 | 1373.23434538963 | 1372.41554331609 | 1374.05314746317 |
| Female | 2022 | 1354.97314509644 | 1325.782827 | 1384.16346292888 |
| Female | 2023 | 1342.52255122879 | 1298.68855435431 | 1386.35654810327 |
| Female | 2024 | 1330.23982451251 | 1267.84341177232 | 1392.63623725271 |
| Female | 2025 | 1317.99955170158 | 1234.15547299004 | 1401.84363041311 |
| Female | 2026 | 1305.85371549045 | 1198.00864611036 | 1413.69878487055 |
| Female | 2027 | 1293.40888737818 | 1159.13518089223 | 1427.68259386413 |
| Female | 2028 | 1281.17146619932 | 1118.5202201051 | 1443.82271229354 |
| Female | 2029 | 1269.07809043753 | 1076.37925324591 | 1461.77692762916 |
| Female | 2030 | 1257.05066426467 | 1032.75512300066 | 1481.34620552868 |
| Female | 2031 | 1245.11263838778 | 987.649574763673 | 1502.57570201189 |
| Female | 2032 | 1232.94297292241 | 940.747844462182 | 1525.13810138264 |
| Female | 2033 | 1220.90836130439 | 892.604697880509 | 1549.21202472826 |
| Female | 2034 | 1209.00165595393 | 843.3650229 | 1574.63828904447 |
| Female | 2035 | 1197.1856364558 | 793.069043005123 | 1601.30222990648 |
| Female | 2036 | 1185.48940032223 | 741.73424829715 | 1629.24455234731 |
| Female | 2037 | 1173.67596015179 | 689.171220285288 | 1658.1807000183 |
| Female | 2038 | 1161.98879007829 | 635.717091116254 | 1688.26048904033 |
| Female | 2039 | 1150.42758480143 | 581.479812395728 | 1719.37535720713 |
| Female | 2040 | 1138.95688307283 | 526.497836934116 | 1751.41592921154 |
| Both | 1990 | 858.027269641954 | 857.304841815536 | 858.749697468372 |
| Both | 1991 | 856.051053014935 | 855.339960961399 | 856.762145068472 |
| Both | 1992 | 855.397987249347 | 854.695876068581 | 856.100098430114 |
| Both | 1993 | 860.481262463818 | 859.785397630178 | 861.177127297459 |
| Both | 1994 | 862.444094153465 | 861.755520949069 | 863.132667357862 |
| Both | 1995 | 858.115267200005 | 857.435828424199 | 858.794705975811 |
| Both | 1996 | 850.294743168808 | 849.625371544183 | 850.964114793432 |
| Both | 1997 | 841.68205891216 | 841.02308070959 | 842.341037114729 |
| Both | 1998 | 835.372819902688 | 834.722785987334 | 836.022853818041 |
| Both | 1999 | 835.356470492417 | 834.712858595297 | 836.000082389536 |
| Both | 2000 | 830.645141295288 | 830.009656499015 | 831.280626091561 |
| Both | 2001 | 824.435679538104 | 823.809368564913 | 825.061990511295 |
| Both | 2002 | 817.748518697805 | 817.132885738694 | 818.364151656916 |
| Both | 2003 | 812.628630086247 | 812.022998291238 | 813.234261881255 |
| Both | 2004 | 803.585764322045 | 802.992464138575 | 804.179064505514 |
| Both | 2005 | 799.012735735917 | 798.429176317016 | 799.596295154817 |
| Both | 2006 | 791.624441544674 | 791.052504784984 | 792.196378304363 |
| Both | 2007 | 785.575267735907 | 785.014447460526 | 786.136088011289 |
| Both | 2008 | 783.281505711043 | 782.730407374568 | 783.832604047518 |
| Both | 2009 | 777.011213694353 | 776.471312900073 | 777.551114488633 |
| Both | 2010 | 773.465075696129 | 772.93516039682 | 773.994990995439 |
| Both | 2011 | 768.353012574529 | 767.833251778508 | 768.87277337055 |
| Both | 2012 | 763.108259109334 | 762.598434905457 | 763.618083313211 |
| Both | 2013 | 758.212601462776 | 757.712055619098 | 758.713147306453 |
| Both | 2014 | 754.463104491567 | 753.971087069063 | 754.955121914072 |
| Both | 2015 | 756.779692299948 | 756.293899796074 | 757.265484803823 |
| Both | 2016 | 758.430948032099 | 757.951733073739 | 758.910162990458 |
| Both | 2017 | 756.934632383329 | 756.463249323583 | 757.406015443075 |
| Both | 2018 | 759.165139648758 | 758.7005892 | 759.629690141915 |
| Both | 2019 | 756.927973438773 | 756.471561019776 | 757.38438585777 |
| Both | 2020 | 746.748598051134 | 746.301876427965 | 747.195319674303 |
| Both | 2021 | 747.71138353304 | 747.269934934353 | 748.152832131726 |
| Both | 2022 | 740.215334035932 | 722.906886676195 | 757.523781395668 |
| Both | 2023 | 734.147246347132 | 708.869357625974 | 759.42513506829 |
| Both | 2024 | 728.12542241248 | 692.718644630327 | 763.532200194633 |
| Both | 2025 | 722.133476929021 | 674.974336008404 | 769.292617849638 |
| Both | 2026 | 716.234933346985 | 655.865223572562 | 776.604643121408 |
| Both | 2027 | 710.244031642297 | 635.268215472739 | 785.219847811855 |
| Both | 2028 | 704.329110309865 | 613.629987155088 | 795.028233464641 |
| Both | 2029 | 698.447310117578 | 591.061450913684 | 805.833169321471 |
| Both | 2030 | 692.593751485038 | 567.611312113541 | 817.576190856535 |
| Both | 2031 | 686.80370316421 | 543.286260603164 | 830.321145725255 |
| Both | 2032 | 680.918015456662 | 517.918535067029 | 843.917495846294 |
| Both | 2033 | 675.068985752499 | 491.759470408334 | 858.378501096664 |
| Both | 2034 | 669.247238849279 | 464.884642003279 | 873.609835695279 |
| Both | 2035 | 663.458173908929 | 437.331224885323 | 889.585122932535 |
| Both | 2036 | 657.731689673338 | 409.110152535649 | 906.353226811027 |
| Both | 2037 | 651.945031854409 | 380.115377885466 | 923.774685823351 |
| Both | 2038 | 646.197656641753 | 350.51184589283 | 941.883467390675 |
| Both | 2039 | 640.487338001616 | 320.359007104534 | 960.615668898699 |
| Both | 2040 | 634.810472751305 | 289.686111539676 | 979.934833962934 |

**Table S12. Detailed PAF results of DALYs**

| **Sex** | **Age** | **Risk Factor** | **PAF Percentage** | **PAF with 95CI** |
| --- | --- | --- | --- | --- |
| Male | 55-59 years | Behavioral risks | 19.89140975 | 19.9% (7.8% - 32.3%) |
| Male | 55-59 years | All risk factors | 19.89140975 | 19.9% (7.8% - 32.3%) |
| Male | 60-64 years | Behavioral risks | 19.39749242 | 19.4% (7.1% - 31.6%) |
| Male | 60-64 years | All risk factors | 19.39749242 | 19.4% (7.1% - 31.6%) |
| Male | 65-69 years | Behavioral risks | 18.92418112 | 18.9% (6.5% - 31.1%) |
| Male | 65-69 years | All risk factors | 18.92418112 | 18.9% (6.5% - 31.1%) |
| Male | 70-74 years | Behavioral risks | 18.55741698 | 18.6% (6.1% - 30.8%) |
| Male | 70-74 years | All risk factors | 18.55741698 | 18.6% (6.1% - 30.8%) |
| Male | 95+ years | All risk factors | 17.47685488 | 17.5% (4.1% - 30.3%) |
| Male | 95+ years | Behavioral risks | 17.47685488 | 17.5% (4.1% - 30.3%) |
| Male | 90-94 years | Behavioral risks | 17.36192886 | 17.4% (4.2% - 30.0%) |
| Male | 90-94 years | All risk factors | 17.36192886 | 17.4% (4.2% - 30.0%) |
| Male | 75-79 years | Behavioral risks | 17.20036288 | 17.2% (5.1% - 29.0%) |
| Male | 75-79 years | All risk factors | 17.20036288 | 17.2% (5.1% - 29.0%) |
| Male | 85-89 years | Behavioral risks | 17.00609278 | 17.0% (4.2% - 29.2%) |
| Male | 85-89 years | All risk factors | 17.00609278 | 17.0% (4.2% - 29.2%) |
| Male | 80-84 years | Behavioral risks | 16.52586475 | 16.5% (4.2% - 28.4%) |
| Male | 80-84 years | All risk factors | 16.52586475 | 16.5% (4.2% - 28.4%) |
| Male | 95+ years | Dietary risks | 12.03096169 | 12.0% (-0.0% - 25.7%) |
| Male | 95+ years | Diet high in red meat | 12.03096169 | 12.0% (-0.0% - 25.7%) |
| Male | 65-69 years | Dietary risks | 11.99958327 | 12.0% (-0.0% - 25.4%) |
| Male | 65-69 years | Diet high in red meat | 11.99958327 | 12.0% (-0.0% - 25.4%) |
| Male | 70-74 years | Dietary risks | 11.99063972 | 12.0% (-0.0% - 25.5%) |
| Male | 70-74 years | Diet high in red meat | 11.99063972 | 12.0% (-0.0% - 25.5%) |
| Male | 60-64 years | Dietary risks | 11.92481076 | 11.9% (-0.0% - 25.1%) |
| Male | 60-64 years | Diet high in red meat | 11.92481076 | 11.9% (-0.0% - 25.1%) |
| Male | 55-59 years | Dietary risks | 11.91763534 | 11.9% (-0.0% - 25.5%) |
| Male | 55-59 years | Diet high in red meat | 11.91763534 | 11.9% (-0.0% - 25.5%) |
| Male | 90-94 years | Dietary risks | 11.82324989 | 11.8% (-0.0% - 25.0%) |
| Male | 90-94 years | Diet high in red meat | 11.82324989 | 11.8% (-0.0% - 25.0%) |
| Male | 85-89 years | Dietary risks | 11.68245105 | 11.7% (-0.0% - 24.7%) |
| Male | 85-89 years | Diet high in red meat | 11.68245105 | 11.7% (-0.0% - 24.7%) |
| Male | 75-79 years | Dietary risks | 11.39432113 | 11.4% (-0.0% - 24.2%) |
| Male | 75-79 years | Diet high in red meat | 11.39432113 | 11.4% (-0.0% - 24.2%) |
| Male | 80-84 years | Dietary risks | 11.36045774 | 11.4% (-0.0% - 23.9%) |
| Male | 80-84 years | Diet high in red meat | 11.36045774 | 11.4% (-0.0% - 23.9%) |
| Male | 55-59 years | High alcohol use | 8.219350174 | 8.2% (5.6% - 11.0%) |
| Male | 60-64 years | High alcohol use | 7.713783855 | 7.7% (5.3% - 10.4%) |
| Male | 65-69 years | High alcohol use | 7.178911031 | 7.2% (4.8% - 9.7%) |
| Male | 70-74 years | High alcohol use | 6.774199677 | 6.8% (4.5% - 9.3%) |
| Male | 75-79 years | High alcohol use | 5.900607428 | 5.9% (3.8% - 8.0%) |
| Male | 90-94 years | High alcohol use | 5.632410607 | 5.6% (2.7% - 8.3%) |
| Male | 95+ years | High alcohol use | 5.533545356 | 5.5% (2.6% - 8.3%) |
| Male | 85-89 years | High alcohol use | 5.367949501 | 5.4% (3.0% - 7.8%) |
| Male | 80-84 years | High alcohol use | 5.192051692 | 5.2% (3.2% - 7.4%) |
| Male | 55-59 years | Secondhand smoke | 0.967479645 | 1.0% (-0.2% - 2.2%) |
| Male | 55-59 years | Tobacco | 0.967479645 | 1.0% (-0.2% - 2.2%) |
| Male | 60-64 years | Secondhand smoke | 0.890237727 | 0.9% (-0.2% - 2.0%) |
| Male | 60-64 years | Tobacco | 0.890237727 | 0.9% (-0.2% - 2.0%) |
| Male | 65-69 years | Secondhand smoke | 0.795599708 | 0.8% (-0.2% - 1.8%) |
| Male | 65-69 years | Tobacco | 0.795599708 | 0.8% (-0.2% - 1.8%) |
| Male | 70-74 years | Secondhand smoke | 0.788015056 | 0.8% (-0.2% - 1.8%) |
| Male | 70-74 years | Tobacco | 0.788015056 | 0.8% (-0.2% - 1.8%) |
| Male | 85-89 years | Secondhand smoke | 0.751532223 | 0.8% (-0.2% - 1.7%) |
| Male | 85-89 years | Tobacco | 0.751532223 | 0.8% (-0.2% - 1.7%) |
| Male | 75-79 years | Secondhand smoke | 0.750530844 | 0.8% (-0.2% - 1.7%) |
| Male | 75-79 years | Tobacco | 0.750530844 | 0.8% (-0.2% - 1.7%) |
| Male | 90-94 years | Secondhand smoke | 0.74020428 | 0.7% (-0.2% - 1.7%) |
| Male | 90-94 years | Tobacco | 0.74020428 | 0.7% (-0.2% - 1.7%) |
| Male | 95+ years | Secondhand smoke | 0.739027314 | 0.7% (-0.2% - 1.7%) |
| Male | 95+ years | Tobacco | 0.739027314 | 0.7% (-0.2% - 1.7%) |
| Male | 80-84 years | Secondhand smoke | 0.733307377 | 0.7% (-0.2% - 1.6%) |
| Male | 80-84 years | Tobacco | 0.733307377 | 0.7% (-0.2% - 1.6%) |
| Female | 75-79 years | All risk factors | 31.19250187 | 31.2% (9.3% - 48.3%) |
| Female | 70-74 years | All risk factors | 31.06110301 | 31.1% (9.2% - 47.8%) |
| Female | 90-94 years | All risk factors | 30.94844514 | 30.9% (9.5% - 48.2%) |
| Female | 85-89 years | All risk factors | 30.91762686 | 30.9% (9.2% - 48.2%) |
| Female | 65-69 years | All risk factors | 30.74112121 | 30.7% (9.1% - 47.1%) |
| Female | 60-64 years | All risk factors | 30.53948754 | 30.5% (9.7% - 46.5%) |
| Female | 95+ years | All risk factors | 30.52829247 | 30.5% (9.4% - 47.2%) |
| Female | 80-84 years | All risk factors | 30.35178208 | 30.4% (8.7% - 47.4%) |
| Female | 55-59 years | All risk factors | 29.24583872 | 29.2% (9.7% - 44.8%) |
| Female | 90-94 years | Behavioral risks | 20.31603742 | 20.3% (5.8% - 33.7%) |
| Female | 95+ years | Behavioral risks | 20.29811666 | 20.3% (5.5% - 34.0%) |
| Female | 85-89 years | Behavioral risks | 19.97190034 | 20.0% (5.7% - 33.3%) |
| Female | 75-79 years | Behavioral risks | 19.55378084 | 19.6% (5.9% - 32.6%) |
| Female | 80-84 years | Behavioral risks | 19.4289388 | 19.4% (5.4% - 32.5%) |
| Female | 70-74 years | Behavioral risks | 19.33114658 | 19.3% (6.1% - 32.1%) |
| Female | 60-64 years | Behavioral risks | 19.23311478 | 19.2% (6.3% - 31.8%) |
| Female | 65-69 years | Behavioral risks | 19.04854959 | 19.0% (6.0% - 31.7%) |
| Female | 55-59 years | Behavioral risks | 18.70731119 | 18.7% (6.0% - 31.1%) |
| Female | 70-74 years | Metabolic risks | 14.77166943 | 14.8% (-2.0% - 30.0%) |
| Female | 75-79 years | Metabolic risks | 14.69429664 | 14.7% (-2.0% - 30.1%) |
| Female | 65-69 years | Metabolic risks | 14.66857982 | 14.7% (-1.9% - 29.5%) |
| Female | 60-64 years | Metabolic risks | 14.21870496 | 14.2% (-1.7% - 28.4%) |
| Female | 85-89 years | Metabolic risks | 13.87984762 | 13.9% (-1.9% - 28.6%) |
| Female | 80-84 years | Metabolic risks | 13.77584365 | 13.8% (-1.9% - 28.4%) |
| Female | 90-94 years | Metabolic risks | 13.52879271 | 13.5% (-1.8% - 27.6%) |
| Female | 95+ years | Dietary risks | 13.20919545 | 13.2% (-0.0% - 28.1%) |
| Female | 95+ years | Diet high in red meat | 13.20919545 | 13.2% (-0.0% - 28.1%) |
| Female | 55-59 years | Metabolic risks | 13.16436944 | 13.2% (-1.6% - 26.6%) |
| Female | 90-94 years | Dietary risks | 13.10234638 | 13.1% (-0.0% - 27.9%) |
| Female | 90-94 years | Diet high in red meat | 13.10234638 | 13.1% (-0.0% - 27.9%) |
| Female | 95+ years | Metabolic risks | 13.00742046 | 13.0% (-1.6% - 26.5%) |
| Female | 85-89 years | Dietary risks | 12.90534811 | 12.9% (-0.0% - 27.5%) |
| Female | 85-89 years | Diet high in red meat | 12.90534811 | 12.9% (-0.0% - 27.5%) |
| Female | 80-84 years | Dietary risks | 12.60973376 | 12.6% (-0.0% - 26.7%) |
| Female | 80-84 years | Diet high in red meat | 12.60973376 | 12.6% (-0.0% - 26.7%) |
| Female | 75-79 years | Dietary risks | 12.60161298 | 12.6% (-0.0% - 26.9%) |
| Female | 75-79 years | Diet high in red meat | 12.60161298 | 12.6% (-0.0% - 26.9%) |
| Female | 70-74 years | Dietary risks | 12.43954894 | 12.4% (-0.0% - 26.5%) |
| Female | 70-74 years | Diet high in red meat | 12.43954894 | 12.4% (-0.0% - 26.5%) |
| Female | 65-69 years | Dietary risks | 12.30899502 | 12.3% (-0.0% - 26.5%) |
| Female | 65-69 years | Diet high in red meat | 12.30899502 | 12.3% (-0.0% - 26.5%) |
| Female | 60-64 years | Dietary risks | 12.20962834 | 12.2% (-0.0% - 26.1%) |
| Female | 60-64 years | Diet high in red meat | 12.20962834 | 12.2% (-0.0% - 26.1%) |
| Female | 55-59 years | Dietary risks | 11.85926269 | 11.9% (-0.0% - 25.4%) |
| Female | 55-59 years | Diet high in red meat | 11.85926269 | 11.9% (-0.0% - 25.4%) |
| Female | 60-64 years | High body-mass index | 9.929290354 | 9.9% (-0.3% - 19.4%) |
| Female | 65-69 years | High body-mass index | 9.819078985 | 9.8% (-0.3% - 19.3%) |
| Female | 70-74 years | High body-mass index | 9.620430377 | 9.6% (-0.3% - 18.9%) |
| Female | 55-59 years | High body-mass index | 9.470721357 | 9.5% (-0.3% - 18.5%) |
| Female | 75-79 years | High body-mass index | 9.362228911 | 9.4% (-0.3% - 18.7%) |
| Female | 95+ years | High body-mass index | 8.833705772 | 8.8% (-0.2% - 17.7%) |
| Female | 90-94 years | High body-mass index | 8.814101355 | 8.8% (-0.2% - 17.7%) |
| Female | 85-89 years | High body-mass index | 8.651528134 | 8.7% (-0.2% - 17.3%) |
| Female | 80-84 years | High body-mass index | 8.417134408 | 8.4% (-0.2% - 16.9%) |
| Female | 75-79 years | High fasting plasma glucose | 6.111596769 | 6.1% (-1.8% - 14.6%) |
| Female | 80-84 years | High fasting plasma glucose | 6.047501361 | 6.0% (-1.7% - 14.4%) |
| Female | 70-74 years | High fasting plasma glucose | 5.930377847 | 5.9% (-1.7% - 13.9%) |
| Female | 85-89 years | High fasting plasma glucose | 5.921330242 | 5.9% (-1.6% - 14.1%) |
| Female | 65-69 years | High fasting plasma glucose | 5.601890843 | 5.6% (-1.6% - 13.1%) |
| Female | 90-94 years | High fasting plasma glucose | 5.351538135 | 5.4% (-1.6% - 12.6%) |
| Female | 60-64 years | High fasting plasma glucose | 4.964728462 | 5.0% (-1.4% - 11.4%) |
| Female | 95+ years | High fasting plasma glucose | 4.737856181 | 4.7% (-1.4% - 11.0%) |
| Female | 55-59 years | High fasting plasma glucose | 4.243160404 | 4.2% (-1.3% - 9.9%) |
| Female | 95+ years | Low physical activity | 3.779012004 | 3.8% (0.8% - 6.6%) |
| Female | 90-94 years | Low physical activity | 3.776426509 | 3.8% (0.8% - 6.6%) |
| Female | 85-89 years | Low physical activity | 3.739194751 | 3.7% (0.8% - 6.5%) |
| Female | 80-84 years | Low physical activity | 3.683981112 | 3.7% (0.8% - 6.5%) |
| Female | 55-59 years | Tobacco | 3.344144793 | 3.3% (1.2% - 5.5%) |
| Female | 95+ years | High alcohol use | 3.249154409 | 3.2% (1.6% - 5.0%) |
| Female | 75-79 years | Low physical activity | 3.19876331 | 3.2% (0.6% - 5.7%) |
| Female | 90-94 years | High alcohol use | 3.132536357 | 3.1% (1.6% - 4.7%) |
| Female | 60-64 years | Tobacco | 3.092260992 | 3.1% (1.3% - 4.9%) |
| Female | 60-64 years | High alcohol use | 2.958802447 | 3.0% (2.0% - 4.2%) |
| Female | 70-74 years | Low physical activity | 2.839866198 | 2.8% (0.6% - 4.9%) |
| Female | 85-89 years | High alcohol use | 2.83 | 2.8% (1.6% - 4.1%) |
| Female | 70-74 years | High alcohol use | 2.800831455 | 2.8% (1.8% - 4.0%) |
| Female | 75-79 years | High alcohol use | 2.796924678 | 2.8% (1.8% - 4.0%) |
| Female | 65-69 years | Tobacco | 2.76867035 | 2.8% (1.1% - 4.5%) |
| Female | 55-59 years | High alcohol use | 2.736298305 | 2.7% (1.9% - 3.8%) |
| Female | 65-69 years | High alcohol use | 2.690277765 | 2.7% (1.8% - 3.9%) |
| Female | 80-84 years | High alcohol use | 2.510763042 | 2.5% (1.6% - 3.7%) |
| Female | 65-69 years | Low physical activity | 2.492273201 | 2.5% (0.5% - 4.5%) |
| Female | 70-74 years | Tobacco | 2.490071336 | 2.5% (1.1% - 3.9%) |
| Female | 60-64 years | Low physical activity | 2.248257034 | 2.2% (0.4% - 3.9%) |
| Female | 75-79 years | Tobacco | 2.211045081 | 2.2% (0.9% - 3.5%) |
| Female | 55-59 years | Low physical activity | 1.998322916 | 2.0% (0.4% - 3.5%) |
| Female | 55-59 years | Smoking | 1.974068684 | 2.0% (1.5% - 2.5%) |
| Female | 60-64 years | Smoking | 1.963601737 | 2.0% (1.5% - 2.5%) |
| Female | 80-84 years | Tobacco | 1.840239209 | 1.8% (0.7% - 3.0%) |
| Female | 85-89 years | Tobacco | 1.789236967 | 1.8% (0.7% - 2.9%) |
| Female | 65-69 years | Smoking | 1.77242408 | 1.8% (1.3% - 2.2%) |
| Female | 70-74 years | Smoking | 1.642362876 | 1.6% (1.2% - 2.1%) |
| Female | 90-94 years | Tobacco | 1.63700302 | 1.6% (0.7% - 2.6%) |
| Female | 75-79 years | Smoking | 1.442857776 | 1.4% (1.0% - 1.8%) |
| Female | 55-59 years | Secondhand smoke | 1.394328132 | 1.4% (-0.3% - 3.1%) |
| Female | 95+ years | Tobacco | 1.370248436 | 1.4% (0.5% - 2.2%) |
| Female | 60-64 years | Secondhand smoke | 1.15 | 1.1% (-0.3% - 2.5%) |
| Female | 80-84 years | Smoking | 1.146470258 | 1.1% (0.8% - 1.5%) |
| Female | 85-89 years | Smoking | 1.129884058 | 1.1% (0.8% - 1.5%) |
| Female | 90-94 years | Smoking | 1.07149924 | 1.1% (0.7% - 1.4%) |
| Female | 65-69 years | Secondhand smoke | 1.011540769 | 1.0% (-0.2% - 2.3%) |
| Female | 70-74 years | Secondhand smoke | 0.859574585 | 0.9% (-0.2% - 1.9%) |
| Female | 95+ years | Smoking | 0.858602271 | 0.9% (0.6% - 1.3%) |
| Female | 75-79 years | Secondhand smoke | 0.777586487 | 0.8% (-0.2% - 1.7%) |
| Female | 80-84 years | Secondhand smoke | 0.700909338 | 0.7% (-0.2% - 1.6%) |
| Female | 85-89 years | Secondhand smoke | 0.666014591 | 0.7% (-0.2% - 1.5%) |
| Female | 90-94 years | Secondhand smoke | 0.571001152 | 0.6% (-0.1% - 1.3%) |
| Female | 95+ years | Secondhand smoke | 0.51575472 | 0.5% (-0.1% - 1.2%) |
| Both | 75-79 years | All risk factors | 30.87597946 | 30.9% (9.3% - 47.8%) |
| Both | 90-94 years | All risk factors | 30.76790968 | 30.8% (9.5% - 47.9%) |
| Both | 70-74 years | All risk factors | 30.69774979 | 30.7% (9.3% - 47.1%) |
| Both | 85-89 years | All risk factors | 30.65377905 | 30.7% (9.2% - 47.8%) |
| Both | 65-69 years | All risk factors | 30.41952814 | 30.4% (9.2% - 46.4%) |
| Both | 95+ years | All risk factors | 30.40782851 | 30.4% (9.4% - 47.0%) |
| Both | 60-64 years | All risk factors | 30.27448942 | 30.3% (9.8% - 46.0%) |
| Both | 80-84 years | All risk factors | 30.07591511 | 30.1% (8.8% - 46.8%) |
| Both | 55-59 years | All risk factors | 29.07268858 | 29.1% (9.7% - 44.5%) |
| Both | 90-94 years | Behavioral risks | 20.27660996 | 20.3% (5.7% - 33.7%) |
| Both | 95+ years | Behavioral risks | 20.2719707 | 20.3% (5.5% - 34.0%) |
| Both | 85-89 years | Behavioral risks | 19.91542438 | 19.9% (5.7% - 33.2%) |
| Both | 75-79 years | Behavioral risks | 19.5001298 | 19.5% (5.9% - 32.5%) |
| Both | 80-84 years | Behavioral risks | 19.37069461 | 19.4% (5.4% - 32.4%) |
| Both | 70-74 years | Behavioral risks | 19.3086503 | 19.3% (6.1% - 32.1%) |
| Both | 60-64 years | Behavioral risks | 19.23701393 | 19.2% (6.3% - 31.8%) |
| Both | 65-69 years | Behavioral risks | 19.04507734 | 19.0% (6.0% - 31.7%) |
| Both | 55-59 years | Behavioral risks | 18.72917869 | 18.7% (6.0% - 31.1%) |
| Both | 75-79 years | Metabolic risks | 14.36268396 | 14.4% (-2.0% - 29.5%) |
| Both | 70-74 years | Metabolic risks | 14.3428238 | 14.3% (-1.9% - 29.1%) |
| Both | 65-69 years | Metabolic risks | 14.26987307 | 14.3% (-1.9% - 28.6%) |
| Both | 60-64 years | Metabolic risks | 13.88077919 | 13.9% (-1.7% - 27.8%) |
| Both | 85-89 years | Metabolic risks | 13.61713481 | 13.6% (-1.9% - 28.1%) |
| Both | 80-84 years | Metabolic risks | 13.50155627 | 13.5% (-1.9% - 27.9%) |
| Both | 90-94 years | Metabolic risks | 13.3493644 | 13.3% (-1.8% - 27.2%) |
| Both | 95+ years | Dietary risks | 13.19826409 | 13.2% (-0.0% - 28.0%) |
| Both | 95+ years | Diet high in red meat | 13.19826409 | 13.2% (-0.0% - 28.0%) |
| Both | 90-94 years | Dietary risks | 13.08528729 | 13.1% (-0.0% - 27.8%) |
| Both | 90-94 years | Diet high in red meat | 13.08528729 | 13.1% (-0.0% - 27.8%) |
| Both | 55-59 years | Metabolic risks | 12.92100499 | 12.9% (-1.5% - 26.1%) |
| Both | 95+ years | Metabolic risks | 12.88754309 | 12.9% (-1.6% - 26.2%) |
| Both | 85-89 years | Dietary risks | 12.88212156 | 12.9% (-0.0% - 27.4%) |
| Both | 85-89 years | Diet high in red meat | 12.88212156 | 12.9% (-0.0% - 27.4%) |
| Both | 80-84 years | Dietary risks | 12.58461794 | 12.6% (-0.0% - 26.7%) |
| Both | 80-84 years | Diet high in red meat | 12.58461794 | 12.6% (-0.0% - 26.7%) |
| Both | 75-79 years | Dietary risks | 12.5740713 | 12.6% (-0.0% - 26.8%) |
| Both | 75-79 years | Diet high in red meat | 12.5740713 | 12.6% (-0.0% - 26.8%) |
| Both | 70-74 years | Dietary risks | 12.42659432 | 12.4% (-0.0% - 26.5%) |
| Both | 70-74 years | Diet high in red meat | 12.42659432 | 12.4% (-0.0% - 26.5%) |
| Both | 65-69 years | Dietary risks | 12.30057934 | 12.3% (-0.0% - 26.5%) |
| Both | 65-69 years | Diet high in red meat | 12.30057934 | 12.3% (-0.0% - 26.5%) |
| Both | 60-64 years | Dietary risks | 12.20287332 | 12.2% (-0.0% - 26.1%) |
| Both | 60-64 years | Diet high in red meat | 12.20287332 | 12.2% (-0.0% - 26.1%) |
| Both | 55-59 years | Dietary risks | 11.86030874 | 11.9% (-0.0% - 25.4%) |
| Both | 55-59 years | Diet high in red meat | 11.86030874 | 11.9% (-0.0% - 25.4%) |
| Both | 60-64 years | High body-mass index | 9.693279155 | 9.7% (-0.3% - 18.9%) |
| Both | 65-69 years | High body-mass index | 9.552114959 | 9.6% (-0.3% - 18.7%) |
| Both | 70-74 years | High body-mass index | 9.341083873 | 9.3% (-0.3% - 18.4%) |
| Both | 55-59 years | High body-mass index | 9.295571901 | 9.3% (-0.3% - 18.2%) |
| Both | 75-79 years | High body-mass index | 9.150962315 | 9.2% (-0.3% - 18.3%) |
| Both | 95+ years | High body-mass index | 8.752329973 | 8.8% (-0.2% - 17.5%) |
| Both | 90-94 years | High body-mass index | 8.697266536 | 8.7% (-0.2% - 17.5%) |
| Both | 85-89 years | High body-mass index | 8.487868934 | 8.5% (-0.2% - 16.9%) |
| Both | 80-84 years | High body-mass index | 8.249571511 | 8.2% (-0.2% - 16.5%) |
| Both | 75-79 years | High fasting plasma glucose | 5.973741771 | 6.0% (-1.7% - 14.3%) |
| Both | 80-84 years | High fasting plasma glucose | 5.927125073 | 5.9% (-1.6% - 14.1%) |
| Both | 85-89 years | High fasting plasma glucose | 5.809201662 | 5.8% (-1.6% - 13.8%) |
| Both | 70-74 years | High fasting plasma glucose | 5.758349722 | 5.8% (-1.6% - 13.5%) |
| Both | 65-69 years | High fasting plasma glucose | 5.449778256 | 5.4% (-1.6% - 12.7%) |
| Both | 90-94 years | High fasting plasma glucose | 5.280527558 | 5.3% (-1.5% - 12.4%) |
| Both | 60-64 years | High fasting plasma glucose | 4.846813631 | 4.8% (-1.4% - 11.2%) |
| Both | 95+ years | High fasting plasma glucose | 4.694166768 | 4.7% (-1.4% - 10.9%) |
| Both | 55-59 years | High fasting plasma glucose | 4.164818351 | 4.2% (-1.3% - 9.7%) |
| Both | 95+ years | Low physical activity | 3.744157946 | 3.7% (0.8% - 6.6%) |
| Both | 90-94 years | Low physical activity | 3.72629947 | 3.7% (0.8% - 6.5%) |
| Both | 85-89 years | Low physical activity | 3.668357817 | 3.7% (0.8% - 6.4%) |
| Both | 80-84 years | Low physical activity | 3.610543981 | 3.6% (0.7% - 6.3%) |
| Both | 55-59 years | Tobacco | 3.300059031 | 3.3% (1.2% - 5.4%) |
| Both | 95+ years | High alcohol use | 3.270152344 | 3.3% (1.6% - 5.0%) |
| Both | 90-94 years | High alcohol use | 3.165560341 | 3.2% (1.7% - 4.7%) |
| Both | 75-79 years | Low physical activity | 3.126560398 | 3.1% (0.6% - 5.6%) |
| Both | 60-64 years | High alcohol use | 3.071863386 | 3.1% (2.1% - 4.3%) |
| Both | 60-64 years | Tobacco | 3.039865485 | 3.0% (1.2% - 4.8%) |
| Both | 70-74 years | High alcohol use | 2.916189011 | 2.9% (1.9% - 4.1%) |
| Both | 85-89 years | High alcohol use | 2.874503827 | 2.9% (1.7% - 4.2%) |
| Both | 75-79 years | High alcohol use | 2.866750867 | 2.9% (1.8% - 4.1%) |
| Both | 55-59 years | High alcohol use | 2.837813042 | 2.8% (2.0% - 3.9%) |
| Both | 65-69 years | High alcohol use | 2.812436695 | 2.8% (1.8% - 4.0%) |
| Both | 70-74 years | Low physical activity | 2.757372909 | 2.8% (0.5% - 4.8%) |
| Both | 65-69 years | Tobacco | 2.714843366 | 2.7% (1.1% - 4.4%) |
| Both | 80-84 years | High alcohol use | 2.564067042 | 2.6% (1.6% - 3.7%) |
| Both | 70-74 years | Tobacco | 2.44058456 | 2.4% (1.0% - 3.9%) |
| Both | 65-69 years | Low physical activity | 2.424479819 | 2.4% (0.5% - 4.3%) |
| Both | 60-64 years | Low physical activity | 2.19480947 | 2.2% (0.4% - 3.8%) |
| Both | 75-79 years | Tobacco | 2.178038709 | 2.2% (0.9% - 3.4%) |
| Both | 55-59 years | Low physical activity | 1.96137207 | 2.0% (0.4% - 3.4%) |
| Both | 55-59 years | Smoking | 1.937427398 | 1.9% (1.5% - 2.4%) |
| Both | 60-64 years | Smoking | 1.916859126 | 1.9% (1.4% - 2.4%) |
| Both | 80-84 years | Tobacco | 1.818143856 | 1.8% (0.7% - 2.9%) |
| Both | 85-89 years | Tobacco | 1.769575973 | 1.8% (0.7% - 2.9%) |
| Both | 65-69 years | Smoking | 1.724044096 | 1.7% (1.3% - 2.2%) |
| Both | 90-94 years | Tobacco | 1.625108377 | 1.6% (0.7% - 2.5%) |
| Both | 70-74 years | Smoking | 1.594559234 | 1.6% (1.2% - 2.1%) |
| Both | 75-79 years | Smoking | 1.410221564 | 1.4% (1.0% - 1.8%) |
| Both | 55-59 years | Secondhand smoke | 1.386432805 | 1.4% (-0.3% - 3.1%) |
| Both | 95+ years | Tobacco | 1.36442953 | 1.4% (0.5% - 2.2%) |
| Both | 60-64 years | Secondhand smoke | 1.141926325 | 1.1% (-0.3% - 2.5%) |
| Both | 80-84 years | Smoking | 1.123562386 | 1.1% (0.8% - 1.4%) |
| Both | 85-89 years | Smoking | 1.108451084 | 1.1% (0.8% - 1.4%) |
| Both | 90-94 years | Smoking | 1.057264656 | 1.1% (0.7% - 1.4%) |
| Both | 65-69 years | Secondhand smoke | 1.00567464 | 1.0% (-0.2% - 2.3%) |
| Both | 70-74 years | Secondhand smoke | 0.857545405 | 0.9% (-0.2% - 1.9%) |
| Both | 95+ years | Smoking | 0.85067266 | 0.9% (0.6% - 1.3%) |
| Both | 75-79 years | Secondhand smoke | 0.777003635 | 0.8% (-0.2% - 1.7%) |
| Both | 80-84 years | Secondhand smoke | 0.701579226 | 0.7% (-0.2% - 1.6%) |
| Both | 85-89 years | Secondhand smoke | 0.667660028 | 0.7% (-0.2% - 1.5%) |
| Both | 90-94 years | Secondhand smoke | 0.57326795 | 0.6% (-0.1% - 1.3%) |
| Both | 95+ years | Secondhand smoke | 0.51782741 | 0.5% (-0.1% - 1.2%) |

**Table S13. The prediction results of the BAPC model under different scenarios(ASPR)**

| **Model** | **Year** | **ASR** | **Lower** | **Upper** | **Category** |
| --- | --- | --- | --- | --- | --- |
| Primary | 1990 | 891.27 | 890.52 | 892.02 | Baseline |
| Primary | 1991 | 881.53 | 880.80 | 882.27 | Baseline |
| Primary | 1992 | 872.78 | 872.06 | 873.50 | Baseline |
| Primary | 1993 | 870.82 | 870.11 | 871.53 | Baseline |
| Primary | 1994 | 870.19 | 869.49 | 870.89 | Baseline |
| Primary | 1995 | 871.47 | 870.78 | 872.17 | Baseline |
| Primary | 1996 | 871.81 | 871.12 | 872.49 | Baseline |
| Primary | 1997 | 868.82 | 868.15 | 869.50 | Baseline |
| Primary | 1998 | 866.97 | 866.30 | 867.63 | Baseline |
| Primary | 1999 | 869.96 | 869.30 | 870.62 | Baseline |
| Primary | 2000 | 871.40 | 870.74 | 872.05 | Baseline |
| Primary | 2001 | 872.31 | 871.66 | 872.95 | Baseline |
| Primary | 2002 | 873.10 | 872.46 | 873.74 | Baseline |
| Primary | 2003 | 875.70 | 875.06 | 876.33 | Baseline |
| Primary | 2004 | 878.90 | 878.27 | 879.52 | Baseline |
| Primary | 2005 | 887.13 | 886.51 | 887.74 | Baseline |
| Primary | 2006 | 893.13 | 892.52 | 893.74 | Baseline |
| Primary | 2007 | 898.07 | 897.47 | 898.67 | Baseline |
| Primary | 2008 | 905.22 | 904.62 | 905.81 | Baseline |
| Primary | 2009 | 907.99 | 907.40 | 908.57 | Baseline |
| Primary | 2010 | 911.15 | 910.57 | 911.73 | Baseline |
| Primary | 2011 | 911.39 | 910.83 | 911.96 | Baseline |
| Primary | 2012 | 909.83 | 909.27 | 910.39 | Baseline |
| Primary | 2013 | 909.55 | 909.00 | 910.10 | Baseline |
| Primary | 2014 | 909.28 | 908.74 | 909.82 | Baseline |
| Primary | 2015 | 911.87 | 911.34 | 912.41 | Baseline |
| Primary | 2016 | 914.35 | 913.83 | 914.88 | Baseline |
| Primary | 2017 | 914.88 | 914.36 | 915.39 | Baseline |
| Primary | 2018 | 918.27 | 917.76 | 918.78 | Baseline |
| Primary | 2019 | 918.01 | 917.50 | 918.51 | Baseline |
| Primary | 2020 | 910.77 | 910.28 | 911.27 | Baseline |
| Primary | 2021 | 914.59 | 914.10 | 915.08 | Baseline |
| Primary | 2022 | 914.33 | 897.74 | 930.92 | Baseline |
| Primary | 2023 | 913.39 | 888.65 | 938.12 | Baseline |
| Primary | 2024 | 912.30 | 877.19 | 947.42 | Baseline |
| Primary | 2025 | 911.10 | 863.88 | 958.32 | Baseline |
| Primary | 2026 | 909.98 | 849.05 | 970.91 | Baseline |
| Primary | 2027 | 908.74 | 832.48 | 985.00 | Baseline |
| Primary | 2028 | 907.44 | 814.53 | 1,000.35 | Baseline |
| Primary | 2029 | 905.95 | 795.23 | 1,016.67 | Baseline |
| Primary | 2030 | 904.33 | 774.67 | 1,033.99 | Baseline |
| Primary | 2031 | 902.73 | 752.90 | 1,052.56 | Baseline |
| Primary | 2032 | 900.97 | 729.67 | 1,072.26 | Baseline |
| Primary | 2033 | 899.11 | 705.20 | 1,093.01 | Baseline |
| Primary | 2034 | 897.11 | 679.52 | 1,114.70 | Baseline |
| Primary | 2035 | 895.01 | 652.68 | 1,137.34 | Baseline |
| Primary | 2036 | 892.96 | 624.74 | 1,161.17 | Baseline |
| Primary | 2037 | 890.76 | 595.50 | 1,186.01 | Baseline |
| Primary | 2038 | 888.50 | 565.10 | 1,211.89 | Baseline |
| Primary | 2039 | 886.14 | 533.59 | 1,238.68 | Baseline |
| Primary | 2040 | 883.68 | 500.98 | 1,266.38 | Baseline |
| Sensitivity_RW1 | 1990 | 891.23 | 890.47 | 891.98 | Model_Sensitivity |
| Sensitivity_RW1 | 1991 | 881.53 | 880.80 | 882.27 | Model_Sensitivity |
| Sensitivity_RW1 | 1992 | 872.78 | 872.06 | 873.50 | Model_Sensitivity |
| Sensitivity_RW1 | 1993 | 870.82 | 870.11 | 871.53 | Model_Sensitivity |
| Sensitivity_RW1 | 1994 | 870.19 | 869.49 | 870.89 | Model_Sensitivity |
| Sensitivity_RW1 | 1995 | 871.49 | 870.79 | 872.18 | Model_Sensitivity |
| Sensitivity_RW1 | 1996 | 871.82 | 871.14 | 872.51 | Model_Sensitivity |
| Sensitivity_RW1 | 1997 | 868.82 | 868.15 | 869.50 | Model_Sensitivity |
| Sensitivity_RW1 | 1998 | 866.96 | 866.29 | 867.63 | Model_Sensitivity |
| Sensitivity_RW1 | 1999 | 869.96 | 869.30 | 870.62 | Model_Sensitivity |
| Sensitivity_RW1 | 2000 | 871.40 | 870.74 | 872.05 | Model_Sensitivity |
| Sensitivity_RW1 | 2001 | 872.31 | 871.66 | 872.95 | Model_Sensitivity |
| Sensitivity_RW1 | 2002 | 873.10 | 872.46 | 873.74 | Model_Sensitivity |
| Sensitivity_RW1 | 2003 | 875.69 | 875.06 | 876.33 | Model_Sensitivity |
| Sensitivity_RW1 | 2004 | 878.89 | 878.27 | 879.52 | Model_Sensitivity |
| Sensitivity_RW1 | 2005 | 887.13 | 886.51 | 887.75 | Model_Sensitivity |
| Sensitivity_RW1 | 2006 | 893.13 | 892.52 | 893.75 | Model_Sensitivity |
| Sensitivity_RW1 | 2007 | 898.07 | 897.47 | 898.67 | Model_Sensitivity |
| Sensitivity_RW1 | 2008 | 905.22 | 904.62 | 905.81 | Model_Sensitivity |
| Sensitivity_RW1 | 2009 | 907.98 | 907.39 | 908.57 | Model_Sensitivity |
| Sensitivity_RW1 | 2010 | 911.15 | 910.57 | 911.73 | Model_Sensitivity |
| Sensitivity_RW1 | 2011 | 911.39 | 910.82 | 911.96 | Model_Sensitivity |
| Sensitivity_RW1 | 2012 | 909.83 | 909.27 | 910.39 | Model_Sensitivity |
| Sensitivity_RW1 | 2013 | 909.55 | 909.00 | 910.10 | Model_Sensitivity |
| Sensitivity_RW1 | 2014 | 909.28 | 908.73 | 909.82 | Model_Sensitivity |
| Sensitivity_RW1 | 2015 | 911.87 | 911.34 | 912.41 | Model_Sensitivity |
| Sensitivity_RW1 | 2016 | 914.35 | 913.82 | 914.88 | Model_Sensitivity |
| Sensitivity_RW1 | 2017 | 914.87 | 914.35 | 915.39 | Model_Sensitivity |
| Sensitivity_RW1 | 2018 | 918.27 | 917.76 | 918.79 | Model_Sensitivity |
| Sensitivity_RW1 | 2019 | 918.01 | 917.50 | 918.51 | Model_Sensitivity |
| Sensitivity_RW1 | 2020 | 910.77 | 910.28 | 911.27 | Model_Sensitivity |
| Sensitivity_RW1 | 2021 | 914.60 | 914.11 | 915.09 | Model_Sensitivity |
| Sensitivity_RW1 | 2022 | 921.81 | 905.88 | 937.74 | Model_Sensitivity |
| Sensitivity_RW1 | 2023 | 926.90 | 907.54 | 946.27 | Model_Sensitivity |
| Sensitivity_RW1 | 2024 | 931.21 | 908.96 | 953.46 | Model_Sensitivity |
| Sensitivity_RW1 | 2025 | 933.84 | 909.04 | 958.65 | Model_Sensitivity |
| Sensitivity_RW1 | 2026 | 938.07 | 910.66 | 965.47 | Model_Sensitivity |
| Sensitivity_RW1 | 2027 | 941.43 | 909.96 | 972.91 | Model_Sensitivity |
| Sensitivity_RW1 | 2028 | 945.58 | 910.63 | 980.53 | Model_Sensitivity |
| Sensitivity_RW1 | 2029 | 949.00 | 910.90 | 987.11 | Model_Sensitivity |
| Sensitivity_RW1 | 2030 | 950.93 | 909.88 | 991.99 | Model_Sensitivity |
| Sensitivity_RW1 | 2031 | 954.28 | 910.18 | 998.38 | Model_Sensitivity |
| Sensitivity_RW1 | 2032 | 956.57 | 908.18 | 1,004.97 | Model_Sensitivity |
| Sensitivity_RW1 | 2033 | 959.51 | 907.22 | 1,011.79 | Model_Sensitivity |
| Sensitivity_RW1 | 2034 | 961.94 | 906.03 | 1,017.84 | Model_Sensitivity |
| Sensitivity_RW1 | 2035 | 963.09 | 903.76 | 1,022.43 | Model_Sensitivity |
| Sensitivity_RW1 | 2036 | 965.49 | 902.65 | 1,028.34 | Model_Sensitivity |
| Sensitivity_RW1 | 2037 | 966.86 | 899.61 | 1,034.11 | Model_Sensitivity |
| Sensitivity_RW1 | 2038 | 968.83 | 897.46 | 1,040.19 | Model_Sensitivity |
| Sensitivity_RW1 | 2039 | 970.51 | 895.25 | 1,045.77 | Model_Sensitivity |
| Sensitivity_RW1 | 2040 | 971.00 | 892.01 | 1,049.98 | Model_Sensitivity |
| Sensitivity_InformativePrior | 1990 | 891.28 | 890.53 | 892.04 | Model_Sensitivity |
| Sensitivity_InformativePrior | 1991 | 881.53 | 880.79 | 882.27 | Model_Sensitivity |
| Sensitivity_InformativePrior | 1992 | 872.77 | 872.05 | 873.49 | Model_Sensitivity |
| Sensitivity_InformativePrior | 1993 | 870.81 | 870.09 | 871.52 | Model_Sensitivity |
| Sensitivity_InformativePrior | 1994 | 870.19 | 869.49 | 870.89 | Model_Sensitivity |
| Sensitivity_InformativePrior | 1995 | 871.48 | 870.79 | 872.17 | Model_Sensitivity |
| Sensitivity_InformativePrior | 1996 | 871.82 | 871.14 | 872.51 | Model_Sensitivity |
| Sensitivity_InformativePrior | 1997 | 868.82 | 868.15 | 869.50 | Model_Sensitivity |
| Sensitivity_InformativePrior | 1998 | 866.96 | 866.29 | 867.63 | Model_Sensitivity |
| Sensitivity_InformativePrior | 1999 | 869.96 | 869.30 | 870.62 | Model_Sensitivity |
| Sensitivity_InformativePrior | 2000 | 871.40 | 870.74 | 872.05 | Model_Sensitivity |
| Sensitivity_InformativePrior | 2001 | 872.31 | 871.66 | 872.96 | Model_Sensitivity |
| Sensitivity_InformativePrior | 2002 | 873.10 | 872.46 | 873.74 | Model_Sensitivity |
| Sensitivity_InformativePrior | 2003 | 875.69 | 875.06 | 876.32 | Model_Sensitivity |
| Sensitivity_InformativePrior | 2004 | 878.89 | 878.27 | 879.51 | Model_Sensitivity |
| Sensitivity_InformativePrior | 2005 | 887.13 | 886.51 | 887.74 | Model_Sensitivity |
| Sensitivity_InformativePrior | 2006 | 893.13 | 892.52 | 893.74 | Model_Sensitivity |
| Sensitivity_InformativePrior | 2007 | 898.07 | 897.47 | 898.68 | Model_Sensitivity |
| Sensitivity_InformativePrior | 2008 | 905.22 | 904.63 | 905.82 | Model_Sensitivity |
| Sensitivity_InformativePrior | 2009 | 907.99 | 907.40 | 908.58 | Model_Sensitivity |
| Sensitivity_InformativePrior | 2010 | 911.16 | 910.58 | 911.74 | Model_Sensitivity |
| Sensitivity_InformativePrior | 2011 | 911.40 | 910.83 | 911.97 | Model_Sensitivity |
| Sensitivity_InformativePrior | 2012 | 909.83 | 909.27 | 910.39 | Model_Sensitivity |
| Sensitivity_InformativePrior | 2013 | 909.54 | 908.99 | 910.10 | Model_Sensitivity |
| Sensitivity_InformativePrior | 2014 | 909.27 | 908.73 | 909.82 | Model_Sensitivity |
| Sensitivity_InformativePrior | 2015 | 911.87 | 911.34 | 912.41 | Model_Sensitivity |
| Sensitivity_InformativePrior | 2016 | 914.36 | 913.83 | 914.88 | Model_Sensitivity |
| Sensitivity_InformativePrior | 2017 | 914.88 | 914.36 | 915.40 | Model_Sensitivity |
| Sensitivity_InformativePrior | 2018 | 918.28 | 917.76 | 918.79 | Model_Sensitivity |
| Sensitivity_InformativePrior | 2019 | 918.01 | 917.50 | 918.51 | Model_Sensitivity |
| Sensitivity_InformativePrior | 2020 | 910.77 | 910.27 | 911.26 | Model_Sensitivity |
| Sensitivity_InformativePrior | 2021 | 914.59 | 914.10 | 915.08 | Model_Sensitivity |
| Sensitivity_InformativePrior | 2022 | 916.59 | 891.23 | 941.96 | Model_Sensitivity |
| Sensitivity_InformativePrior | 2023 | 917.96 | 868.85 | 967.06 | Model_Sensitivity |
| Sensitivity_InformativePrior | 2024 | 918.98 | 840.59 | 997.37 | Model_Sensitivity |
| Sensitivity_InformativePrior | 2025 | 919.53 | 807.50 | 1,031.56 | Model_Sensitivity |
| Sensitivity_InformativePrior | 2026 | 920.35 | 770.68 | 1,070.01 | Model_Sensitivity |
| Sensitivity_InformativePrior | 2027 | 921.17 | 729.73 | 1,112.61 | Model_Sensitivity |
| Sensitivity_InformativePrior | 2028 | 922.15 | 685.31 | 1,158.99 | Model_Sensitivity |
| Sensitivity_InformativePrior | 2029 | 922.81 | 637.38 | 1,208.25 | Model_Sensitivity |
| Sensitivity_InformativePrior | 2030 | 923.10 | 586.17 | 1,260.03 | Model_Sensitivity |
| Sensitivity_InformativePrior | 2031 | 923.56 | 532.09 | 1,315.02 | Model_Sensitivity |
| Sensitivity_InformativePrior | 2032 | 923.88 | 474.72 | 1,373.03 | Model_Sensitivity |
| Sensitivity_InformativePrior | 2033 | 924.28 | 414.42 | 1,434.14 | Model_Sensitivity |
| Sensitivity_InformativePrior | 2034 | 924.47 | 351.23 | 1,497.71 | Model_Sensitivity |
| Sensitivity_InformativePrior | 2035 | 924.38 | 285.27 | 1,563.49 | Model_Sensitivity |
| Sensitivity_InformativePrior | 2036 | 924.45 | 216.71 | 1,632.18 | Model_Sensitivity |
| Sensitivity_InformativePrior | 2037 | 924.35 | 145.29 | 1,703.42 | Model_Sensitivity |
| Sensitivity_InformativePrior | 2038 | 924.35 | 71.21 | 1,777.48 | Model_Sensitivity |
| Sensitivity_InformativePrior | 2039 | 924.22 | -5.43 | 1,853.87 | Model_Sensitivity |
| Sensitivity_InformativePrior | 2040 | 923.81 | -84.57 | 1,932.19 | Model_Sensitivity |
| Sensitivity_NoCohort | 1990 | 891.27 | 890.51 | 892.02 | Model_Sensitivity |
| Sensitivity_NoCohort | 1991 | 881.52 | 880.78 | 882.26 | Model_Sensitivity |
| Sensitivity_NoCohort | 1992 | 872.75 | 872.02 | 873.47 | Model_Sensitivity |
| Sensitivity_NoCohort | 1993 | 870.78 | 870.07 | 871.50 | Model_Sensitivity |
| Sensitivity_NoCohort | 1994 | 870.15 | 869.44 | 870.85 | Model_Sensitivity |
| Sensitivity_NoCohort | 1995 | 871.45 | 870.76 | 872.15 | Model_Sensitivity |
| Sensitivity_NoCohort | 1996 | 871.81 | 871.13 | 872.50 | Model_Sensitivity |
| Sensitivity_NoCohort | 1997 | 868.82 | 868.14 | 869.50 | Model_Sensitivity |
| Sensitivity_NoCohort | 1998 | 866.96 | 866.29 | 867.63 | Model_Sensitivity |
| Sensitivity_NoCohort | 1999 | 869.97 | 869.31 | 870.64 | Model_Sensitivity |
| Sensitivity_NoCohort | 2000 | 871.41 | 870.75 | 872.06 | Model_Sensitivity |
| Sensitivity_NoCohort | 2001 | 872.32 | 871.67 | 872.97 | Model_Sensitivity |
| Sensitivity_NoCohort | 2002 | 873.11 | 872.47 | 873.75 | Model_Sensitivity |
| Sensitivity_NoCohort | 2003 | 875.70 | 875.07 | 876.33 | Model_Sensitivity |
| Sensitivity_NoCohort | 2004 | 878.89 | 878.26 | 879.51 | Model_Sensitivity |
| Sensitivity_NoCohort | 2005 | 887.12 | 886.50 | 887.74 | Model_Sensitivity |
| Sensitivity_NoCohort | 2006 | 893.12 | 892.51 | 893.74 | Model_Sensitivity |
| Sensitivity_NoCohort | 2007 | 898.06 | 897.46 | 898.67 | Model_Sensitivity |
| Sensitivity_NoCohort | 2008 | 905.22 | 904.62 | 905.81 | Model_Sensitivity |
| Sensitivity_NoCohort | 2009 | 907.98 | 907.39 | 908.57 | Model_Sensitivity |
| Sensitivity_NoCohort | 2010 | 911.15 | 910.57 | 911.73 | Model_Sensitivity |
| Sensitivity_NoCohort | 2011 | 911.39 | 910.82 | 911.96 | Model_Sensitivity |
| Sensitivity_NoCohort | 2012 | 909.82 | 909.26 | 910.39 | Model_Sensitivity |
| Sensitivity_NoCohort | 2013 | 909.54 | 908.99 | 910.09 | Model_Sensitivity |
| Sensitivity_NoCohort | 2014 | 909.27 | 908.73 | 909.82 | Model_Sensitivity |
| Sensitivity_NoCohort | 2015 | 911.88 | 911.34 | 912.42 | Model_Sensitivity |
| Sensitivity_NoCohort | 2016 | 914.36 | 913.84 | 914.89 | Model_Sensitivity |
| Sensitivity_NoCohort | 2017 | 914.88 | 914.36 | 915.40 | Model_Sensitivity |
| Sensitivity_NoCohort | 2018 | 918.28 | 917.77 | 918.79 | Model_Sensitivity |
| Sensitivity_NoCohort | 2019 | 918.01 | 917.51 | 918.52 | Model_Sensitivity |
| Sensitivity_NoCohort | 2020 | 910.77 | 910.27 | 911.26 | Model_Sensitivity |
| Sensitivity_NoCohort | 2021 | 914.59 | 914.10 | 915.08 | Model_Sensitivity |
| Sensitivity_NoCohort | 2022 | 876.63 | 824.01 | 929.24 | Model_Sensitivity |
| Sensitivity_NoCohort | 2023 | 874.31 | 812.92 | 935.70 | Model_Sensitivity |
| Sensitivity_NoCohort | 2024 | 872.00 | 799.00 | 945.00 | Model_Sensitivity |
| Sensitivity_NoCohort | 2025 | 869.70 | 782.72 | 956.68 | Model_Sensitivity |
| Sensitivity_NoCohort | 2026 | 867.40 | 764.47 | 970.33 | Model_Sensitivity |
| Sensitivity_NoCohort | 2027 | 865.11 | 744.57 | 985.65 | Model_Sensitivity |
| Sensitivity_NoCohort | 2028 | 862.83 | 723.25 | 1,002.40 | Model_Sensitivity |
| Sensitivity_NoCohort | 2029 | 860.55 | 700.69 | 1,020.40 | Model_Sensitivity |
| Sensitivity_NoCohort | 2030 | 858.27 | 677.01 | 1,039.54 | Model_Sensitivity |
| Sensitivity_NoCohort | 2031 | 856.01 | 652.32 | 1,059.69 | Model_Sensitivity |
| Sensitivity_NoCohort | 2032 | 853.75 | 626.70 | 1,080.80 | Model_Sensitivity |
| Sensitivity_NoCohort | 2033 | 851.49 | 600.21 | 1,102.78 | Model_Sensitivity |
| Sensitivity_NoCohort | 2034 | 849.24 | 572.91 | 1,125.58 | Model_Sensitivity |
| Sensitivity_NoCohort | 2035 | 847.00 | 544.86 | 1,149.15 | Model_Sensitivity |
| Sensitivity_NoCohort | 2036 | 844.77 | 516.08 | 1,173.45 | Model_Sensitivity |
| Sensitivity_NoCohort | 2037 | 842.54 | 486.62 | 1,198.45 | Model_Sensitivity |
| Sensitivity_NoCohort | 2038 | 840.31 | 456.51 | 1,224.11 | Model_Sensitivity |
| Sensitivity_NoCohort | 2039 | 838.09 | 425.78 | 1,250.40 | Model_Sensitivity |
| Sensitivity_NoCohort | 2040 | 835.88 | 394.46 | 1,277.30 | Model_Sensitivity |
| Scenario_Treatment | 1990 | 891.27 | 890.52 | 892.02 | Intervention_Scenarios |
| Scenario_Treatment | 1991 | 881.53 | 880.80 | 882.27 | Intervention_Scenarios |
| Scenario_Treatment | 1992 | 872.78 | 872.06 | 873.50 | Intervention_Scenarios |
| Scenario_Treatment | 1993 | 870.82 | 870.11 | 871.53 | Intervention_Scenarios |
| Scenario_Treatment | 1994 | 870.19 | 869.49 | 870.89 | Intervention_Scenarios |
| Scenario_Treatment | 1995 | 871.47 | 870.78 | 872.17 | Intervention_Scenarios |
| Scenario_Treatment | 1996 | 871.81 | 871.12 | 872.49 | Intervention_Scenarios |
| Scenario_Treatment | 1997 | 868.82 | 868.15 | 869.50 | Intervention_Scenarios |
| Scenario_Treatment | 1998 | 866.97 | 866.30 | 867.63 | Intervention_Scenarios |
| Scenario_Treatment | 1999 | 869.96 | 869.30 | 870.62 | Intervention_Scenarios |
| Scenario_Treatment | 2000 | 871.40 | 870.74 | 872.05 | Intervention_Scenarios |
| Scenario_Treatment | 2001 | 872.31 | 871.66 | 872.95 | Intervention_Scenarios |
| Scenario_Treatment | 2002 | 873.10 | 872.46 | 873.74 | Intervention_Scenarios |
| Scenario_Treatment | 2003 | 875.70 | 875.06 | 876.33 | Intervention_Scenarios |
| Scenario_Treatment | 2004 | 878.90 | 878.27 | 879.52 | Intervention_Scenarios |
| Scenario_Treatment | 2005 | 887.13 | 886.51 | 887.74 | Intervention_Scenarios |
| Scenario_Treatment | 2006 | 893.13 | 892.52 | 893.74 | Intervention_Scenarios |
| Scenario_Treatment | 2007 | 898.07 | 897.47 | 898.67 | Intervention_Scenarios |
| Scenario_Treatment | 2008 | 905.22 | 904.62 | 905.81 | Intervention_Scenarios |
| Scenario_Treatment | 2009 | 907.99 | 907.40 | 908.57 | Intervention_Scenarios |
| Scenario_Treatment | 2010 | 911.15 | 910.57 | 911.73 | Intervention_Scenarios |
| Scenario_Treatment | 2011 | 911.39 | 910.83 | 911.96 | Intervention_Scenarios |
| Scenario_Treatment | 2012 | 909.83 | 909.27 | 910.39 | Intervention_Scenarios |
| Scenario_Treatment | 2013 | 909.55 | 909.00 | 910.10 | Intervention_Scenarios |
| Scenario_Treatment | 2014 | 909.28 | 908.74 | 909.82 | Intervention_Scenarios |
| Scenario_Treatment | 2015 | 911.87 | 911.34 | 912.41 | Intervention_Scenarios |
| Scenario_Treatment | 2016 | 914.35 | 913.83 | 914.88 | Intervention_Scenarios |
| Scenario_Treatment | 2017 | 914.88 | 914.36 | 915.39 | Intervention_Scenarios |
| Scenario_Treatment | 2018 | 918.27 | 917.76 | 918.78 | Intervention_Scenarios |
| Scenario_Treatment | 2019 | 918.01 | 917.50 | 918.51 | Intervention_Scenarios |
| Scenario_Treatment | 2020 | 910.77 | 910.28 | 911.27 | Intervention_Scenarios |
| Scenario_Treatment | 2021 | 914.59 | 914.10 | 915.08 | Intervention_Scenarios |
| Scenario_Treatment | 2022 | 914.33 | 897.74 | 930.92 | Intervention_Scenarios |
| Scenario_Treatment | 2023 | 913.39 | 888.65 | 938.12 | Intervention_Scenarios |
| Scenario_Treatment | 2024 | 912.30 | 877.19 | 947.42 | Intervention_Scenarios |
| Scenario_Treatment | 2025 | 747.10 | 708.38 | 785.82 | Intervention_Scenarios |
| Scenario_Treatment | 2026 | 746.18 | 696.22 | 796.15 | Intervention_Scenarios |
| Scenario_Treatment | 2027 | 745.17 | 682.63 | 807.70 | Intervention_Scenarios |
| Scenario_Treatment | 2028 | 744.10 | 667.92 | 820.29 | Intervention_Scenarios |
| Scenario_Treatment | 2029 | 742.88 | 652.09 | 833.67 | Intervention_Scenarios |
| Scenario_Treatment | 2030 | 741.55 | 635.23 | 847.87 | Intervention_Scenarios |
| Scenario_Treatment | 2031 | 740.24 | 617.38 | 863.10 | Intervention_Scenarios |
| Scenario_Treatment | 2032 | 738.79 | 598.33 | 879.25 | Intervention_Scenarios |
| Scenario_Treatment | 2033 | 737.27 | 578.26 | 896.27 | Intervention_Scenarios |
| Scenario_Treatment | 2034 | 735.63 | 557.20 | 914.05 | Intervention_Scenarios |
| Scenario_Treatment | 2035 | 733.91 | 535.20 | 932.62 | Intervention_Scenarios |
| Scenario_Treatment | 2036 | 732.22 | 512.29 | 952.16 | Intervention_Scenarios |
| Scenario_Treatment | 2037 | 730.42 | 488.31 | 972.53 | Intervention_Scenarios |
| Scenario_Treatment | 2038 | 728.57 | 463.38 | 993.75 | Intervention_Scenarios |
| Scenario_Treatment | 2039 | 726.63 | 437.55 | 1,015.72 | Intervention_Scenarios |
| Scenario_Treatment | 2040 | 724.62 | 410.81 | 1,038.44 | Intervention_Scenarios |
| Scenario_Screening | 1990 | 891.27 | 890.52 | 892.02 | Intervention_Scenarios |
| Scenario_Screening | 1991 | 881.53 | 880.80 | 882.27 | Intervention_Scenarios |
| Scenario_Screening | 1992 | 872.78 | 872.06 | 873.50 | Intervention_Scenarios |
| Scenario_Screening | 1993 | 870.82 | 870.11 | 871.53 | Intervention_Scenarios |
| Scenario_Screening | 1994 | 870.19 | 869.49 | 870.89 | Intervention_Scenarios |
| Scenario_Screening | 1995 | 871.47 | 870.78 | 872.17 | Intervention_Scenarios |
| Scenario_Screening | 1996 | 871.81 | 871.12 | 872.49 | Intervention_Scenarios |
| Scenario_Screening | 1997 | 868.82 | 868.15 | 869.50 | Intervention_Scenarios |
| Scenario_Screening | 1998 | 866.97 | 866.30 | 867.63 | Intervention_Scenarios |
| Scenario_Screening | 1999 | 869.96 | 869.30 | 870.62 | Intervention_Scenarios |
| Scenario_Screening | 2000 | 871.40 | 870.74 | 872.05 | Intervention_Scenarios |
| Scenario_Screening | 2001 | 872.31 | 871.66 | 872.95 | Intervention_Scenarios |
| Scenario_Screening | 2002 | 873.10 | 872.46 | 873.74 | Intervention_Scenarios |
| Scenario_Screening | 2003 | 875.70 | 875.06 | 876.33 | Intervention_Scenarios |
| Scenario_Screening | 2004 | 878.90 | 878.27 | 879.52 | Intervention_Scenarios |
| Scenario_Screening | 2005 | 887.13 | 886.51 | 887.74 | Intervention_Scenarios |
| Scenario_Screening | 2006 | 893.13 | 892.52 | 893.74 | Intervention_Scenarios |
| Scenario_Screening | 2007 | 898.07 | 897.47 | 898.67 | Intervention_Scenarios |
| Scenario_Screening | 2008 | 905.22 | 904.62 | 905.81 | Intervention_Scenarios |
| Scenario_Screening | 2009 | 907.99 | 907.40 | 908.57 | Intervention_Scenarios |
| Scenario_Screening | 2010 | 911.15 | 910.57 | 911.73 | Intervention_Scenarios |
| Scenario_Screening | 2011 | 911.39 | 910.83 | 911.96 | Intervention_Scenarios |
| Scenario_Screening | 2012 | 909.83 | 909.27 | 910.39 | Intervention_Scenarios |
| Scenario_Screening | 2013 | 909.55 | 909.00 | 910.10 | Intervention_Scenarios |
| Scenario_Screening | 2014 | 909.28 | 908.74 | 909.82 | Intervention_Scenarios |
| Scenario_Screening | 2015 | 911.87 | 911.34 | 912.41 | Intervention_Scenarios |
| Scenario_Screening | 2016 | 914.35 | 913.83 | 914.88 | Intervention_Scenarios |
| Scenario_Screening | 2017 | 914.88 | 914.36 | 915.39 | Intervention_Scenarios |
| Scenario_Screening | 2018 | 918.27 | 917.76 | 918.78 | Intervention_Scenarios |
| Scenario_Screening | 2019 | 918.01 | 917.50 | 918.51 | Intervention_Scenarios |
| Scenario_Screening | 2020 | 910.77 | 910.28 | 911.27 | Intervention_Scenarios |
| Scenario_Screening | 2021 | 914.59 | 914.10 | 915.08 | Intervention_Scenarios |
| Scenario_Screening | 2022 | 914.33 | 897.74 | 930.92 | Intervention_Scenarios |
| Scenario_Screening | 2023 | 913.39 | 888.65 | 938.12 | Intervention_Scenarios |
| Scenario_Screening | 2024 | 912.30 | 877.19 | 947.42 | Intervention_Scenarios |
| Scenario_Screening | 2025 | 898.57 | 852.00 | 945.14 | Intervention_Scenarios |
| Scenario_Screening | 2026 | 884.96 | 825.70 | 944.21 | Intervention_Scenarios |
| Scenario_Screening | 2027 | 871.26 | 798.14 | 944.37 | Intervention_Scenarios |
| Scenario_Screening | 2028 | 857.53 | 769.73 | 945.33 | Intervention_Scenarios |
| Scenario_Screening | 2029 | 843.67 | 740.56 | 946.78 | Intervention_Scenarios |
| Scenario_Screening | 2030 | 829.72 | 710.76 | 948.69 | Intervention_Scenarios |
| Scenario_Screening | 2031 | 815.84 | 680.44 | 951.25 | Intervention_Scenarios |
| Scenario_Screening | 2032 | 801.86 | 649.41 | 954.31 | Intervention_Scenarios |
| Scenario_Screening | 2033 | 787.84 | 617.93 | 957.75 | Intervention_Scenarios |
| Scenario_Screening | 2034 | 773.76 | 586.08 | 961.43 | Intervention_Scenarios |
| Scenario_Screening | 2035 | 759.64 | 553.96 | 965.32 | Intervention_Scenarios |
| Scenario_Screening | 2036 | 745.62 | 521.66 | 969.57 | Intervention_Scenarios |
| Scenario_Screening | 2037 | 731.53 | 489.05 | 974.01 | Intervention_Scenarios |
| Scenario_Screening | 2038 | 717.46 | 456.32 | 978.60 | Intervention_Scenarios |
| Scenario_Screening | 2039 | 703.37 | 423.54 | 983.20 | Intervention_Scenarios |
| Scenario_Screening | 2040 | 689.27 | 390.77 | 987.78 | Intervention_Scenarios |
| Scenario_Lifestyle | 1990 | 891.27 | 890.52 | 892.02 | Intervention_Scenarios |
| Scenario_Lifestyle | 1991 | 881.53 | 880.80 | 882.27 | Intervention_Scenarios |
| Scenario_Lifestyle | 1992 | 872.78 | 872.06 | 873.50 | Intervention_Scenarios |
| Scenario_Lifestyle | 1993 | 870.82 | 870.11 | 871.53 | Intervention_Scenarios |
| Scenario_Lifestyle | 1994 | 870.19 | 869.49 | 870.89 | Intervention_Scenarios |
| Scenario_Lifestyle | 1995 | 871.47 | 870.78 | 872.17 | Intervention_Scenarios |
| Scenario_Lifestyle | 1996 | 871.81 | 871.12 | 872.49 | Intervention_Scenarios |
| Scenario_Lifestyle | 1997 | 868.82 | 868.15 | 869.50 | Intervention_Scenarios |
| Scenario_Lifestyle | 1998 | 866.97 | 866.30 | 867.63 | Intervention_Scenarios |
| Scenario_Lifestyle | 1999 | 869.96 | 869.30 | 870.62 | Intervention_Scenarios |
| Scenario_Lifestyle | 2000 | 871.40 | 870.74 | 872.05 | Intervention_Scenarios |
| Scenario_Lifestyle | 2001 | 872.31 | 871.66 | 872.95 | Intervention_Scenarios |
| Scenario_Lifestyle | 2002 | 873.10 | 872.46 | 873.74 | Intervention_Scenarios |
| Scenario_Lifestyle | 2003 | 875.70 | 875.06 | 876.33 | Intervention_Scenarios |
| Scenario_Lifestyle | 2004 | 878.90 | 878.27 | 879.52 | Intervention_Scenarios |
| Scenario_Lifestyle | 2005 | 887.13 | 886.51 | 887.74 | Intervention_Scenarios |
| Scenario_Lifestyle | 2006 | 893.13 | 892.52 | 893.74 | Intervention_Scenarios |
| Scenario_Lifestyle | 2007 | 898.07 | 897.47 | 898.67 | Intervention_Scenarios |
| Scenario_Lifestyle | 2008 | 905.22 | 904.62 | 905.81 | Intervention_Scenarios |
| Scenario_Lifestyle | 2009 | 907.99 | 907.40 | 908.57 | Intervention_Scenarios |
| Scenario_Lifestyle | 2010 | 911.15 | 910.57 | 911.73 | Intervention_Scenarios |
| Scenario_Lifestyle | 2011 | 911.39 | 910.83 | 911.96 | Intervention_Scenarios |
| Scenario_Lifestyle | 2012 | 909.83 | 909.27 | 910.39 | Intervention_Scenarios |
| Scenario_Lifestyle | 2013 | 909.55 | 909.00 | 910.10 | Intervention_Scenarios |
| Scenario_Lifestyle | 2014 | 909.28 | 908.74 | 909.82 | Intervention_Scenarios |
| Scenario_Lifestyle | 2015 | 911.87 | 911.34 | 912.41 | Intervention_Scenarios |
| Scenario_Lifestyle | 2016 | 914.35 | 913.83 | 914.88 | Intervention_Scenarios |
| Scenario_Lifestyle | 2017 | 914.88 | 914.36 | 915.39 | Intervention_Scenarios |
| Scenario_Lifestyle | 2018 | 918.27 | 917.76 | 918.78 | Intervention_Scenarios |
| Scenario_Lifestyle | 2019 | 918.01 | 917.50 | 918.51 | Intervention_Scenarios |
| Scenario_Lifestyle | 2020 | 910.77 | 910.28 | 911.27 | Intervention_Scenarios |
| Scenario_Lifestyle | 2021 | 914.59 | 914.10 | 915.08 | Intervention_Scenarios |
| Scenario_Lifestyle | 2022 | 914.33 | 897.74 | 930.92 | Intervention_Scenarios |
| Scenario_Lifestyle | 2023 | 913.39 | 888.65 | 938.12 | Intervention_Scenarios |
| Scenario_Lifestyle | 2024 | 903.18 | 868.42 | 937.95 | Intervention_Scenarios |
| Scenario_Lifestyle | 2025 | 892.97 | 846.69 | 939.25 | Intervention_Scenarios |
| Scenario_Lifestyle | 2026 | 882.95 | 823.83 | 942.08 | Intervention_Scenarios |
| Scenario_Lifestyle | 2027 | 872.93 | 799.68 | 946.19 | Intervention_Scenarios |
| Scenario_Lifestyle | 2028 | 862.97 | 774.61 | 951.32 | Intervention_Scenarios |
| Scenario_Lifestyle | 2029 | 852.94 | 748.70 | 957.18 | Intervention_Scenarios |
| Scenario_Lifestyle | 2030 | 842.89 | 722.04 | 963.75 | Intervention_Scenarios |
| Scenario_Lifestyle | 2031 | 832.99 | 694.74 | 971.25 | Intervention_Scenarios |
| Scenario_Lifestyle | 2032 | 823.05 | 666.57 | 979.52 | Intervention_Scenarios |
| Scenario_Lifestyle | 2033 | 813.14 | 637.77 | 988.50 | Intervention_Scenarios |
| Scenario_Lifestyle | 2034 | 803.22 | 608.40 | 998.03 | Intervention_Scenarios |
| Scenario_Lifestyle | 2035 | 793.33 | 578.53 | 1,008.13 | Intervention_Scenarios |
| Scenario_Lifestyle | 2036 | 783.59 | 548.23 | 1,018.95 | Intervention_Scenarios |
| Scenario_Lifestyle | 2037 | 773.84 | 517.34 | 1,030.34 | Intervention_Scenarios |
| Scenario_Lifestyle | 2038 | 764.16 | 486.02 | 1,042.29 | Intervention_Scenarios |
| Scenario_Lifestyle | 2039 | 754.51 | 454.33 | 1,054.68 | Intervention_Scenarios |
| Scenario_Lifestyle | 2040 | 744.90 | 422.30 | 1,067.49 | Intervention_Scenarios |
| Scenario_Pessimistic | 1990 | 891.27 | 890.52 | 892.02 | Intervention_Scenarios |
| Scenario_Pessimistic | 1991 | 881.53 | 880.80 | 882.27 | Intervention_Scenarios |
| Scenario_Pessimistic | 1992 | 872.78 | 872.06 | 873.50 | Intervention_Scenarios |
| Scenario_Pessimistic | 1993 | 870.82 | 870.11 | 871.53 | Intervention_Scenarios |
| Scenario_Pessimistic | 1994 | 870.19 | 869.49 | 870.89 | Intervention_Scenarios |
| Scenario_Pessimistic | 1995 | 871.47 | 870.78 | 872.17 | Intervention_Scenarios |
| Scenario_Pessimistic | 1996 | 871.81 | 871.12 | 872.49 | Intervention_Scenarios |
| Scenario_Pessimistic | 1997 | 868.82 | 868.15 | 869.50 | Intervention_Scenarios |
| Scenario_Pessimistic | 1998 | 866.97 | 866.30 | 867.63 | Intervention_Scenarios |
| Scenario_Pessimistic | 1999 | 869.96 | 869.30 | 870.62 | Intervention_Scenarios |
| Scenario_Pessimistic | 2000 | 871.40 | 870.74 | 872.05 | Intervention_Scenarios |
| Scenario_Pessimistic | 2001 | 872.31 | 871.66 | 872.95 | Intervention_Scenarios |
| Scenario_Pessimistic | 2002 | 873.10 | 872.46 | 873.74 | Intervention_Scenarios |
| Scenario_Pessimistic | 2003 | 875.70 | 875.06 | 876.33 | Intervention_Scenarios |
| Scenario_Pessimistic | 2004 | 878.90 | 878.27 | 879.52 | Intervention_Scenarios |
| Scenario_Pessimistic | 2005 | 887.13 | 886.51 | 887.74 | Intervention_Scenarios |
| Scenario_Pessimistic | 2006 | 893.13 | 892.52 | 893.74 | Intervention_Scenarios |
| Scenario_Pessimistic | 2007 | 898.07 | 897.47 | 898.67 | Intervention_Scenarios |
| Scenario_Pessimistic | 2008 | 905.22 | 904.62 | 905.81 | Intervention_Scenarios |
| Scenario_Pessimistic | 2009 | 907.99 | 907.40 | 908.57 | Intervention_Scenarios |
| Scenario_Pessimistic | 2010 | 911.15 | 910.57 | 911.73 | Intervention_Scenarios |
| Scenario_Pessimistic | 2011 | 911.39 | 910.83 | 911.96 | Intervention_Scenarios |
| Scenario_Pessimistic | 2012 | 909.83 | 909.27 | 910.39 | Intervention_Scenarios |
| Scenario_Pessimistic | 2013 | 909.55 | 909.00 | 910.10 | Intervention_Scenarios |
| Scenario_Pessimistic | 2014 | 909.28 | 908.74 | 909.82 | Intervention_Scenarios |
| Scenario_Pessimistic | 2015 | 911.87 | 911.34 | 912.41 | Intervention_Scenarios |
| Scenario_Pessimistic | 2016 | 914.35 | 913.83 | 914.88 | Intervention_Scenarios |
| Scenario_Pessimistic | 2017 | 914.88 | 914.36 | 915.39 | Intervention_Scenarios |
| Scenario_Pessimistic | 2018 | 918.27 | 917.76 | 918.78 | Intervention_Scenarios |
| Scenario_Pessimistic | 2019 | 918.01 | 917.50 | 918.51 | Intervention_Scenarios |
| Scenario_Pessimistic | 2020 | 910.77 | 910.28 | 911.27 | Intervention_Scenarios |
| Scenario_Pessimistic | 2021 | 914.59 | 914.10 | 915.08 | Intervention_Scenarios |
| Scenario_Pessimistic | 2022 | 914.33 | 897.74 | 930.92 | Intervention_Scenarios |
| Scenario_Pessimistic | 2023 | 913.39 | 888.65 | 938.12 | Intervention_Scenarios |
| Scenario_Pessimistic | 2024 | 912.30 | 877.19 | 947.42 | Intervention_Scenarios |
| Scenario_Pessimistic | 2025 | 911.10 | 863.88 | 958.32 | Intervention_Scenarios |
| Scenario_Pessimistic | 2026 | 982.78 | 916.97 | 1,048.59 | Intervention_Scenarios |
| Scenario_Pessimistic | 2027 | 981.44 | 899.08 | 1,063.80 | Intervention_Scenarios |
| Scenario_Pessimistic | 2028 | 980.04 | 879.69 | 1,080.38 | Intervention_Scenarios |
| Scenario_Pessimistic | 2029 | 978.43 | 858.85 | 1,098.01 | Intervention_Scenarios |
| Scenario_Pessimistic | 2030 | 976.67 | 836.64 | 1,116.71 | Intervention_Scenarios |
| Scenario_Pessimistic | 2031 | 974.95 | 813.14 | 1,136.77 | Intervention_Scenarios |
| Scenario_Pessimistic | 2032 | 973.04 | 788.05 | 1,158.04 | Intervention_Scenarios |
| Scenario_Pessimistic | 2033 | 971.04 | 761.62 | 1,180.46 | Intervention_Scenarios |
| Scenario_Pessimistic | 2034 | 968.88 | 733.88 | 1,203.87 | Intervention_Scenarios |
| Scenario_Pessimistic | 2035 | 966.61 | 704.90 | 1,228.33 | Intervention_Scenarios |
| Scenario_Pessimistic | 2036 | 964.39 | 674.72 | 1,254.06 | Intervention_Scenarios |
| Scenario_Pessimistic | 2037 | 962.02 | 643.14 | 1,280.89 | Intervention_Scenarios |
| Scenario_Pessimistic | 2038 | 959.57 | 610.31 | 1,308.84 | Intervention_Scenarios |
| Scenario_Pessimistic | 2039 | 957.03 | 576.28 | 1,337.77 | Intervention_Scenarios |
| Scenario_Pessimistic | 2040 | 954.38 | 541.06 | 1,367.70 | Intervention_Scenarios |

**Table S14. The sensitivity analysis results of the BAPC model under different scenarios(ASPR)**

| **Category** | **Year** | **Scenarios**  **count** | **Mean projection** | **Uncertainty range** |
| --- | --- | --- | --- | --- |
| Baseline | 2022 | 1 | 914.33 | 0.00 |
| Baseline | 2023 | 1 | 913.39 | 0.00 |
| Baseline | 2024 | 1 | 912.30 | 0.00 |
| Baseline | 2025 | 1 | 911.10 | 0.00 |
| Baseline | 2026 | 1 | 909.98 | 0.00 |
| Baseline | 2027 | 1 | 908.74 | 0.00 |
| Baseline | 2028 | 1 | 907.44 | 0.00 |
| Baseline | 2029 | 1 | 905.95 | 0.00 |
| Baseline | 2030 | 1 | 904.33 | 0.00 |
| Baseline | 2031 | 1 | 902.73 | 0.00 |
| Baseline | 2032 | 1 | 900.97 | 0.00 |
| Baseline | 2033 | 1 | 899.11 | 0.00 |
| Baseline | 2034 | 1 | 897.11 | 0.00 |
| Baseline | 2035 | 1 | 895.01 | 0.00 |
| Baseline | 2036 | 1 | 892.96 | 0.00 |
| Baseline | 2037 | 1 | 890.76 | 0.00 |
| Baseline | 2038 | 1 | 888.50 | 0.00 |
| Baseline | 2039 | 1 | 886.14 | 0.00 |
| Baseline | 2040 | 1 | 883.68 | 0.00 |
| Model_Sensitivity | 2022 | 3 | 905.01 | 45.18 |
| Model_Sensitivity | 2023 | 3 | 906.39 | 52.59 |
| Model_Sensitivity | 2024 | 3 | 907.40 | 59.21 |
| Model_Sensitivity | 2025 | 3 | 907.69 | 64.15 |
| Model_Sensitivity | 2026 | 3 | 908.60 | 70.66 |
| Model_Sensitivity | 2027 | 3 | 909.24 | 76.32 |
| Model_Sensitivity | 2028 | 3 | 910.19 | 82.75 |
| Model_Sensitivity | 2029 | 3 | 910.79 | 88.46 |
| Model_Sensitivity | 2030 | 3 | 910.77 | 92.66 |
| Model_Sensitivity | 2031 | 3 | 911.28 | 98.27 |
| Model_Sensitivity | 2032 | 3 | 911.40 | 102.83 |
| Model_Sensitivity | 2033 | 3 | 911.76 | 108.02 |
| Model_Sensitivity | 2034 | 3 | 911.88 | 112.69 |
| Model_Sensitivity | 2035 | 3 | 911.49 | 116.09 |
| Model_Sensitivity | 2036 | 3 | 911.57 | 120.73 |
| Model_Sensitivity | 2037 | 3 | 911.25 | 124.32 |
| Model_Sensitivity | 2038 | 3 | 911.16 | 128.52 |
| Model_Sensitivity | 2039 | 3 | 910.94 | 132.42 |
| Model_Sensitivity | 2040 | 3 | 910.23 | 135.12 |
| Intervention_Scenarios | 2022 | 4 | 914.33 | 0.00 |
| Intervention_Scenarios | 2023 | 4 | 913.39 | 0.00 |
| Intervention_Scenarios | 2024 | 4 | 910.02 | 9.12 |
| Intervention_Scenarios | 2025 | 4 | 862.43 | 164.00 |
| Intervention_Scenarios | 2026 | 4 | 874.22 | 236.60 |
| Intervention_Scenarios | 2027 | 4 | 867.70 | 236.27 |
| Intervention_Scenarios | 2028 | 4 | 861.16 | 235.93 |
| Intervention_Scenarios | 2029 | 4 | 854.48 | 235.55 |
| Intervention_Scenarios | 2030 | 4 | 847.71 | 235.13 |
| Intervention_Scenarios | 2031 | 4 | 841.01 | 234.71 |
| Intervention_Scenarios | 2032 | 4 | 834.18 | 234.25 |
| Intervention_Scenarios | 2033 | 4 | 827.32 | 233.77 |
| Intervention_Scenarios | 2034 | 4 | 820.37 | 233.25 |
| Intervention_Scenarios | 2035 | 4 | 813.37 | 232.70 |
| Intervention_Scenarios | 2036 | 4 | 806.46 | 232.17 |
| Intervention_Scenarios | 2037 | 4 | 799.45 | 231.60 |
| Intervention_Scenarios | 2038 | 4 | 792.44 | 242.11 |
| Intervention_Scenarios | 2039 | 4 | 785.38 | 253.66 |
| Intervention_Scenarios | 2040 | 4 | 778.29 | 265.11 |
